# Streamlining SuFEx Inhibitor Development: A Unified Approach Using Photolabile and Orthogonal Sulfinate Protecting Groups

**DOI:** 10.1002/anie.202505984

**Published:** 2025-05-19

**Authors:** Twinkle I. Patel, Makayla L. Williams, Yumeng Chi, Ramkrishna Laha, Matthew J. Moschitto

**Affiliations:** ^1^ Department of Medicinal Chemistry Ernest Mario School of Pharmacy Rutgers, the State University of New Jersey 163 Frelinghuysen Road Piscataway New Jersey USA

**Keywords:** Drug discovery, Organosulfur chemistry, Photochemistry, Sulfonyl fluorides

## Abstract

Sulfonyl fluorides (SFs) have gained significant importance due to their classification as a click reaction and therefore have seen increased use in drug discovery and biochemistry. Their use, however, is complicated by the methods by which they are synthesized and their general synthetic instability. This results in SFs being introduced late in a synthetic route with minimal structural diversity. Masking the reactivity of a sulfonyl fluoride by protecting the parent sulfinate is one method to ameliorate these issues. Current sulfinate protecting groups (SPGs), however, possess limited stability. This study outlines the discovery and selection of SPGs based on their overall stability, ease of synthesis, and simple deprotection conditions. This includes the discovery of two novel, photolabile SPGs, *para*‐methoxybenzyl Rongalite and *ortho*‐nitrobenzyl Rongalite that can be directly converted to the sulfonyl fluoride using light and selectfluor. Along with known SPG, 2‐trimethylsilylethyl sulfone (SES), all three SPGs were found to possess broad stability when exposed to numerous common synthetic conditions and are easily coupled to aryl halides from their sulfinate salt precursor. Their utility is then demonstrated in the synthesis of SPG containing building blocks that are incorporated into several bioactive small molecules.

## Graphical abstract

This Study outlines the discovery and selection of sulfinate protecting groups (SPGs) based on their overall stability, ease of synthesis, and simple deprotection conditions, including two novel, photolabile SPGs that can be directly converted to the sulfonyl fluoride (SFs) using light and selectfluor. This streamlined approach to SF synthesis allows for their incorporation at any point in a synthetic endeavor, enabling access to complex SF‐containing small molecules.

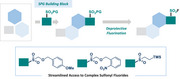

## Introduction

**Figure 1:**
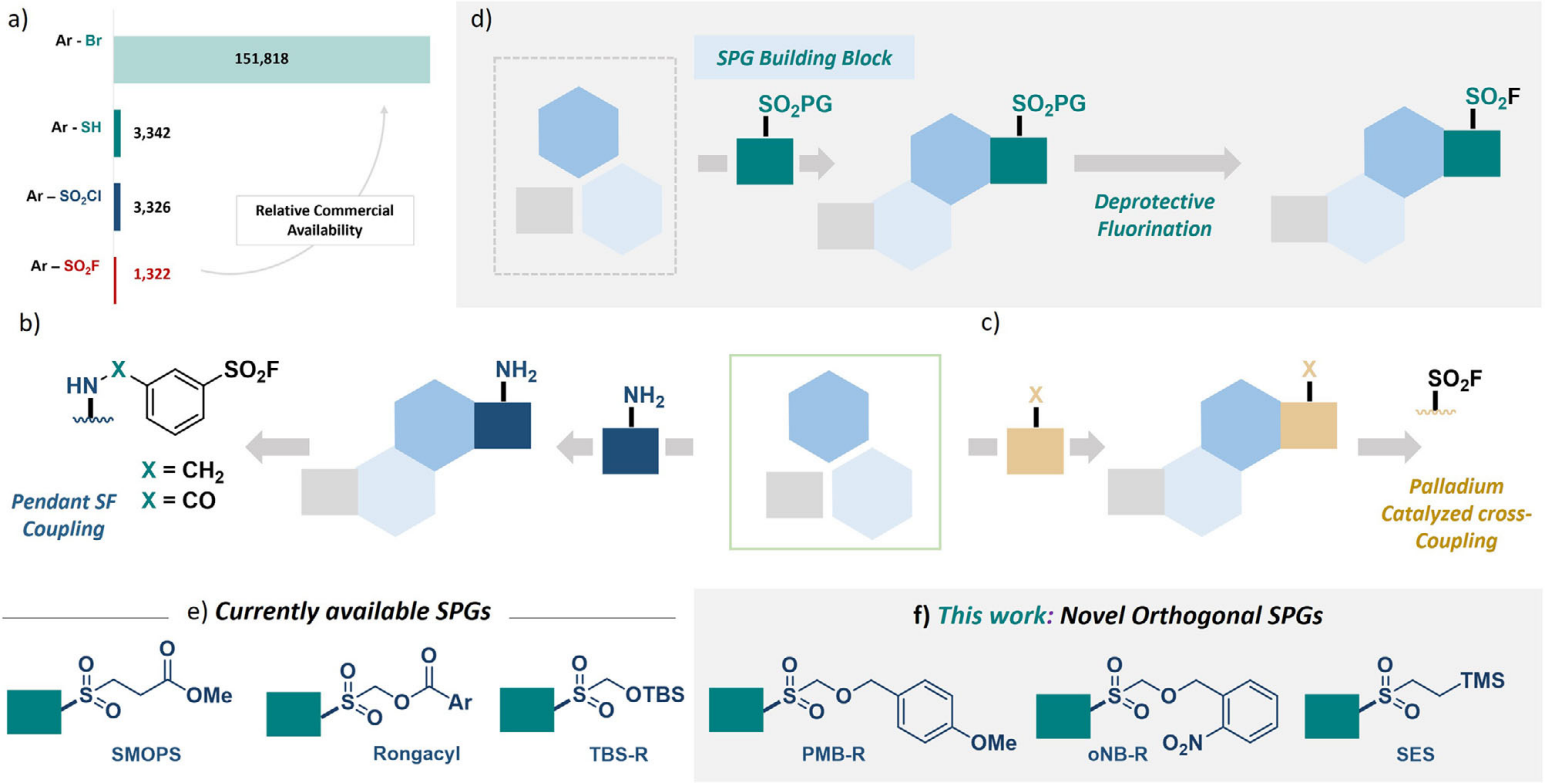
a) Commercial availability of potential organosulfur substrates as determined from Reaxys. b) The current paradigm in SF synthesis is via late‐stage pendant coupling, where a preformed aryl SF is coupled to a core structure at the end of the synthesis. c) Late‐Stage direct SF functionalization offers an alternative in which a functionality (e.g., aryl bromide) is carried through multiple steps and then converted to the SF via coupling. d) The application of SPG containing Building Blocks. e) Chemical structures of currently available SPGs. f) This work: novel orthogonal SPGs.

Over the past decade, sulfonyl fluorides (SFs) have gained significant attention due to their “click‐like” reactivity enabled by sulfur fluoride exchange (SuFEx) chemistry.^[^ ^1^, ^2^, ^3^, ^4^ ^]^ SFs serve as a versatile synthon for accessing other sulfur(VI) functionalities, such as sulfonamides, and are widely used in the development of biochemical probes and protein inhibitors.^[^ ^4^, ^5^, ^6^, ^7^, ^8^, ^9^, ^10^, ^11^, ^12^, ^13^, ^14^, ^15^ ^]^ Most organosulfur‐containing small molecules are derived commercially from either the thiol or sulfonyl chloride. These functionalities, however, are less readily available when compared to other common synthons, such as aryl halides (Figure 1a). This limited availability restricts access to a broader chemical space, thereby hindering drug discovery efforts. Improving access to sulfur containing small molecules from widely accessible starting materials is therefore of high importance.

Although SFs contain enhanced stability under physiological conditions,^[^ ^16^, ^17^ ^]^ their use is often complicated by their incompatibility with various common synthetic conditions such as nucleophiles, base, and high temperature. This results in SFs being incorporated onto a core scaffold late in the synthetic route. There are two main approaches for introducing SFs in a synthetic route: first, a premade aryl SF is often coupled to a pendant group (such as an amine) through amide bond formation or amine alkylation at the end of a synthetic route (Figure 1b). This approach leads to a pendant SF that is exposed and, therefore, susceptible to off‐target reactivity. Examples of this approach are evident in the synthesis of SF‐containing inhibitors for IAP, MCL‐1, BCL‐6, and HSP27.^[^ ^11^, ^12^, ^13^, ^14^, ^18^ ^]^ Although these approaches offers high yields, they rely on the commercial availability of the aryl sulfonyl halide or thiol, thus restricting the functional diversity possible. The second option is direct late‐stage SF functionalization whereby a functionality is carried through various steps and then coupled with a sulfur dioxide equivalent to form the SF (Figure 1c). Many efforts have been made to advance this area including the direct coupling of aryl halides and diazonium salts to sulfur dioxide surrogate, DABSO, under transition metal catalysis and various photocatalytic methods.^[^ ^19^, ^20^, ^21^ ^]^ These efforts usually result in sulfinate salts that can then be oxidized to the SF using selectfluor or N‐fluorobenzenesulfonimide (NFSI). In these cases, although the late‐stage installation is elegant, the parent starting material (for example an aryl bromide) must be introduced late stage and/or be compatible with previous synthetic steps.

Sulfinate protecting groups (SPGs) represent a promising approach to overcome the limitations associated SF synthetic instability and lack of structural diversity. The protected sulfinate can be carried through the necessary synthetic transformations and then subsequently deprotected and coupled to more abundant starting materials such as aryl halides (Figure 1d). This would allow for not only greater structural diversity but also permit a more combinatorial approach to developing varied SF‐containing structures through the use of SPG building blocks. Currently, three common SPGs are used (Figure 1e). Most common is 3‐methoxy‐3‐oxopropane‐1‐sulfone (SMOPS)^[^ ^22^ ^]^ and its newly disclosed t‐butyl analogue STOPS,^[^ ^23^ ^]^ which undergoes deprotection to the sulfinate in basic conditions. More recently, Rongacyl^[^ ^24^ ^]^ and TBS‐Rongalite (TBS‐R)^[^ ^25^ ^]^ have also been introduced and undergo deprotection with hydroxide and fluoride, respectively. As with any protecting group, high stability and orthogonal deprotection conditions are required. In the course of our laboratory's efforts towards the synthesis of SF‐containing small molecule inhibitors, we found that sulfones bearing all three known SPGs had low stability in many common synthetic transformations. Thus, in this study, a comprehensive analysis of SPG synthesis and stability was undertaken. This ultimately resulted in the discovery of three additional SPGs with broad stability and unique, selective deprotection conditions.

## Results and Discussion

### Synthesis of aryl SFs

In addition to SMOPS, Rongacyl, and TBS‐R, three additional SPGs were envisioned. *Para*‐methoxybenzyl‐Rongalite (PMB‐R) and *ortho*‐nitrobenzyl Rongalite (oNB‐R) were intended to be deprotected under oxidizing conditions and light, respectively, while 2‐trimethylsilylethylsulfone (SES) would be deprotected with fluoride (Figure 1f). SES is a common protecting group for sulfonamides; however, its use as a SPG is much rarer with only a few reports showing its use in forming sulfinates.^[^ ^26^, ^27^ ^]^ Combined with the already known protecting groups SMOPS, Rongacyl, and TBS‐R, PMB‐R, oNB‐R, and SES should provide orthogonal deprotection conditions and stability profiles.

**Figure 2:**
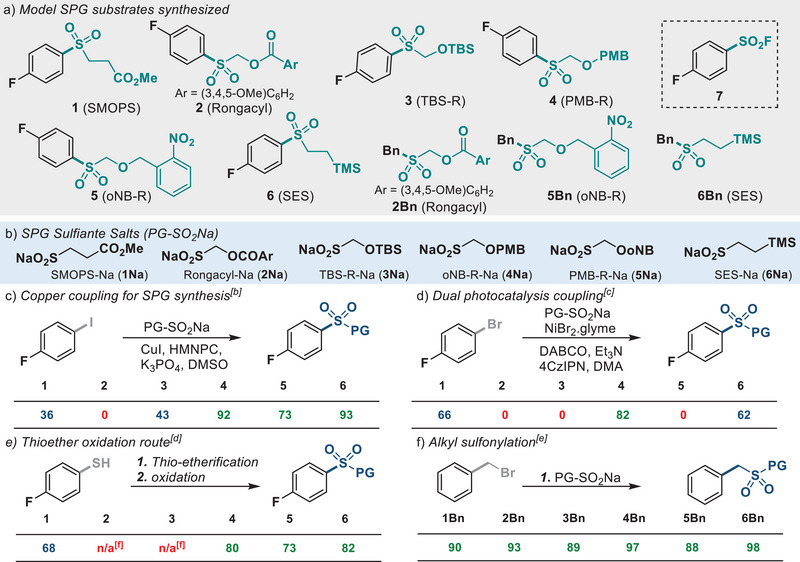
a) Model SPG substrates used in SPG synthesis and stability studies; b) isolated yield of copper coupling conditions, conditions: 4‐fluoroiodobenzene (0.23 mmol, 1 equiv), PG‐SO_2_Na (1.5–3 equiv), CuI (6‐10 mol %), (2*S*,4*R*)‐4‐hydroxy‐*N*‐(2‐methylnaphthalen‐1‐yl)pyrrolidine‐2‐carboxamide (HMNPC, 6–10 mol %), K_3_PO_4_ (1 equiv), DMSO, 50 °C, 24 h; c) isolated yields of dual photocatalysis, conditions: 4‐fluoroiodobenzene (0.1 mmol, 1 equiv), PG‐SO_2_Na (2 equiv), NiBr_2_DME (5 mol %), 4CzIPN (0.2 mol %), DABCO (2.2 equiv), Et_3_N (0.5 equiv), DMA, blue LED, 24 h; d) isolated yields of two‐step oxidation sequence, conditions: (1) see Supporting Information for alkylation (2) *m*‐CPBA (3 equiv), CH_2_Cl_2_, 23 °C, 12 h, or Na_2_WO_4_ (0.5 equiv), H_2_O_2_ (5 equiv), MeOH, 23 °C, 24 h; e) isolated yields  of alkylation, conditions: BnBr (0.29 mmol, 1 equiv),  PG‐SO_2_Na (1.5 equiv), DMSO, 23 °C, 24 h. f) not applicable due to lack of access to the corresponding alkyl halide.

The use of a copper catalyst and the proline‐derived ligand, (2*S*,4*R*)‐4‐hydroxy‐*N*‐(2‐methylnaphthalen‐1‐yl)pyrrolidine‐2‐carboxamide (HMNPC) has been used in the S‐arylation of sulfinates with aryl iodides under mild conditions.^[^ ^28^ ^]^ The sulfinate salts, SMOPS‐Na (**1Na**), Rongacyl‐Na (**2Na**), and TBS‐R‐Na, (**3Na**) are all known (Figure 2b)^[^ ^22^, ^24^, ^25^ ^]^ while the sulfinate salts PMB‐R‐Na (**4Na**) and oNB‐R‐Na (**5Na**) were made from the corresponding chloromethyl ether in a facile manner on decagram scale (see Supporting Information for synthesis). The resulting solids were shelf stable (2+ months), non‐hygroscopic powders. SES sulfinate (SES‐Na, **6Na**) was made from vinyltrimethylsilane according to a modified literature procedure (See Supporting Information).^[^ ^26^, ^29^ ^]^ All reactions proceeded in high yield. PMB‐R‐Na (**4Na**), oNB‐R‐Na (**5Na**), and SES‐Na (**6Na**) sulfinates coupled with aryl iodides in high yields at 50 °C in the presence of CuI/HMNPC (Figure 2c). In contrast, SMOPS‐Na (**1Na**), Rongacyl‐Na (**2Na**), and TBS‐R‐Na (**3Na**) resulted in either low yields or no product. It is worth noting that higher yields for the coupling of TBS‐R‐Na (**3Na**) was obtainable when the aryl iodide was held in excess (3 equiv) as per the original literature report.^[^ ^25^ ^]^ A recent report has also shown that SMOPS‐Na (and its more stable t‐butyl analogue, STOPS‐Na) can couple with aryl iodides in similar yields under copper‐catalyzed conditions.^[^ ^23^ ^]^ Only SES‐Na (**6Na**) coupled with aryl bromides albeit at elevated (100 °C) temperatures. Aryl chlorides and triflates did not couple.

Recently, König reported universal conditions for the coupling of aryl bromides and various nucleophiles, including sulfinates, using nickel and the photocatalyst 4CzIPN.^[^ ^30^ ^]^ SMOPS‐R‐Na (**1Na**), PMB‐R‐Na (**4Na**), and SES‐Na (**6Na**) were effectively coupled with aryl bromides using NiBr_2_(DME), 4CzIPN, and DABCO in 66%, 82%, and 62% yield, respectively (Figure 2d). The PMB‐R‐Na (**4Na**) coupling required air‐free conditions, while SES‐Na (**6Na**) required the presence of oxygen (air). oNB‐R‐Na (**5Na**) failed to engage, and Rongacyl‐Na (**2Na**) degraded. This photocatalytic method provides a straightforward method to access protected sulfinates from the much more readily accessible aryl bromide. Efforts are ongoing to further the utility of this reaction.

It is also possible to access the protected aryl sulfones from the thiol via S‐alkylation and subsequent oxidation with sodium tungstate (Figure 2e). Aryl PMB‐R and oNB‐R sulfone functionalities can all be made via this route in high yield. SES and SMOPS sulfone functionalities are made via the addition of the thiol to the vinyl trimethylsilane and methyl acrylate with AIBN, respectively (See Supporting Information). TBS‐R and Rongacyl sulfones cannot be accessed via this method due to the inability to obtain the corresponding alkyl halide. To access alkyl sulfones bearing SPGs, benzyl bromide was reacted with the corresponding sulfinate in high yield (Figure 2f).

**Figure 3:**
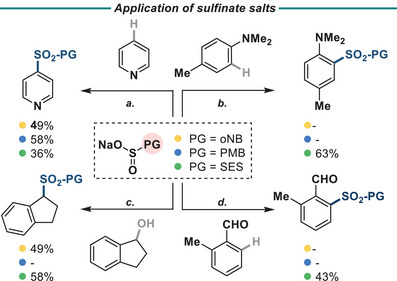
Summary of sulfinate reactions. Yields refer to isolated yield with corresponding sulfinate (PG‐SO_2_Na). Reagents and conditions: a) (i) Tf_2_O (1.1 equiv) (ii) N‐methyl piperidine (3.2 equiv), (iii) PG‐SO_2_Na (1.3 equiv), CH_2_Cl_2_; (b) PG‐SO_2_Na (5 equiv), [Ir(dF(CF_3_)ppy)_2_(dtbpy)]PF_6_ (1 mol%), K_2_S_2_O_8_ (3 equiv), Bu_4_NHSO_4_ (0.2 equiv), MeCN:H_2_O (10:1) Blue LEDs, 72 h; c) PG‐SO_2_Na (1.5 equiv), FeCl_3_ (15 mol %), TMSCl (1.2 equiv), CH_2_Cl_2_ 50 °C, 18 h; d) R‐SO_2_Na, β‐alanine, CuF_2_, Cu(OAc)_2_, K_2_CO_3_, HFIP 100 °C, 18 h.

These sulfinate reagents are also competent in numerous sulfinate‐based chemistries (Figure 3). SES‐Na (**6Na**) is compatible Willis’ photocatalytic sulfonylation of aniline^[^ ^31^ ^]^ or Bull's sulfonylation of benzaldehyde.^[^ ^32^ ^]^ SES‐Na (**6Na**) and oNB‐R‐Na (**5Na**) are compatible in Shreedhar's iron‐catalyzed sulfonylation of alcohols,^[^ ^33^ ^]^ while PMB‐R‐Na (**4Na**), oNB‐R‐Na (**5Na**) and SES‐Na (**6Na**) are all compatible with Manolikakes’ *para*‐sulfonylation of pyridines using Tf_2_O.^[^ ^34^ ^]^

### Stability of SPGs

As with any protecting group, broad stability is required. To provide insight for their use in any synthetic endeavour, the stability of SPGs was investigated in a variety of synthetic conditions. The top ten most‐used synthetic transformations in medicinal chemistry were chosen for SPG stability testing (Table 1).^[^ ^35^ ^]^ These conditions included: amide coupling (HATU), S_N_Ar (Et_2_N^i^Pr, 110 °C), acidic Boc deprotection (TFA), ester hydrolysis (NaOH), Suzuki coupling (Pd(PPh_3_)_4,_ K_3_PO_4_, 110 °C), amine S_N_2 (K_2_CO_3_)_,_ reductive amination (Na(CN)BH_3_), hydrogenation (Pd/C, H_2_), and Buchwald‐Hartwig amination (Pd_2_(dba)_3_, P(o‐tolyl)_3_, NaO^t^Bu, 100 °C). Model aryl sulfones **1**–**6** and benzyl sulfones **2Bn**, **5Bn**, and **6Bn** were added to the reaction mixture for a certain transformation, and upon completion of the reaction, the percentage of recovered aryl sulfone was calculated. Sulfonyl fluoride **7** was also subjected to these same conditions. The conditions chosen were the most common conditions found for each transformation. It is possible that in optimizing a reaction, one may find specific conditions that are less harsh and therefore result in greater retention of the SPG. In each transformation, the reaction itself proceeded in greater than 70% yield unless otherwise noted (see Supporting Information).

**Table 1:** SPG stability study.

		Percent protected sulfinate compound recovered^b)^								
Reaction	Conditions^a)^	**1**	**3**	**4**	**5**	**6**	**2Bn**	**5Bn**	**6Bn**	**7**
		*SMOPS*	*TBS‐R*	*PMB‐R*	*oNB‐R*	*SES*	*Rong*.	*oNB‐R*	*SES*	*SF*
Amide Coupling	RCOOH, RNH_2_, HATU, DIPEA, 23 °C, 24 h	100	100	100	100	100	88	100	100	73
S_N_Ar	Het‐X, R‐NH_2_, DIPEA, 110 °C, 16 h	11	0	57	48	100	3	52	100	0
Boc Deprotection	BocNHR, TFA, 23 °C, 12 h	100	94	0	100	100	100	100	100	68
Ester hydrolysis	RCO_2_Me, NaOH, 23 °C, 12 h	0	0	100	100	100	0	92	100	0
Suzuki–Miyaura	ArI, ArB(OH)_2_, Pd(PPh_3_)_4_, K_3_PO_4_, 110 °C, 12 h	0	41	51	82	100	33	76	100	0
Amine S_N_2	R_2_NH, Alk‐Br, K_2_CO_3_, 23 °C, 2 h	100	100	100	100	100	100	100	100	100
Reductive Amination	R‐CHO, R‐NH_2_, Na(CN)BH_3_, 23 °C 18 h	82	100	100	100	100	0	54	100	0
Hydrogenation	Olefin, H_2_, Pd/C, 23 °C, 12 h	100	100^c)^	100^c)^	52	100	90	50	100	100
Buchwald–Hartwig	ArI, R_2_NH, Pd_2_(dba)_3_, NaO^t^Bu, 100 °C 24 h	0	0	35	10	100	0	9	100	0

^a)^ See supporting information for full conditions; reaction yield was greater than 70% unless otherwise noted.

^b)^ Yields refer to percent protected sulfinate recovered as calculated from ^19^F NMR or ^1^H with an internal standard.

^c)^ Parent reaction did not yield a product.

Overall, all SPGs were stable under mild reaction conditions such as amide coupling, amine S_N_2, and acidic Boc deprotection. Similarly, under reductive amination and hydrogenation conditions, most SPGs demonstrated high stability. The oNB‐R protecting group (**5** and **5a**) was partially reduced to the aniline under hydrogenation conditions and did not hydrolyze. It is worth noting that under hydrogenation conditions, the PMB‐R (**4**) protecting group was stable. In reactions with harsher conditions, such as SN_Ar_, Suzuki coupling, and ester hydrolysis, PMB‐R, oNB‐R, and SES (**4**, **5**, and **6**) were superior to known SPGs, which degraded under these conditions. Only the SES protecting group was stable under Buchwald‐Hartwig coupling conditions with potassium *tert*‐butoxide as base. Stability for this reaction, however, is highly dependent on conditions. For example, switching the base to cesium carbonate and xantphos as ligand resulted in a higher retention of oNB‐R, **5**. As a point of comparison, the parent sulfonyl fluoride (**7**) only survived S_N_2, acidic deprotection, and amide coupling, highlighting the need for SPGs.

### Deprotection

Deprotection of TBS‐R (**3**) and Rongacyl (**2Bn**) were accomplished according to their literature methods.^[^ ^24^, ^25^ ^]^ TBS‐R (**3**) has the benefit of being a one‐pot deprotection (CsF, Selectfluor). SMOPS (**1**) and Rongacyl (**2Bn**) require a two‐step procedure with deprotection via sodium hydride (SMOPS) and sodium hydroxide (Rongacyl) followed by workup and selectfluor addition. Given that PMB groups are generally deprotected under oxidizing conditions, we first looked for methods that would allow for selectfluor to act in a dual oxidation‐fluorination manner.

**Figure 4:**
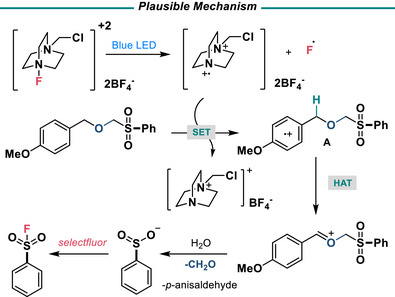
Mechanism of action.

Photocatalytic deprotections of PMB groups are known under oxidative conditions with trichlorobromomethane.^[^ ^36^ ^]^ Conversely, selectfluor, when irradiated, can form the 1,4‐ diazoniabicyclo[2.2.2]octane (TEDA) radical cation, which can undergo single electron transfer and subsequent HAT with PMB groups.^[^ ^37^, ^38^, ^39^, ^40^ ^]^ This SET‐HAT mechanism in the presence of water could therefore be an avenue for deprotection of the PMB‐R SPG (Figure 4). Under irradiation in the presence of 3 equiv of selectfluor, deprotection and fluorination of **8** was able to occur in 89% yield (Table 2, entry 1). These conditions provide a direct, metal‐free method to mildly deprotect and fluorinate PMB‐R‐protected aryl sulfones. Lower yields were obtained when 1 or 2 equivalents of selectfluor were used (Table 2, entries 2 and 3). Switching to NFSI, the absence of selectfluor, or the addition of TEMPO resulted in little to no product (Table 2, entries 4, 5, and 6). The presence of water is essential for the successful removal of the protecting group (Table S1, entry 9). The most likely mechanism is therefore a radical‐based mechanism involving the 1,40‐diazoniabicyclo[2.2.2]octane (TEDA) radical cation (Figure 4). SET yields intermediate **A,** which undergoes HAT by either another equivalent of the TEDA radical cation or fluoride radical. Subsequent hydrolysis and loss of formaldehyde yields the sulfinate, which is rapidly oxidized to the SF.

**Table 2:** Optimization of PMB‐R deprotection / fluorination^a)^

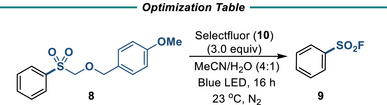		
Entry	Variation from standard conditions	Yield (%)^a)^
1	As above	97 (89%)^b)^
2	1.0 equiv of **10** instead of 3.0 equiv	10
3	2.0 equiv of **10** instead of 3.0 equiv	22
4	NFSI instead of **10**	17
5	No selectfluor	0
6	4.0 equiv TEMPO	0

Conditions: **8** (0.2 mmol), **10** (3.0 equiv), (MeCN:H_2_O (0.2 M), 23 °C, 16 h, N_2_.

^a)^ Yields determined by ^1^H NMR analysis with 4‐fluoroacetophenone as an internal standard.

^b)^ Isolated yields are in parentheses, TEMPO = 2,2,6,6‐tetramethyl‐1‐piperidinyloxy, NFSI = N‐fluorobenzenesulfonimide.

**Figure 5:**
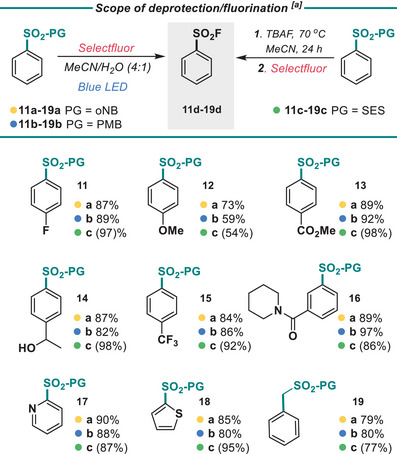
^a)^ Yields refer to isolated yields; yields in brackets refer to NMR yields with 4‐fluoroacetophenone as an internal standard. Conditions for deprotection of **11a‐19a: 11** (0.2 mmol), selectfluor (5.0 equiv), (MeCN:H_2_O (0.2 M), 40 °C, 36 h, N_2_; for deprotection of **11b‐19b**: **11b** (0.2 mmol), selectfluor (3.0 equiv), (MeCN:H_2_O (0.2 M), 40 °C, 16 h, N_2_; for deprotection of **11c‐19c**: **11c** (0.2 mmol), TBAF (1.1 equiv, 1 M in THF), THF 70 °C 16 h, then selectfluor (1.2 equiv), MeCN, 23 °C, 1 h. ^b)^ See Supporting Information for detailed conditions.

A small scope was investigated for this deprotection (Figure 5). The deprotection of the PMB was tolerable to both electron‐deficient (**13b** and **15b**) as well as electron‐rich (**12b**) ring systems. Amides were tolerable, as well as pyridine and thiophene heterocycles (**17b** and **18b**). Alcohols were also well tolerated (**14b**). Alkyl benzyl sulfinates were also deprotected cleanly (**19b**).

**Figure 6:**
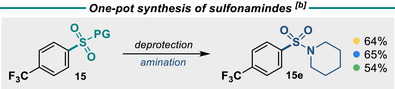
Conditions for amination of **15a**: Et_3_N (5 equiv), piperidine (3 equiv), 85 °C, 16 h; for amination of **15b** and **15c**: Et_3_N (5 equiv), piperidine (3 equiv), 40 °C, 16 h.

Turning to the oNB‐R protecting group, deprotection and fluorination can occur cleanly under irradiation with blue light in the presence of selectfluor. We propose a deprotection mechanism similar to that of a Norrish‐type II process (See Supporting Information for a mechanistic discussion). The scope of this reaction was similarly wide, with high yields for electron‐rich, electron‐poor, and heterocyclic ring systems (Figure 5, **11a**‐**19a**). Finally, the SES protecting group was deprotected using TBAF at 70 °C, followed by cooling and addition of selectfluor. The yield, although slightly lower than the deprotection for oNB‐R and PMB‐R groups, was still high across all substrates tested (**11c**‐**19c**). Although a high temperature of 70 °C is required, fluoride is a weaker base than other common deprotection bases, such as methoxide, and is less nucleophilic. All three protecting groups can be transformed directly into the corresponding sulfonamide by the addition of an amine after fluorination (Figure 6).

## Summary

Overall, considering the synthesis, stability, and deprotection, SES and oNB‐R proved to be the most useful SPGs (Table 3). SES was the most stable across all conditions tested and could be synthesized from the aryl bromide via copper coupling or nickel dual photocatalysis. SES was amenable to most sulfinate coupling chemistry, further demonstrating its utility. SES, however, had harsher deprotection conditions with TBAF at elevated temperatures. oNB‐R, on the other hand, had stability across most conditions and had extremely mild and orthogonal deprotection conditions. PMB‐R was a useful SPG in basic conditions but showed instability under harsher conditions. PMB‐R, however, also has a selective and unique deprotection method and is easily coupled to aryl bromides. Both PMB‐R and oNB‐R are highly useful functionalities given their novel and orthogonal photolabile deprotection and fluorination conditions. All three functionalities outperformed the current known suite of SPGs in synthesis, stability, and deprotection.

**Table 3:** Overview of stability and deprotection/ fluorination of SPGs.

	**1**	**3**	**4**	**5**	**6**	**2Bn**
Stability Ranking^a)^	5	4	3	2	1	6
Deprotection Conditions^b)^	1. NaH 2. **10**	**10** CsF	**10** hν	**10** hν	TBAF 70 °C then **10**	1. NaOH 2. NFSI
One‐pot	**×**	**√**	**√**	**√**	**√**	**×**
Yield^c)^	44%	91%	84%	86%	89%	76%

^a)^ Based off average yield of all 10 reactions from Table 1.

^b)^ See Supporting Information for detailed conditions; temperature of the reaction is 23 °C unless otherwise noted.

^c)^ Yields refer to isolated yield.

### SPGs in Drug Discovery

**Figure 7:**
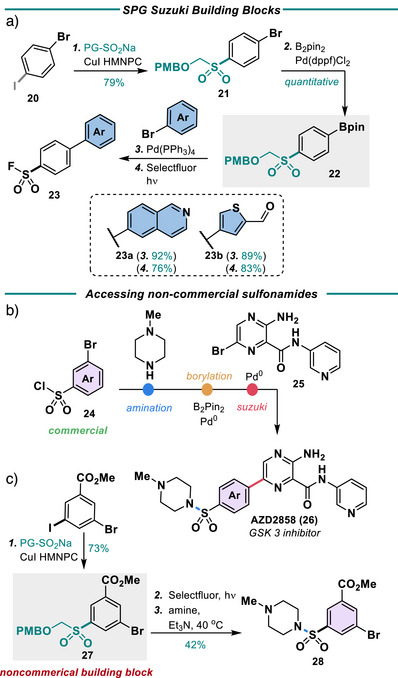
Streaming SuFEx inhibitor development through SPG building blocks (in grey). a) SPG Suzuki building blocks. Reagents and conditions: 1. CuI (10 mol%), HMPNC (10 mol%), PMB‐R‐Na (1.5 equiv), and K_3_PO_4_ (1 equiv), DMSO, 40 °C, 24 h; 2. B_2_Pin_2_ (1.5 equiv), Pd(dppf)Cl_2_ (5 mol%), KOAc (3 equiv), DMSO, 100 °C, 1 h; 3. Aryl halide (1.5 equiv), Pd(PPh_3_)_4_ (5 mol%), Na_2_CO_3_ (3 equiv), DME: H_2_O, 100 °C, 3 h; 4. selectfluor (3 equiv), MeCN: H_2_O_,_ Blue LEDs, 16 h; b) Literature synthesis of AZD2858; c) Access to non‐commercial analogs. Reagents and conditions: 1. CuI (10 mol%), HMPNC (10 mol%), PMB‐R‐Na (1.5 equiv), and K_3_PO_4_ (1 equiv), DMSO, 40 °C, 24 h; 2. selectfluor (3 equiv), MeCN: H_2_O_,_ Blue LEDs, 16 h, then Et_3_N (5 equiv), piperidine (3 equiv), 40 °C, 16 h.

**Figure 8:**
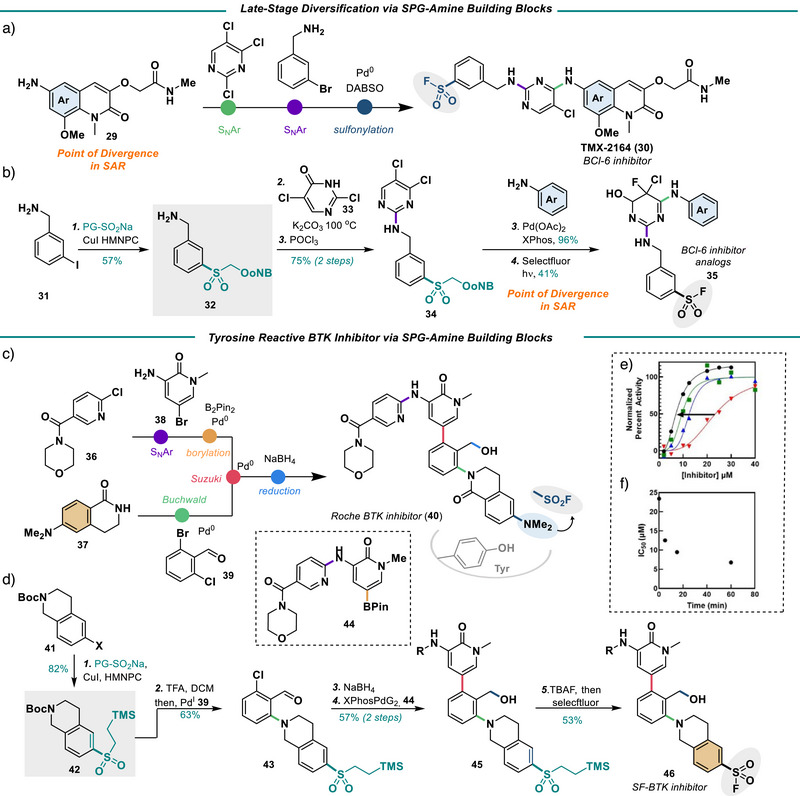
Streamlining SuFEx inhibitors through SPG building blocks; a) Literature synthesis of BCl‐6 inhibitor TMX‐2164; b) Late stage diversification of BCl‐6 inhibitor analog. Reagents and conditions: 1. CuI (10 mol%), HMPNC (10 mol%), oNB‐R‐Na (3 equiv), and K_3_PO_4_ (1 equiv), DMSO, 40 °C, 24 h; 2. **33** (1.2 equiv), DIPEA (1.2 equiv), dioxane, 100 °C, 16 h, then POCl_3_ (3 equiv), 100 °C, 2 h; 3. aniline (2 equiv), Pd(OAc)_2_ (9 mol%), XantPhos (10 mol%), Cs_2_CO_3_ (1.4 equiv), dioxane, 110 °C, 24 h; selectfluor (5 equiv), MeCN: H_2_O_,_ Blue LEDs, 36 h; c) Roche's process route to BTK inhibitor **40**; d) Synthesis of compound **46**. Reagents and conditions: 1. CuI (6 mol%), HMPNC (6 mol%), SES‐Na (1.5 equiv), and K_3_PO_4_ (1 equiv), DMSO, 100 °C, 24 h; 2. TFA:CH_2_Cl_2_ then, **39** (3 equiv), Pd(OAc)_2_ (9 mol%), XantPhos (10 mol%), Cs_2_CO_3_ (1.4 equiv), dioxane, 110 °C, 24 h; 3. **44** (2 equiv), XphosPdG_2_ (5 mol%), K_3_PO_4_ (2 equiv), dioxane:water, 130 °C MW, 90 min; 5. TBAF (10 equiv), MeCN, 90 °C, 16 h, then selectfluor (4 equiv), 23 °C 2 h; e) Dose‐response curve of compound **46** at various preincubation time points: ⬤ = 60 min, ■ = 15 min, ▲ = 5 min, ▼ = 0 min; (f) Plot of IC_50_ and preincubation time.

In drug discovery, the introduction of sulfonamides or SFs often occurs from the sulfonyl chloride or thiol. This narrows the scope of SAR studies to commercially available sulfonyl halides or thiols. Overall, SPGs can be used in drug discovery programs to streamline the synthesis of SFs and sulfonamides through SPG‐containing building blocks (Figures 7 and 8). SPG containing building blocks have an aryl‐protected sulfone with an additional coupling handle. These coupling handles can be applied to numerous synthetic reactions anywhere in a synthetic route. Specifically, SPG‐containing building blocks can be used for late‐stage diversification of common intermediates to expand structural diversity, leading to both complex SFs and sulfonamides. For example, a Suzuki Building Block can be used to form a variety of advanced biaryl substrates (Figure 7a). To demonstrate this, bromoiodobenzene was coupled to the PMB‐R‐Na (**4Na**) to yield building block **21**, which was then borylated. At this point, the boronic ester was coupled to two different aryl bromides via a Suzuki reaction.^[^ ^41^ ^]^ Deprotection yielded the corresponding sulfonyl fluoride, which could be further functionalized if necessary.

In another example, in literature, AZD2858, a GSK3 inhibitor, is synthesized from the parent sulfonyl chloride by first forming the sulfonamide and then subsequently undergoing a Suzuki reaction with N‐methyl pyrazine (Figure 7b). In an SAR study of this molecule, each sulfonamide must be synthesized first from the sulfonyl chloride, borylated, and then finally coupled. The lack of availability of certain thiol or sulfonyl halide ring systems would therefore lead to a restricted coverage of chemical space in the SAR study. We therefore focused on accessing sulfonamides that are not readily available commercially, such as building block **27 (**Figure 7c). As a proof of concept, 3‐bromo‐5‐iodobenzoate was coupled to the PMB‐R‐Na (**4Na**) to yield **27**, which can be readily converted into sulfonamide **28**. At this point, the boronic ester can be coupled via a Suzuki reaction to N‐methyl pyrazine following known procedures.

Similarly, SPG‐containing amine building blocks, which contain an aryl sulfone bearing an SPG and an amine coupling handle, can also be employed as demonstrated in the synthesis of analogs of BCl‐6 inhibitor TMX‐2164 (**30,** Figure 8a). The literature synthesis of TMX‐2164 proceeds via S_N_Ar, Buchwald coupling with the *m*‐bromobenzylamine, and finally sulfonylation via Willis’ palladium‐DABSO conditions (Figure 8a). In the development of SAR for molecule TMX‐2164, one would want to be able to diversify the aryl amine component last; having a SF would not allow for this late stage S_N_Ar. Instead, a benzylamine SPG building block can be employed. Benzyl amine **31** was first coupled to the oNB‐R‐Na **(5Na)** to yield the SPG building block **32**. Subsequent S_N_Ar with chloropyrimidine, followed by chlorination of the hydroxyl group, yielded **34**. No degradation of the SPG was noted during these harsh steps. Finally, Buchwald coupling of aniline yielded the corresponding analog, which was subsequently deprotected and fluorinated to obtain BCl‐6 analog **35** (Figure 7b). In the course of this fluorination, we found that the pyrimidine ring system was also fluorinated through an interesting mechanism resulting from the electron‐donating capability of the additional nitrogen (See Supporting Information); overall, we have found this issue specifically with aminopyrimidine ring systems. While unfortunate for this molecule, the structure nevertheless is an interesting BCl‐6 analog.

Another example of a SPG‐containing amine building block is seen in the synthesis of a tyrosine reactive covalent inhibitor of the enzyme Bruton Tyrosine Kinase (BTK). To demonstrate the utility of these SPGs, a tyrosine reactive covalent inhibitor of the enzyme BTK was developed. BTK is a well‐known target for the treatment of leukaemia and lymphomas and is critical for B‐cell proliferation in cancer.^[^ ^42^, ^43^ ^]^ Currently, all FDA approved BTK inhibitors are covalent inhibitors containing an acrylamide that reacts with Cys481. Mutations, however, of Cys481 have led to resistance to covalent BTK inhibitors^[^ ^44^ ^]^; thus, recently, more reversible inhibitors have entered various stages of clinical trials.^[^ ^42^, ^43^, ^45^, ^46^ ^]^ Given the success of covalent BTK inhibitors, covalent inhibitors or probes that react with tyrosine or lysine residues would be beneficial.

In the crystal structure of compound **40**, Roche's first‐generation reversible BTK inhibitor, the dimethylaniline moiety is found in close proximity to Tyr551 (Figure 8c).^[^ ^47^ ^]^ If the dimethylaniline was converted to a sulfonyl fluoride, reactivity with tyrosine could be possible. Roche's synthesis of **40** contains numerous C‐C and C‐N bond formation steps such as Suzuki coupling, S_N_Ar, and Buchwald‐Hartwig coupling.^[^ ^47^, ^48^ ^]^ These bond‐forming steps would require that the sulfonyl fluoride be installed in the last step; however, this is complicated by the fact that carrying an aryl halide through multiple cross‐coupling steps would also not be feasible. SPGs, therefore, could be ideal for this application.

Amine **41** was chosen over the corresponding amide as a starting material due to the availability of the material and a lack of reactivity of the amide when a *para‐*withdrawing group is present. The SES group was chosen as the SPG over the oNB‐R group due to a higher stability in Buchwald‐Hartwig coupling conditions. SES‐Na was easily coupled to amine **41** in 82% yield (Figure 8d). In a manner similar to the process route, the amine was then coupled to 2‐chloro‐4‐bromobenzaldehyde in the presence of palladium at 110 °C. No degradation of the SPG was observed. The aryl chloride was then subsequently coupled to boronate ester **44** in moderate yield, and the aldehyde was reduced, again with no degradation of the SPG. Finally deprotection/fluorination of the SES group was accomplished with TBAF at 70 °C, followed by selectfluor addition to yield SF **46**. Compound **46** was tested in a BTK kinase glo assay and was found to have an initial IC_50_ of 23 *µ*M (Figure 8e). The IC_50_ decreased as the preincubation time increased, indicating that compound **46** displays irreversible binding behaviour (Figure 8f). Overall, the IC_50_ is quite higher than compound **40** (7 nM), indicating a misalignment in initial binding. Further efforts are ongoing to elaborate the structure of **46** and elucidate the kinetics of its binding and reactivity, which should result in a more potent species. In all cases discussed in this section, the synthesis of these compounds would not have been possible with previously known SPGs, given the harshness of the conditions. This highlights the utility of the proposed photolabile SPGs and the SES protecting group.

## Conclusions

In conclusion, we have undertaken a comprehensive study of SPG stability by subjecting six different SPGs to a variety of common synthetic transformations. Additionally, methods to synthesize aryl containing SPGs were also assessed. From these studies, it was found that 2‐trimethylsilylethylsulfone (SES) and the newly disclosed *ortho*‐nitrobenzyl Rongalite sulfone (oNB‐R) possessed a broad stability profile, a facile method of synthesis, and a selective and orthogonal deprotection/fluorination procedure. P*ara*‐methoxybenzyl Rongalite was also found to have broad stability and an intriguing deprotection/fluorination condition of selectfluor and light. These latter two SPGs are the first examples of photolabile SPGs.

Ultimately, the SPG chosen in a synthetic endeavor should be tailored to the synthetic conditions used and the functionality present. The studies above outline the benefits and drawbacks of each SPG while providing additional groups with novel, orthogonal deprotection conditions. Overall, these groups should allow for a more structural diversity in the synthesis of SF‐containing small molecule inhibitors or probes.

## Author Contributions

T.I.P., R.L., and M.J.M. conceived the program. T.I.P. performed synthesis, stability, and deprotection studies. Y.C. and M.W. aided in sulfinate synthesis. T.I.P. and M.J.M. wrote and contributed to the manuscript.

## Conflict of Interests

A provisional patent application has been filed by Rutgers University, which covers the synthesis and use of benzyloxy sulfinate protecting groups.

## Supporting information



Supporting Information

## Data Availability

The data that support the findings of this study are available in the Supporting Information of this article.
