# Supplementary material for: Streamlining SuFEx Inhibitor Development: A Unified Approach Using Photolabile and Orthogonal Sulfinate Protecting Groups
Source: Angew Chem Int Ed Engl. 2025 May 19;64(27):e202505984. doi: 10.1002/anie.202505984 (PMC12207385; doi:10.1002/anie.202505984)
Supplement: Supplementary file 1 — Supporting Information [file ANIE-64-e202505984-s001.pdf]

Supporting Information

Streamlining SuFEx Inhibitor Development: A Unified Approach Using Photolabile and Orthogonal Sulfinate Protecting Groups

Twinkle I. Patel, Makayla L. Williams, Yumeng Chi, Ramkrishna Laha, and Matthew J. Moschitto*

Department of Medicinal Chemistry, Rutgers, The State University of New Jersey, Piscataway, NJ 08854-8020, USA

Table of Contents

General Information 3
Synthesis of Reagents 3
Sulfone Synthesis: 10
Application of sulfinates: 22
Stability of SPGs: 28
Substrate Scope 31
Deprotection/fluorination of SPGs: 41
oNB-R deprotection mechanism 51
Synthesis of SPG Suzuki Building Blocks 53
Spectra: 68
References: 172

General Information

All chemicals were purchased from Ambeed, Sigma Aldrich, or ThermoFisher Scientific and used without purification. Anhydrous solvents (THF, CH_2Cl_2, DMF, CH_3CN) were purified before use by passing through a column composed of activated alumina and a supported copper redox catalyst. ACS grade MeCN was purchased from Fisher Chemicals and used without purification. Yields refer to chromatographically and spectroscopically (^1H NMR) homogeneous material unless otherwise noted. Analytical thin-layer chromatography (TLC) was performed using Merck Silica Gel 60 Å F-254 precoated plates (0.25 mm thickness), and components were visualized by ultraviolet light (254 nm) and/or by staining with $KMnO_4$ or phosphomolybdic acid. Flash column chromatography was performed on a Teledyne CombiFlash NextGen 300 automated flash purification system with various Teledyne cartridges (4-80 g, 40-63 µm, 60 Å). Purifications were performed with hexanes and ethyl acetate unless otherwise noted. High-performance liquid chromatography purification was performed by using ACCQPrep HP150 system with water acetonitrile as the eluent. ^1H and ^{13}C NMR spectra were recorded on a Bruker Advance-III spectrometer at 400 MHz and 101 MHz, respectively, in $CDCl_3$, MeOD, or DMSO-*d*6. Chemical shifts were reported in ppm; multiplicities are indicated by s = singlet, d = doublet, t = triplet, q = quartet, p = pentet, sep = septet, dd = doublet of doublet, dt = doublet of triplet, dp = doublet of pentet, m = multiplet, br = broad resonance. Coupling constants 'J' were reported in Hz. High-resolution mass spectral data were obtained on a Waters SYNAPT XS (TOF) high resolution mass spectrometer using electron spray ionization (ESI) in the positive ion mode. All photoredox reactions were performed using a Kessil PR160L-Blue LED lamp (30 W, λmax = 440 nm, 100% intensity, 5 cm from the wall of vial) with continuous air flow for cooling. Luminescent assays were performed on a Biotek Synergy HTX multimode plate reader.

Synthesis of Reagents

Synthesis of **Sodium 3-methoxy-3-oxopropane-1-sulfinate (1Na, SMOPS-Na)**

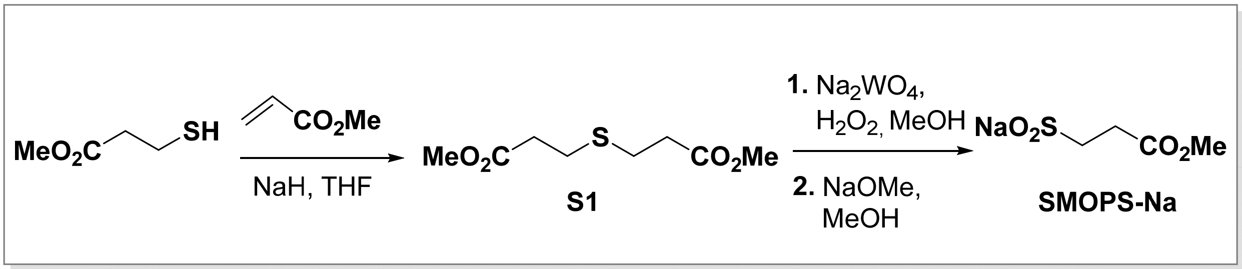

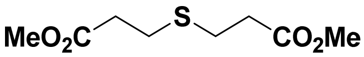

S1

Dimethyl 3,3'-thiodipropionate (S1)

To a solution of 3-mercaptopropionate (2.18 mL, 20.00 mmol) in THF, NaH (960 mg, 24.00 mmol, 60% dispersion in oil, 1.2 equiv) was added portion wise at 0 °C. The reaction mixture was stirred at 0 °C for 30 minutes. Then, methyl acrylate (2.17 mL, 24.00 mmol, 1.2 equiv) was added dropwise at 0 °C and the resulting solution was stirred at room temperature for 4 h. After completion of the reaction, ammonium chloride was added to quench the reaction at 0 °C. The organic layer was extracted with dichloromethane (X3) and the combined organic layers were dried over Na_2SO_4. The solvent was removed under reduced pressure, and the resulting residue was purified by flash chromatography to obtain the desired product as a yellow oil (4.04 g, 98% yield). The spectral data matched that of literature.[1]

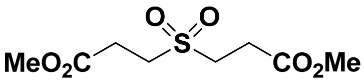

S2

Dimethyl 3,3'-sulfonyldipropionate (S2)

To a solution of **S1** (1.00 g, 4.84 mmol) and Na_2WO_4 (809 mg, 2.42 mmol, 0.5 equiv) in MeOH (50 mL), add H_2O_2 aqueous (2.8 mL, 48.40 mmol, 50% v/v, 10 equiv) dropwise at 0 °C under N_2 atmosphere. After completion of reaction, a solution of $Na_2S_2O_5$ (2 grams) in 25 mL water was added to the reaction mixture at 0 °C. The organic layer was extracted with dichloromethane (X3), and the combined organic layers were washed with brine and then dried over Na_2SO_4. The solvent was removed under reduced pressure, and the resulting residue was then washed with diethyl ether three times to obtain the desired sulfone as a white solid (1.11 g, 96% yield). **^{1}H NMR** (400 MHz, $CDCl_3$) δ 3.74 (s, 6H), 3.35 (t, *J* = 7.4 Hz, 4H), 2.89 (t, *J* = 7.4 Hz, 4H); **^{13}C NMR** (101 MHz, $CDCl_3$) δ 170.9, 52.6, 48.9, 26.9; **HRMS** (ESI-TOF) m/z: $[M+Na]^+$ calc'd for $C_8H_{14}NaO_6S$ 261.0409; found: 261.0412.

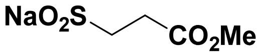

Sodium 3-methoxy-3-oxopropane-1-sulfinate (SMOPS-Na)

To a solution of **S2** (1.00 g, 4.19 mmol) in MeOH (20 mL), NaOMe (0.93 mL, 4.19 mmol, 1 equiv) was added at 0 °C. The reaction mixture was then warmed to room temperature and stirred for one hour. After completion of the reaction, the solvent was removed under reduced pressure,

and the resulting residue was then washed with diethyl ether three times, to obtain the desired product as a white solid (700 mg, 96% yield). The spectral data matched that of literature.[2]

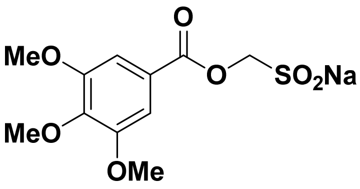

Synthesis of **Sodium ((3,4,5-trimethoxybenzoyl)oxy)methanesulfinate (2Na, Rongacyl-Na)**

Sodium ((3,4,5-trimethoxybenzoyl)oxy)methanesulfinate was synthesized according to the known literature procedure. The spectral data matched that of literature.[3]

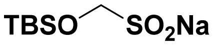

Synthesis of **Sodium ((tert-butyldimethylsilyl)oxy)methanesulfinate (3Na, TBS-R-Na)**

Sodium ((tert-butyldimethylsilyl)oxy)methanesulfinate was synthesized according to the known literature procedure. The spectral data matched that of literature.[4]

Synthesis of **Sodium ((4-methoxybenzyl)oxy)methanesulfinate (4Na, PMB-R-Na)**

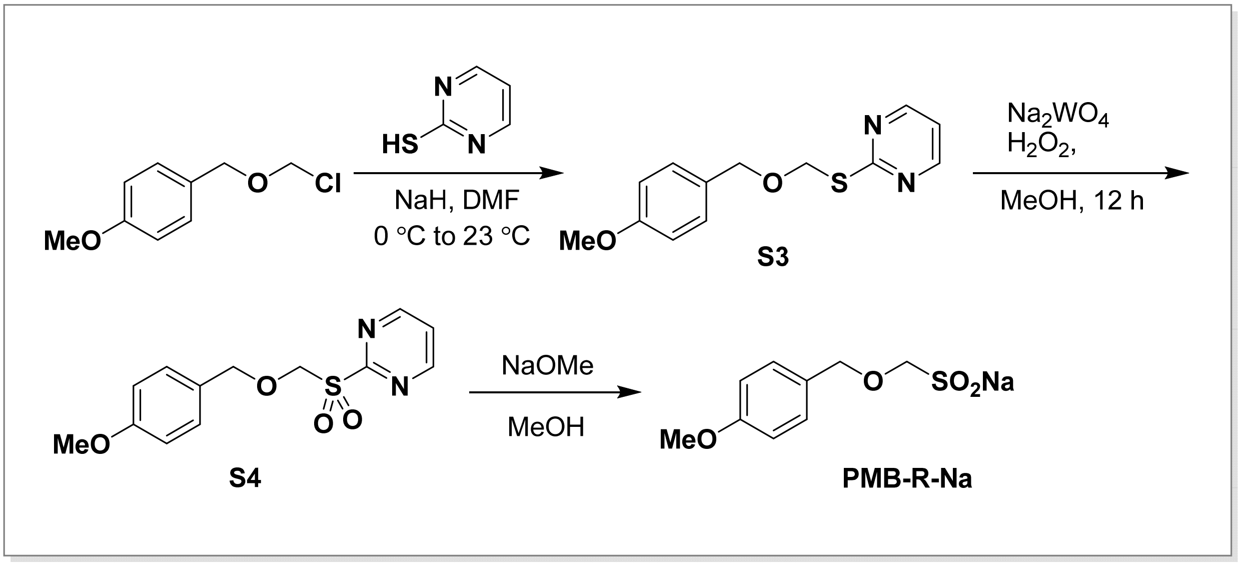

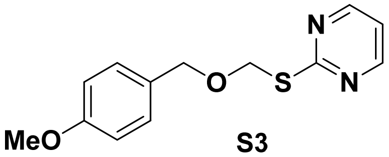

S3

2-((((4-methoxybenzyl)oxy)methyl)thio)pyrimidine (S3)

A 250 mL round-bottom flask was charged with 2-mercaptopyrimidine (3.85 g, 34.33 mmol) and dry DMF (170 mL). NaH (1.65 g, 41.19 mmol, 60% dispersion in oil, 1.2 equiv) was added to the reaction mixture portion wise at 0 °C under N_2 atmosphere. After one hour, 1-((chloromethoxy)methyl)-4-methoxybenzene (PMB-Cl) (6.41 g, 34.33 mmol) was added dropwise to the mixture over 10 minutes. The reaction mixture was then warmed to room temperature and stirred overnight. After completion of the reaction, ammonium chloride was added to quench the reaction at 0 °C. The organic layer was extracted with ethyl acetate (X3) and the combined organic layers were dried over Na_2SO_4. The solvent was removed under reduced pressure, and the resulting residue was purified by flash chromatography to obtain **S3** as a yellow solid (8.55 g, 95% yield). **^{1}H NMR** (400 MHz, $CDCl_3$) δ 8.57 (d, *J* = 4.8 Hz, 2H), 7.30 – 7.26 (m, 2H), 7.02 (t, *J* = 4.8 Hz, 1H), 6.87 (d, *J* = 8.7 Hz, 2H), 5.42 (s, 2H), 4.63 (s, 2H), 3.80 (s, 3H); **^{13}C NMR** (101 MHz, $CDCl_3$) δ 170.9, 159.4, 157.5, 129.9, 129.2, 117.2, 113.8, 70.9, 70.4, 55.3; **HRMS** (ESI-TOF) m/z: $[M+H]^+$ calc'd for $C_{13}H_{15}N_2O_2S$ 263.0849; found: 263.0854.

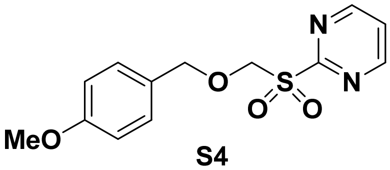

S4

2-((((4-methoxybenzyl)oxy)methyl)sulfonyl)pyrimidine (S4)

To a solution of **S3** (4.40 g, 16.77 mmol) and Na_2WO_4 (2.80 g, 8.39 mmol, 0.5 equiv) in MeOH (160 mL), was added *aq.* H_2O_2 (14.37 mL, 0.25 mol, 50% v/v, 15 equiv) dropwise at 0 °C under N_2 atmosphere. After completion of the reaction, a solution of $Na_2S_2O_5$ (8.8 grams) in 90 mL water was added to the reaction mixture at 0 °C. The organic layer was extracted with dichloromethane (X3), and the combined organic layers were washed with brine and then dried over Na_2SO_4. The solvent was removed under reduced pressure, and the resulting residue was then washed with diethyl ether (X3) to obtain **S4** as a white solid (4.54 g, 92% yield). **^{1}H NMR** (400 MHz, $CDCl_3$) δ 8.97 (d, *J* = 4.8 Hz, 2H), 7.57 (t, *J* = 4.9 Hz, 1H), 7.25 (d, *J* = 8.6 Hz, 2H), 6.86 (d, *J* = 8.6 Hz, 2H), 5.04 (s, 2H), 4.88 (s, 2H), 3.80 (s, 3H); **^{13}C NMR** (101 MHz, $CDCl_3$) δ 165.1, 159.9, 158.8, 130.5, 127.7, 124.0, 114.1, 80.8, 74.3, 55.3; **HRMS** (ESI-TOF) m/z: $[M+Na]^+$ calc'd for $C_{13}H_{14}N_2NaO_2S$: 317.0572; found: 317.0584.

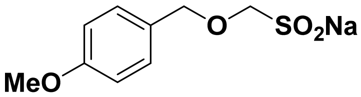

Sodium ((4-methoxybenzyl)oxy)methanesulfinate (4Na, PMB-R-Na)

To a solution of **S4** (4.00 g, 13.59 mmol) in MeOH (40 mL), NaOMe (3.02 mL, 13.59 mmol, 1 equiv) was added at 0 °C. The reaction mixture was then warmed to room temperature and stirred for one hour. After completion of the reaction, the solvent was removed under reduced pressure,

and the resulting residue was then washed with diethyl ether three times, to obtain the desired product as a white solid (3.14 g, 97% yield). **^{1}H NMR** (400 MHz, DMSO) δ 7.23 (d, *J* = 8.6 Hz, 2H), 6.88 (d, *J* = 8.7 Hz, 2H), 4.58 (s, 2H), 3.74 (s, 3H), 3.30 (s, 2H). **^{13}C NMR** (101 MHz, DMSO) δ 158.6, 130.5, 129.3, 113.5, 93.5, 72.1, 55.0; **HRMS** (ESI-TOF) m/z: $[M+H]^+$ calc'd for $C_9H_{12}NaO_4S$: 239.0349; found: 239.0359.

Synthesis of **Sodium ((2-nitrobenzyl)oxy)methanesulfinate (5Na, oNB-R-Na)**

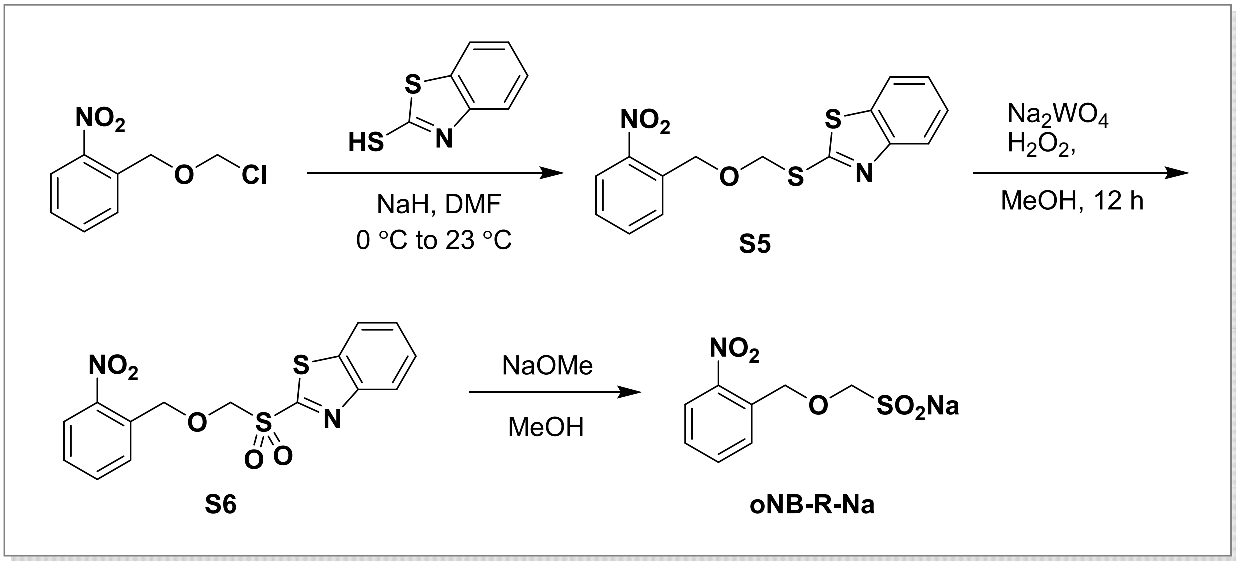

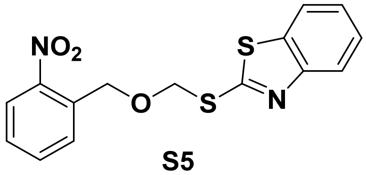

S5

2-((((2-nitrobenzyl)oxy)methyl)thio)benzo[*d*]thiazole (S5)

A 100 mL round-bottom flask was charged with 2-mercaptobenzothiazole (1.69 g, 10.09 mmol) and dry DMF (40 mL). NaH (483 mg, 12.12 mmol, 60% dispersion in oil, 1.2 equiv) was added to the reaction mixture portion wise at 0 °C under N_2 atmosphere. After one hour, 1-((chloromethoxy)methyl)-2-nitrobenzene (oNB-Cl) (2.03 g, 10.09 mmol) was added dropwise to the mixture over 10 minutes. The reaction mixture was then warmed to room temperature and stirred overnight. After completion of the reaction, ammonium chloride was added to quench the reaction at 0 °C. The organic layer was extracted with ethyl acetate (X3) and the combined organic layers were dried over Na_2SO_4. The solvent was removed under reduced pressure, and the resulting residue was purified by flash chromatography to obtain the desired product as a light yellow solid (2.68 g, 80% yield). **^{1}H NMR** (400 MHz, $CDCl_3$) δ 8.08 (dd, *J* = 8.2, 1.4 Hz, 1H), 7.96 – 7.89 (m, 1H), 7.78 (d, *J* = 1.3 Hz, 2H), 7.62 (td, *J* = 7.6, 1.3 Hz, 1H), 7.44 (ddd, *J* = 8.3, 7.3,

1.3 Hz, 2H), 7.33 (ddd, J = 8.3, 7.3, 1.2 Hz, 1H), 5.61 (s, 2H), 5.18 (s, 2H); **^{13}C NMR** (101 MHz, $CDCl_3$) δ 164.7, 153.0, 147.3, 135.7, 133.8, 129.0, 128.3, 126.3, 124.8, 124.7, 122.1, 121.1, 74.8, 68.4; **HRMS** (ESI-TOF) m/z: $[M+H]^+$ calc'd for $C_{15}H_{13}N_2O_3S_2$: 333.0362; found: 333.0367.

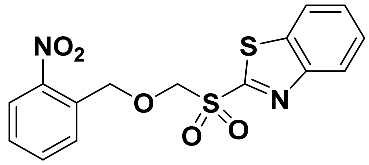

S6

2-((((2-nitrobenzyl)oxy)methyl)sulfonyl)benzo[*d*]thiazole (S6)

To a solution of **S5** (2.60 g, 7.82 mmol) and Na_2WO_4 (1.30 g, 3.91 mmol, 0.5 equiv) in MeOH (80 mL), was added *aq.* H_2O_2 (2.2 mL, 39.1 mmol, 50% v/v, 5 equiv) dropwise at 0 °C under N_2 atmosphere. After completion of the reaction, a solution of $Na_2S_2O_5$ (5.2 grams) in 40 mL water was added to the reaction mixture at 0 °C. The organic layer was extracted with dichloromethane (X3), and the combined organic layers were washed with brine and then dried over Na_2SO_4. The solvent was removed under reduced pressure, and the resulting residue was then washed with diethyl ether (X3) to obtain **S6** as a white solid (2.56 g, 90% yield). **^{1}H NMR** (400 MHz, $CDCl_3$) δ 8.25 (d, J = 7.4 Hz, 1H), 8.04 (ddd, J = 17.1, 7.7, 1.2 Hz, 2H), 7.70 – 7.53 (m, 4H), 7.45 (ddd, J = 8.7, 7.0, 2.0 Hz, 1H), 5.35 (s, 2H), 5.16 (s, 2H); **^{13}C NMR** (101 MHz, $CDCl_3$) δ 164.3, 152.9, 147.2, 137.3, 133.9, 132.4, 129.2, 129.0, 128.4, 127.9, 125.8, 125.1, 122.4, 85.5, 72.3; **HRMS** (ESI-TOF) m/z: $[M+H]^+$ calc'd for $C_{15}H_{13}N_2O_5S_2$: 365.0260; found: 365.0267.

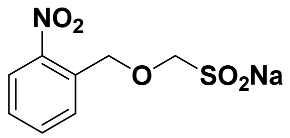

Sodium ((2-nitrobenzyl)oxy)methanesulfinate (5Na, oNB-R-Na)

To a solution of **S6** (1.60 g, 4.39 mmol) in MeOH (22 mL), NaOMe (1 mL, 4.39 mmol, 1 equiv) was added at 0 °C. The reaction mixture was then warmed to room temperature and stirred for one hour. After completion of the reaction, the solvent was removed under reduced pressure, and the resulting residue was then washed with diethyl ether three times, to obtain the desired product as a white solid (1.00 g, 97% yield). **^{1}H NMR** (400 MHz, DMSO) δ 8.05 (dd, J = 8.1, 1.2 Hz, 1H), 7.83 – 7.70 (m, 2H), 7.61 – 7.51 (m, 1H), 5.06 (s, 2H), 3.45 (s, 2H); **^{13}C NMR** (101 MHz, DMSO) δ 147.1, 134.6, 133.8, 128.9, 128.4, 124.4, 93.9, 69.1; **HRMS** (ESI-TOF) m/z: $[M+H]^+$ calc'd for $C_8H_9NNaO_5S$: 254.0094; found: 254.0097.

Synthesis of **Sodium 2-(trimethylsilyl)ethane-1-sulfinate (6Na, SES-Na)**

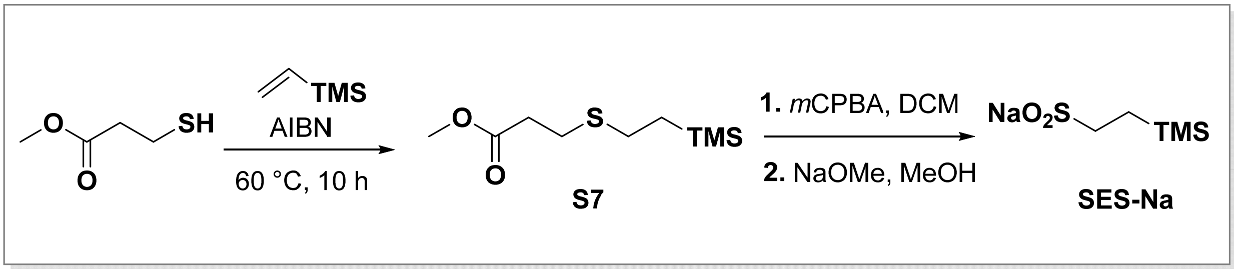

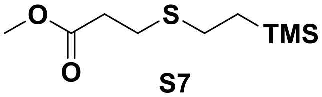
S7

Methyl 3-((2-(trimethylsilyl)ethyl)thio)propanoate (S7)

A sealed 20 mL microwave vial was charged with methyl 3-mercaptopropionate (4.50 mL, 41.61 mmol), trimethyl(vinyl)silane (7.31 mL, 49.9 mmol, 1.2 equiv) and azobisisobutyronitrile (68.3 mg, 1 mol%). The reaction mixture was stirred at 70 °C in an oil bath for 12 h. After cooling to room temperature, the crude product was purified by flash chromatography (eluting with ethyl acetate/hexanes) to afford the desired product as colorless oil (9.08 g, 99% yield). **^{1}H NMR** (400 MHz, $CDCl_3$) δ 3.70 (s, 3H), 2.80 (t, *J* = 7.4 Hz, 2H), 2.67 – 2.49 (m, 4H), 0.91 – 0.78 (m, 2H), 0.02 (s, 9H); **^{13}C NMR** (101 MHz, $CDCl_3$) δ 172.3, 51.6, 34.6, 27.6, 26.7, 17.2, -1.8; **HRMS** (ESI-TOF) m/z: $[M+H]^+$ calc'd for $C_9H_{21}O_2SSi$: 221.1026; found: 221.1033.

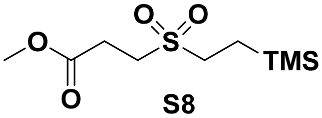
S8

Methyl 3-((2-(trimethylsilyl)ethyl)sulfonyl)propanoate (S8)

To a stirred solution of **S7** (9.08 g, 41.19 mmol) in DCM (400 mL), *m*CPBA (25.39 g, 2.5 equiv) was slowly added at 0 °C. The resulting solution was stirred for 24 h at room temperature. Upon completion, the product was extracted with DCM and washed with sodium bicarbonate and brine. The organic extracts were dried over anhydrous Na_2SO_4, filtered, and concentrated under reduced pressure. The crude product was purified by column chromatography (eluting with ethyl acetate/hexanes) to obtain the corresponding sulfone (**S8**) as a white solid (9.15 g, 88% yield).**^{1}H NMR** (400 MHz, $CDCl_3$) δ 3.74 (s, 3H), 3.30 (t, *J* = 7.5 Hz, 2H), 3.04 – 2.73 (m, 4H), 1.27 – 0.85 (m, 2H), 0.07 (s, 9H); **^{13}C NMR** (101 MHz, $CDCl_3$) δ 171.0, 52.3, 49.8, 46.7, 26.7, 8.4, -2.1; **HRMS** (ESI-TOF) m/z: $[M+H]^+$ calc'd for $C_9H_{21}O_4SSi$: 253.0924; found: 253.0929.

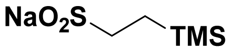

Sodium 2-(trimethylsilyl)ethane-1-sulfinate (6Na, SES-Na)

To a solution of **S8** (8.09 g, 32.09 mmol) in MeOH (200 mL), NaOMe (7.13 mL, 32.09 mmol, 1 equiv) was added at 0 °C. The reaction mixture was then warmed to room temperature and stirred for one hour. After completion of reaction, the solvent was removed under reduced pressure, and the resulting residue was then washed with diethyl ether three times to obtain the desired product as a white solid (5.74 g, 95% yield). **^{1}H NMR** (400 MHz, MeOD) δ 2.21 – 2.14 (m, 2H), 0.82 – 0.74 (m, 2H), 0.02 (s, 9H). **^{13}C NMR** (101 MHz, MeOD) δ 57.6, 9.3, -1.8; **HRMS** (ESI-TOF) m/z: $[M+H]^+$ calc'd for $C_5H_{14}NaO_2SSi$: 189.0381; found: 189.0383.

Sulfone Synthesis:

1. Aryl Iodides to Aryl Sulfones

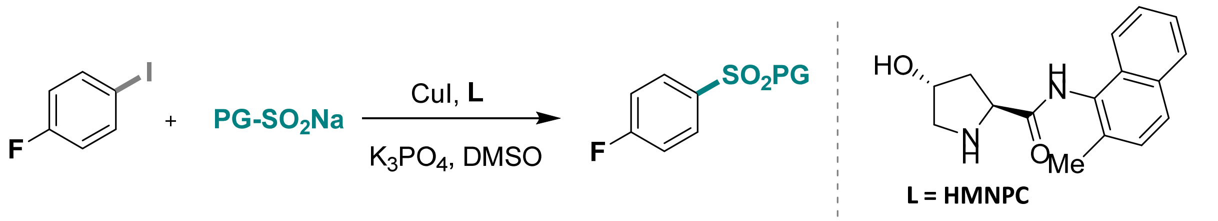

General Procedure A1

A sealed microwave vial was charged with CuI (6 mol%), **L** (6 mol%), PG-SO_2Na (2 equiv), aryl iodide (if solid), and K_3PO_4 (1 equiv). The vial was then evacuated and backfilled with argon, and aryl iodide (if liquid) and DMSO were then added into the tube via syringe. The reaction mixture was stirred at 35 °C in an oil bath for 24 h. After cooling to room temperature, the crude product was diluted with ethyl acetate and filtrated through silica gel. Then the filtrate was concentrated under reduced pressure. The residue was purified by flash chromatography (eluting with ethyl acetate/hexanes) to afford the corresponding aryl sulfone.

General Procedure A2

A sealed microwave vial was charged with CuI (6 mol%), **L** (6 mol%), PG-RSO_2Na (1.5 equiv), aryl iodide (if solid), and K_3PO_4 (1 equiv). The vial was then evacuated and backfilled with argon, and aryl iodide (if liquid) and DMSO were then added into the tube via syringe. The reaction mixture was stirred at 50 °C in an oil bath for 24 h. After cooling to room temperature, the crude product was diluted with ethyl acetate and filtrated through silica gel. Then the filtrate was

concentrated under reduced pressure. The residue was purified by flash chromatography (eluting with ethyl acetate/hexanes) to afford the corresponding aryl sulfone.

General Procedure A3

A sealed microwave vial was charged with CuI (6 mol%), **L** (6 mol%), PG-SO_2Na (1.5 equiv), aryl iodide (if solid), and K_3PO_4 (1 equiv). The vial was then evacuated and backfilled with argon, and aryl iodide (if liquid) and DMSO were then added into the tube via syringe. The reaction mixture was stirred at 35 °C in an oil bath for 24 h. After cooling to room temperature, the crude product was diluted with ethyl acetate and filtrated through silica gel. Then the filtrate was concentrated under reduced pressure. The residue was purified by flash chromatography (eluting with ethyl acetate/hexanes) to afford the corresponding aryl sulfone.

General Procedure A4

A sealed microwave vial was charged with CuI (10 mol%), **L** (10 mol%), PG-SO_2Na (3 equiv), aryl iodide (if solid), and K_3PO_4 (1 equiv). The vial was then evacuated and backfilled with argon, and aryl iodide (if liquid) and DMSO were then added into the tube via syringe. The reaction mixture was stirred at 50 °C in an oil bath for 24 h. After cooling to room temperature, the crude product was diluted with ethyl acetate and filtrated through silica gel. Then the filtrate was concentrated under reduced pressure. The residue was purified by flash chromatography (eluting with ethyl acetate/hexanes) to afford the corresponding aryl sulfone.

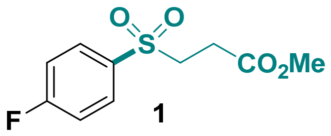

1

Methyl 3-((4-fluorophenyl)sulfonyl)propanoate (1)

According to general procedure A1, 1-fluoro-4-iodobenzene (8.6 µL, 0.07 mmol), SMOPS (26.00 mg, 0.15 mmol), and K_3PO_4 (16 mg, 0.01 mmol) in 0.4 mL DMSO yielded **1** as a white solid (6.65 mg, 36 % yield). **^{1}H NMR** (400 MHz, $CDCl_3$) δ 8.00 – 7.88 (m, 2H), 7.30 – 7.23 (m, 2H), 3.66 (s, 3H), 3.58 – 3.22 (m, 2H), 2.77 (t, J = 7.6 Hz, 2H); **^{13}C NMR** (101 MHz, $CDCl_3$) δ 170.4, 166.1 (d, J = 256.9 Hz), 134.8 (d, J = 3.2 Hz), 131.2 (d, J = 9.6 Hz), 116.9 (d, J = 22.6 Hz), 52.4, 51.8, 27.7; **^{19}F NMR** (376 MHz, $CDCl_3$) δ -102.7; **HRMS** (ESI-TOF) m/z: $[M+H]^+$ calc'd for $C_{10}H_{12}FO_4S$ 247.0435; found: 247.0430.

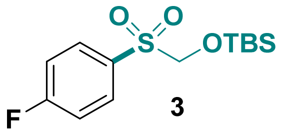

3

tert-butyl(((4-fluorophenyl)sulfonyl)methoxy)dimethylsilane (3)

According to general procedure A1, 1-fluoro-4-iodobenzene (8.6 µL, 0.07 mmol), TBS-R-Na (34.85 mg, 0.15 mmol), K_3PO_4 (16 mg, 0.01 mmol, and (2S,4R)-N-(2,6-dimethylphenyl)- 4-hydroxypyrrolidine-2-carboxamide, DMPHPC (instead of HMNPC) (10 mol %) in 0.4 mL DMSO yielded **3** as a white solid (6.65 mg, 36 % yield). The spectral data matched that of literature.[4]

Notes:

- When HMNPC was used as a ligand, trace amount of product was obtained.
- In accordance with the literature,[4] TBS-R-Na (16.26 mg, 0.07 mmol), DMPHPC (10 mol %), CuI (10 mol%), and 1-fluoro-4-iodobenzene (16.14 µL, 0.15 mmol, 2 equiv) yielded **3** as a white solid (15.34 mg, 72 % yield).

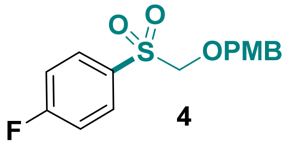

4

1-fluoro-4-((((4-methoxybenzyl)oxy)methyl)sulfonyl)benzene (4)

According to general procedure A2, 1-fluoro-4-iodobenzene (8.6 µL, 0.07 mmol), PMB-R-Na (26.80 mg, 0.10 mmol), and K_3PO_4 (16 mg, 0.01 mmol) in 0.2 mL DMSO yielded **4** as a white solid (21.00 mg, 92 % yield). **^{1}H NMR** (400 MHz, $CDCl_3$) δ 8.01 – 7.89 (m, 2H), 7.25 – 7.18 (m, 4H), 6.88 (d, *J* = 8.6 Hz, 2H), 4.84 (s, 2H), 4.52 (s, 2H), 3.81 (s, 3H); **^{13}C NMR** (101 MHz, $CDCl_3$) δ 166.3 (d, *J* = 256.6 Hz), 160.1, 133.6 (d, *J* = 3.1 Hz), 131.9 (d, *J* = 9.6 Hz), 130.3, 127.8, 116.7 (d, *J* =22.5 Hz), 114.2, 84.5, 74.4, 55.4; **^{19}F NMR** (376 MHz, $CDCl_3$) δ -103.0; **HRMS** (ESI-TOF) m/z: $[M+Na]^+$ calc'd for $C_{15}H_{15}FNaO_4S$: 333.0567; found: 333.0577.

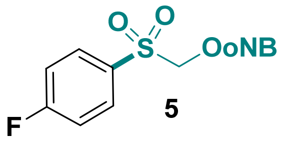

5

1-((((4-fluorophenyl)sulfonyl)methoxy)methyl)-2-nitrobenzene (5)

According to general procedure A4, 1-fluoro-4-iodobenzene (8.6 µL, 0.07 mmol), oNB-R-Na (50.84 mg, 0.22 mmol), and K_3PO_4 (16 mg, 0.01 mmol) in 0.4 mL DMSO yielded **5** as a yellow solid (17.81 mg, 73 % yield). **^{1}H NMR** (400 MHz, $CDCl_3$) δ 8.09 (dd, *J* = 8.1, 1.3 Hz, 1H), 7.99 – 7.92 (m, 2H), 7.63 (ddd, *J* = 14.8, 8.8, 7.3 Hz, 2H), 7.50 (td, *J* = 7.6, 7.0, 1.7 Hz, 1H), 7.31 – 7.21 (m, 2H), 5.28

(s, 2H), 4.69 (s, 2H); **^{13}C NMR** (101 MHz, $CDCl_3$) δ 166.4 (d, *J* = 257.3 Hz), 147.5, 133.9, 133.4 (d, *J* = 3.1 Hz), 132.6, 131.9 (d, *J* = 9.8 Hz), 129.2 (d, *J* = 10.8 Hz), 125.2, 116.9 (d, *J* = 22.7 Hz), 86.2, 72.1; **^{19}F NMR** (376 MHz, $CDCl_3$) δ -102.4; **HRMS** (ESI-TOF) m/z: $[M+H]^+$ calc'd for $C_{14}H_{13}FNO_5S$ 326.0493; found: 326.0499.

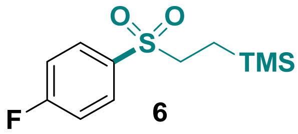

6

(2-((4-fluorophenyl)sulfonyl)ethyl)trimethylsilane (6)

According to general procedure A3, 1-fluoro-4-iodobenzene (8.6 µL, 0.07 mmol), SES-Na (18.83 mg, 0.10 mmol), and K_3PO_4 (16 mg, 0.01 mmol) in 0.2 mL DMSO yielded **6** as a yellow solid (16.95 mg, 93 % yield). **^{1}H NMR** (400 MHz, $CDCl_3$) δ 8.16 – 7.72 (m, 2H), 7.30 – 7.21 (m, 2H), 3.11 – 2.89 (m, 2H), 1.17 – 0.71 (m, 2H), 0.01 (s, 9H); **^{13}C NMR** (101 MHz, $CDCl_3$) δ 165.93 (d, *J* = 255.9 Hz), 134.93 (d, *J* = 3.3 Hz), 131.26 (d, *J* = 9.5 Hz), 116.69 (d, *J* = 22.6 Hz), 53.11, 9.39, -1.90; **^{19}F NMR** (376 MHz, $CDCl_3$) δ -103.8; **HRMS** (ESI-TOF) m/z: $[M+Na]^+$ calc'd for $C_{11}H_{17}FNaO_2SSi$ 283.0600; found: 283.0607.

2. Aryl Bromides to Aryl Sulfones

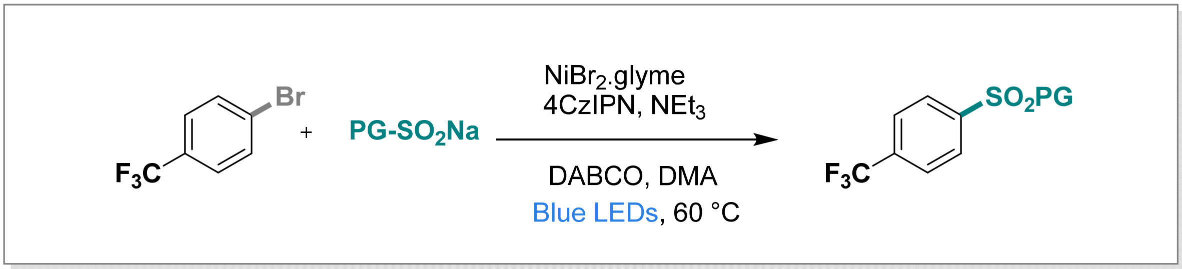

General Procedure B

A sealed vial was charged with PG-SO_2Na (0.2 mmol, 2 equiv), $NiBr_2$.glyme (5 mol%), 4CzIPN (0.2 mol%), DABCO (25 mg, 0.22 mmol, 2.2 equiv) and NEt_3 (7 µL, 0.06 mmol, 0.6 equiv). The vial was then evacuated and backfilled with argon (X3), and 1-bromo-4-(trifluoromethyl) benzene (14 µL, 0.1 mmol) and degassed DMA (0.4 mL) were then added into the vial via syringe. The reaction mixture was stirred at 60 °C under Blue LED irradiation for 4 h. After cooling to room temperature, the crude product was extracted with ethyl acetate and washed with brine. The organic extracts were dried over anhydrous Na_2SO_4, filtered, and concentrated under reduced pressure. The residue was purified by flash chromatography (eluting with ethyl acetate/hexanes) to afford the corresponding aryl sulfone.

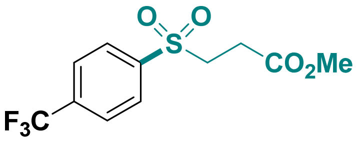

Methyl 3-((4-(trifluoromethyl)phenyl)sulfonyl)propanoate

The general procedure B with SMOPS-Na (35 mg, 0.2 equiv) was adopted to afford the desired product as a white solid (19.55 mg, 66% yield). The spectral data matched that of literature. [5]

Note: The reaction was performed in the presence of air (O_2).

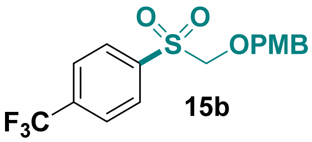

15b

1-methoxy-4-((((4-(trifluoromethyl)phenyl)sulfonyl)methoxy)methyl)benzene (15b)

The general procedure B with PMB-R-Na (48 mg, 0.2 equiv) was adopted to afford **15b** as a light yellow solid (36.03 mg, 82% yield). **^{1}H NMR** (400 MHz, $CDCl_3$) δ 8.07 (d, *J* = 8.2 Hz, 2H), 7.83 (d, *J*

= 8.1 Hz, 2H), 7.22 (d, *J* = 8.7 Hz, 2H), 6.88 (d, *J* = 8.7 Hz, 2H), 4.86 (s, 2H), 4.56 (s, 2H), 3.81 (s, 3H); **^{13}C NMR** (101 MHz, $CDCl_3$) δ 160.1, 141.1, 135.7 (q, *J* = 33.0 Hz), 130.3, 129.6, 128.7, 127.6, 126.4 (q, *J* = 3.7 Hz), 114.2, 84.2, 74.5, 55.4; **^{19}F NMR** (376 MHz, $CDCl_3$) δ -63.2; **HRMS** (ESI-TOF) m/z: $[M+Na]^+$ calc'd for $C_{16}H_{15}F_3NaO_4S$ 383.0541; found: 383.0543.

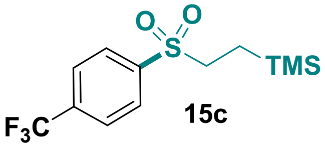

15c

trimethyl(2-((4-(trifluoromethyl)phenyl)sulfonyl)ethyl)silane (15c)

The general procedure B with SES-Na (38 mg, 0.2 equiv) was adopted to afford **15c** as a white solid (38 mg, 62 % yield). **^{1}H NMR** (400 MHz, $CDCl_3$) δ 8.05 (d, *J* = 7.8 Hz, 2H), 7.85 (d, *J* = 8.1 Hz, 2H), 3.06 – 2.94 (m, 2H), 0.95 – 0.89 (m, 2H), 0.01 (s, 9H); **^{13}C NMR** (101 MHz, $CDCl_3$) δ 142.5, 135.4 (q, *J* = 33.1 Hz), 129.1, 126.5 (q, *J* = 3.7 Hz), 123.3 (d, *J* = 273.0 Hz), 52.9, 9.2, -2.0; **^{19}F NMR** (376 MHz, $CDCl_3$) δ -63.2; **HRMS** (ESI-TOF) m/z: $[M+Na]^+$ calc'd for $C_{12}H_{17}F_3NaO_2SSi$ 333.0568; found: 333.0570.

Note: The reaction was performed in the presence of air (O_2).

3. Aryl Thiols to Aryl Sulfones

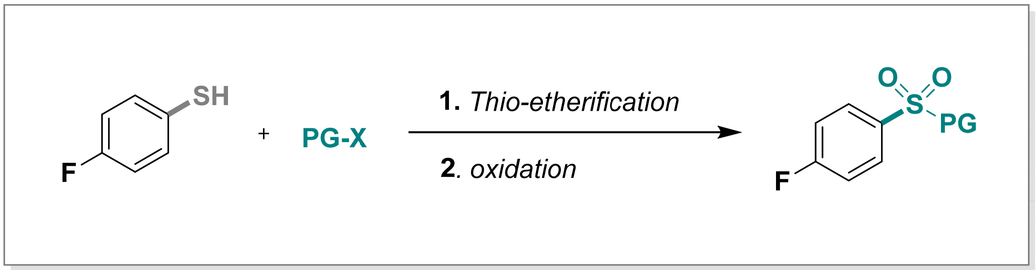

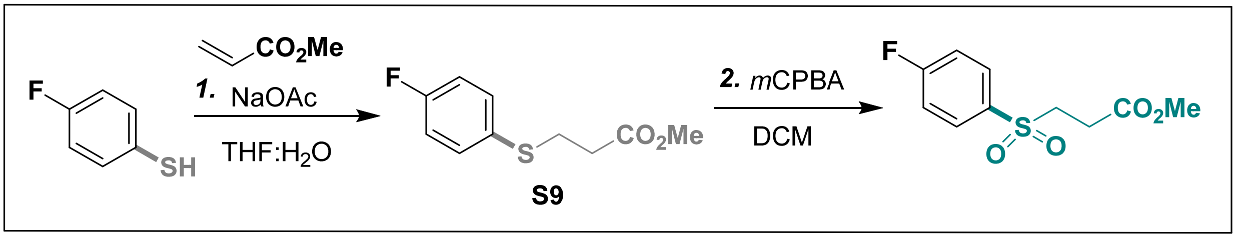

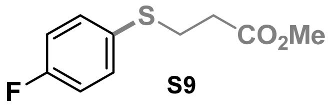

S9

Methyl 3-((4-fluorophenyl)thio)propanoate (S9)

To the solution of 4-fluorobenzenethiol (0.25 mL, 2.34 mmol) in THF:H_2O (4.4 mL, 1:1 v/v) was added methyl acrylate (0.21 mL, 2.34 mmol, 1 equiv) and sodium acetate (29 mg, 0.35 mmol, 0.15 equiv). The reaction mixture was stirred at room temperature for 18 h. Upon completion, the product was extracted with ethyl acetate and washed with brine. The organic extracts were dried over anhydrous Na_2SO_4, filtered, and concentrated under reduced pressure. The crude product was purified by column chromatography (eluting with ethyl acetate/hexanes) to obtain the desired product as yellow oil (386.04 mg, 77% yield). The spectral data matched that of literature.[6]

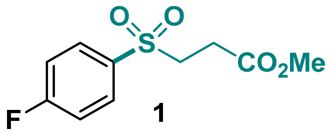

1

Methyl 3-((4-fluorophenyl)sulfonyl)propanoate (1)

To a stirred solution of **S9** (676 mg, 3.15 mmol) in DCM (60 mL), *m*CPBA (1.6 g, 3 equiv) was slowly added at 0 °C. The resulting solution was stirred for 24 h at room temperature. Upon completion, the product was extracted with DCM and washed with sodium bicarbonate and brine. The organic extracts were dried over anhydrous Na_aSO_4, filtered, and concentrated under reduced pressure. The crude product was purified by column chromatography (eluting with ethyl acetate/hexanes) to obtain **1** as a white solid (698.12 mg, 90% yield). **^{1}H NMR** (400 MHz, $CDCl_3$) δ 8.00 – 7.88 (m, 2H), 7.30 – 7.23 (m, 2H), 3.66 (s, 3H), 3.58 – 3.22 (m, 2H), 2.77 (t, *J* = 7.6 Hz, 2H); **^{13}C NMR** (101 MHz, $CDCl_3$) δ 170.4, 166.1 (d, *J* = 256.9 Hz), 134.8 (d, *J* = 3.2 Hz), 131.2 (d, *J* = 9.6 Hz), 116.9 (d, *J* = 22.6 Hz), 52.4, 51.8, 27.7; **^{19}F NMR** (376 MHz, $CDCl_3$) δ -102.7; **HRMS** (ESI-TOF) m/z: $[M+H]^+$ calc'd for $C_{10}H_{12}FO_4S$ 247.0435; found: 247.0430.

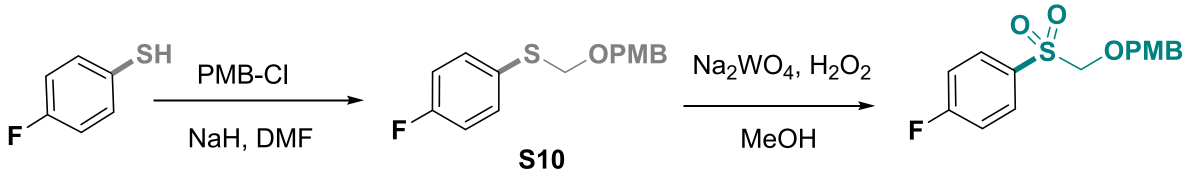

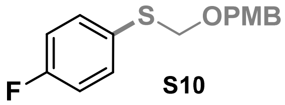

S10

(4-fluorophenyl)(((4-methoxybenzyl)oxy)methyl)sulfane (S10)

A 25 mL round-bottom flask was charged with 4-fluorobenzenethiol (0.27 mL, 2.52 mmol) and dry DMF (10 mL). NaH (120 mg, 3 mmol, 60% dispersion in oil, 1.2 equiv) was added to the reaction mixture portion wise at 0 °C under N_2 atmosphere. After one hour, 1-((chloromethoxy)methyl)-4-methoxybenzene (470.32 mg, 2.52 mmol) was added dropwise to the mixture over 10 minutes. The reaction mixture was then warmed to room temperature and stirred overnight. After completion of the reaction, ammonium chloride was added to quench the reaction at 0 °C. The organic layer was extracted with ethyl acetate (X3) and the combined organic layers were dried over Na_2SO_4. The solvent was removed under reduced pressure, and the resulting residue was purified by flash chromatography to obtain the desired product as a yellow solid (610.23 mg, 87% yield). **^{1}H NMR** (400 MHz, $CDCl_3$) δ 7.58 – 7.44 (m, 2H), 7.31 – 7.23 (m, 2H), 7.02 (t, *J* = 8.7 Hz, 2H), 6.91 (d, *J* = 8.7 Hz, 2H), 4.97 (s, 2H), 4.66 (s, 2H), 3.83 (s, 3H); **^{13}C NMR** (101 MHz, $CDCl_3$) δ 163.5, 161.0, 159.5, 133.0 (d, *J* = 8.1 Hz), 130.9 (d, *J* = 3.4 Hz), 129.9, 129.1, 116.1 (d, *J* = 21.8 Hz), 114.0, 75.6, 69.5, 55.3; **^{19}F NMR** (376 MHz, $CDCl_3$) δ -115.0**; HRMS** (ESI-TOF) m/z: $[M+CH_3CN+H]^+$ calc'd for $C_{17}H_{19}FNO_2S$ 320.1121; found: 320.1121.

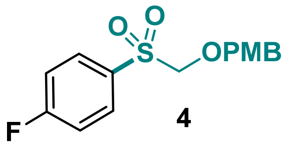

4

1-fluoro-4-((((4-methoxybenzyl)oxy)methyl)sulfonyl)benzene (4)

To a solution of **S10** (270 mg, 0.97 mmol) and Na_2WO_4 (161.93 mg, 0.48 mmol, 0.5 equiv) in MeOH (10 mL), was added *aq.* H_2O_2 (0.28 mL, 4.85 mol, 50% v/v, 5 equiv) dropwise at 0 °C under N_2 atmosphere. After completion of the reaction, a solution of $Na_2S_2O_5$ (500 mg) in 5 mL water was added to the reaction mixture at 0 °C. The organic layer was extracted with dichloromethane (X3), and the combined organic layers were washed with brine and then dried over Na_2SO_4. The solvent was removed under reduced pressure, and the resulting residue was purified by flash chromatography to obtain **4** as a white solid (280 mg, 93% yield). **^{1}H NMR** (400 MHz, $CDCl_3$) δ 8.01 – 7.89 (m, 2H), 7.25 – 7.18 (m, 4H), 6.88 (d, *J* = 8.6 Hz, 2H), 4.84 (s, 2H), 4.52 (s, 2H), 3.81 (s, 3H); **^{13}C NMR** (101 MHz, $CDCl_3$) δ 166.3 (d, *J* = 256.6 Hz), 160.1, 133.6 (d, *J* = 3.1 Hz), 131.9 (d, *J* = 9.6 Hz), 130.3, 127.8, 116.7 (d, *J* = 22.5 Hz), 114.2, 84.5, 74.4, 55.4; **^{19}F NMR** (376 MHz, $CDCl_3$) δ -103.0; **HRMS** (ESI-TOF) m/z: $[M+Na]^+$ calc'd for $C_{15}H_{15}FNaO_4S$ 333.0567; found: 333.0577.

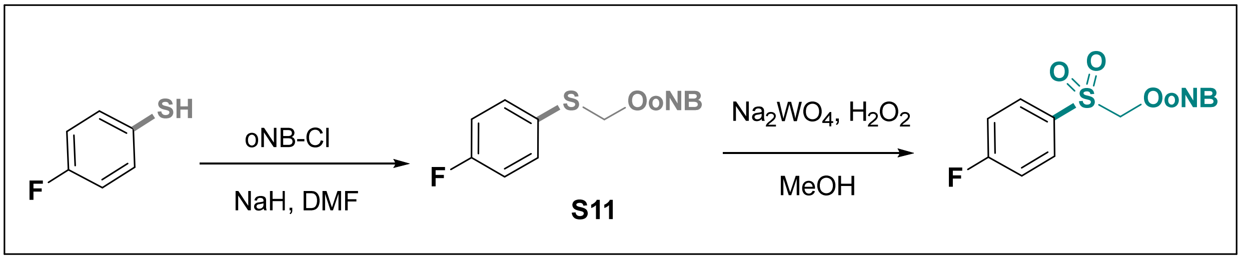

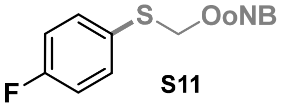

S11

(4-fluorophenyl)(((2-nitrobenzyl)oxy)methyl)sulfane (S11)

A 25 mL round-bottom flask was charged with 4-fluorobenzenethiol (0.27 mL, 2.52 mmol) and dry DMF (10 mL). NaH (120 mg, 3 mmol, 60% dispersion in oil, 1.2 equiv) was added to the reaction mixture portion wise at 0 °C under N_2 atmosphere. After one hour, 1-((chloromethoxy)methyl)-2-nitrobenzene (508.05 mg, 2.52 mmol) was added dropwise to the mixture over 10 minutes. The reaction mixture was then warmed to room temperature and stirred overnight. After completion of the reaction, ammonium chloride was added to quench the reaction at 0 °C. The organic layer was extracted with ethyl acetate (X3) and the combined organic layers were dried over Na_2SO_4. The solvent was removed under reduced pressure, and the resulting residue was purified by flash chromatography to obtain the desired product as a yellow solid (606.10 mg, 82% yield). **^{1}H NMR** (400 MHz, $CDCl_3$) δ 8.08 (dd, *J* = 8.2, 1.4 Hz, 1H), 7.70 (dd, *J* = 7.8, 1.4 Hz, 1H), 7.64 – 7.59 (m, 1H), 7.50 – 7.38 (m, 3H), 7.01 (t, *J* = 8.7 Hz, 2H), 5.07 (s, 4H); **^{13}C NMR** (101 MHz, $CDCl_3$) δ 163.90, 161.45, 147.69, 134.29, 133.94, 133.61 (d, *J* = 8.2 Hz), 130.57 (d, *J* = 3.4 Hz), 129.16, 128.51, 125.08, 116.40 (d, *J* = 21.9 Hz), 77.35, 67.35; **^{19}F NMR** (376 MHz, $CDCl_3$) δ -114.5; **HRMS** (ESI-TOF) m/z: $[M+CH_3CN+H]^+$ calc'd for $C_{16}H_{16}FN_2O_3S$ 335.0866; found: 335.0868.

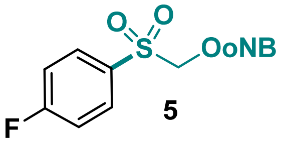

5

1-((((4-fluorophenyl)sulfonyl)methoxy)methyl)-2-nitrobenzene (5)

To a solution of **S11** (260 mg, 0.88 mmol) and Na_2WO_4 (147.98 mg, 0.44 mmol, 0.5 equiv) in MeOH (8 mL), was added *aq.* H_2O_2 (0.25 mL, 4.4 mol, 50% v/v, 5 equiv) dropwise at 0 °C under N_2 atmosphere. After completion of reaction, a solution of $Na_2S_2O_5$ (500 mg) in 5 mL water was added to the reaction mixture at 0 °C. The organic layer was extracted with dichloromethane (X3), and the combined organic layers were washed with brine and then dried over Na_2SO_4. The solvent was removed under reduced pressure, and the resulting residue was purified by flash chromatography to obtain **5** as a yellow solid (263.37 mg, 92% yield). **^{1}H NMR** (400 MHz, $CDCl_3$)

δ 8.09 (dd, *J* = 8.1, 1.3 Hz, 1H), 7.99 – 7.92 (m, 2H), 7.63 (ddd, *J* = 14.8, 8.8, 7.3 Hz, 2H), 7.50 (td, *J* = 7.6, 7.0, 1.7 Hz, 1H), 7.31 – 7.21 (m, 2H), 5.28 (s, 2H), 4.69 (s, 2H); **^{13}C NMR** (101 MHz, $CDCl_3$) δ 166.4 (d, *J* = 257.3 Hz), 147.5, 133.9, 133.4 (d, *J* = 3.1 Hz), 132.6, 131.9 (d, *J* = 9.8 Hz), 129.2 (d, *J* = 10.8 Hz), 125.2, 116.9 (d, *J* = 22.7 Hz), 86.2, 72.1; **^{19}F NMR** (376 MHz, $CDCl_3$) δ -102.4; **HRMS** (ESI-TOF) m/z: $[M+H]^+$ calc'd for $C_{14}H_{13}FNO_5S$ 326.0493; found: 326.0499.

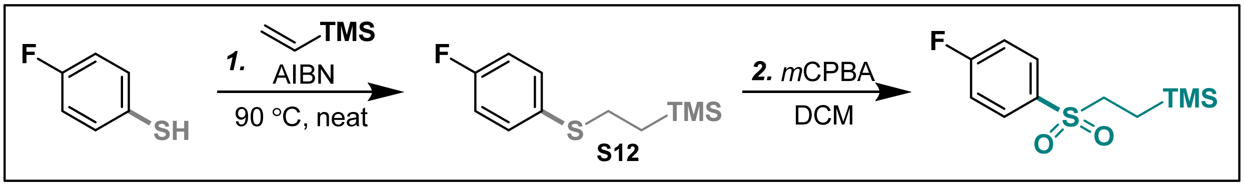

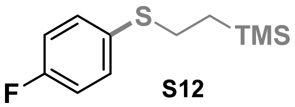

S12

(2-((4-fluorophenyl)thio)ethyl)trimethylsilane (S12)

A sealed microwave vial was charged with 4-fluorobenzenethiol (0.64 mL, 6 mmol, 1.5 equiv), trimethyl(vinyl)silane (0.6 mL, 4 mmol) and azobisisobutyronitrile (1 mol%). The reaction mixture was stirred at 90 °C in an oil bath for 1 h. After cooling to room temperature, the crude product was diluted with diethyl ether and washed with aqueous KOH (1 N) and brine to remove excess of thiol. The crude product was purified by flash chromatography (eluting with ethyl acetate/hexanes) to afford the desired product as colorless oil (877 mg, 96% yield). **^{1}H NMR** (400 MHz, $CDCl_3$) δ 7.32 (dd, *J* = 8.9, 5.2 Hz, 2H), 6.99 (t, *J* = 8.7 Hz, 2H), 3.10 – 2.76 (m, 2H), 1.02 – 0.68 (m, 2H), 0.02 (s, 9H).; **^{13}C NMR** (101 MHz, $CDCl_3$) δ 162.8, 160.4, 132.0 (d, *J* = 7.8 Hz), 115.9 (d, *J* = 21.7 Hz), 30.9, 17.0, -1.8; **^{19}F NMR** (376 MHz, $CDCl_3$) δ -116.1; **HRMS** (ESI-TOF) m/z: $[M+CH_3CN+H]^+$ calc'd for $C_{13}H_{21}FNSSi$ 270.1148; found: 270.1149.

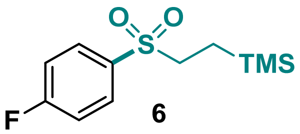

6

(2-((4-fluorophenyl)sulfonyl)ethyl)trimethylsilane (6)

To a stirred solution of **S12** (200 mg, 0.87 mmol) in DCM (60 mL), *m*CPBA (537.43 mg, 2.5 equiv) was slowly added at 0 °C. The resulting solution was stirred for 24 h at room temperature. Upon completion, the product was extracted with DCM and washed with sodium bicarbonate and brine. The organic extracts were dried over anhydrous Na_2SO_4, filtered, and concentrated under reduced pressure. The crude product was purified by column chromatography (eluting with ethyl acetate/hexanes) to obtain **6** as a white solid (194 mg, 86% yield). **^{1}H NMR** (400 MHz, $CDCl_3$) δ

8.16 – 7.72 (m, 2H), 7.30 – 7.21 (m, 2H), 3.11 – 2.89 (m, 2H), 1.17 – 0.71 (m, 2H), 0.01 (s, 9H); **^{13}C NMR** (101 MHz, $CDCl_3$) δ 165.93 (d, *J* = 255.9 Hz), 134.93 (d, *J* = 3.3 Hz), 131.26 (d, *J* = 9.5 Hz), 116.69 (d, *J* = 22.6 Hz), 53.11, 9.39, -1.90; **^{19}F NMR** (376 MHz, $CDCl_3$) δ -103.8; **HRMS** (ESI-TOF) m/z: $[M+Na]^+$ calc'd for $C_{11}H_{17}FNaO_2SSi$ 283.0600; found: 283.0607.

4. Alkyl Halides to Alkyl Sulfones

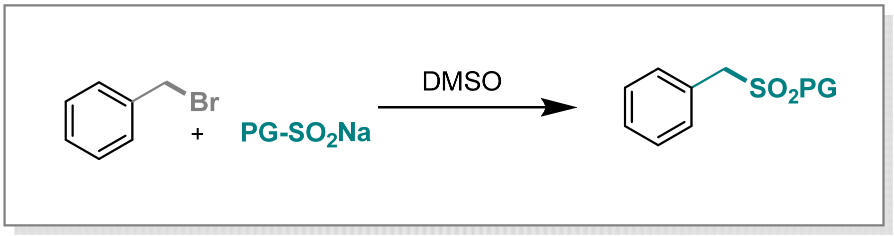

General Procedure C

A mixture of benzyl bromide and PG-SO_2Na (1.5 equiv, unless otherwise noted) in anhydrous DMSO was stirred under N_2 atmosphere at room temperature for 12 h. Upon completion, the reaction mixture was diluted in diethyl ether and washed twice with H_2O. The organic layer was dried with Na_2SO_4, filtered, and concentrated under reduced pressure. The residue was purified by column chromatography (eluting with ethyl acetate/hexanes) to afford the desired alkyl sulfone product.

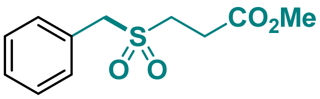

1Bn

Methyl 3-(benzylsulfonyl)propanoate (1Bn)

According to the general procedure C, benzyl bromide (34.7 µL, 0.29 mmol), SMOPS (76 mg, 0.43 mmol, 1.5 equiv) in 1.2 mL DMSO yielded **1Bn** as a white crystalline solid (63 mg, 90% yield). **^{1}H NMR** (400 MHz, $CDCl_3$) δ 7.51 – 7.36 (m, 5H), 4.28 (s, 2H), 3.72 (s, 3H), 3.19 (t, *J* = 7.6 Hz, 2H), 2.80 (t, *J* = 7.5 Hz, 2H); **^{13}C NMR** (101 MHz, $CDCl_3$) δ 171.0, 130.8, 129.3, 127.8, 60.3, 52.5, 46.7, 26.7; **HRMS** (ESI-TOF) m/z: $[M+H]^+$ calc'd for $C_{11}H_{15}O_4S$ 243.0686; found: 243.0696.

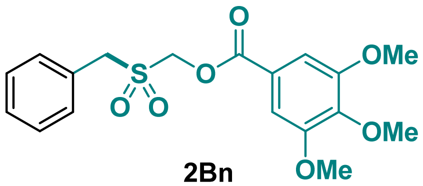

2Bn

(Benzylsulfonyl)methyl 3,4,5-trimethoxybenzoate (2Bn)

A 10 mL round-bottom flask was charged with benzyl bromide (0.14 mL, 1.16 mmol), rongacyl (551 mg, 1.75 mmol, 1.5 equiv), tetrabutyl ammonium bromide (170 mg, 0.52 mmol, 0.3 equiv), and DMA (5 mL). The solution was stirred at 80 °C for 24 h. The reaction was cooled to room temperature and slowly poured into a stirred 0.5 M $NaHCO_3$ (50 mL) and the suspension was stirred for 2 h. Solid was filtered off, washed with water (40 mL) and EtOAc-hexane (1:2). After drying on filter under vacuum suction the obtained solid was dried *in vacuo* at 45 °C to obtain **2Bn** as a white solid (410 mg, 93%). The spectral data matched that of literature. [3]

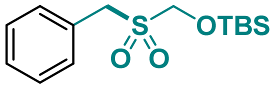

3Bn

((Benzylsulfonyl)methoxy)(tert-butyl)dimethylsilane (3Bn)

According to the general procedure C, benzyl bromide (34.7 µL, 0.29 mmol), TBS-R-Na (101mg, 0.43 mmol, 1.5 equiv) in 1.2 mL DMSO yielded **3Bn** as a white crystalline solid (77.60 mg, 89% yield). The spectral data matched that of literature.[4]

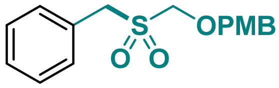

4Bn

1-(((Benzylsulfonyl)methoxy)methyl)-4-methoxybenzene (4Bn)

According to the general procedure C, benzyl bromide (33 µL, 0.28 mmol), PMB-R-Na (100 mg, 0.42 mmol, 1.5 equiv) in 1.2 mL DMSO yielded **4Bn** as a white solid (83.21 mg, 97% yield). **^{1}H NMR** (400 MHz, $CDCl_3$) δ 7.41 – 7.35 (m, 5H), 7.31 (d, *J* = 8.6 Hz, 2H), 6.92 (d, *J* = 8.7 Hz, 2H), 4.88 (s, 2H), 4.32 (s, 2H), 4.28 (s, 2H), 3.83 (s, 3H); **^{13}C NMR** (101 MHz, $CDCl_3$) δ 160.1, 130.9, 130.5, 129.1, 129.1, 127.9, 127.8, 114.2, 79.5, 74.3, 56.5, 55.5; **HRMS** (ESI-TOF) m/z: $[M+Na]^+$ calc'd for $C_{16}H_{18}O_4S$ 329.0818; found: 329.0829.

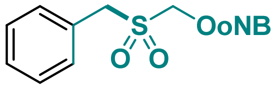

5Bn

1-(((Benzylsulfonyl)methoxy)methyl)-2-nitrobenzene (5Bn)

According to the general procedure C, benzyl bromide (0.14 mL, 1.16 mmol), oNB-R-Na (393 mg, 1.74 mmol, 1.5 equiv) in 5 mL DMSO yielded **5Bn** as a light yellow solid (328 mg, 88% yield). **^{1}H NMR** (400 MHz, $CDCl_3$) δ 8.11 (d, *J* = 8.7 Hz, 1H), 7.75 – 7.63 (m, 2H), 7.57 – 7.49 (m, 1H), 7.43 – 7.38 (m, 5H), 5.32 (s, 2H), 4.43 (s, 2H), 4.35 (s, 2H); **^{13}C NMR** (101 MHz, $CDCl_3$) δ 147.8, 133.9, 132.3, 130.9, 129.6, 129.3, 129.3, 127.6, 125.2, 81.3, 72.1, 56.9; **HRMS** (ESI-TOF) m/z: $[M+H]^+$ calc'd for $C_{15}H_{16}NO_5S$ 322.0744; found: 322.0749.

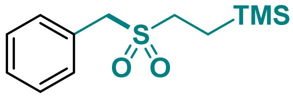

6Bn

(2-(benzylsulfonyl)ethyl)trimethylsilane (6Bn)

According to the general procedure C, benzyl bromide (0.14 mL, 1.16 mmol), SES-Na (327.6 mg, 1.74 mmol, 1.5 equiv) in 5 mL DMSO yielded **6Bn** as a white solid (292 mg, 98% yield). **^{1}H NMR** (400 MHz, $CDCl_3$) δ 7.40 (s, 5H), 4.23 (s, 2H), 2.85 – 2.55 (m, 2H), 1.14 – 0.91 (m, 2H), 0.01 (s, 9H); **^{13}C NMR** (101 MHz, $CDCl_3$) δ 130.6, 128.4, 58.2, 47.6, 8.8, -2.0; **HRMS** (ESI-TOF) m/z: $[M+Na]^+$ calc'd for $C_{12}H_{20}NaO_2SSi$ 279.0851; found: 279.0852.

Application of sulfinates:

1. C-4 Sulfonylation of pyridine

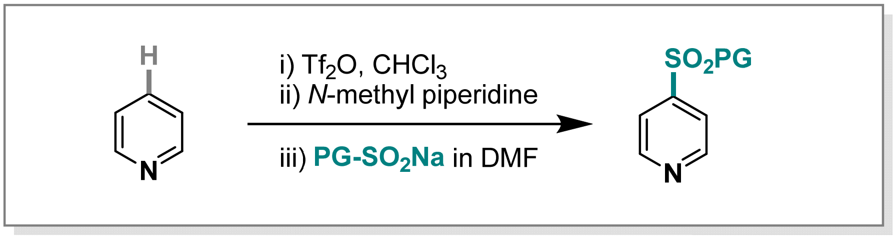

General procedure G

A dried and nitrogen flushed round-bottom flask, equipped with a rubber septum and a magnetic stirring bar was charged with pyridine (24 µL, 0.3 mmol, 1.0 equiv) and dry CH_2Cl_2 (3.0 mL). After cooling to −30 °C, a solution of Tf_2O (55 µL, 0.33 mmol, 1.1 equiv) in dry CH_2Cl_2 (1.2 mL) was added dropwise over 10 min resulting in a colorless suspension. After complete addition the

suspension was stirred for another 30 min at −30 °C. *N*-methylpiperidine (0.12 mL, 0.96 mmol, 3.2 equiv) was added, and after 10 min, a solution of the PG-SO_2Na (0.39 mmol, 1.3 equiv) in DMF (3 ml, 0.1 M) was added. The resulting solution was stirred at −30 °C for another 2 h and slowly warmed to ambient temperature overnight. The reaction mixture was diluted with CH_2Cl_2 and transferred in a separation funnel. Aqueous saturated $NaHCO_3$ was added, the organic layer was separated, and the aqueous phase was extracted with CH_2Cl_2. The combined organic extracts were dried over Na_2SO_4 and evaporated under reduced pressure. Purification of the crude product by flash column chromatography (n-hexane/EtOAc) yielded the desired sulfone.

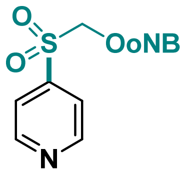

S13a

4-((((2-nitrobenzyl)oxy)methyl)sulfonyl)pyridine (S13a)

Following general procedure G, oNB-R-Na (88 mg, 0.39 mmol) yielded the corresponding sulfone as a yellow solid (45 mg, 49% yield). **^{1}H NMR** (400 MHz, $CDCl_3$) δ 8.93 (d, *J* = 6.0 Hz, 2H), 8.10 (dd, *J* = 8.1, 1.3 Hz, 1H), 7.79 (d, *J* = 6.1 Hz, 2H), 7.65 (td, *J* = 7.5, 1.3 Hz, 1H), 7.61 – 7.40 (m, 2H), 5.32 (s, 2H), 4.75 (s, 2H); **^{13}C NMR** (101 MHz, $CDCl_3$) δ 151.4, 147.5, 145.5, 134.0, 132.2, 129.2 (d, *J* = 4.6 Hz), 125.3, 121.9, 81.6 (d, *J* = 838.2 Hz), 72.3; **HRMS** (ESI-TOF) m/z: $[M+H]^+$ calc'd for $C_{13}H_{13}N_2O_5S$ 309.0540; found: 309.0547.

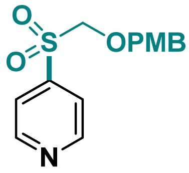

S13b

4-((((4-methoxybenzyl)oxy)methyl)sulfonyl)pyridine (S13b)

Following general procedure G, PMB-R-Na (93 mg, 0.39 mmol) yielded the corresponding sulfone as a yellow oil (51 mg, 58% yield). **^{1}H NMR** (400 MHz, $CDCl_3$) δ 8.90 (d, *J* = 5.0 Hz, 2H), 7.78 (d, *J* = 4.0 Hz, 2H), 7.21 (d, *J* = 6.9 Hz, 2H), 6.88 (d, *J* = 6.7 Hz, 2H), 4.85 (s, 2H), 4.57 (d, *J* = 1.6 Hz, 2H), 3.81 (d, *J* = 1.7 Hz, 3H); **^{13}C NMR** (101 MHz, $CDCl_3$) δ 160.0, 151.1, 145.6, 130.3, 127.3, 114.2, 83.9, 74.4, 55.3; **HRMS** (ESI-TOF) m/z: $[M+H]^+$ calc'd for $C_{14}H_{16}NO_4S$ 294.0795; found: 294.0807.

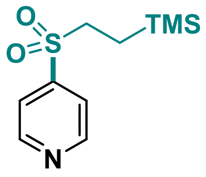

S13c

4-((2-(trimethylsilyl)ethyl)sulfonyl)pyridine (S13c)

Following general procedure G, SES-Na (73 mg, 0.39 mmol) yielded the corresponding sulfone as a yellow oil (26 mg, 36% yield). ^{1}**H NMR** (400 MHz, $CDCl_3$) δ 8.92 (s, 2H), 7.77 (d, *J* = 5.0 Hz, 2H), 3.12 – 2.83 (m, 2H), 0.98 – 0.80 (m, 2H), 0.02 (s, 9H); ^{13}**C NMR** (101 MHz, $CDCl_3$) δ 151.4, 147.0, 121.6, 52.6, 9.1, -1.9; **HRMS** (ESI-TOF) m/z: $[M+H]^+$ calc'd for $C_{10}H_{18}NO_2SSi$ 244.0822; found: 244.0833.

2. Sulfonylation of aniline

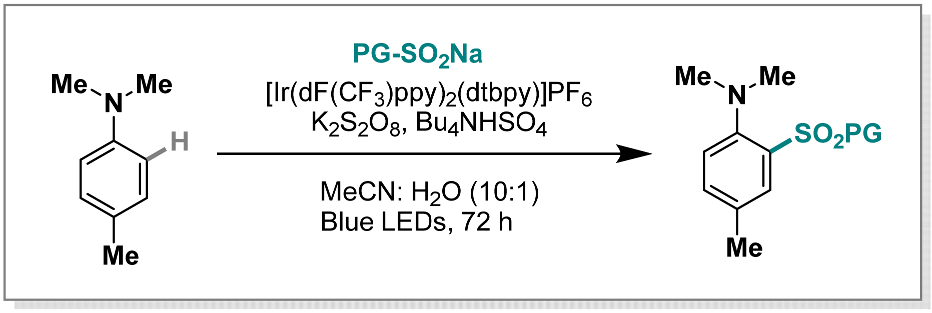

General Procedure H

To a round-bottomed flask charged with $[Ir(dF(CF_3)ppy)_2(dtbpy)]PF_6$ (1 mol %), potassium persulfate (300 mg, 1.11 mmol, 3 equiv), tetrabutylammonium hydrogensulfate (25 mg, 0.07 mmol, 0.20 equiv) and PG-SO_2Na (1.84 mmol, 5 equiv) was added a solution of *N,N*-4-trimethylaniline (51.4 µL, 0.37 mmol) in 10:1 MeCN:H_2O (3.7 mL, 0.1 M) under an inert atmosphere. The mixture was stirred for 72 h under irradiation with blue LEDs. The mixture was neutralized with saturated sodium bicarbonate followed by extraction with EtOAc. The combined organic extracts were dried under Na_2SO_4, filtered and the volatiles were removed under reduced pressure. Purification of the crude product by flash column chromatography (n-hexane/EtOAc) furnished the desired sulfone.

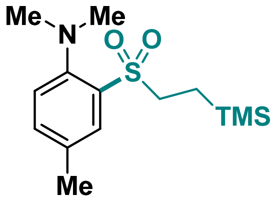

S14

***N,N*,4-trimethyl-2-((2-(trimethylsilyl)ethyl)sulfonyl)aniline (S14)**

Following general procedure H, SES-Na (348 mg, 1.84 mmol) yielded the corresponding sulfone as a yellow oil (70 mg, 63% yield). **^{1}H NMR** (400 MHz, $CDCl_3$) δ 7.84 (s, 1H), 7.39 (dd, *J* = 8.2, 2.2 Hz, 1H), 7.30 (d, *J* = 8.1 Hz, 1H), 3.65 – 3.43 (m, 2H), 2.73 (s, 6H), 2.38 (s, 3H), 0.87 – 0.70 (m, 2H), 0.01 (s, 9H); **^{13}C NMR** (101 MHz, $CDCl_3$ δ 151.6, 135.3, 135.2, 134.2, 130.9, 123.2, 50.3, 46.4, 20.9, 9.6, -1.9; **HRMS** (ESI-TOF) m/z: $[M+H]^+$ calc'd for $C_{14}H_{26}NO_2SSi$ 300.1448; found: 300.1455.

3. Sulfonylation of benzaldehyde

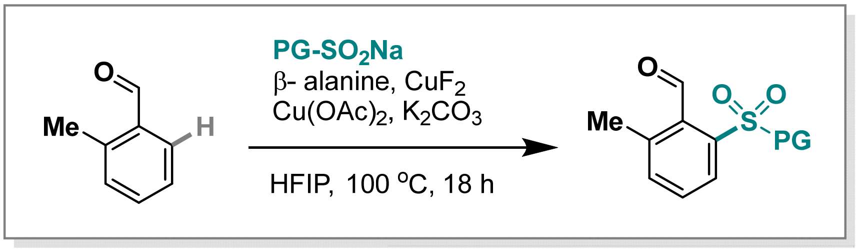

General Procedure I

Potassium carbonate (72 mg, 0.52 mmol, 2 equiv) and copper(II) fluoride (53 mg, 0.52 mmol, 2 equiv) were added sequentially to a microwave vial which was then flame dried under argon until a blue color just appeared (ca. 2–5 seconds). The microwave vial was allowed to cool to room temperature and copper(II) acetate (24 mg, 0.13 mmol, 50 mol%), β-alanine (4.5 mg, 0.05 mmol, 25 mol%), R-SO_2Na (0.26 mmol) and 2-methylbenzaldehyde (76 µL, 0.65 mmol, 2.5 equiv) were added to a microwave vial sequentially under argon. The vial was sealed, and HFIP (1.3 mL, 0.2 M) was added. The vial was submerged in a preheated oil bath to 100 °C for 18 h. The reaction was allowed to cool to room temperature, diluted with EtOAc, and the organic phase was washed with a saturated aqueous solution of ammonium chloride. The product was extracted from the aqueous phase with EtOAc, and the combined organic extracts were dried over Na_2SO_4, filtered, and concentrated *in vacuo*. The crude product was purified by flash column chromatography (n-hexane/EtOAc) to obtain the desired sulfone.

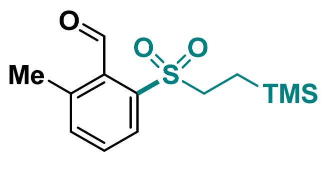

S15

2-methyl-6-((2-(trimethylsilyl)ethyl)sulfonyl)benzaldehyde (S15)

Following general procedure I, SES-Na (50 mg, 0.26 mmol) yielded the corresponding sulfone as a white solid (32 mg, 43% yield). **^{1}H NMR** (400 MHz, $CDCl_3$) δ 10.74 (s, 1H), 7.89 (d, *J* = 7.3 Hz, 1H), 7.55 (q, *J* = 8.5, 8.0 Hz, 2H), 3.31 – 3.02 (m, 2H), 2.50 (s, 3H), 1.08 – 0.83 (m, 2H), 0.03 (s, 9H); **^{13}C NMR** (101 MHz, $CDCl_3$ δ 194.8, 139.1, 138.7, 136.7, 131.1, 128.6, 54.8, 29.8, 20.0, 9.0, -1.9; **HRMS** (ESI-TOF) m/z: $[M+Na]^+$ calc'd for $C_{13}H_{21}NaO_3SSi$ 307.0800; found: 307.0799.

4. Sulfonylation of alcohols

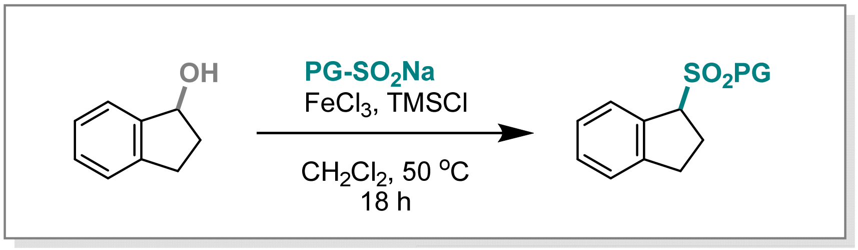

General Procedure J

To a solution of 2,3-dihydro-1*H*-inden-1-ol (50 mg, 0.37 mmol), R-SO_2Na (0.56 mmol, 1.5 equiv) and $FeCl_3$ (9 mg, 15 mol%) in CH_2Cl_2 (1.2 mL), was added TMSCl (93.92 µL, 0.74 mol, 1.2 equiv) under N_2 atmosphere. The resulting solution was stirred at 45 °C and monitored using TLC. After completion of reaction, the reaction mixture was quenched with $NaHCO_3$. The organic layer was extracted with ethyl acetate (X3), and the combined organic layers were washed with brine and then dried over Na_2SO_4. The solvent was removed under reduced pressure, and the resulting residue was purified by flash chromatography to obtain the desired sulfone.

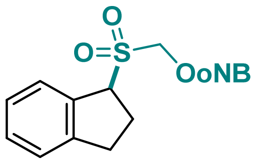

S16a

1-((((2-nitrobenzyl)oxy)methyl)sulfonyl)-2,3-dihydro-1*H*-indene (S16a)

Following general procedure J, oNB-R-Na (141.53 mg, 0.56 mmol, 1.5 equiv) yielded the corresponding sulfone as a yellow solid (63 mg, 49% yield). **^{1}H NMR** (400 MHz, $CDCl_3$) δ 8.10 (d, *J* = 8.1 Hz, 1H), 7.70 – 7.61 (m, 2H), 7.58 (d, *J* = 7.6 Hz, 1H), 7.51 (td, *J* = 7.5, 6.6, 2.1 Hz, 1H), 7.36 – 7.29 (m, 2H), 7.28 – 7.20 (m, 1H), 5.30 (s, 2H), 4.78 (dd, *J* = 9.3, 2.5 Hz, 1H), 4.65 (d, *J* = 12.8 Hz, 1H), 4.52 (d, *J* = 12.8 Hz, 1H), 3.28 (dt, *J* = 16.8, 8.7 Hz, 1H), 2.98 (ddd, *J* = 16.2, 9.3, 2.6 Hz, 1H), 2.78 (ddt, *J* = 14.1, 8.6, 2.6 Hz, 1H), 2.56 (dq, *J* = 14.5, 9.2 Hz, 1H); **^{13}C NMR** (101 MHz, $CDCl_3$) δ 147.7, 146.2, 134.0, 133.9, 132.4, 129.7, 129.5, 129.2, 127.1, 126.7, 125.4, 125.2, 81.7, 72.0, 67.0, 31.4, 26.7; **HRMS** (ESI-TOF) m/z: $[M+Na]^+$ calc'd for $C_{17}H_{17}NNaO_5S$ 370.0725; found: 370.0728.

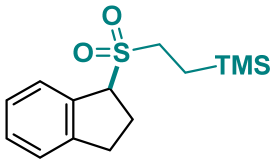

S16b

(2-((2,3-dihydro-1*H*-inden-1-yl)sulfonyl)ethyl)trimethylsilane (S16b)

Following general procedure J, SES-R-Na (105.44 mg, 0.56 mmol, 1.5 equiv) yielded the corresponding sulfone as a light yellow solid (60 mg, 58% yield). **^{1}H NMR** (400 MHz, $CDCl_3$) δ 7.55 (d, *J* = 7.5 Hz, 1H), 7.38 – 7.21 (m, 3H), 4.62 (dd, *J* = 9.1, 3.1 Hz, 1H), 3.24 (dt, *J* = 16.7, 8.6 Hz, 1H), 3.00 (ddd, *J* = 16.3, 9.3, 3.2 Hz, 1H), 2.86 – 2.73 (m, 2H), 2.68 (ddt, *J* = 14.7, 8.6, 3.1 Hz, 1H), 2.56 (dq, *J* = 14.5, 9.0 Hz, 1H), 1.14 – 0.94 (m, 2H), 0.02 (s, 9H); **^{13}C NMR** (101 MHz, $CDCl_3$) δ 145.7, 134.6, 129.5, 127.1, 126.7, 125.4, 67.8, 45.9, 31.3, 27.1, 7.7, -1.9; **HRMS** (ESI-TOF) m/z: $[M+Na]^+$ calc'd for $C_{14}H_{22}NaO_2SSi$ 305.1007; found: 305.1011.

Stability of SPGs:

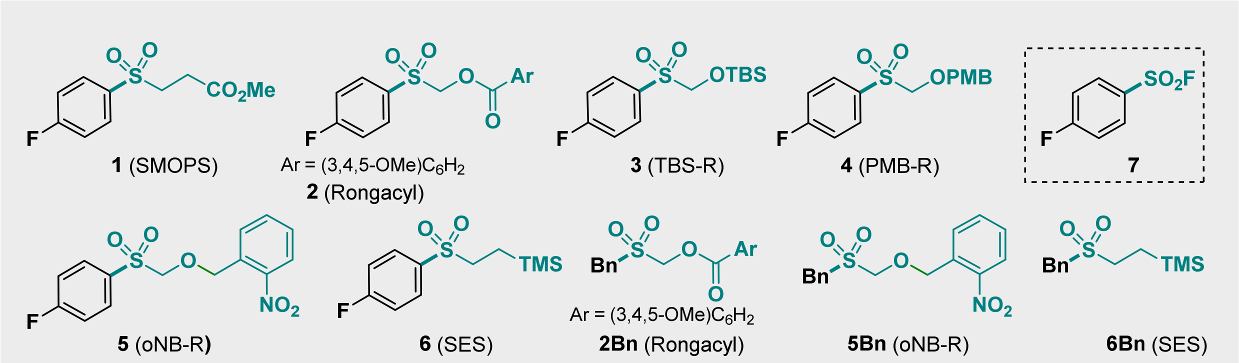

1. Amide Coupling

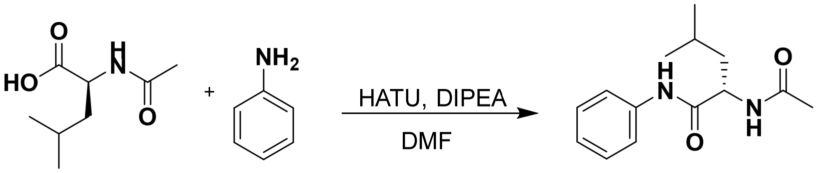

To the solution of acetyl-*L*-leucine (200 mg, 1.15 mmol) in 6 mL DMF, was added DIPEA (0.60 mL, 3.45 mmol, 3 equiv) and HATU (524 mg, 1.38 mmol, 1.2 equiv). The resulting mixture was stirred at room temperature for 1 hour, after which aniline (0.11 mL, 1.27 mmol, 1.1 equiv) was added and stirred overnight. Upon completion, the product was extracted with ethyl acetate and washed with brine. The organic extracts were dried over anhydrous Na_2SO_4, filtered, and concentrated under reduced pressure. The crude product was purified by column chromatography (eluting with ethyl acetate/hexanes) to obtain (S)-2-acetamido-4-methyl-N-phenylpentanamide as a white solid (206 mg, 72% yield).**^{1}H NMR** (400 MHz, DMSO) δ 10.04 (s, 1H), 8.13 (d, *J* = 7.9 Hz, 1H), 7.61 (d, *J* = 7.4 Hz, 2H), 7.36 – 7.24 (m, 2H), 7.11 – 6.92 (m, 1H), 4.45 (td, *J* = 8.9, 5.9 Hz, 1H), 1.86 (s, 3H), 1.72 – 1.57 (m, 1H), 1.58 – 1.43 (m, 2H), 0.90 (dd, *J* = 13.3, 6.5 Hz, 6H); **^{13}C NMR** (101 MHz, DMSO) δ 171.35, 169.23, 138.99, 128.62, 123.23, 119.29, 51.88, 40.93, 24.33, 22.92, 22.39, 21.70; **HRMS** (ESI-TOF) m/z: $[M+H]^+$ calc'd for $C_{14}H_{21}N_2O_2$ 249.1598; found: 249.1612.

An example for the setup of stability testing of **1**:

To the solution of acetyl-*L*-leucine (17.3 mg, 0.1 mmol) and **1** (25 mg, 0.1 mmol, 1 equiv) in 0.5 mL DMF, was added DIPEA (0.05 mL, 0.3 mmol, 3 equiv) and HATU (45.6 mg, 0.12 mmol, 1.2 equiv). The resulting mixture was stirred at room temperature for 1 hour, after which aniline (10 µL, 0.11 mmol, 1.1 equiv) was added and stirred overnight. Upon completion, the product was extracted with ethyl acetate and washed with brine. The organic extracts were dried over anhydrous Na_2SO_4, filtered, and concentrated under reduced pressure. To the crude residue was added 4-fluoro-acetophenone (12 µL, 0.1 mmol, 1 equiv) as an internal standard. The NMR yield was calculated as the percentage of **1** recovered after the completion of reaction.

2. SN_{Ar}

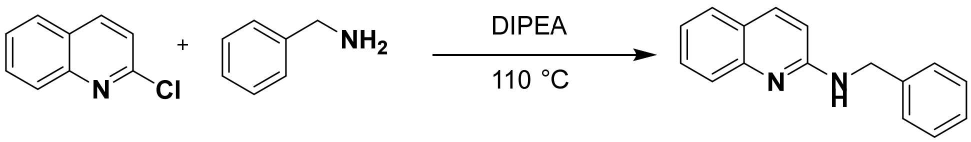

An example for the setup of stability testing of **1**:

In a sealed vial were combined 2-chloroquinoline (16.4 mg, 0.1 mmol), benzylamine (13 µL, 0.12 mmol, 1.2 equiv), **1** (25 mg, 0.1 mmol, 1 equiv), and DIPEA (35 µL, 0.2 mmol, 2 equiv). The solution was stirred at 110 °C for 16 h. The reaction was cooled to room temperature and 4-fluoro-acetophenone (12 µL, 0.1 mmol, 1 equiv) was added as an internal standard. The NMR yield was calculated as the percentage of **1** recovered after the completion of reaction.

3. Boc deprotection

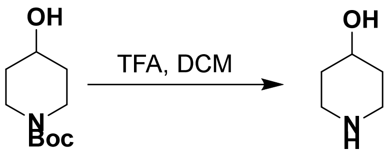

An example for the setup of stability testing of **1**:

To the solution of *tert*-butyl 4-hydroxypiperidine-1-carboxylate (20 mg, 0.1 mmol) and **1** (25 mg, 0.1 mmol, 1 equiv) in 0.5 mL DCM, was added 0.5 mL trifluoroacetic acid. The resulting mixture was stirred at room temperature for 12 h. Upon completion, the solvent was removed under reduced pressure and 4-fluoro-acetophenone (12 µL, 0.1 mmol, 1 equiv) was added as an internal standard. The NMR yield was calculated as the percentage of **1** recovered after the completion of reaction.

4. Ester hydrolysis

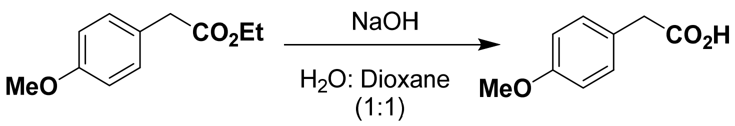

An example for the setup of stability testing of **1**:

To the solution of ethyl 2-(4-methoxyphenyl)acetate (18 µL, 0.1 mmol) and **1** (25 mg, 0.1 mmol, 1 equiv) in 0.2 mL H_2O:Dioxane (1:1), was added sodium hydroxide (12 mg, 0.3 mmol, 3 equiv). The resulting mixture was stirred at room temperature for 12 h. Upon completion, 4-fluoro-acetophenone (12 µL, 0.1 mmol, 1 equiv) was added as an internal standard. The NMR yield was calculated as the percentage of **1** recovered after the completion of reaction.

5. Suzuki-Miyaura Coupling

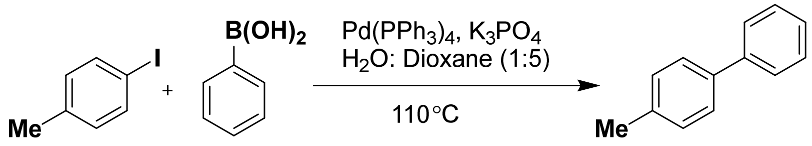

An example for the setup of stability testing of **1**:

A sealed vial was charged with 1-iodo-4-methylbenzene (22 mg, 0.1 mmol), phenyl boronic acid (16 mg, 0.13 mmol, 1.3 equiv), **1** (25 mg, 0.1 mmol, 1 equiv), $Pd(PPh_3)_4$ (12 mg, 10 mol%), and K_3PO_4 (32 mg, 0.15 mmol. 1.5 equiv). The vial was then evacuated and backfilled with argon (X3), and degassed 0.48 mL H_2O: Dioxane (1:5) were then added into the vial via syringe. The resulting mixture was stirred at 110 °C for 12 h. The reaction was cooled to room temperature and 4-fluoro-acetophenone (12 µL, 0.1 mmol, 1 equiv) was added as an internal standard. The NMR yield was calculated as the percentage of **1** recovered after the completion of reaction.

6. Amine SN_2

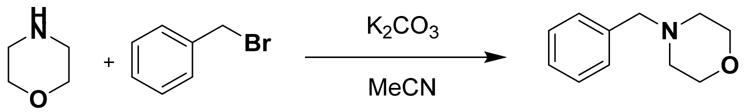

An example for the setup of stability testing of **1**:

To a suspension of morpholine (8.6 µL, 0.1 mmol), **1** (25 mg, 0.1 mmol, 1 equiv), and K_2CO_3 (18 mg, 0.13 mmol. 1.3 equiv) in 0.12 mL CH_3CN, was added benzyl bromide (13 µL, 0.11 mmol, 1.1 equiv). The resulting mixture was stirred at room temperature for 2 h. Upon completion, 4-fluoro-acetophenone (12 µL, 0.1 mmol, 1 equiv) was added as an internal standard. The NMR yield was calculated as the percentage of **1** recovered after the completion of reaction.

7. Reductive Amination

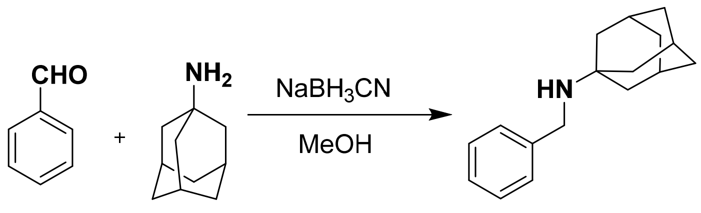

An example for the setup of stability testing of **1**:

To the solution of benzaldehyde (10.2 µL, 0.1 mmol), adamantan-1-amine (18.1 mg, 0.12 mmol, 1.2 equiv), and **1** (25 mg, 0.1 mmol, 1 equiv) in 0.4 mL MeOH, was added sodium cyanoborohydride (18.85 mg, 0.3 mmol, 3 equiv). The resulting mixture was stirred at room temperature for 12 h. Upon completion, 4-fluoro-acetophenone (12 µL, 0.1 mmol, 1 equiv) was added as an internal standard. The NMR yield was calculated as the percentage of **1** recovered after the completion of reaction.

8. Hydrogenation

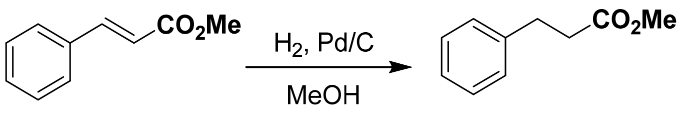

An example for the setup of stability testing of **1**:

To a solution of methyl cinnamate (16.2 mg, 0.1 mmol) and **1** (25 mg, 0.1 mmol, 1 equiv) in 0.3 mL MeOH, was added Pd/C (10 mol%). The resulting mixture was stirred under H_2 atmosphere for 12 h. Upon completion, 4-fluoro-acetophenone (12 µL, 0.1 mmol, 1 equiv) was added as an internal standard. The NMR yield was calculated as the percentage of **1** recovered after the completion of reaction.

9. Buchwald-Hartwig Coupling

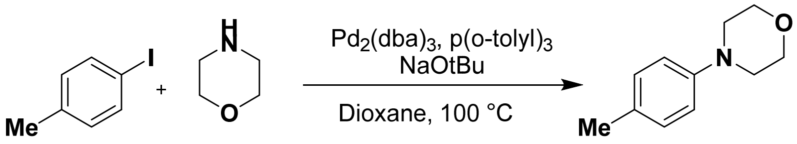

An example for the setup of stability testing of **1**:

A sealed vial was charged with 1-iodo-4-methylbenzene (22 mg, 0.1 mmol), morpholine (20 µL, 0.24 mmol, 2.4 equiv), **1** (25 mg, 0.1 mmol, 1 equiv), NaOtBu (27 mg, 0.28 mmol, 2.8 equiv), $Pd_2(dba)_3$ (1 mol%) and $p(o\text{-tolyl})_3$ (1 mol%). The vial was then evacuated and backfilled with argon (X3), and degassed 0.4 mL dioxane was then added into the vial via syringe. The resulting mixture was stirred at 100 °C for 24 h. The reaction was cooled to room temperature and 4-fluoro-acetophenone (12 µL, 0.1 mmol, 1 equiv) was added as an internal standard. The NMR yield was calculated as the percentage of **1** recovered after the completion of reaction.

Substrate Scope

Synthesis of Sulfones:

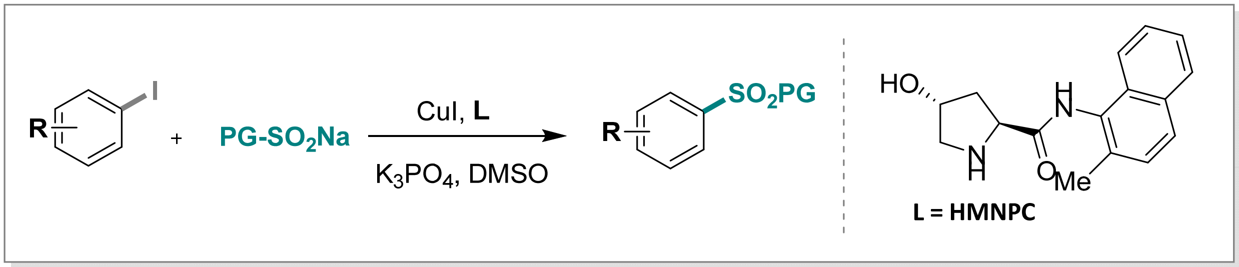

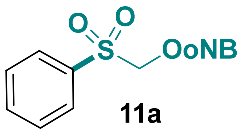

11a

1-nitro-2-(((phenylsulfonyl)methoxy)methyl)benzene (11a)

According to general procedure A4, iodobenzene (8.4 µL, 0.07 mmol), oNB-R-Na (50.80 mg, 0.22 mmol), **L** (2.02 mg), CuI (1.4 mg), and K_3PO_4 (16 mg, 0.07 mmol) in 0.4 mL DMSO yielded **11a** as a yellow solid (9.70 mg, 42 % yield). **^{1}H NMR** (400 MHz, $CDCl_3$) δ 8.08 (d, *J* = 8.7 Hz, 1H), 7.94 (d, *J* = 8.1 Hz, 2H), 7.70 (t, *J* = 7.5 Hz, 1H), 7.66 – 7.55 (m, 4H), 7.48 (ddd, *J* = 8.7, 6.4, 2.5 Hz, 1H), 5.25 (s, 2H), 4.71 (s, 2H); **^{13}C NMR** (101 MHz, $CDCl_3$) δ 147.3, 137.3, 134.4, 133.9, 132.8, 129.4, 129.0, 128.9, 128.9, 125.0, 86.1, 71.9; **HRMS** (ESI-TOF) m/z: $[M+H]^+$ calc'd for $C_{14}H_{14}NO_5S$ 308.0587; found: 308.0588.

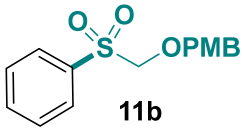

11b

1-methoxy-4-(((phenylsulfonyl)methoxy)methyl)benzene (11b)

According to general procedure A2, iodobenzene (8.4 µL, 0.07 mmol), PMB-R-Na (26.80 mg, 0.11 mmol), **L** (1.2 mg), CuI (0.8 mg), and K_3PO_4 (16 mg, 0.07 mmol) in 0.2 mL DMSO yielded **11b** as a white solid (15.6 mg, 71 % yield). **^{1}H NMR** (400 MHz, $CDCl_3$) δ 7.98 – 7.89 (m, 2H), 7.70 – 7.65 (m, 1H), 7.61 – 7.53 (m, 2H), 7.22 (d, *J* = 8.7 Hz, 2H), 6.87 (d, *J* = 8.7 Hz, 2H), 4.83 (s, 2H), 4.54 (s, 2H), 3.81 (s, 3H); **^{13}C NMR** (101 MHz, $CDCl_3$) δ 159.9, 137.6, 134.1, 130.2, 129.3, 128.9, 127.9, 114.1, 84.4, 74.2, 55.3; **HRMS** (ESI-TOF) m/z: $[M+Na]^+$ calc'd for $C_{15}H_{16}O_4S$ 315.0662; found: 315.0670.

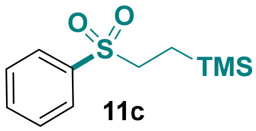

11c

trimethyl(2-(phenylsulfonyl)ethyl)silane (11c)

According to general procedure A3, iodobenzene (25.6 µL, 0.23 mmol), SES-Na (65 mg, 0.34 mmol), **L** (3.7 mg), CuI (2.6 mg), and K_3PO_4 (49 mg, 0.23 mmol) in 0.6 mL DMSO yielded **11c** as a white solid (45 mg, 80 % yield). **^{1}H NMR** (400 MHz, $CDCl_3$) δ 7.97 – 7.87 (m, 2H), 7.72 – 7.63 (m, 1H), 7.62 – 7.52 (m, 2H), 3.08 – 2.87 (m, 2H), 1.04 – 0.83 (m, 2H), 0.01 (s, 9H); **^{13}C NMR** (101 MHz, $CDCl_3$) δ 138.9, 133.7, 129.3, 128.3, 52.8, 9.2, -1.9; **HRMS** (ESI-TOF) m/z: $[M+Na]^+$ calc'd for $C_{11}H_{18}NaO_2SSi$ 265.0694; found: 265.0700.

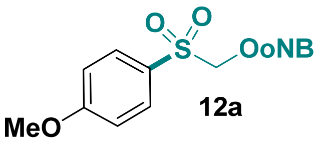

12a

1-((((4-methoxyphenyl)sulfonyl)methoxy)methyl)-2-nitrobenzene (12a)

According to general procedure A4, 1-iodo-4-methoxybenzene (54 mg, 0.23 mmol), oNB-R-Na (156 mg, 0.69 mmol), **L** (6.2 mg), CuI (4.4 mg), and K_3PO_4 (49 mg, 0.23 mmol) in 1.3 mL DMSO yielded **12a** as a light yellow solid (64 mg, 83 % yield). **^{1}H NMR** (400 MHz, $CDCl_3$) δ 8.10 – 8.02 (m, 1H), 7.85 (d, *J* = 8.9 Hz, 2H), 7.68 – 7.54 (m, 2H), 7.53 – 7.40 (m, 1H), 7.02 (d, *J* = 8.9 Hz, 2H), 5.23 (s, 2H), 4.66 (s, 2H), 3.89 (s, 3H); **^{13}C NMR** (101 MHz, $CDCl_3$) δ 164.4, 147.4, 133.9, 133.0, 131.2, 129.2, 128.9, 128.7, 125.1, 114.7, 86.3, 71.9, 55.9; **HRMS** (ESI-TOF) m/z: $[M+Na]^+$ calc'd for $C_{15}H_{15}NNaO_6S$ 360.0518; found: 360.0529.

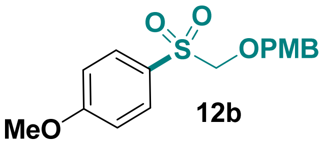

12b

1-methoxy-4-((((4-methoxybenzyl)oxy)methyl)sulfonyl)benzene (12b)

According to general procedure A2, 1-iodo-4-methoxybenzene (54 mg, 0.23 mmol), PMB-R-Na (82 mg, 0.34 mmol), L (3.7 mg), CuI (2.6 mg), and K_3PO_4 (49 mg, 0.23 mmol) in 0.6 mL DMSO yielded **12b** as a white solid (64 mg, 92 % yield). **^{1}H NMR** (400 MHz, $CDCl_3$) δ 7.86 (d, *J* = 9.0 Hz, 2H), 7.22 (d, *J* = 8.7 Hz, 2H), 7.02 (d, *J* = 9.0 Hz, 2H), 6.87 (d, *J* = 8.7 Hz, 2H), 4.82 (s, 2H), 4.50 (s, 2H), 3.88 (s, 3H), 3.81 (s, 3H); **^{13}C NMR** (101 MHz, $CDCl_3$) δ 164.2, 159.9, 131.1, 130.2, 129.0, 128.1, 114.6, 114.1, 84.6, 74.2, 55.8, 55.4; **HRMS** (ESI-TOF) m/z: $[M+Na]^+$ calc'd for $C_{16}H_{18}NaO_5S$ 345.0767; found: 345.0778.

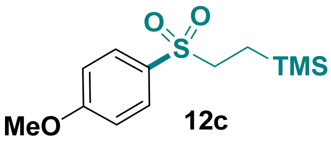

12c

(2-((4-methoxyphenyl)sulfonyl)ethyl)trimethylsilane (12c)

According to general procedure A3, 1-iodo-4-methoxybenzene (54 mg, 0.23 mmol), SES-Na (65 mg, 0.34 mmol), **L** (3.7 mg), CuI (2.6 mg), and K_3PO_4 (49 mg, 0.23 mmol) in 0.6 mL DMSO yielded **12c** as a white solid (60 mg, 96 % yield). **^{1}H NMR** (400 MHz, $CDCl_3$) δ 7.83 (d, *J* = 8.9 Hz, 2H), 7.02 (d, *J* = 8.9 Hz, 2H), 3.89 (s, 3H), 3.08 – 2.84 (m, 2H), 1.05 – 0.78 (m, 2H), -0.00 (s, 9H); **^{13}C NMR**

(101 MHz, $CDCl_3$) δ 163.7, 130.5, 130.4, 114.5, 55.8, 53.1, 9.5, -1.9; **HRMS** (ESI-TOF) m/z: $[M+Na]^+$ calc'd for $C_{12}H_{20}NaO_3SSi$ 295.0795; found: 295.0812.

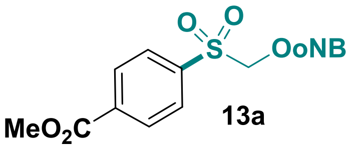

13a

Methyl 4-((((2-nitrobenzyl)oxy)methyl)sulfonyl)benzoate (13a)

According to general procedure A4, methyl 4-iodobenzoate (60.2 mg, 0.23 mmol), oNB-R-Na (156 mg, 0.69 mmol), **L** (6.2 mg), CuI (4.4 mg), and K_3PO_4 (49 mg, 0.23 mmol) in 1.3 mL DMSO yielded **13a** as a light orange solid (53 mg, 63 % yield). **^{1}H NMR** (400 MHz, $CDCl_3$) δ 8.23 (d, *J* = 8.5 Hz, 2H), 8.09 (dd, *J* = 8.2, 1.3 Hz, 1H), 8.06 – 7.97 (m, 2H), 7.73 – 7.55 (m, 2H), 7.50 (ddd, *J* = 8.9, 7.3, 1.7 Hz, 1H), 5.29 (s, 2H), 4.74 (s, 2H), 3.98 (s, 3H); **^{13}C NMR** (101 MHz, $CDCl_3$) δ 165.5, 147.3, 141.1, 135.4, 133.9, 132.5, 130.5, 129.0, 125.1, 86.0, 72.1, 52.9; **HRMS** (ESI-TOF) m/z: $[M+H]^+$ calc'd for $C_{16}H_{16}NO_7S$ 366.0642; found: 366.0648.

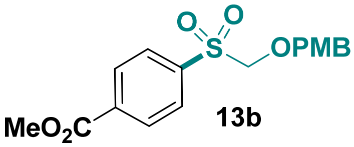

13b

Methyl 4-((((4-methoxybenzyl)oxy)methyl)sulfonyl)benzoate (13b)

According to general procedure A2, methyl 4-iodobenzoate (60.2 mg, 0.23 mmol), PMB-R-Na (82 mg, 0.34 mmol), **L** (3.7 mg), CuI (2.6 mg), and K_3PO_4 (49 mg, 0.23 mmol) in 0.6 mL DMSO yielded **13b** as a white solid (70 mg, 87 % yield). **^{1}H NMR** (400 MHz, $CDCl_3$) δ 8.22 (d, *J* = 8.5 Hz, 2H), 8.01 (d, *J* = 8.6 Hz, 2H), 7.21 (d, *J* = 8.7 Hz, 2H), 6.87 (d, *J* = 8.6 Hz, 2H), 4.84 (s, 2H), 4.56 (s, 2H), 3.97 (s, 3H), 3.81 (s, 3H); **^{13}C NMR** (101 MHz, $CDCl_3$) δ 165.6, 160.0, 141.4, 135.1, 130.3, 130.3, 129.0, 127.6, 114.1, 84.3, 74.4, 55.4, 52.8; **HRMS** (ESI-TOF) m/z: $[M+Na]^+$ calc'd for $C_{17}H_{18}NaO_6S$ 373.0716; found: 373.0722.

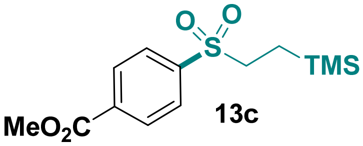

13c

Methyl 4-((2-(trimethylsilyl)ethyl)sulfonyl)benzoate (15c)

According to general procedure A3, methyl 4-iodobenzoate (60.2 mg, 0.23 mmol), SES-Na (65 mg, 0.34 mmol), **L** (3.7 mg), CuI (2.6 mg), and K_3PO_4 (49 mg, 0.23 mmol) in 0.6 mL DMSO yielded

13c as a white solid (65 mg, 94 % yield). **^{1}H NMR** (400 MHz, $CDCl_3$) δ 8.23 (d, *J* = 8.7 Hz, 2H), 7.99 (d, *J* = 8.7 Hz, 2H), 3.98 (s, 3H), 3.06 – 2.98 (m, 2H), 0.94 – 0.88 (m, 2H), 0.01 (s, 9H); **^{13}C NMR** (101 MHz, $CDCl_3$) δ 165.6, 142.7, 134.9, 130.4, 128.5, 52.8, 9.2, -2.0; **HRMS** (ESI-TOF) m/z: $[M+Na]^+$ calc'd for $C_{13}H_{20}NaO_4SSi$ 323.0744; found: 323.0748.

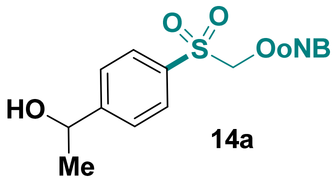

14a

1-(4-((((2-nitrobenzyl)oxy)methyl)sulfonyl)phenyl)ethan-1-ol (14a)

According to general procedure A4, 1-(4-iodophenyl)ethan-1-ol (57 mg, 0.23 mmol), oNB-R-Na (156 mg, 0.69 mmol), **L** (6.2 mg), CuI (4.4 mg), and K_3PO_4 (49 mg, 0.23 mmol) in 1.3 mL DMSO yielded **14a** as yellow oil (53 mg, 66 % yield). **^{1}H NMR** (400 MHz, $CDCl_3$) δ 8.08 (d, *J* = 8.2 Hz, 1H), 7.94 – 7.84 (d, *J* = 6.9 Hz, 2H), 7.64 (d, *J* = 5.4 Hz, 2H), 7.58 (d, *J* = 7.9 Hz, 2H), 7.53 – 7.48 (m, 1H), 5.21 (s, 2H), 5.07 – 4.97 (m, 1H), 4.71 (s, 2H), 1.98 (s, 1H), 1.54 (d, *J* = 6.5 Hz, 3H); **^{13}C NMR** (101 MHz, $CDCl_3$) δ 153.1, 147.3, 135.9, 134.0, 132.8, 129.2, 128.9, 126.4, 125.1, 86.2, 71.9, 69.7, 25.5; **HRMS** (ESI-TOF) m/z: $[M+H]^+$ calc'd for $C_{16}H_{18}NO_6S$ 352.0849; found: 352.0850.

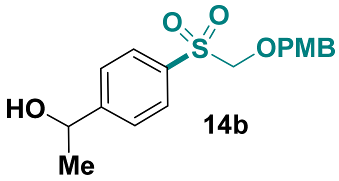

14b

1-(4-((((4-methoxybenzyl)oxy)methyl)sulfonyl)phenyl)ethan-1-ol (14b)

According to general procedure A2, 1-(4-iodophenyl)ethan-1-ol (57 mg, 0.23 mmol), PMB-R-Na (82 mg, 0.34 mmol), **L** (3.7 mg), CuI (2.6 mg), and K_3PO_4 (49 mg, 0.23 mmol) in 0.6 mL DMSO yielded **14b** as a white solid (68 mg, 88 % yield). **^{1}H NMR** (400 MHz, $CDCl_3$) δ 7.91 (dd, *J* = 8.3, 1.5 Hz, 2H), 7.57 (d, *J* = 8.3 Hz, 2H), 7.23 (d, *J* = 7.3 Hz, 2H), 6.87 (d, *J* = 8.5 Hz, 2H), 5.00 (q, *J* = 6.5 Hz, 1H), 4.84 (s, 2H), 4.53 (s, 2H), 3.81 (s, 3H) 1.94 (s, 1H), 1.52 (d, *J* = 6.5 Hz, 3H); **^{13}C NMR** (101 MHz, $CDCl_3$) δ 159.9, 152.8, 136.1, 130.3, 129.1, 127.9, 126.2, 114.1, 84.4, 74.3, 69.6, 55.4, 25.5; **HRMS** (ESI-TOF) m/z: $[M+Na]^+$ calc'd for $C_{17}H_{21}NaO_5S$ 359.0929; found: 359.0939.

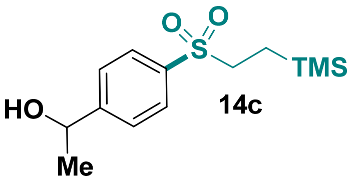

14c

1-(4-((2-(trimethylsilyl)ethyl)sulfonyl)phenyl)ethan-1-ol (14c)

According to general procedure A3, 1-(4-iodophenyl)ethan-1-ol (57 mg, 0.23 mmol), SES-Na (65 mg, 0.34 mmol), **L** (3.7 mg), CuI (2.6 mg), and K_3PO_4 (49 mg, 0.23 mmol) in 0.6 mL DMSO yielded **14c** as a white solid (59 mg, 90 % yield). **^{1}H NMR** (400 MHz, $CDCl_3$) δ 7.88 (d, *J* = 8.5 Hz, 2H), 7.58 (d, *J* = 8.3 Hz, 2H), 5.02 (d, *J* = 6.9 Hz, 1H), 3.17 – 2.87 (m, 2H), 1.96 (s, 1H), 1.64 – 1.43 (d, *J* = 6.4 Hz, 3H), 1.02 – 0.85 (m, 2H), 0.01 (s, 9H); **^{13}C NMR** (101 MHz, $CDCl_3$) δ 152.1, 137.6, 128.6, 126.3, 69.8, 52.9, 25.6, 9.2, -1.9; **HRMS** (ESI-TOF) m/z: $[M+Na]^+$ calc'd for $C_{13}H_{22}NaO_3SSi$ 309.0957; found: 309.0963.

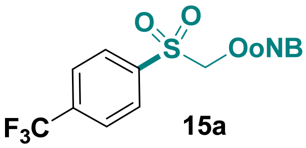

15a

1-nitro-2-((((4-(trifluoromethyl)phenyl)sulfonyl)methoxy)methyl)benzene (15a)

According to general procedure A4, 1-iodo-4-(trifluoromethyl)benzene (33.4 µL, 0.23 mmol), oNB-R-Na (156 mg, 0.69 mmol), **L** (6.2 mg), CuI (4.4 mg), and K_3PO_4 (49 mg, 0.23 mmol) in 1.3 mL DMSO yielded **15a** as a yellow solid (63 mg, 73 % yield). **^{1}H NMR** (400 MHz, $CDCl_3$) δ 8.16 – 8.04 (m, 3H), 7.85 (d, *J* = 8.2 Hz, 2H), 7.65 (td, *J* = 7.5, 1.3 Hz, 1H), 7.58 (d, *J* = 6.3 Hz, 1H), 7.51 (td, *J* = 7.7, 1.7 Hz, 1H), 5.32 (s, 2H), 4.74 (s, 2H); **^{13}C NMR** (101 MHz, $CDCl_3$) δ 147.5, 140.9, 136.0 (q, *J* = 33.2 Hz), 133.9, 132.3, 129.6, 129.2 (d, *J* = 3.2 Hz), 126.6 (q, *J* = 3.7 Hz), 125.2, 86.0, 72.2; **^{19}F NMR** (376 MHz, $CDCl_3$) δ -63.2; **HRMS** (ESI-TOF) m/z: $[M+H]^+$ calc'd for $C_{15}H_{13}F_3NO_5S$ 376.0461; found: 376.0464.

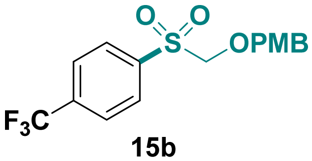

15b

1-methoxy-4-((((4-(trifluoromethyl)phenyl)sulfonyl)methoxy)methyl)benzene(15b)

According to general procedure A2, 1-iodo-4-(trifluoromethyl)benzene (33.4 µL, 0.23 mmol), PMB-R-Na (82 mg, 0.34 mmol), **L** (3.7 mg), CuI (2.6 mg), and K_3PO_4 (49 mg, 0.23 mmol) in 0.6 mL DMSO yielded **15b** as a light yellow solid (72 mg, 87 % yield). **^{1}H NMR** (400 MHz, $CDCl_3$) δ 8.07 (d,

J = 8.2 Hz, 2H), 7.83 (d, *J* = 8.1 Hz, 2H), 7.22 (d, *J* = 8.7 Hz, 2H), 6.88 (d, *J* = 8.7 Hz, 2H), 4.86 (s, 2H), 4.56 (s, 2H), 3.81 (s, 3H); **^{13}C NMR** (101 MHz, $CDCl_3$) δ 160.1, 141.1, 135.7 (q, *J* = 33.0 Hz), 130.3, 129.6, 128.7, 127.6, 126.4 (q, *J* = 3.7 Hz), 114.2, 84.2, 74.5, 55.4; **^{19}F NMR** (376 MHz, $CDCl_3$) δ -63.2; **HRMS** (ESI-TOF) m/z: $[M+Na]^+$ calc'd for $C_{16}H_{15}F_3NaO_4S$ 383.0541; found: 383.0543.

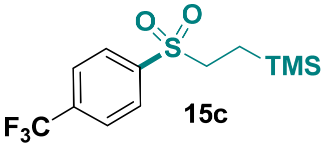

15c

trimethyl(2-((4-(trifluoromethyl)phenyl)sulfonyl)ethyl)silane (15c)

According to general procedure A3, 1-iodo-4-(trifluoromethyl)benzene (33.4 µL, 0.23 mmol), SES-Na (65 mg, 0.34 mmol), L (3.7 mg), CuI (2.6 mg), and K_3PO_4 (49 mg, 0.23 mmol) in 0.6 mL DMSO yielded **15c** as a white solid (69 mg, 96 % yield). **^{1}H NMR** (400 MHz, $CDCl_3$) δ 8.05 (d, *J* = 7.8 Hz, 2H), 7.85 (d, *J* = 8.1 Hz, 2H), 3.06 – 2.94 (m, 2H), 0.95 – 0.89 (m, 2H), 0.01 (s, 9H); **^{13}C NMR** (101 MHz, $CDCl_3$) δ 142.5, 135.4 (q, *J* = 33.1 Hz), 129.1, 126.5 (q, *J* = 3.7 Hz), 123.3 (d, *J* = 273.0 Hz), 52.9, 9.2, -2.0; **^{19}F NMR** (376 MHz, $CDCl_3$) δ -63.2; **HRMS** (ESI-TOF) m/z: $[M+Na]^+$ calc'd for $C_{12}H_{17}F_3NaO_2SSi$ 333.0568; found: 333.0570.

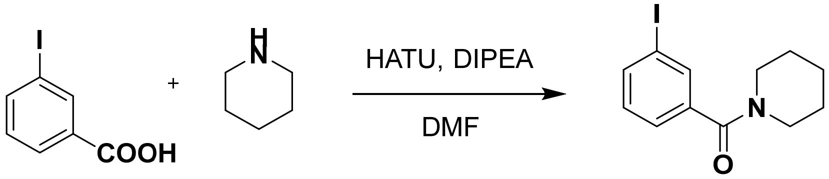

To the solution of 3-iodobenzoic acid (1 g, 4.03 mmol) in 20 mL DMF, was added *N,N*-Diisopropylethylamine (DIPEA) (2.1 mL, 12.09 mmol, 3 equiv) and 1-[Bis(dimethylamino)methylene]-1H-1,2,3-triazolo[4,5-b]pyridinium3-oxide hexafluorophosphate (HATU) (1.8 g, 4.83 mmol, 1.2 equiv). The resulting mixture was stirred at room temperature for 1 hour, after which piperidine (0.43 mL, 4.43 mmol, 1.1 equiv) was added and stirred overnight. Upon completion, the product was extracted with ethyl acetate and washed with brine. The organic extracts were dried over anhydrous Na_aSO_4, filtered, and concentrated under reduced pressure. The crude product was purified by column chromatography (eluting with ethyl acetate/hexanes) to obtain (3-iodophenyl)(piperidin-1-yl)methanone as a yellow solid (1.12 g, 88% yield).**^{1}H NMR** (400 MHz, $CDCl_3$) δ 7.73 (dh, *J* = 4.6, 1.3 Hz, 2H), 7.34 (dt, *J* = 7.6, 1.3 Hz, 1H), 7.17 – 7.10 (m, 1H), 3.68 (s, 2H), 3.32 (s, 2H), 1.69 – 1.59 (m, 4H), 1.52 (s, 2H); **^{13}C NMR** (101 MHz, $CDCl_3$) δ 168.0, 138.3, 138.0, 135.4, 129.9, 125.6, 94.0, 48.5, 42.9, 26.3, 25.3, 24.3; **HRMS** (ESI-TOF) m/z: $[M+H]^+$ calc'd for $C_{12}H_{15}INO$ 316.0193; found: 316.0202.

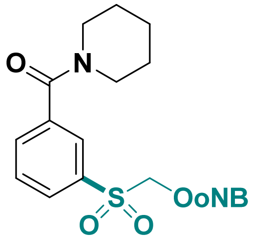

16a

(3-((((2-nitrobenzyl)oxy)methyl)sulfonyl)phenyl)(piperidin-1-yl)methanone (16a)

According to general procedure A4, (3-iodophenyl)(piperidin-1-yl)methanone (72.48 mg, 0.23 mmol), oNB-R-Na (156 mg, 0.69 mmol), **L** (6.2 mg), CuI (4.4 mg), and K_3PO_4 (49 mg, 0.23 mmol) in 1.3 mL DMSO yielded **16a** as a white solid (73 mg, 76 % yield). **^{1}H NMR** (400 MHz, $CDCl_3$) δ 8.09 (dd, *J* = 8.1, 1.3 Hz, 1H), 8.02 – 7.91 (m, 2H), 7.73 (d, *J* = 7.7 Hz, 1H), 7.71 – 7.56 (m, 3H), 7.58 – 7.42 (m, 1H), 5.30 (s, 2H), 4.72 (s, 2H), 3.71 (s, 2H), 3.28 (s, 2H), 1.68 (s, 4H), 1.50 (s, 2H); **^{13}C NMR** (101 MHz, $CDCl_3$) δ 168.0, 147.3, 138.0, 137.7, 134.0, 132.8, 132.7, 129.8 (d, *J* = 6.7 Hz), 129.1, 128.9, 127.3, 125.1, 86.0, 72.0, 48.9, 43.4, 26.6, 25.6, 24.5; **HRMS** (ESI-TOF) m/z: $[M+H]^+$ calc'd for $C_{20}H_{23}N_2O_6S$ 419.1271; found: 419.1293.

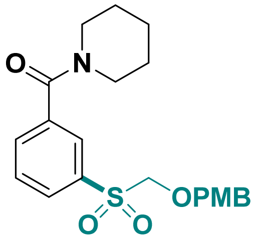

16b

(3-((((4-methoxybenzyl)oxy)methyl)sulfonyl)phenyl)(piperidin-1-yl)methanone (16b)

According to general procedure A2, (3-iodophenyl)(piperidin-1-yl)methanone (72.48 mg, 0.23 mmol), PMB-R-Na (82 mg, 0.34 mmol), **L** (3.7 mg), CuI (2.6 mg), and K_3PO_4 (49 mg, 0.23 mmol) in 0.6 mL DMSO yielded **16b** as a white solid (83 mg, 89 % yield). **^{1}H NMR** (400 MHz, $CDCl_3$) δ 8.00 – 7.89 (m, 2H), 7.71 (dt, *J* = 7.7, 1.4 Hz, 1H), 7.62 (t, *J* = 7.7 Hz, 1H), 7.23 (d, *J* = 8.7 Hz, 2H), 6.88 (d, *J* = 8.7 Hz, 2H), 4.84 (s, 2H), 4.53 (s, 2H), 3.81 (s, 3H), 3.71 (s, 2H), 3.25 (s, 2H), 1.67 (s, 4H), 1.48 (s, 2H); **^{13}C NMR** (101 MHz, $CDCl_3$) δ 168.0, 159.9, 137.8, 137.7, 132.6, 130.2, 129.6 (d, *J* = 2.1 Hz), 127.7, 127.2, 114.1, 84.3, 74.3, 55.3, 48.8, 43.3, 26.5, 25.5, 24.4; **HRMS** (ESI-TOF) m/z: $[M+H]^+$ calc'd for $C_{21}H_{26}NO_5S$ 404.1526; found: 404.1548.

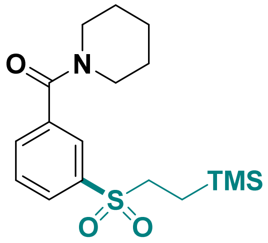

16c

Piperidin-1-yl(3-((2-(trimethylsilyl)ethyl)sulfonyl)phenyl)methanone (16c)

According to general procedure A3, (3-iodophenyl)(piperidin-1-yl)methanone (72.48 mg, 0.23 mmol), SES-Na (65 mg, 0.34 mmol), L (3.7 mg), CuI (2.6 mg), and K_3PO_4 (49 mg, 0.23 mmol) in 0.6 mL DMSO yielded **16c** as a white solid (75 mg, 92 % yield). **^{1}H NMR** (400 MHz, $CDCl_3$) δ 7.96 – 7.89 (m, 2H), 7.69 (dt, *J* = 7.7, 1.5 Hz, 1H), 7.63 (td, *J* = 7.6, 0.7 Hz, 1H), 3.73 (s, 2H), 3.30 (s, 2H), 3.08 – 2.85 (m, 2H), 1.69 (s, 4H), 1.53 (s, 2H), 1.01 – 0.84 (m, 2H), 0.01 (s, 9H); **^{13}C NMR** (101 MHz, $CDCl_3$) δ 168.2, 139.3, 137.9, 132.0, 129.7, 129.1, 126.6, 52.8, 48.9, 43.3, 26.5, 25.6, 24.4, 9.1, -2.0; **HRMS** (ESI-TOF) m/z: $[M+H]^+$ calc'd for $C_{17}H_{28}NO_3SSi$ 354.1554; found: 354.1570.

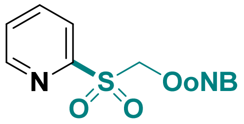

17a

2-((((2-nitrobenzyl)oxy)methyl)sulfonyl)pyridine (17a)

According to general procedure A4, 2-iodopyridine (25 µL, 0.23 mmol), oNB-R-Na (156 mg, 0.69 mmol), **L** (6.2 mg), CuI (4.4 mg), and K_3PO_4 (49 mg, 0.23 mmol) in 1.3 mL DMSO yielded **17a** as a yellow solid (29 mg, 41 % yield). **^{1}H NMR** (400 MHz, $CDCl_3$ δ 8.77 (dd, *J* = 4.7, 0.8 Hz, 1H), 8.12 (d, *J* = 7.8 Hz, 1H), 8.04 (dd, *J* = 8.2, 1.2 Hz, 1H), 7.97 (td, *J* = 7.8, 1.7 Hz, 1H), 7.63 – 7.53 (m, 3H), 7.44 (ddd, *J* = 8.6, 6.8, 2.1 Hz, 1H), 5.20 (s, 2H), 5.07 (s, 2H); **^{13}C NMR** (101 MHz, $CDCl_3$) δ 156.0, 150.5, 147.2, 138.2, 133.8, 132.7, 129.0, 128.7, 127.7, 124.9, 123.5, 82.8, 71.8; **HRMS** (ESI-TOF) m/z: $[M+H]^+$ calc'd for $C_{13}H_{13}N_2O_5S$ 309.0540; found: 309.0534.

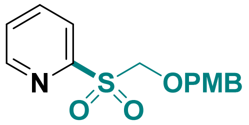

17b

2-((((4-methoxybenzyl)oxy)methyl)sulfonyl)pyridine (17b)

According to general procedure A2, 2-iodopyridine (25 µL, 0.23 mmol), PMB-R-Na (82 mg, 0.34 mmol), **L** (3.7 mg), CuI (2.6 mg), and K_3PO_4 (49 mg, 0.23 mmol) in 0.6 mL DMSO yielded **17b** as a clear oil (59 mg, 88 % yield). **^{1}H NMR** (400 MHz, $CDCl_3$) δ 8.76 (ddd, *J* = 4.7, 1.8, 1.0 Hz, 1H), 8.16 (dt, *J* = 7.9, 1.0 Hz, 1H), 7.98 (td, *J* = 7.8, 1.7 Hz, 1H), 7.56 (ddd, *J* = 7.6, 4.7, 1.2 Hz, 1H), 7.19 (d, *J* = 8.6 Hz, 2H), 6.85 (d, *J* = 8.7 Hz, 2H), 4.89 (s, 2H), 4.78 (s, 2H), 3.79 (s, 3H); **^{13}C NMR** (101 MHz, $CDCl_3$) δ 160.0, 156.2, 150.6, 138.2, 130.3, 127.9, 127.6, 123.7, 114.1, 81.2, 74.2, 55.4; **HRMS** (ESI-TOF) m/z: $[M+Na]^+$ calc'd for $C_{14}H_{15}NNaO_4S$ 316.0619; found: 316.0605.

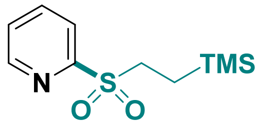

17c

2-((2-(trimethylsilyl)ethyl)sulfonyl)pyridine (17c)

According to general procedure A3, 2-iodopyridine (25 µL, 0.23 mmol), SES-Na (65 mg, 0.34 mmol), **L** (3.7 mg), CuI (2.6 mg), and K_3PO_4 (49 mg, 0.23 mmol) in 0.6 mL DMSO yielded **17c** as a white solid (50 mg, 90 % yield). **^{1}H NMR** (400 MHz, $CDCl_3$) δ 8.76 (ddd, *J* = 4.7, 1.7, 0.9 Hz, 1H), 8.11 (dt, *J* = 7.8, 1.1 Hz, 1H), 7.97 (td, *J* = 7.8, 1.7 Hz, 1H), 7.55 (ddd, *J* = 7.7, 4.7, 1.2 Hz, 1H), 3.37 – 3.22 (m, 2H), 1.06 – 0.80 (m, 2H), 0.03 (s, 9H); **^{13}C NMR** (101 MHz, $CDCl_3$) δ 157.1, 150.3, 138.2, 127.4, 122.6, 48.4, 8.4, -2.0; **HRMS** (ESI-TOF) m/z: $[M+H]^+$ calc'd for $C_{10}H_{18}NO_2SSi$ 244.0822; found: 244.0830.

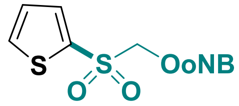

18a

2-((((2-nitrobenzyl)oxy)methyl)sulfonyl)thiophene (18a)

According to general procedure A4, 2-iodothiophene (23.4 µL, 0.23 mmol), oNB-R-Na (156 mg, 0.69 mmol), **L** (6.2 mg), CuI (4.4 mg), and K_3PO_4 (49 mg, 0.23 mmol) in 1.3 mL DMSO yielded **18a** as a yellow solid (49 mg, 68 % yield). **^{1}H NMR** (400 MHz, $CDCl_3$ δ 8.77 (dd, *J* = 4.7, 0.8 Hz, 1H), 8.12 (d, *J* = 7.8 Hz, 1H), 8.04 (dd, *J* = 8.2, 1.2 Hz, 1H), 7.97 (td, *J* = 7.8, 1.7 Hz, 1H), 7.63 – 7.53 (m, 3H), 7.44 (ddd, *J* = 8.6, 6.8, 2.1 Hz, 1H), 5.20 (s, 2H), 5.07 (s, 2H); **^{13}C NMR** (101 MHz, $CDCl_3$) δ 156.0, 150.5, 147.2, 138.2, 133.8, 132.7, 129.0, 128.7, 127.7, 124.9, 123.5, 82.8, 71.8; **HRMS** (ESI-TOF) m/z: $[M+Na]^+$ calc'd for $C_{12}H_{11}NNaO_5S_2$ 335.9976; found: 335.9982.

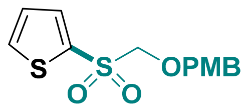

18b

2-((((4-methoxybenzyl)oxy)methyl)sulfonyl)thiophene (18b)

According to general procedure A2, 2-iodothiophene (23.4 µL, 0.23 mmol), PMB-R-Na (82 mg, 0.34 mmol), **L** (3.7 mg), CuI (2.6 mg), and K_3PO_4 (49 mg, 0.23 mmol) in 0.6 mL DMSO yielded **18b** as a white solid (60 mg, 89 % yield). ^{1}**H NMR** (400 MHz, $CDCl_3$) δ 8.76 (ddd, *J* = 4.7, 1.8, 1.0 Hz, 1H), 8.16 (dt, *J* = 7.9, 1.0 Hz, 1H), 7.98 (td, *J* = 7.8, 1.7 Hz, 1H), 7.56 (ddd, *J* = 7.6, 4.7, 1.2 Hz, 1H), 7.19 (d, *J* = 8.6 Hz, 2H), 6.85 (d, *J* = 8.7 Hz, 2H), 4.89 (s, 2H), 4.78 (s, 2H), 3.79 (s, 3H); ^{13}**C NMR** (101 MHz, $CDCl_3$) δ 7.82 – 7.69 (m, 2H), 7.28 – 7.24 (m, 2H), 7.18 (dd, *J* = 5.0, 3.8 Hz, 1H), 6.89 (d, *J* = 8.7 Hz, 2H), 4.87 (s, 2H), 4.60 (s, 2H), 3.81 (s, 3H); **HRMS** (ESI-TOF) m/z: $[M+Na]^+$ calc'd for $C_{13}H_{14}NaO_4S_2$ 321.0231; found: 321.0230.

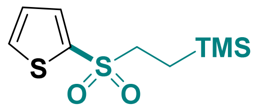

18c

trimethyl(2-(thiophen-2-ylsulfonyl)ethyl)silane (18c)

According to general procedure A3, 2-iodothiophene (23.4 µL, 0.23 mmol), SES-Na (65 mg, 0.34 mmol), **L** (3.7 mg), CuI (2.6 mg), and K_3PO_4 (49 mg, 0.23 mmol) in 0.6 mL DMSO yielded **18c** as a yellow solid (50 mg, 88 % yield). ^{1}**H NMR** (400 MHz, $CDCl_3$) δ 7.77 – 7.65 (m, 2H), 7.17 (dd, *J* = 5.0, 3.8 Hz, 1H), 3.19 – 2.97 (m, 2H), 1.06 – 0.90 (m, 2H), 0.02 (s, 9H); ^{13}**C NMR** (101 MHz, $CDCl_3$) δ 139.8, 134.2, 133.9, 128.0, 54.4, 9.7, -1.9; **HRMS** (ESI-TOF) m/z: $[M+Na]^+$ calc'd for $C_9H_{16}NaO_2S_2Si$ 271.0259; found: 271.0269.

Deprotection/fluorination of SPGs:

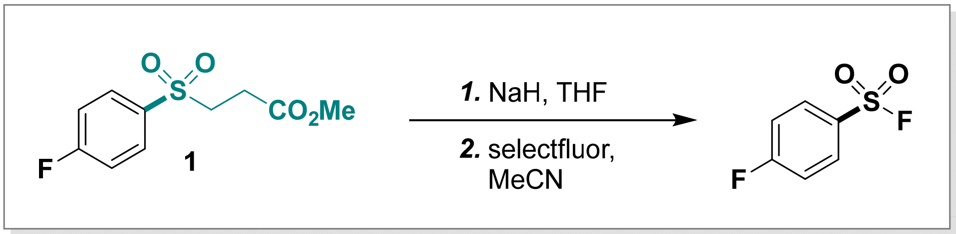

To the solution of **1** (25 mg, 0.1 mmol) in 2 mL THF, was added sodium hydride (4.4 mg, 0.11 mmol, 60% dispersion in oil, 1.1 equiv) at 0 °C. The resulting mixture was stirred at room temperature for 12 h. After the reaction is complete, remove the solvent under reduced pressure. Wash the residue with hexane (X3) to obtain sodium 4-fluorobenzenesulfinate as a white solid. To the vial containing the solution of selectfluor (53 mg, 0.15 equiv, 1.5 equiv) in 0.5

mL MeCN, was added sodium 4-fluorobenzenesulfinate (0.1 mmol). The resulting mixture was stirred at room temperature for 2 h. Upon completion, the reaction mixture was diluted with water and the product was extracted with ethyl acetate. The organic extracts were dried over anhydrous Na_2SO_4, filtered, and concentrated under reduced pressure. The crude product was purified by column chromatography (eluting with ethyl acetate/hexanes) to obtain 4-fluorobenzenesulfonyl fluoride as a clear oil (7.80 mg, 44% yield). The spectral data matched that of literature.[7]

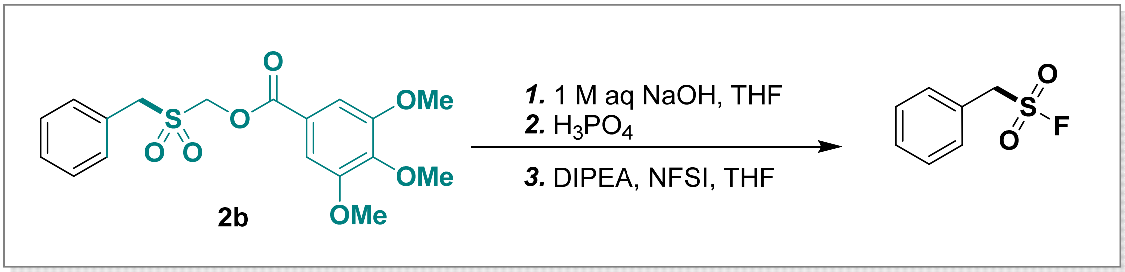

A mixture of **2b** (48 mg, 0.12 mmol) in 0.4 mL THF was treated with 1 M NaOH (0.3 mL, 0.3 mmol, 2.5 equiv), followed by stirring at room temperature for 24 h. 2 M H_3PO_4 (1.5 mL) and brine (0.5 mL) were then added to the mixture followed by extraction with ethyl acetate (2 X 1.5 mL). The combined organic extracts were transferred to a round-bottom flask. Under N_2, DIPEA (0.07 mmol, 0.36 mmol, 3 equiv) was added, the mixture was cooled to 0 °C, and a solution of NFSI (40 mg, 0.12 mmol, 1 equiv) in dry THF (0.5 mL) was added dropwise. Upon complete addition, the mixture was warmed to room temperature and the reaction was stirred for an additional 1 h. Without workup, silica gel was added to the reaction mixture, and all solvent was evaporated. The dried material was then purified via flash chromatography to give phenylmethanesulfonyl fluoride as a clear oil (16 mg, 76% yield). The spectral data matched that of literature.[8]

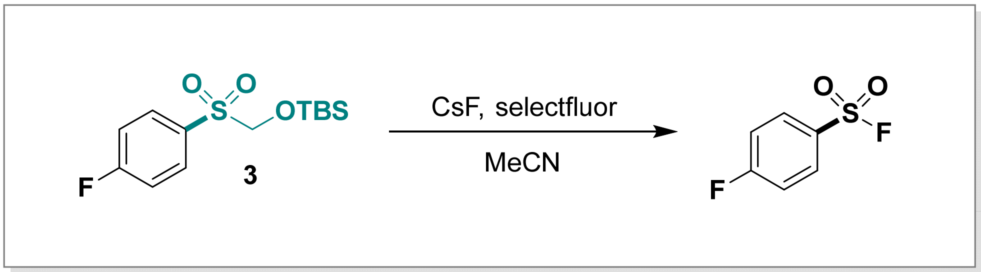

A mixture of **3** (20 mg, 0.07 mmol), CsF (15 mg, 0.09 mmol, 1.5 equiv), and selectfluor (47 mg, 0.13 mmol, 2.0 equiv) in 0.3 mL of anhydrous MeCN was prepared under argon atmosphere. The reaction mixture was stirred at room temperature for 24 h, and water was then added. The layers were separated, and the aqueous layer was extracted with ethyl acetate. The combined organic layers were washed with water and brine, dried over Na_2SO_4, and concentrated under reduced pressure. The crude product was purified by column chromatography (eluting with ethyl acetate/hexanes) to obtain 4-fluorobenzenesulfonyl fluoride as a clear oil (11 mg, 91% yield). The spectral data matched that of literature.[7]

Table S1. Optimization of PMB-R deprotection / fluorination[a

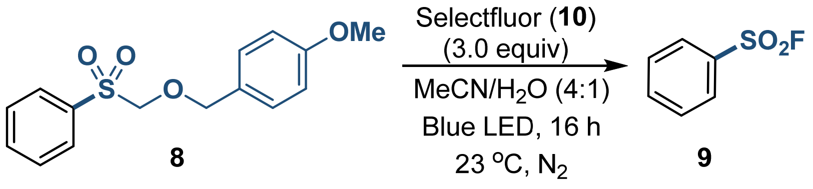

Entry	Variation from standard conditions	Yield (%)[b]
1	As above	97 (89%)[c]
2	2 mol% (Ir[dF(CF$_3$)ppy]$_2$(dtbpy))PF$_6$	85
3	2 mol% Mes-Acr catalyst	52
4	1.0 equiv of **10** instead of 3.0 equiv	10
5	2.0 equiv of **10** instead of 3.0 equiv	22
6	MeCN only, no H_2O	0
7	NFSI instead of **10**	17
8	No selectfluor	0
9	Open atmosphere	25
10	60 ˚C	trace
11	4.0 equiv TEMPO	0
12	No Light	0

[a] Conditions: **8** (0.2 mmol), **10** (3.0 equiv), (MeCN:H_2O (0.2 M), 23 °C, 16 h, N_2. [b] yields determined by ^{1}H NMR analysis with 4-fluoroacetophenone as an internal standard. [c] isolated yields are in parentheses, Mes-Acr = 9-Mesityl-3,6-di-tert-butyl-10-phenylacridinium tetrafluoroborate TEMPO = 2,2,6,6-tetramethyl-1-piperidinyloxy, NFSI = N-fluorobenzenesulfonimide

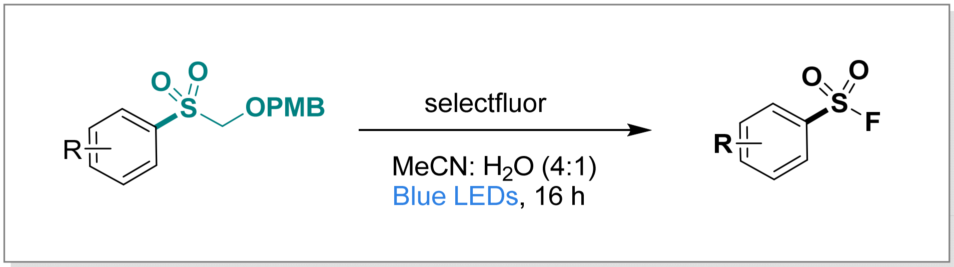

General Procedure D

A 1-dram vial was charged with sulfone (0.08 mmol) and selectfluor (90.9 mg, 0.25 mmol, 3 equiv). The vial was then evacuated and backfilled with argon (X3), and degassed MeCN:H_2O (1.6 mL, 4:1) was then added into the vial via syringe. The reaction was irradiated at 40-50 °C (radiant heating) at approximately 5.0 cm from a Kessil blue LED (λ_{max} = 440 nm, 100% intensity) for 16 h. Upon completion, the reaction mixture was diluted with water, and the product was extracted with ethyl acetate. The organic extracts were dried over anhydrous Na_aSO_4, filtered, and concentrated under reduced pressure. The crude product was purified by column chromatography (eluting with ethyl acetate/hexanes) to obtain the corresponding sulfonyl fluoride.

Note: Presence of air in the reaction mixture results in the formation of the oxidized benzoyl side product.

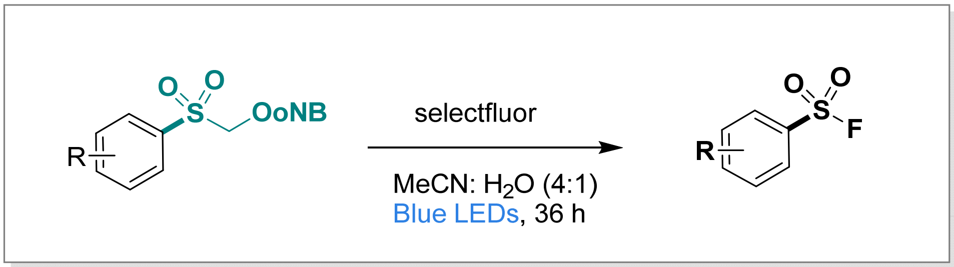

General Procedure E

A 1-dram vial was charged with sulfone (0.08 mmol) and selectfluor (142 mg, 0.4 mmol, 5 equiv). The vial was then evacuated and backfilled with argon (X3), and degassed MeCN:H_2O (1.6 mL, 4:1) were then added into the vial via syringe. The reaction was irradiated at 40-50 °C at approximately 5.0 cm from a Kessil blue LED (λ_{max} = 440 nm, 100% intensity) for 36 h. Upon completion, the reaction mixture was diluted with water, and the product was extracted with ethyl acetate. The organic extracts were dried over anhydrous Na_2SO_4, filtered, and concentrated under reduced pressure. The crude product was purified by column chromatography (eluting with ethyl acetate/hexanes) to obtain the corresponding sulfonyl fluoride.

Note: Presence of air in the reaction mixture results in the formation of the oxidized benzoyl side product.

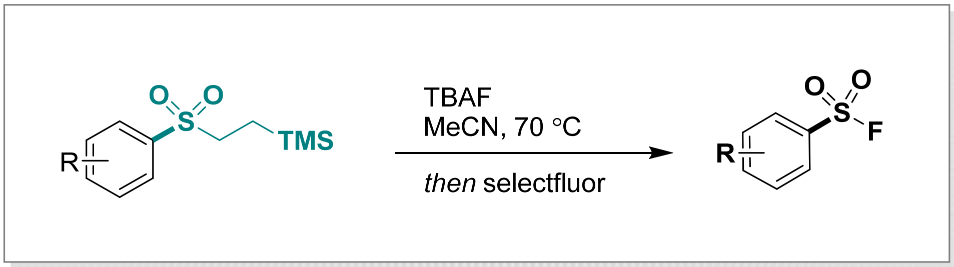

General Procedure F

A 1-dram vial was charged with sulfone (0.08 mmol) and tetra-n-butylammonium fluoride, TBAF (0.12 mL, 0.12 mmol, 1.5 equiv). The vial was then evacuated and backfilled with argon (X3), and degassed MeCN (0.6 mL) was then added into the vial via syringe. The reaction was stirred at 70 °C for 16 h, until the disappearance of starting material (as shown by TLC). The reaction was allowed to warm to room temperature and then selectfluor (57 mg, 0.16 mmol, 2 equiv) was added. The mixture was stirred at room temperature for 2 h. Upon completion, the reaction mixture was diluted with water and the product was extracted with ethyl acetate. The organic extracts were dried over anhydrous Na_2SO_4, filtered, and concentrated under reduced pressure. The crude product was purified by column chromatography (eluting with ethyl acetate/hexanes) to obtain the corresponding sulfonyl fluoride.

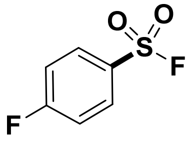

4-fluorobenzenesulfonyl fluoride (7)

Following general procedure E, **5** (26 mg, 0.08 mmol) gave the corresponding sulfonyl fluoride as a colorless oil (12 mg, 84% yield).
Following general procedure D, **4** (25 mg, 0.08 mmol) gave the corresponding sulfonyl fluoride as a colorless oil (12.30 mg, 86% yield).
Following general procedure F, **6** (21 mg, 0.08 mmol) gave the corresponding sulfonyl fluoride as a colorless oil (13 mg, 89% yield).
^{1}H NMR (400 MHz, $CDCl_3$) δ 8.15 – 8.00 (m, 2H), 7.32 (dd, J = 9.0, 7.9 Hz, 2H)
The spectral data matched that of literature.[7]

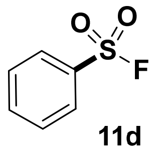

11d

Benzenesulfonyl fluoride (11d)

Following general procedure E, **11a** (25 mg, 0.08 mmol) gave the corresponding sulfonyl fluoride as a colorless oil (11 mg, 87% yield).
Following general procedure D, **11b** (24 mg, 0.08 mmol) gave the corresponding sulfonyl fluoride as a colorless oil (11.50 mg, 89% yield).
Following general procedure F, **11c** (25 mg, 0.08 mmol) gave the corresponding sulfonyl fluoride (97% NMR yield calculated using 4-fluoro acetophenone as an internal standard).
^{1}H NMR (400 MHz, $CDCl_3$) δ 8.03 (d, J = 7.9 Hz, 2H), 7.79 (t, J = 7.5 Hz, 1H), 7.64 (t, J = 7.7 Hz, 2H)
The spectral data matched that of literature.[7]

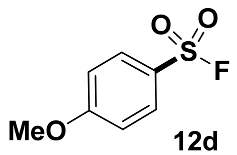

12d

4-methoxybenzenesulfonyl fluoride (12d)

Following general procedure E, **12a** (14 mg, 0.04 mmol) gave the corresponding sulfonyl fluoride as a colorless oil (5.55 mg, 73% yield).
Following general procedure D, **12b** (26 mg, 0.08 mmol) gave the corresponding sulfonyl fluoride as a colorless oil (9 mg, 59% yield).
Following general procedure F, **12c** (22 mg, 0.08 mmol) gave the corresponding sulfonyl fluoride (54% NMR yield calculated using 4-fluoro acetophenone as an internal standard).
^{1}H NMR (400 MHz, $CDCl_3$) δ 7.95 (d, J = 9.0 Hz, 2H), 7.06 (d, J = 8.9 Hz, 2H), 3.92 (s, 3H)
The spectral data matched that of literature.[7]

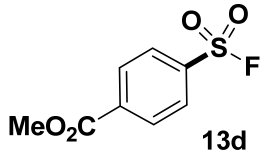

13d

Methyl 4-(fluorosulfonyl)benzoate (13d)

Following general procedure E, **13a** (29 mg, 0.08 mmol) gave the corresponding sulfonyl fluoride as a white solid (15.50 mg, 89% yield).
Following general procedure D, **13b** (28 mg, 0.08 mmol) gave the corresponding sulfonyl fluoride as a white solid (16 mg, 92% yield).

Following general procedure F, **13c** (24 mg, 0.08 mmol) gave the corresponding sulfonyl fluoride (98% NMR yield calculated using 4-fluoro acetophenone as an internal standard).
^{1}H NMR (400 MHz, $CDCl_3$) δ 8.28 (d, J = 8.2 Hz, 2H), 8.10 (d, J = 8.4 Hz, 2H), 4.00 (s, 3H)
The spectral data matched that of literature.[7]

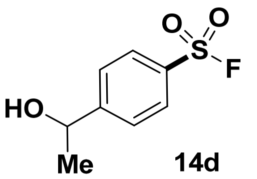

14d

4-(1-hydroxyethyl)benzenesulfonyl fluoride (14d)

Following general procedure E, **14a** (28 mg, 0.08 mmol) gave the corresponding sulfonyl fluoride as a white solid (14.2 mg, 82% yield).
Following general procedure D, **14b** (27 mg, 0.08 mmol) gave the corresponding sulfonyl fluoride as a white solid (14 mg, 87% yield).
Following general procedure F, **14c** (24 mg, 0.08 mmol) gave the corresponding sulfonyl fluoride (98% NMR yield calculated using 4-fluoro acetophenone as an internal standard).
^{1}H NMR (400 MHz, $CDCl_3$) δ 7.99 (d, *J* = 8.5 Hz, 2H), 7.64 (d, *J* = 8.1 Hz, 2H), 5.04 (q, *J* = 6.6 Hz, 1H), 2.15 – 1.92 (m, 1H), 1.53 (d, *J* = 6.6 Hz, 3H); **^{13}C NMR** (101 MHz, $CDCl_3$) δ 154.4, 131.9 (d, *J* = 24.5 Hz), 128.9, 126.7, 69.6, 25.7; **^{19}F NMR** (376 MHz, $CDCl_3$) δ 66.1; **HRMS** (ESI-TOF) m/z: $[M+H]^+$ calc'd for $C_8H_{10}FO_3S$ 205.0335; found: 205.0345.

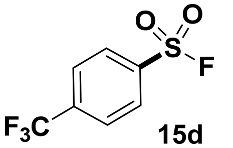

15d

4-(trifluoromethyl)benzenesulfonyl fluoride (15d)

Following general procedure E, **15a** (18 mg, 0.05 mmol) gave the corresponding sulfonyl fluoride as a white solid (8.6 mg, 84% yield).
Following general procedure D, **15b** (29 mg, 0.08 mmol) gave the corresponding sulfonyl fluoride as a white solid (14 mg, 86% yield).
Following general procedure F, **15c** (25 mg, 0.08 mmol) gave the corresponding sulfonyl fluoride (92% NMR yield calculated using 4-fluoro acetophenone as an internal standard).
^{1}H NMR (400 MHz, $CDCl_3$) δ 8.17 (d, J = 8.2 Hz, 2H), 7.92 (d, J = 8.2 Hz, 2H)
The spectral data matched that of literature.[7]

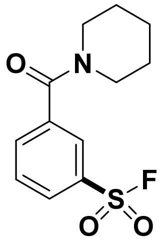

16d

3-(piperidine-1-carbonyl)benzenesulfonyl fluoride (16d)

Following general procedure E, **16a** (14 mg, 0.03 mmol) gave the corresponding sulfonyl fluoride as a white solid (7.2 mg, 89% yield).
Following general procedure D, **16b** (32.3 mg, 0.08 mmol) gave the corresponding sulfonyl fluoride as a white solid (21 mg, 97% yield).
Following general procedure F, **16c** (28 mg, 0.08 mmol) gave the corresponding sulfonyl fluoride (86% NMR yield calculated using 4-fluoro acetophenone as an internal standard).
^{1}H NMR (400 MHz, $CDCl_3$) δ 8.04 (d, *J* = 8.0 Hz, 2H), 7.80 (d, *J* = 7.7 Hz, 1H), 7.69 (t, *J* = 7.8 Hz, 1H), 3.72 (s, 2H), 3.31 (s, 2H), 1.70 (s, 4H), 1.55 (s, 2H); **^{13}C NMR** (101 MHz, $CDCl_3$) δ 167.4, 138.5, 134.0, 133.7 (d, *J* = 25.2 Hz), 130.2, 129.2, 127.0, 49.0, 43.6, 26.6, 25.6, 24.5; **^{19}F NMR** (376 MHz, $CDCl_3$) δ 66.0; **HRMS** (ESI-TOF) m/z: $[M+H]^+$ calc'd for $C_{12}H_{15}FNO_3S$ 272.0751; found: 272.0756.

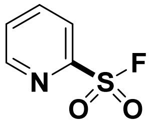

17d

Pyridine-2-sulfonyl fluoride (17d)

Following general procedure E, **17a** (25 mg, 0.08 mmol) gave the corresponding sulfonyl fluoride as a colorless oil (11.62 mg, 90% yield).
Following general procedure D, **17b** (15 mg, 0.05 mmol) gave the corresponding sulfonyl fluoride as a colorless oil (7 mg, 88% yield).
Following general procedure F, **17c** (20 mg, 0.08 mmol) gave the corresponding sulfonyl fluoride as a colorless oil (11.2 mg, 87% yield).
^{1}H NMR (400 MHz, $CDCl_3$) δ 8.86 (d, J = 4.3 Hz, 1H), 8.14 (d, J = 7.9 Hz, 1H), 8.11 – 7.98 (m, 1H), 7.82 – 7.63 (m, 1H)
The spectral data matched that of literature.[7]

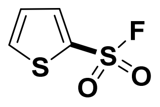

18d

Thiophene-2-sulfonyl fluoride (18d)

Following general procedure E, **18a** (11.3 mg, 0.08 mmol) gave the corresponding sulfonyl fluoride as a colorless oil (11.62 mg, 85% yield).
Following general procedure D, **18b** (24 mg, 0.08 mmol) gave the corresponding sulfonyl fluoride as a colorless oil (10.62 mg, 80% yield).
Following general procedure F, **18c** (20 mg, 0.08 mmol) gave the corresponding sulfonyl fluoride (95% NMR yield calculated using 4-fluoro acetophenone as an internal standard).
^{1}H NMR (400 MHz, $CDCl_3$) δ 7.86 (d, J = 3.9 Hz, 1H), 7.80 (d, J = 5.1 Hz, 1H), 7.17 (t, J = 5.1 Hz, 1H);.
The spectral data matched that of literature.[7]

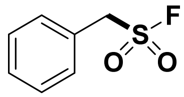

19d

Phenylmethanesulfonyl fluoride (19d)

Following general procedure E, **5a** (26 mg, 0.08 mmol) gave the corresponding sulfonyl fluoride as a colorless oil (11 mg, 79% yield).
Following general procedure D, **4a** (25 mg, 0.08 mmol) gave the corresponding sulfonyl fluoride as a colorless oil (11.2 mg, 80% yield).
Following general procedure F, **6a** (25.6 mg, 0.08 mmol) gave the corresponding sulfonyl fluoride as a colorless oil (10.8 mg, 77% yield).
^{1}H NMR (400 MHz, $CDCl_3$) δ 7.45 (m, 5H), 4.60 (d, J = 3.2 Hz, 2H)
The spectral data matched that of literature.[8]

One-pot synthesis of sulfonamides

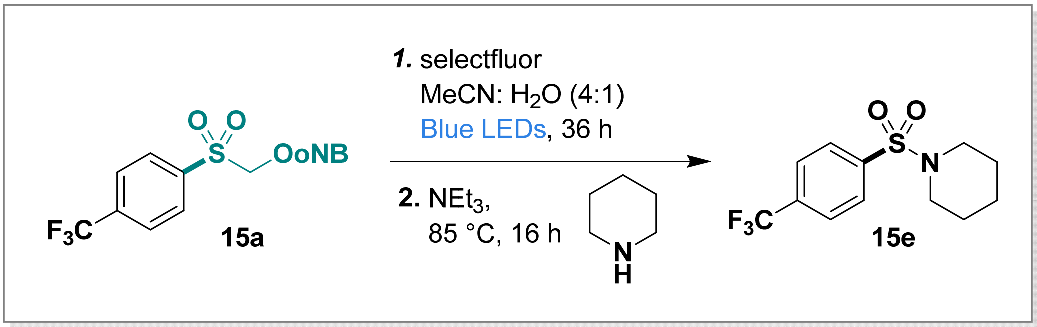

A vial was charged with **15a** (30 mg, 0.08 mmol) and selectfluor (142 mg, 0.4 mmol, 5 equiv). The vial was then evacuated and backfilled with argon (X3), and degassed MeCN:H_2O (1.6 mL, 4:1) was then added into the vial via syringe. The reaction was irradiated at 40-50 °C (radiant heating) at approximately 5.0 cm from a Kessil blue LED (λ_{max} = 440 nm, 100% intensity) for 36 h. Et_3N (55.6 µL, 0.4 mmol, 5 equiv) and piperidine (23.7 µL, 0.24 mmol, 3 equiv) were added to the reaction mixture and stirred at 85 °C for 16 h. Upon completion, the reaction mixture was diluted with water, and the product was extracted with ethyl acetate. The organic extracts were dried over anhydrous Na_2SO_4, filtered, and concentrated under reduced pressure. The crude product was purified by column chromatography (eluting with ethyl acetate/hexanes) to obtain the corresponding sulfonamide as a white solid (15 mg, 65% yield). **^{1}H NMR** (400 MHz, $CDCl_3$) δ 7.89 (d, *J* = 8.1 Hz, 2H), 7.80 (d, *J* = 8.2 Hz, 2H), 3.03 (t, *J* = 5.5 Hz, 4H), 1.66 (p, *J* = 5.7 Hz, 4H), 1.45 (tq, *J* = 8.8, 5.6, 4.5 Hz, 2H); **^{13}C NMR** (101 MHz, $CDCl_3$) δ 140.5, 134.4 (q, *J* = 33.1 Hz), 128.2, 126.3 (q, *J* = 3.7 Hz), 123.4 (d, *J* = 273.0 Hz), 47.0, 25.3, 23.6; **^{19}F NMR** (376 MHz, $CDCl_3$) δ -63.1; **HRMS** (ESI-TOF) m/z: $[M+H]^+$ calc'd for $C_{12}H_{15}F_3NO_2S$ 294.0784; found: 294.0784.

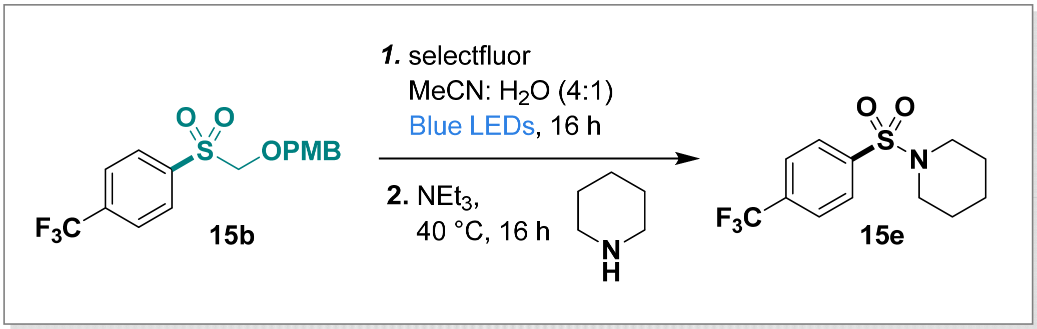

A vial was charged with **15b** (50 mg, 0.14 mmol) and selectfluor (148.79 mg, 0.42 mmol, 3 equiv). The vial was then evacuated and backfilled with argon (X3), and degassed MeCN:H_2O (2.8 mL, 4:1) was then added into the vial via syringe. The reaction was irradiated at 40-50 °C (radiant heating) at approximately 5.0 cm from a Kessil blue LED (λ_{max} = 440 nm, 100% intensity) for 16 h. Then Et_3N (98 µL, 0.7 mmol, 5 equiv) and piperidine (41.49 µL, 0.42 mmol, 3 equiv) were added

to the reaction mixture and stirred at 40 °C for 16 h. Upon completion, the reaction mixture was diluted with water, and the product was extracted with ethyl acetate. The organic extracts were dried over anhydrous Na_2SO_4, filtered, and concentrated under reduced pressure. The crude product was purified by column chromatography (eluting with ethyl acetate/hexanes) to obtain the corresponding sulfonamide as a white solid (26 mg, 64% yield).

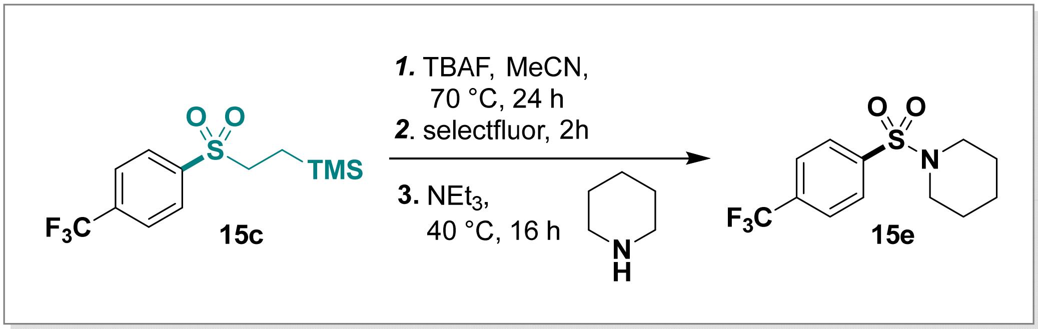

A 1-dram vial was charged with **15c** (49 mg, 0.158 mmol) and TBAF (0.24 mL, 0.237 mmol, 1.5 equiv). The vial was then evacuated and backfilled with argon (X3), and degassed MeCN (0.8 mL) was then added into the vial via syringe. The reaction was stirred at 70 °C for 16 h, until the disappearance of starting material (as shown by TLC). The reaction was allowed to warm to room temperature and then selectfluor (112 mg, 0.316 mmol, 2 equiv) was added. The mixture was stirred at room temperature for 2 h. Then Et_3N (110 µL, 0.79 mmol, 5 equiv) and piperidine (47 µL, 0.474 mmol, 3 equiv) were added to the reaction mixture and stirred at 40 °C for 16 h. Upon completion, the reaction mixture was diluted with water and the product was extracted with ethyl acetate. The organic extracts were dried over anhydrous Na_2SO_4, filtered, and concentrated under reduced pressure. The crude product was purified by column chromatography (eluting with ethyl acetate/hexanes) to obtain the sulfonamide as a white solid (25 mg, 54% yield).

oNB-R deprotection mechanism

The deprotection of the oNB-R sulfone was intended to proceed through a Norrish-type II mechanism as shown in the figure below.[9] The reality is that the mechanism is a bit more complex. Upon conclusion of the oNB-R sulfone deprotection/fluorination reaction, no aldehyde byproducts (ex. *ortho*-nitro or *ortho*-nitroso benzaldehyde) are observed in the reaction (in the PMB-R sulfone deprotection anisaldehyde is detected). Furthermore, byproducts consistent with Norrish type II deprotection mechanisms were observed via MS. Namely, we observed the diazo dicarboxylate **L**. This is a known byproduct that forms from the generation of nitroso species in a Norrish type-II mechanism.[10] Norrish type II mechanisms (specifically the conversion from **A** to **B**) are generally triggered by irradiation in the range of 200-370 nm, not irradiation at 440 nm.[11] If run in the absence of selectfluor, the reaction does not proceed, and the sulfinate intermediate (**H**) is not isolable. Additionally, it is worth noting the benzyl variant (Bn-R sulfone) does not deprotect under these conditions. Therefore, to explain both the Norrish type II byproducts and

these latter points, two mechanisms could be possible. First, the mechanism proceeds like the PMB-R sulfone mechanism (Figure 4) and the *ortho*-nitrobenzaldehyde could be subsequently converted to the observed byproducts via Norrish type mechanisms. Secondly, the selectfluor, or likely, the TEDA radical cation, could act as a shuttle (or assist in the conversion) between starting material **A** and intermediate **C**.

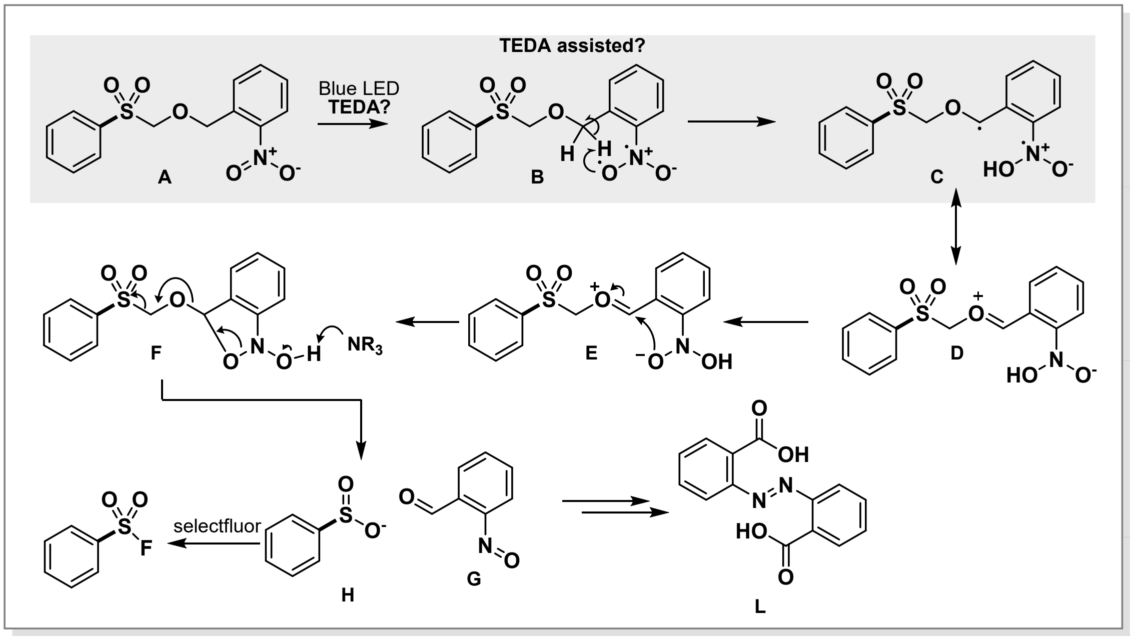

Synthesis of SPG Suzuki Building Blocks

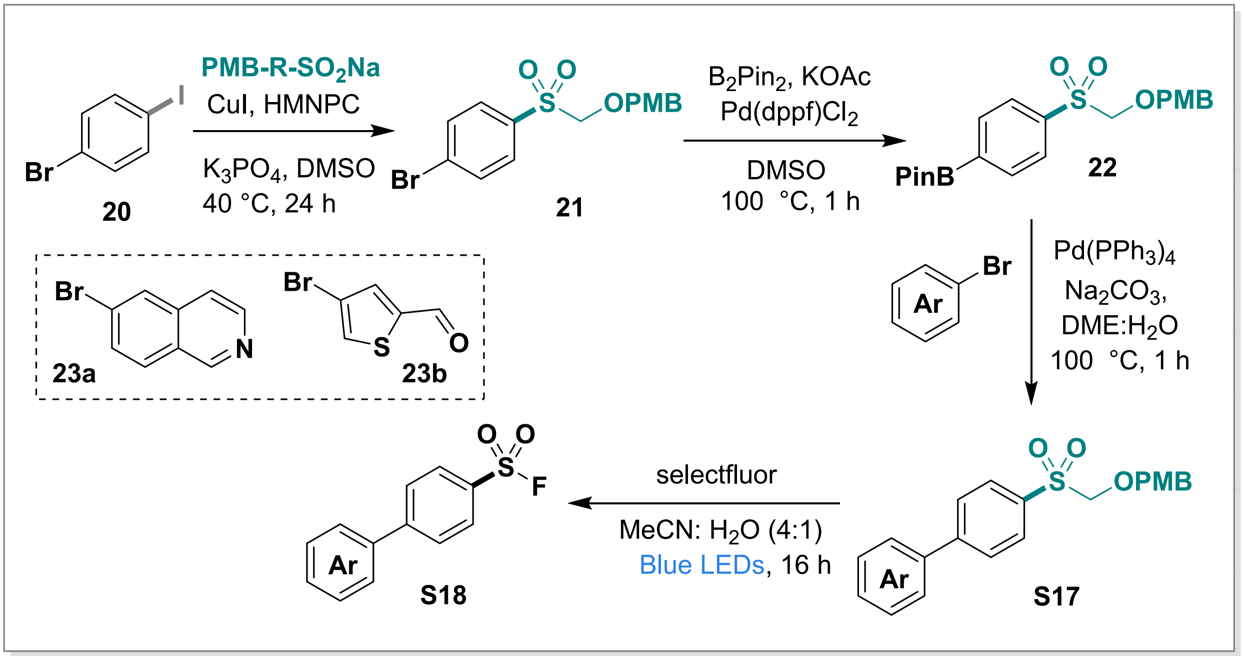

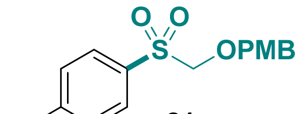

21

1-bromo-4-((((4-methoxybenzyl)oxy)methyl)sulfonyl)benzene (21)

According to general procedure A2, 1-bromo-4-iodobenzene (1.50 g, 5.30 mmol), PMB-R-Na (1.893 g, 7.95 mmol), **L** (143.3 mg), CuI (100.9 mg), and K_3PO_4 (1.125 g, 5.3 mmol) in 13.25 mL DMSO yielded **21** as a off-white solid (1.55 g, 79 % yield). **^{1}H NMR** (400 MHz, $CDCl_3$) δ 7.78 (d, J = 8.6 Hz, 2H), 7.70 (d, J = 8.3 Hz, 2H), 7.21 (d, J = 8.6 Hz, 2H), 6.88 (d, J = 8.6 Hz, 2H), 4.83 (s, 2H), 4.52 (s, 2H), 3.81 (s, 3H); **^{13}C NMR** (101 MHz, $CDCl_3$) δ 159.7, 136.4, 132.3, 130.2, 130.0, 129.2, 127.6, 113.9, 84.3, 74.2, 55.1; **HRMS** (ESI-TOF) m/z: $[M+Na]^+$ calc'd for $C_{15}H_{15}BrNaO_4S$ 392.9772; found: 392.9771.

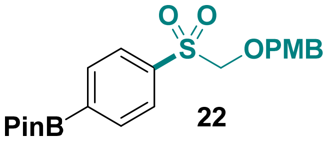

22

2-(4-((((4-methoxybenzyl)oxy)methyl)sulfonyl)phenyl)-4,4,5,5-tetramethyl-1,3,2-dioxaborolane (22)

A sealed microwave vial was charged with **21** (300 mg, 0.81 mmol), $Pd(dppf)Cl_2$.DCM (33.07 mg, 5 mol%), B_2Pin_2 (307 mg, 1.21 mmol, 1.5 equiv), and KOAc (238.49 mg, 2.43 mmol, 3 equiv). The

vial was then evacuated and backfilled with argon (X3) and degassed DMSO (8 mL) was then added into the vial via syringe. The reaction mixture was microwaved at 100 °C for 60 min. After cooling to room temperature, the mixture was filtered to remove excess salts. The reaction mixture was diluted with water and the product was extracted with DCM. The organic extracts were dried over anhydrous Na_2SO_4, filtered, and concentrated under reduced pressure. The crude product was used in the next step without further purification.

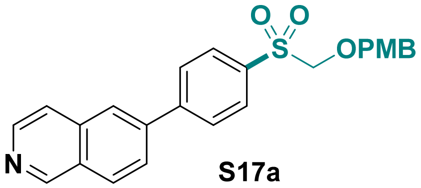

S17a

6-(4-((((4-methoxybenzyl)oxy)methyl)sulfonyl)phenyl)isoquinoline (S17a)

A sealed microwave vial was charged with **22** (167.32 mg, 0.4 mmol), 7-bromisoquinoline (124.83 mg, 0.6 mmol, 1.5 equiv), $Pd(PPh_3)_4$ (23.11 mg, 5 mol%), and Na_2CO_3 (127.19 mg, 1.2 mmol, 3 equiv). The vial was then evacuated and backfilled with argon (X3) and degassed DME: H_2O (4 Ml, 3:1) was then added into the vial via syringe. The reaction mixture was microwaved at 100 °C for 3 hours. After cooling to room temperature, the mixture was filtered hot to remove excess salts. The reaction mixture was diluted with water and the product was extracted with DCM. The organic extracts were dried over anhydrous Na_2SO_4, filtered, and concentrated under reduced pressure. The crude product was purified by high-performance liquid chromatography with a C18 column (Agilent 50 mm x 150 mm) and a gradient of acetonitrile/water to obtain **S17a** (154 mg, 92% yield). **^{1}H NMR** (400 MHz, $CDCl_3$) δ 9.81 (s, 1H), 8.66 (d, *J* = 6.4 Hz, 1H), 8.46 (d, *J* = 8.6 Hz, 1H), 8.29 (s, 1H), 8.23 (d, *J* = 6.4 Hz, 1H), 8.17 (d, *J* = 8.6 Hz, 1H), 8.14 (d, *J* = 8.1 Hz, 2H), 7.93 (d, *J* = 8.1 Hz, 2H), 7.27 (d, *J* = 7.7 Hz, 2H), 6.89 (d, *J* = 8.4 Hz, 2H), 4.91 (s, 2H), 4.62 (s, 2H), 3.81 (s, 3H); **^{13}C NMR** (101 MHz, $CDCl_3$) δ 160.1, 147.2, 146.9, 144.0, 138.9, 138.5, 133.2, 131.3, 130.4, 130.4, 130.0, 128.8, 127.7, 127.2, 125.7, 125.2, 114.2, 84.5, 74.5, 55.4; **HRMS** (ESI-TOF) m/z: $[M+H]^+$ calc'd for $C_{24}H_{22}NO_4S$ 420.1270; found: 420.1272.

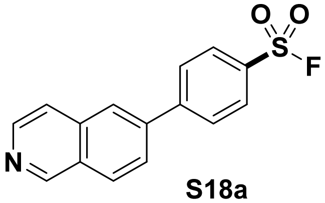

S18a

4-(isoquinolin-6-yl)benzenesulfonyl fluoride (S18a)

A vial was charged with **S16a** (40 mg, 0.09 mmol) and selectfluor (101.34 mg, 0.29 mmol, 3 equiv). The vial was then evacuated and backfilled with argon (X3), and degassed MeCN:H_2O (1.8 mL,

4:1) was then added into the vial via syringe. The reaction was irradiated at 40-50 °C (radiant heating) at approximately 5.0 cm from a Kessil blue LED (λ_{max} = 440 nm, 100% intensity) for 16 h. Upon completion, the reaction mixture was diluted with water, and the product was extracted with ethyl acetate. The organic extracts were dried over anhydrous Na_2SO_4, filtered, and concentrated under reduced pressure. The crude product was purified by column chromatography (eluting with ethyl acetate/hexanes) to obtain **S18a** as a yellow solid (20 mg, 76% yield); **^{1}H NMR** (400 MHz, CD_3CN) δ 9.57 (bs, 1H), 8.77 (bs, 1H), 8.48 (s, 1H), 8.45 – 8.38 (m, 3H), 8.30 (d, *J* = 8.3 Hz, 2H), 8.21 (dd, *J* = 8.6, 1.8 Hz, 1H), 8.08 (d, *J* = 5.5 Hz, 1H); **^{13}C NMR** (101 MHz, CD_3CN) zδ 152.9, 148.6, 143.9, 141.4, 137.1, 132.8 (d, *J* = 24.6 Hz), 130.2, 130.1, 129.9, 129.4*, 127.9, 126.8, 122.2*; **HRMS** (ESI-TOF) m/z: [M+H]$^+$ calc'd for $C_{15}H_{11}FNO_2S$ 288.0495; found: 288.0509.

Note: Carbons 4 and 5 (*) of the isoquinoline do not show up in the standard ^{13}C spectrum due to broadening; they are however visible in the HSQC and HMBC and are in agreement with the shifts for those carbons in the literature ^{13}C of isoquinoline.

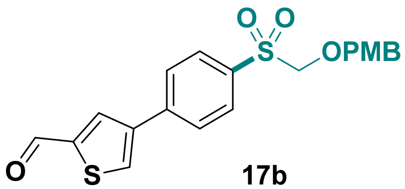

17b

4-(4-((((4-methoxybenzyl)oxy)methyl)sulfonyl)phenyl)thiophene-2-carbaldehyde (S17b)

A sealed microwave vial was charged with **22** (167.32 mg, 0.4 mmol), 4-bromothiophene-2-carbaldehyde (114.63 mg, 0.6 mmol, 1.5 equiv), $Pd(PPh_3)_4$ (23.11 mg, 5 mol%), and Na_2CO_3 (127.19 mg, 1.2 mmol,3 equiv). The vial was then evacuated and backfilled with argon (X3) and degassed DME: H_2O (4 Ml, 3:1) was then added into the vial via syringe. The reaction mixture was microwaved at 100 °C for 3 hours. After cooling to room temperature, the mixture was filtered hot to remove excess salts. The reaction mixture was diluted with water and the product was extracted with DCM. The organic extracts were dried over anhydrous Na_2SO_4, filtered, and concentrated under reduced pressure. The crude product was purified by high-performance liquid chromatography with a C18 column (Agilent 50 mm x 150 mm) and a gradient of acetonitrile/water to obtain **S17b** as a yellow solid (143 mg, 89% yield). **^{1}H NMR** (400 MHz, $CDCl_3$) δ 10.00 (s, 1H), 8.07 (d, *J* = 1.5 Hz, 1H), 8.00 (d, *J* = 8.2 Hz, 3H), 7.24 (d, *J* = 9.7 Hz, 2H), 6.88 (d, *J* = 8.6 Hz, 2H), 4.87 (s, 2H), 4.57 (s, 2H), 3.81 (s, 3H); **^{13}C NMR** (101 MHz, $CDCl_3$) δ 183.0, 160.0, 145.1, 141.6, 139.9, 136.6, 134.5, 131.7, 130.3, 129.8, 127.8, 127.0, 114.2, 84.5, 74.4, 55.4; **HRMS** (ESI-TOF) m/z: [M+NH_4]$^+$ calc'd for $C_{20}H_{22}NO_5S_2$ 420.1270; found: 420.1272.

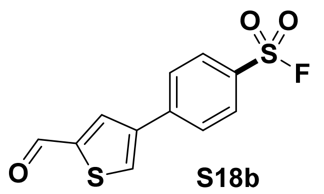

S18b

4-(5-formylthiophen-3-yl)benzenesulfonyl fluoride (S18b)

A vial was charged with **S17b** (38 mg, 0.09 mmol) and selectfluor (101.34 mg, 0.29 mmol, 3 equiv). The vial was then evacuated and backfilled with argon (X3), and degassed MeCN:H_2O (1.8 mL, 4:1) was then added into the vial via syringe. The reaction was irradiated at 40-50 °C (radiant heating) at approximately 5.0 cm from a Kessil blue LED (λ_{max} = 440 nm, 100% intensity) for 16 h. Upon completion, the reaction mixture was diluted with water, and the product was extracted with ethyl acetate. The organic extracts were dried over anhydrous Na_2SO_4, filtered, and concentrated under reduced pressure. The crude product was purified by column chromatography (eluting with ethyl acetate/hexanes) to obtain **S18b** as a white solid (20 mg, 83% yield). **^{1}H NMR** (400 MHz, $CDCl_3$) δ 10.01 (d, *J* = 1.3 Hz, 1H), 8.19 – 8.06 (m, 3H), 8.03 (t, *J* = 1.5 Hz, 1H), 7.84 (d, *J* = 8.3 Hz, 2H); **^{13}C NMR** (101 MHz, $CDCl_3$) δ 182.7, 145.5, 141.2 (d, *J* = 55.6 Hz), 134.0, 132.3, 132.1, 132.0, 129.5, 127.4; **^{19}F NMR** (376 MHz, $CDCl_3$) δ 66.4; **HRMS** (ESI-TOF) m/z: $[M+CH_3CN+H]^+$ calc'd for $C_{12}H_{11}FNO_3S_2$ 312.0164; found: 312.0171.

Synthesis of sulfonamide building block for AZD2858 analog

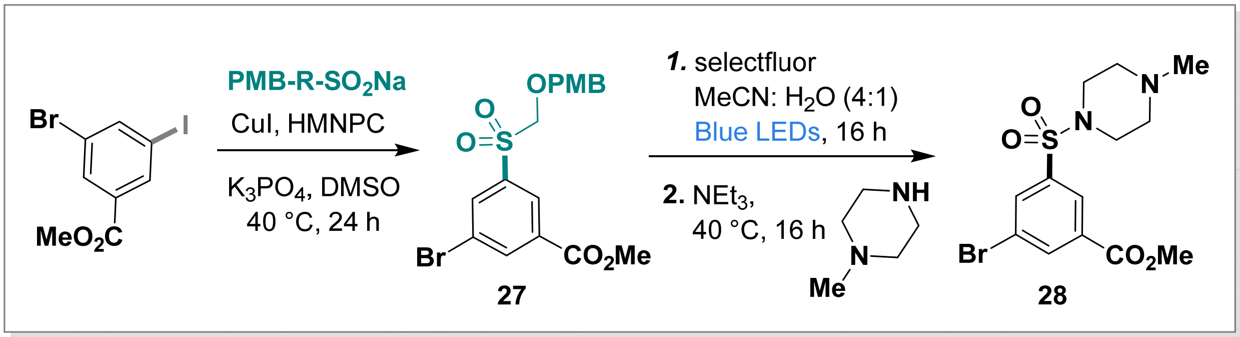

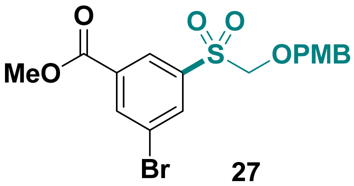

27

methyl 3-bromo-5-((((4-methoxybenzyl)oxy)methyl)sulfonyl)benzoate (27)

According to general procedure A2, methyl 3-bromo-5-iodobenzoate (267 mg, 0.78 mmol), PMB-R-Na (279.85 mg, 1.17 mmol), **L** (21.08 mg), CuI (14.85 mg), and K_3PO_4 (165.57 mg, 0.78 mmol) in 2 mL DMSO yielded **27** as a clear oil (245 mg, 73 % yield). **^{1}H NMR** (400 MHz, $CDCl_3$) δ 8.48 (s, 1H), 8.44 (s, 1H), 8.22 (s, 1H), 7.22 (d, *J* = 8.6 Hz, 2H), 6.88 (d, *J* = 8.7 Hz, 2H), 4.85 (s, 2H), 4.57 (s, 2H), 3.96 (s, 3H), 3.81 (s, 3H); **^{13}C NMR** (101 MHz, $CDCl_3$) δ 164.1, 160.0, 139.8, 137.8, 135.6, 133.1, 130.3, 128.5, 127.4, 123.4, 114.1, 84.1, 74.4, 55.3, 53.0; **HRMS** (ESI-TOF) m/z: $[M+NH_4]^+$ calc'd for $C_{17}H_{21}BrNO_6S$ 446.0267; found: 446.0265.

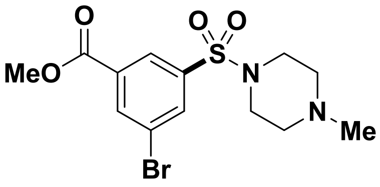

28

methyl 3-bromo-5-((4-methylpiperazin-1-yl)sulfonyl)benzoate (28)

A vial was charged with **27** (95 mg, 0.22 mmol) and selectfluor (235.19 mg, 0.66 mmol, 3 equiv). The vial was then evacuated and backfilled with argon (X3), and degassed MeCN:H_2O (4.4 mL, 4:1) was then added into the vial via syringe. The reaction was irradiated at 40-50 °C (radiant heating) at approximately 5.0 cm from a Kessil blue LED (λ_{max} = 440 nm, 100% intensity) for 16 h. Then Et_3N (0.15 mL, 1.1 mmol, 5 equiv) and 1-methylpiperazine (73.21 µL, 0.66 mmol, 3 equiv) were added to the reaction mixture and stirred at 40 °C for 16 h. Upon completion, the reaction mixture was diluted with water, and the product was extracted with ethyl acetate. The organic extracts were dried over anhydrous Na_2SO_4, filtered, and concentrated under reduced pressure. The crude product was purified by column chromatography (eluting with ethyl acetate/hexanes) to obtain the corresponding sulfonamide **28** as a white solid (35 mg, 42% yield); **^{1}H NMR** (400 MHz, $CDCl_3$) δ 8.38 (s, 1H), 8.30 (s, 1H), 8.06 (s, 1H), 3.97 (s, 3H), 3.10 (s, 4H), 2.51 (s, 4H); **^{13}C NMR** (101 MHz, $CDCl_3$) δ 164.4, 138.3, 136.8, 134.5, 133.1, 127.3, 123.5, 54.0, 53.1, 46.1, 45.8; **HRMS** (ESI-TOF) m/z: $[M+H]^+$ calc'd for $C_{13}H_{18}BrN_2O_4S$ 377.0171; found: 377.0179.

Synthesis of BCl-6 inhibitor analog

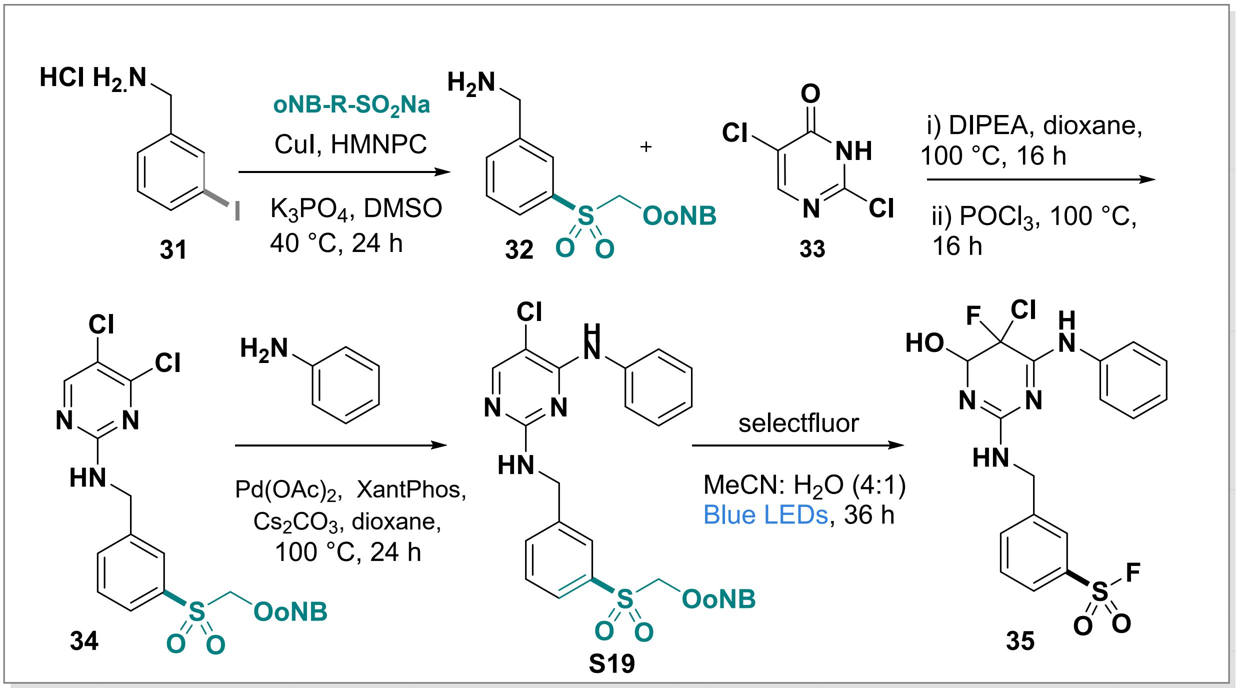

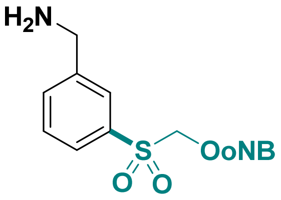

32

(3-((((2-nitrobenzyl)oxy)methyl)sulfonyl)phenyl)methanamine (32)

According to general procedure A4, **31** (754 mg, 2.798 mmol), oNB-R-Na (2 g, 8.39 mmol), **L** (75.69 mg), CuI (53.29 mg), and K_3PO_4 (1.20 g, 2.798 mmol) in 7 mL DMSO yielded **32** as a yellow solid (537 mg, 57 % yield). **^{1}H NMR** (400 MHz, $CDCl_3$ δ 8.07 (d, *J* = 8.2 Hz, 1H), 7.91 (s, 1H), 7.81 (d, *J* = 7.8 Hz, 1H), 7.65 (dd, *J* = 11.9, 6.0 Hz, 3H), 7.58 – 7.44 (m, 2H), 5.24 (s, 2H), 4.71 (s, 2H), 3.98 (s, 2H); **^{13}C NMR** (101 MHz, $CDCl_3$) δ 147.2, 137.4, 133.7, 133.0, 132.7, 129.4, 129.0, 128.8, 127.2, 127.1, 124.8, 86.0, 71.7; **HRMS** (ESI-TOF) m/z: $[M+H]^+$ calc'd for $C_{15}H_{17}N_2O_5S$ 337.0858; found: 337.0861.

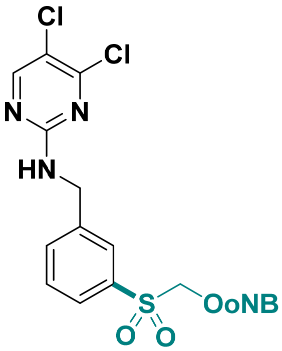

34

4,5-dichloro-N-(3-((((2-nitrobenzyl)oxy)methyl)sulfonyl)benzyl)pyrimidin-2-amine (34)

To the solution of **32** (370 mg, 1.10 mmol, 1.2 equiv) and **33** (151.22 mg, 0.92 mmol) in 12 mL 1,4-dioxane, was added 0.2 mL DIPEA. The resulting mixture was stirred at 100 °C for 16 hours. After completion of reaction, the reaction mixture was allowed to warm at room temperature and then $POCl_3$ (0.26 mL, 2.76 mmol, 3 equiv) was added. The resulting mixture was stirred for 2 hours at 100 °C. The product was extracted with CH_2Cl_2 and washed with brine. The organic extracts were dried over anhydrous Na_2SO_4, filtered, and concentrated under reduced pressure. The crude product was purified by column chromatography (eluting with ethyl acetate/hexanes) to obtain **34** as a yellow solid (334 mg, 75% yield); **^{1}H NMR** (400 MHz, $CDCl_3$) δ 8.07 (d, J = 8.1 Hz, 1H), 7.84 (d, J = 7.7 Hz, 1H), 7.70 – 7.44 (m, 5H), 5.80 (s, 1H), 5.22 (s, 2H), 4.69 (m, 4H); **^{13}C NMR** (101 MHz, $CDCl_3$) δ 160.0, 158.4, 157.8, 147.3, 140.5, 137.7, 133.9, 133.5, 132.6, 129.7, 129.1, 127.9, 125.0, 117.2, 86.2, 71.9, 45.1; **HRMS** (ESI-TOF) m/z: $[M+H]^+$ calc'd for $C_{19}H_{17}Cl_2N_4O_5S$ 483.0297; found: 483.0286.

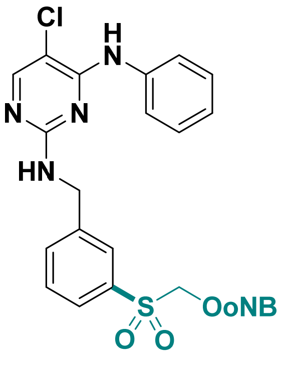

S19

5-chloro-*N*^{2}-(3-((((2-nitrobenzyl)oxy)methyl)sulfonyl)benzyl)-*N*^{4}-phenylpyrimidine-2,4-diamine (S19)

A sealed microwave vial was charged with **34** (300 mg, 0.62 mmol, 2 equiv), aniline (28.34 µL, 0.31 mmol), $Pd(OAc)_2$ (6.26 mg, 9 mol%), XantPhos (17.94 mg, 10 mol%), and Cs_2CO_3 (141.40 mg,

0.43 mmol,1.4 equiv). The vial was then evacuated and backfilled with argon (X3), and degassed dioxane (0.8 mL) was then added into the vial via syringe. The reaction mixture was stirred at 110 °C in an oil bath for 24 h. After cooling to room temperature, the mixture was filtered while hot to remove excess salts. The filtrate was concentrated under reduced pressure, and the resulting residue was purified by flash chromatography (eluting with ethyl acetate/hexanes) to afford **S19** as a light yellow solid (160 mg, 96% yield).^{1}**H NMR** (400 MHz, $CDCl_3$) δ 8.05 (d, J = 8.2 Hz, 1H), 7.92 (s, 1H), 7.91 (s, 1H), 7.82 (d, J = 7.8 Hz, 1H), 7.69 – 7.39 (m, 7H), 7.28 (m, 1H), 7.07 (t, J = 7.4 Hz, 1H), 6.98 (s, 1H), 5.59 (s, 1H), 5.18 (s, 2H), 4.64 (m, 4H); ^{13}**C NMR** (101 MHz, $CDCl_3$) δ 160.1, 155.5, 154.1, 146.9, 141.6, 138.0, 137.3, 133.8, 133.1, 132.6, 128.8, 128.7, 127.4, 127.3, 124.8, 123.8, 120.8, 85.9, 71.7, 45.0; **HRMS** (ESI-TOF) m/z: $[M+H]^+$ calc'd for $C_{25}H_{23}ClN_5O_5S$ 540.1108; found: 540.1114.

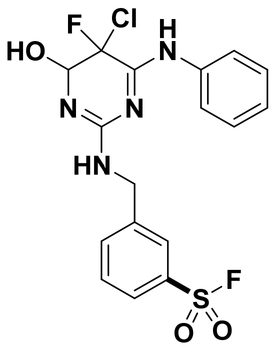

35

3-(((5-chloro-5-fluoro-4-hydroxy-6-(phenylamino)-4,5-dihydropyrimidin-2-yl)amino)methyl)benzenesulfonyl fluoride (35)

A vial was charged with **S17** (80 mg, 0.15 mmol) and selectfluor (265.69 mg, 0.75 mmol, 5 equiv). The vial was then evacuated and backfilled with argon (X3), and degassed MeCN:H_2O (3 mL, 4:1) were then added into the vial via syringe. The reaction was irradiated at 40-50 °C at approximately 5.0 cm from a Kessil blue LED (λ_{max} = 440 nm, 100% intensity) for 36 h. Upon completion, the reaction mixture was diluted with water, and the product was extracted with ethyl acetate. The organic extracts were dried over anhydrous Na_2SO_4, filtered, and concentrated under reduced pressure. The crude product was purified by high-performance liquid chromatography with a C18 column (Agilent 50 mm x 150 mm) and a gradient of acetonitrile/water with 0.1% TFA to obtain the TFA salt of **35** as a yellow solid (34 mg, 41% yield). ^{1}**H NMR** (400 MHz, CD_3CN) δ 11.63 (s, 1H), 10.98 (s, 1H), 7.94 (d, *J* = 7.7 Hz, 1H), 7.90 (s, 1H), 7.68 (dt, *J* = 15.4, 7.9 Hz, 2H), 7.57 (d, *J* = 8.0 Hz, 2H), 7.41 (dd, *J* = 8.5, 6.9 Hz, 2H), 7.33 (t, *J* = 7.3 Hz, 1H), 5.42 (s, 1H), 4.71 (s, 2H); ^{13}**C NMR** (101 MHz, CD_3CN) δ 160.7 (d, *J* = 19.8 Hz), 157.9, 141.6, 135.9, 135.8, 133.7 (d, *J* = 24.3 Hz), 131.5, 130.1, 128.8, 128.4, 127.6, 124.4, 97.9 (d, *J* = 266.0 Hz), 77.5 (d, *J* = 26.3 Hz), 45.5 0; ^{19}**F NMR** (376 MHz, $CDCl_3$) δ 64.7, -138.7; **HRMS** (ESI-TOF) m/z: $[M+H]^+$ calc'd for $C_{17}H_{16}ClF_2N_4O_3S$ 429.0600; found: 429.0603.

Plausible Mechanism:

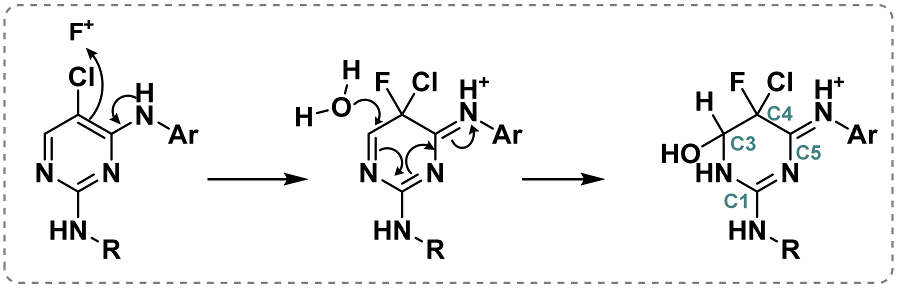

Upon fluorination of the aminopyrimidine at the C4 position, the corresponding iminium is then attacked by water at the C3 position. The addition of water and fluoride is supported by HRMS. Addition to the C3 position is supported by HMBC correlations between the C3 proton and carbons at C1, C4, and C5. No other HMBC correlations with other ring systems are observed. Splitting (J = 266.0 Hz) in the ^{13}C NMR at C4 as well as the fluoride shift is indicative of a geminal fluorochloride.[12] This fluorination is most likely driven by the aryl amino group. Efforts to temper the reactivity of the amino through protecting groups were not successful.

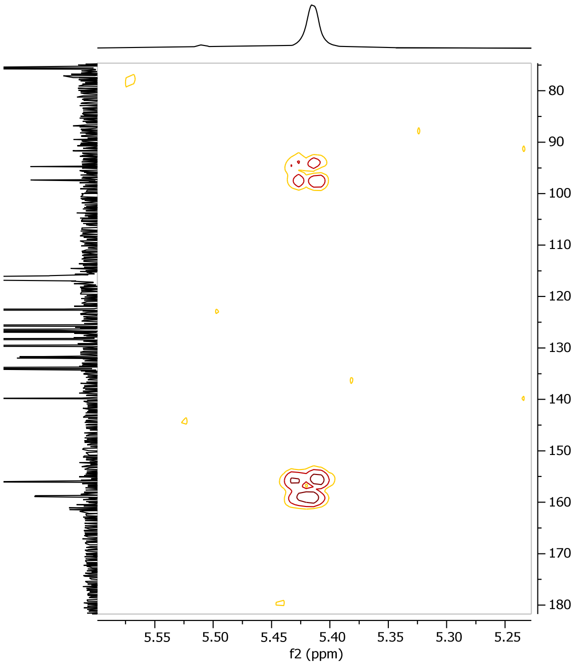

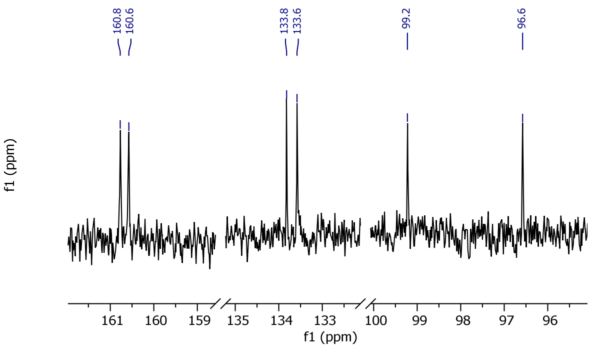

Left: HMBC showing correlations with C1, C4, C5. Right: ^{13}C spectrum of fluoride coupled peaks: ^{13}C NMR (101 MHz, CD_3CN) δ 160.7 (d, $J_{C5\text{-}F}$ = 19.8 Hz), 133.7 (d, $J_{C3\text{-}F}$ = 24.2 Hz), 97.9 (d, $J_{C4\text{-}F}$ = 266.0 Hz). See page 159 for full characterization spectra.

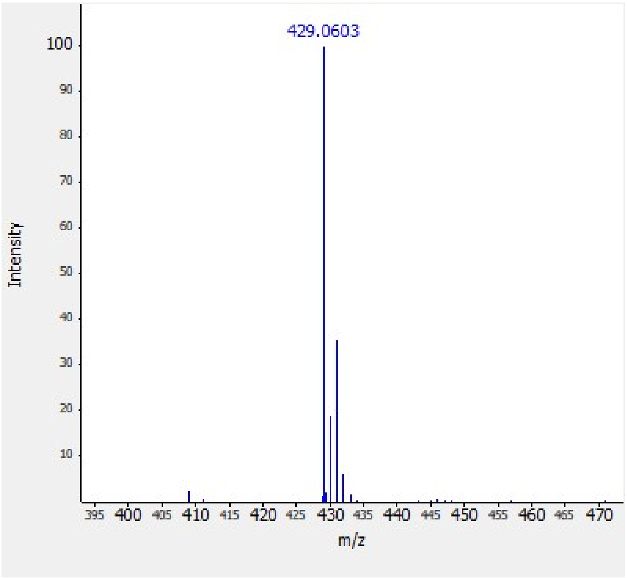
429.0603
100
90
80
70
60
50
40
30
20
10
Intensity
395
400
405
410
415
420
425
430
435
440
445
450
455
460
465
470
m/z

HRMS of **35**

Synthesis of BTK analog:

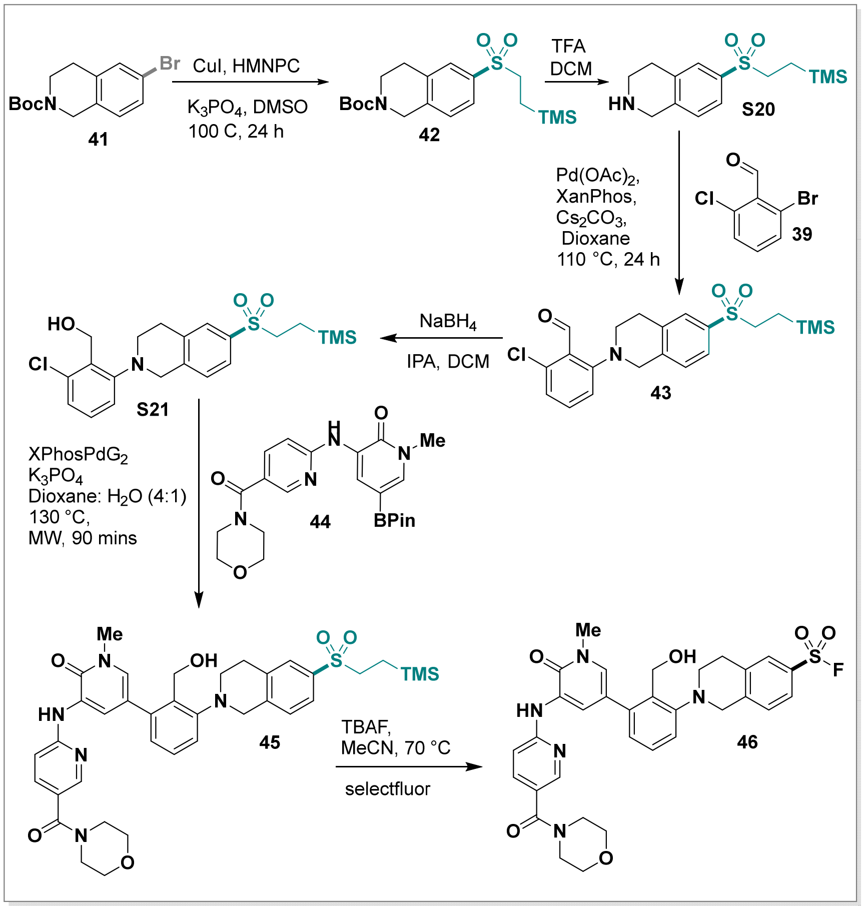

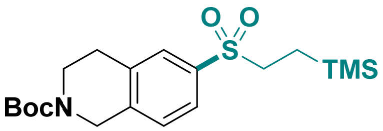

42

tert-butyl 6-((2-(trimethylsilyl)ethyl)sulfonyl)-3,4-dihydroisoquinoline-2(1_H_)-carboxylate (42)

A sealed microwave vial was charged with CuI (22 mg, 6 mol%), **L** (31 mg, 6 mol%), SES-Na (542.27 mg, 2.88 mmol,1.5 equiv), **41** (600 mg, 1.92 mmol), and K_3PO_4 (407.95 mg, 1.92 mmol,1 equiv). The vial was then evacuated and backfilled with argon and DMSO (5 mL) was then added into the tube via syringe. The reaction mixture was stirred at 100 °C in an oil bath for 24 h. After cooling to room temperature, the crude product was diluted with ethyl acetate and filtrated through silica gel. Then the filtrate was concentrated under reduced pressure. The residue was purified by flash chromatography (eluting with ethyl acetate/hexanes) to afford **42** as a white solid (660 mg, 82% yield).**^{1}H NMR** (400 MHz, $CDCl_3$) δ 7.69 (d, *J* = 8.1 Hz, 2H), 7.30 (d, *J* = 7.9 Hz, 1H), 4.66 (s, 2H), 3.69 (t, *J* = 5.9 Hz, 2H), 3.03 – 2.95 (m, 2H), 2.92 (t, *J* = 5.9 Hz, 2H), 1.50 (s, 9H), 1.00 – 0.83

(m, 2H), 0.01 (s, 9H); **^{13}C NMR** (101 MHz, $CDCl_3$) δ 154.6, 139.9, 136.8, 136.2, 128.6, 127.3, 125.9, 80.2, 77.4, 52.8, 45.5, 40.3, 29.0, 28.4, 9.1, -2.0; **HRMS** (ESI-TOF) m/z: $[M+Na]^+$ calc'd for $C_{19}H_{31}NNaO_4SSi$ 420.1641; found: 420.1635.

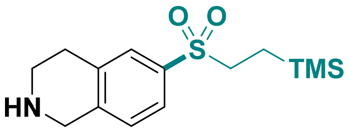

S20

6-((2-(trimethylsilyl)ethyl)sulfonyl)-1,2,3,4-tetrahydroisoquinoline (S20)

To the solution of **42** (40 mg, 0.1 mmol) in 0.5 mL CH_2Cl_2, was added 0.5 mL trifluoroacetic acid. The resulting mixture was stirred at room temperature for 5 h. After completion of reaction, the solvent was removed under reduced pressure. 1 M NaOH was added to the residue at 0 °C, and the resulting mixture was stirred for 30 min at room temperature. The product was extracted with CH_2Cl_2 and washed with brine. The organic extracts were dried over anhydrous Na_2SO_4, filtered, and concentrated under reduced pressure. The crude product was used in next step without further purification. **^{1}H NMR** (400 MHz, $CDCl_3$) δ 7.63 (d, *J* = 6.6 Hz, 2H), 7.20 (d, *J* = 8.4 Hz, 1H), 4.10 (s, 2H), 3.19 (d, *J* = 5.3 Hz, 2H), 3.07 – 2.94 (m, 2H), 2.90 (d, *J* = 10.6 Hz, 2H), 1.02 – 0.83 (m, 2H), 0.01 (s, 9H); **^{13}C NMR** (101 MHz, $CDCl_3$) δ 141.9, 136.1, 135.9, 128.7, 127.0, 125.0, 77.4, 52.4, 47.9, 43.0, 28.8, 8.9, -2.3; **HRMS** (ESI-TOF) m/z: $[M+H]^+$ calc'd for $C_{14}H_{24}NO_2SSi$ 298.1297; found: 298.1301.

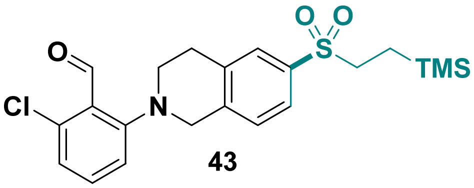

43

2-chloro-6-(6-((2-(trimethylsilyl)ethyl)sulfonyl)-3,4-dihydroisoquinolin-2(1*H*)-yl)benzaldehyde (43)

A sealed microwave vial was charged with **S20** (330 mg, 1.11 mmol) **39** (730.80 mg, 3.33 mmol, 3 equiv), $Pd(OAc)_2$ (22.43 mg, 9 mol%), XantPhos (64.23 mg, 10 mol%), and Cs_2CO_3 (506.32 mg, 1.55 mmol,1.4 equiv). The vial was then evacuated and backfilled with argon (X3), and degassed dioxane (2.8 mL) was then added into the vial via syringe. The reaction mixture was stirred at 110 °C in an oil bath for 24 h. After cooling to room temperature, the mixture was filtered while hot to remove excess salts. The filtrate was concentrated under reduced pressure, and the resulting residue was purified by flash chromatography (eluting with ethyl acetate/hexanes) to afford **42** as a yellow solid (305 mg, 63% yield).**^{1}H NMR** (400 MHz, $CDCl_3$) δ 10.40 (s, 1H), 7.72 (s, 1H), 7.69 (d, *J* = 8.0 Hz, 1H), 7.41 (t, *J* = 8.1 Hz, 1H), 7.28 (s, 1H), 7.10 (dd, *J* = 10.6, 8.1 Hz, 2H), 4.36 (s, 2H), 3.02 – 2.96 (m, 2H), 1.00 – 0.87 (m, 2H), 0.01 (s, 9H); **^{13}C NMR** (101 MHz, $CDCl_3$) δ 189.6, 154.6,

140.2, 138.1, 137.2, 136.1, 134.3, 128.9, 127.4, 125.9, 125.5, 124.4, 117.7, 54.6, 53.0, 52.1, 29.0, 9.3, -1.9; **HRMS** (ESI-TOF) m/z: $[M+H]^+$ calc'd for $C_{21}H_{27}ClNO_3SSi$ 436.1169; found: 436.1187.

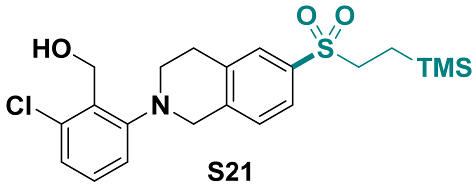

S21

(2-chloro-6-(6-((2-(trimethylsilyl)ethyl)sulfonyl)-3,4-dihydroisoquinolin-2(1*H*)-yl)phenyl)methanol (S21)

To the solution of **43** (300 mg, 0.69 mmol) in 1.5 mL CH_2Cl_2, was added 0.9 mL IPA. The solution was cooled to 5 °C; then $NaBH_4$ (106.15 mg, 2.81 mmol, 4 equiv) was added portionwise over 1 h. Upon completion of the reduction as indicated by TLC, the reaction was quenched with 10% aqueous ammonium chloride solution. The product was extracted with $CH_2Cl_2$and washed with brine. The organic extracts were dried over anhydrous Na_2SO_4, filtered, and concentrated under reduced pressure. The residue was purified by flash chromatography (eluting with ethyl acetate/hexanes) to afford **S21** as a light yellow solid (266 mg, 88% yield). **^{1}H NMR** (400 MHz, $CDCl_3$) δ 7.69 (s, 1H), 7.66 (d, *J* = 8.0 Hz, 1H), 7.24 (s, 1H), 7.23 – 7.16 (m, 2H), 7.10 (dd, *J* = 7.5, 1.8 Hz, 1H), 4.94 (s, 2H), 4.22 (s, 2H), 3.35 (t, *J* = 5.8 Hz, 2H), 3.11 (t, *J* = 5.9 Hz, 2H), 3.05 – 2.91 (m, 2H), 1.02 – 0.80 (m, 2H), -0.00 (s, 9H); **^{13}C NMR** (101 MHz, $CDCl_3$) δ 152.6, 140.6, 137.1, 135.5, 135.0, 133.4, 129.4, 128.9, 127.5, 126.1, 125.7, 119.5, 77.4, 60.3, 55.2, 52.9, 50.8, 29.5, 9.2, -1.9; **HRMS** (ESI-TOF) m/z: $[M+H]^+$ calc'd for $C_{21}H_{29}ClNO_3SSi$ 438.1326; found: 438.1328.

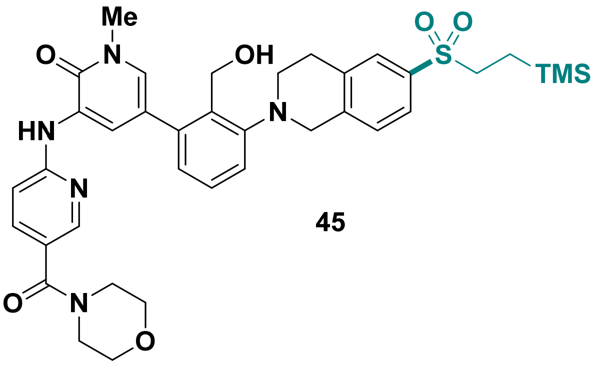

45

5-(2-(hydroxymethyl)-3-(6-((2-(trimethylsilyl)ethyl)sulfonyl)-3,4 dihydroisoquinolin-2(1*H*)-yl)phenyl)-1-methyl-3-((5-(morpholine-4-carbonyl)pyridin-2-yl)amino)pyridin-2(1*H*)-one (45)

44 was synthesized using the known synthetic procedure.[13] A sealed microwave vial was charged with **S21** (396 mg,0.91 mmol), **44** (800 mg, 1.81 mmol, 2 equiv), $XPhosPdG_2$ (36 mg, 5 mol%), and K_3PO_4 (384.21 mg, 1.81 mmol,2 equiv). The vial was then evacuated and backfilled with argon

(X3) and degassed dioxane: H_2O (3 mL, 4:1) was then added into the vial via syringe. The reaction mixture was microwaved at 130 °C for 90 minutes. After cooling to room temperature, the mixture was filtered hot to remove excess salts. The filtrate was concentrated under reduced pressure and the resulting residue was purified by flash chromatography to afford **45** as a yellow solid (370 mg, 57% yield).^{1}**H NMR** (400 MHz, $CDCl_3$) δ 8.61 (d, *J* = 2.3 Hz, 1H), 8.32 (d, *J* = 2.3 Hz, 1H), 8.07 (s, 1H), 7.74 (s, 1H), 7.73 – 7.68 (m, 1H), 7.64 (dd, *J* = 8.6, 2.3 Hz, 1H), 7.38 (t, *J* = 7.8 Hz, 1H), 7.32 – 7.27 (m, 2H), 7.16 (dd, *J* = 7.6, 1.2 Hz, 1H), 6.93 (d, *J* = 2.3 Hz, 1H), 6.82 (d, *J* = 8.6 Hz, 1H), 4.82 (s, 2H), 4.35 (s, 2H), 3.67 (m, 11H), 3.46 (t, *J* = 5.7 Hz, 2H), 3.18 (t, *J* = 5.8 Hz, 2H), 3.08 – 2.94 (m, 2H), 1.09 – 0.86 (m, 2H), 0.03 (s, 9H); ^{13}**C NMR** (101 MHz, $CDCl_3$) δ 168.5, 157.4, 155.6, 152.1, 147.3, 140.9, 139.6, 137.0, 135.6, 134.4, 129.9, 128.9, 128.7, 127.5, 127.3, 126.7, 125.7, 122.4, 120.6, 120.6, 120.3, 111.7, 66.9, 60.6, 55.5, 52.9, 50.9, 38.1, 29.8, 9.2, -1.9; **HRMS** (ESI-TOF) m/z: $[M+H]^+$ calc'd for $C_{37}H_{46}N_5O_6SSi$ 716.2938; found: 716.2938.

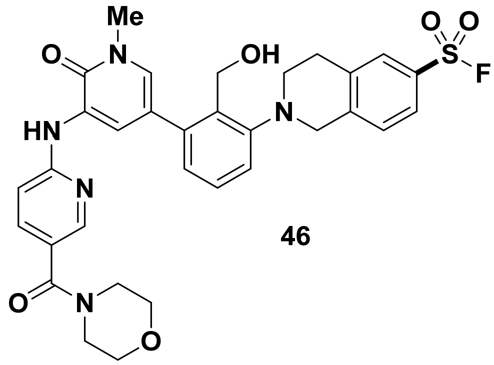

46

2-(2-(hydroxymethyl)-3-(1-methyl-5-((5-(morpholine-4-carbonyl)pyridin-2-yl)amino)-6-oxo-1,6-dihydropyridin-3-yl)phenyl)-1,2,3,4-tetrahydroisoquinoline-6-sulfonyl fluoride (46)

A vial was charged with **45** (0.13 mmol) and TBAF (1.26 mL, 1.26 mmol, 10 equiv). The vial was then evacuated and backfilled with argon (X3), and degassed MeCN (2 mL) was then added into the vial via syringe. The reaction was stirred at 80 °C for 16 h, until the disappearance of starting material (progress monitored by TLC). The reaction was allowed to warm to room temperature and then selectfluor (178.55 mg, 0.50 mmol, 4 equiv) was added. The mixture was stirred at room temperature for 2 h. Upon completion, the reaction mixture was diluted with water and the product was extracted with ethyl acetate. The organic extracts were dried over anhydrous Na_2SO_4, filtered, and concentrated under reduced pressure. The crude product was purified by high-performance liquid chromatography with a C18 column (Agilent 50 mm x 150 mm) and a gradient of acetonitrile/water to obtain **46** as a light yellow solid (42 mg, 53% yield) ^{1}**H NMR** (400 MHz, $CDCl_3$) δ 8.61 (d, *J* = 2.3 Hz, 1H), 8.32 (d, *J* = 2.3 Hz, 1H), 8.07 (s, 1H), 7.86 (s, 1H), 7.82 (d, *J* = 8.1 Hz, 1H), 7.63 (dd, *J* = 8.6, 2.3 Hz, 1H), 7.42 – 7.33 (m, 2H), 7.26 (m, 1H), 7.16 (dd, *J* = 7.6, 1.2 Hz, 1H), 6.96 (d, *J* = 2.2 Hz, 1H), 6.82 (d, *J* = 8.5 Hz, 1H), 4.79 (s, 2H), 4.39 (s, 2H), 3.70 (m, 11H), 3.48 (t, *J* = 5.8 Hz, 2H), 3.19 (t, *J* = 5.9 Hz, 2H); ^{13}**C NMR** (101 MHz, $CDCl_3$) δ 168.5, 157.5, 155.6, 152.1, 147.4, 143.6, 139.9, 137.2, 136.5, 134.6, 129.9, 129.3, 128.9, 128.0, 127.3, 126.7, 125.9,

122.5, 120.8, 120.6, 120.5, 111.8, 67.0, 60.4, 55.7, 50.8, 45.9, 38.3, 29.83, 29.77; **^{19}F NMR** (376 MHz, $CDCl_3$) δ 66.3 **HRMS** (ESI-TOF) m/z: $[M+H]^+$ calc'd for $C_{32}H_{33}FN_5O_6S$ 634.2136; found: 634.2140.

Inhibition Kinetics Characterization

The BTK enzyme system and the Kinase-Glo kinase assay were purchased from SinoBiological and Promega Corporation (V3771) respectively. In each kinase reaction, the concentration of BTK was set to 4 ng/μL. The peptide substrate Poly (4:1 Glu, Tyr) and ATP concentrations were set to 0.2 mg/mL and 2 µM, respectively. BTK was preincubated with different inhibitor concentrations (2-fold serial dilutions, starting at 800 µM) over different time periods (5–60 min), before initiating the kinase reactions. Reactions were started by adding a 2× Poly E4Y1/ATP mixture. The reactions were carried out in a 96-well plate and quenched simultaneously with the addition of 50 µL of the Kinase-Glo Plus reagent and incubated for 15 min to produce a luminescence signal. The signal was measured using BioTek Synergy HTX multi-mode Microplate Reader with an integration time of 0.250 s. The experimental values were fitted into a sigmoidal dose-response curves using GraphPad Prism to obtain IC_{50} values.

Spectra:

^1H NMR ($CDCl_3$, 400 MHz)

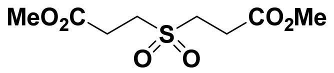

S2

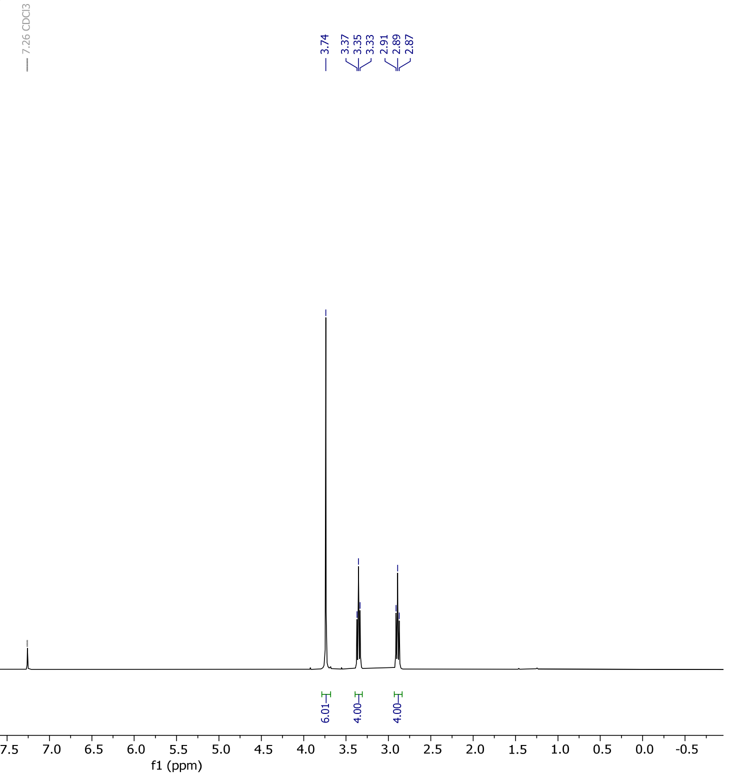

^{13}C NMR ($CDCl_3$; 101 MHz)

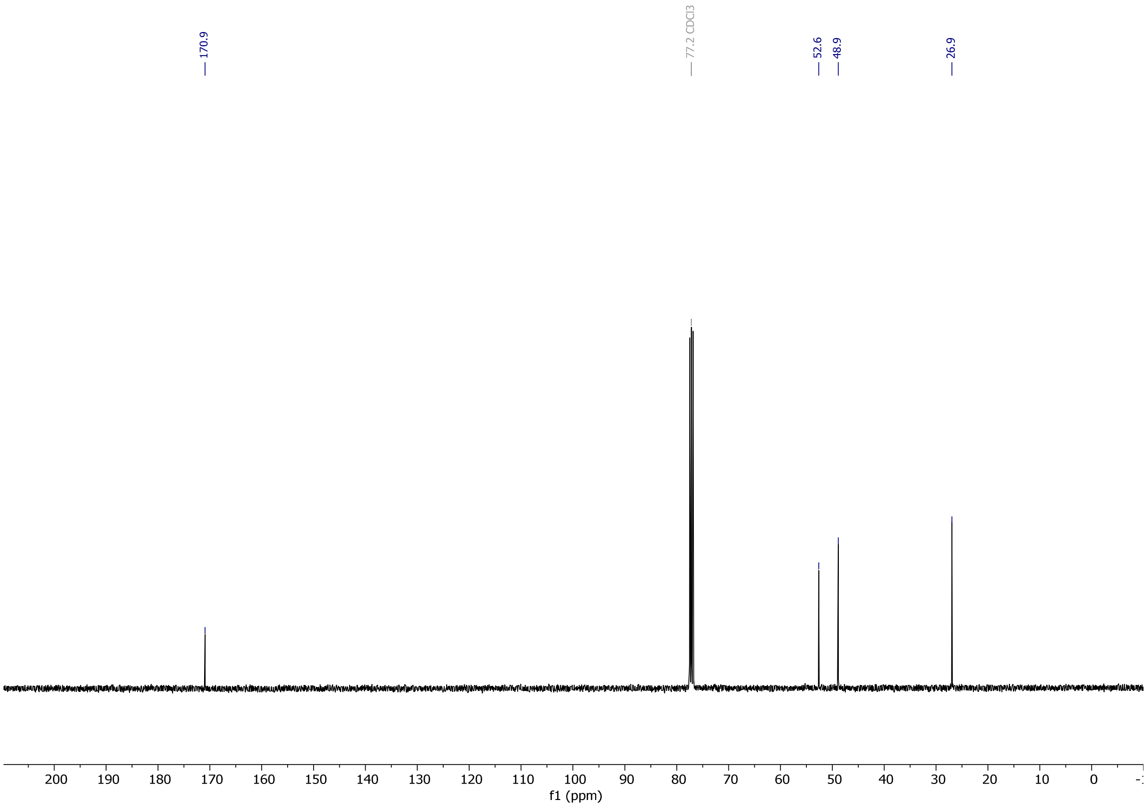

^{1}H NMR ($CDCl_3$, 400 MHz)

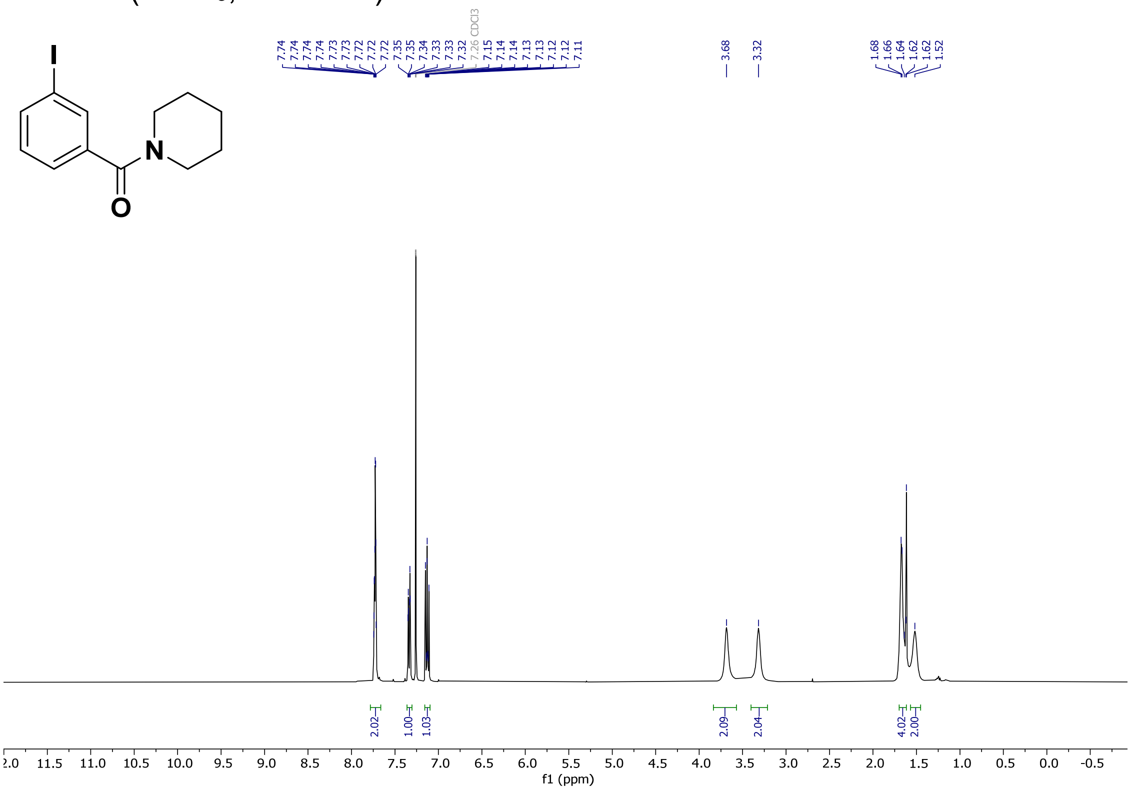

^{13}C NMR ($CDCl_3$; 101 MHz)

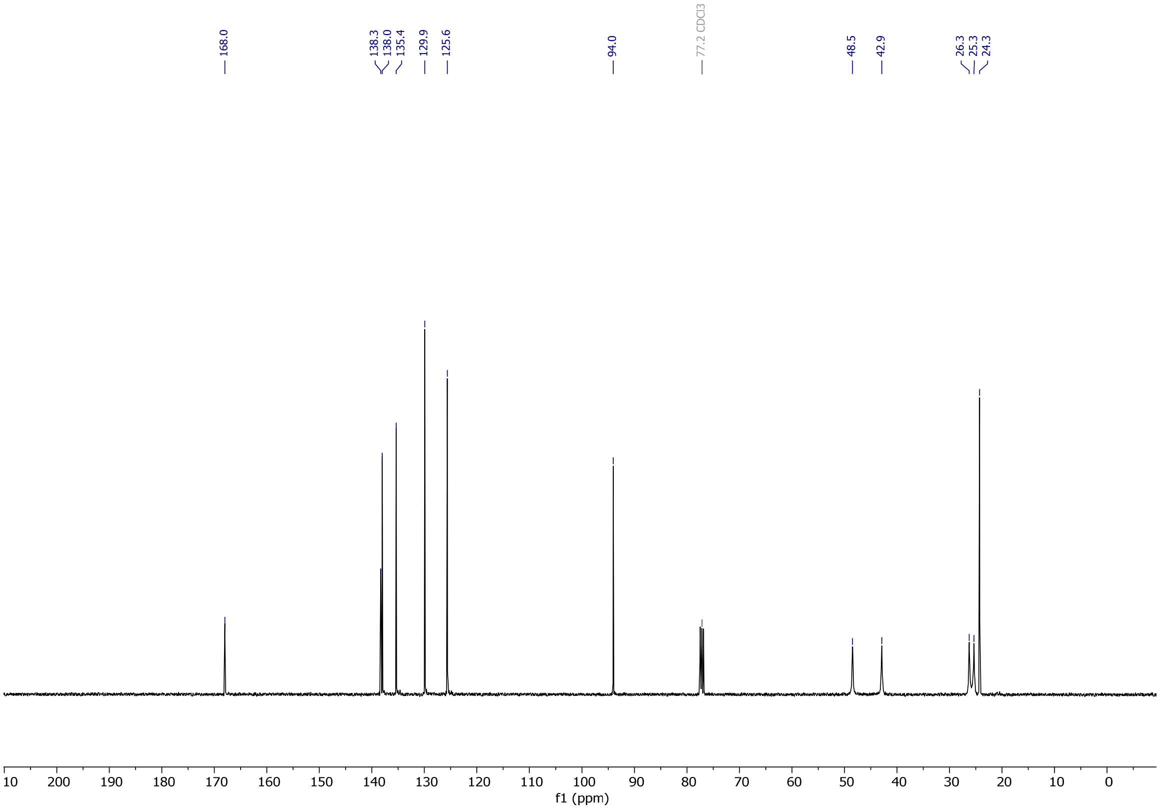

^{1}H NMR ($CDCl_3$, 400 MHz)

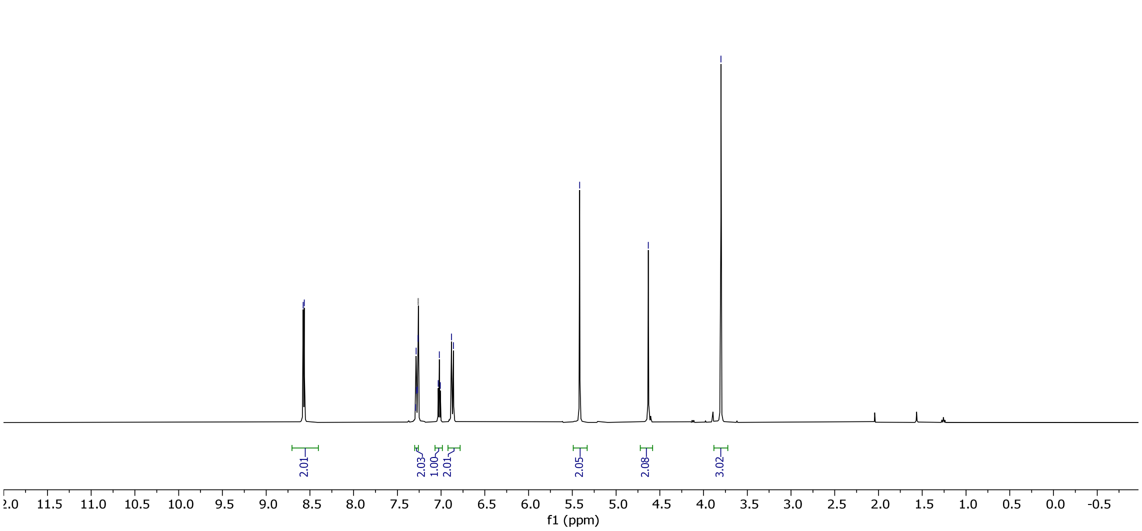

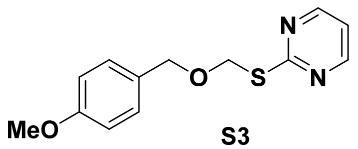

S3

^{13}C NMR ($CDCl_3$; 101 MHz)

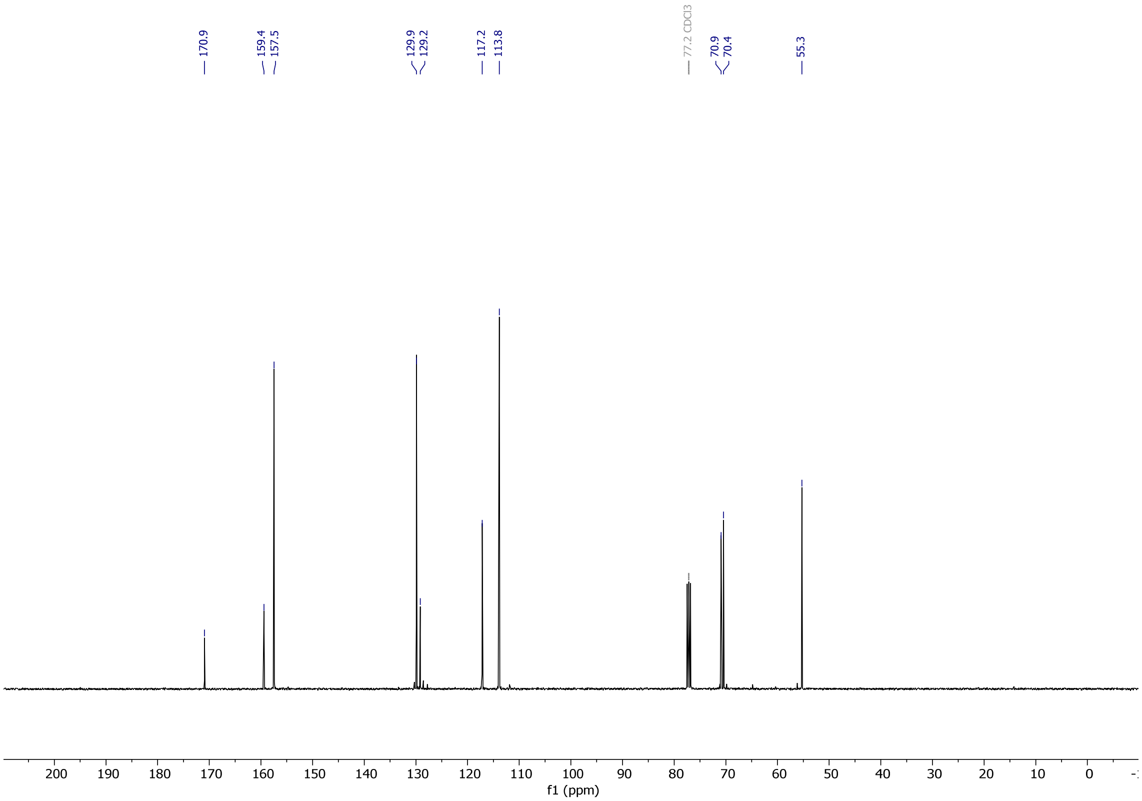

^{1}H NMR ($CDCl_3$, 400 MHz)

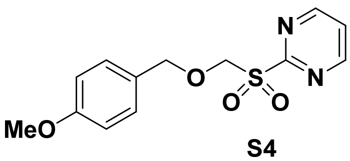

S4

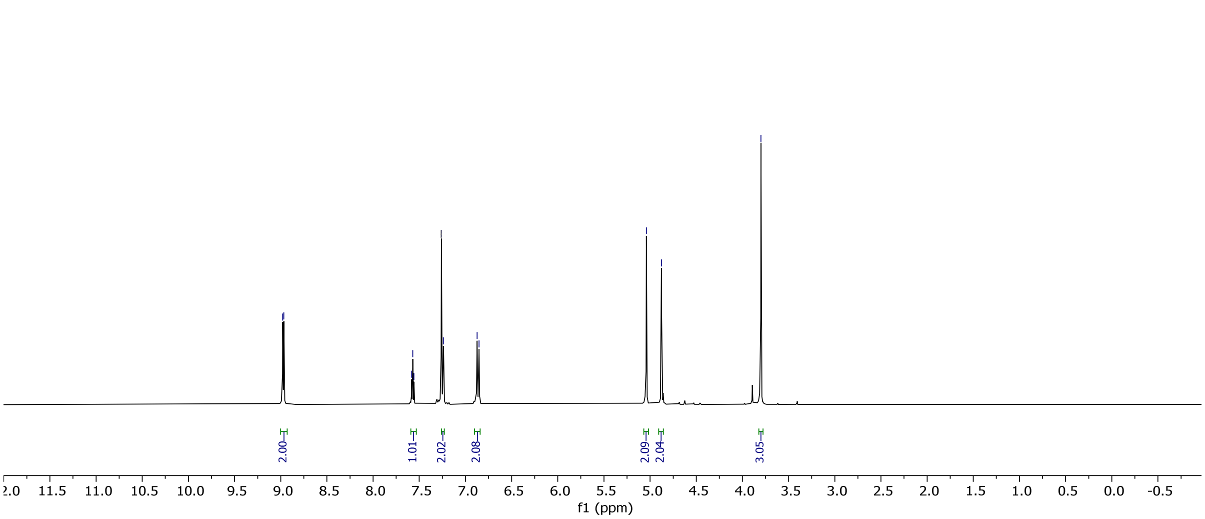

^{13}C NMR ($CDCl_3$; 101 MHz)

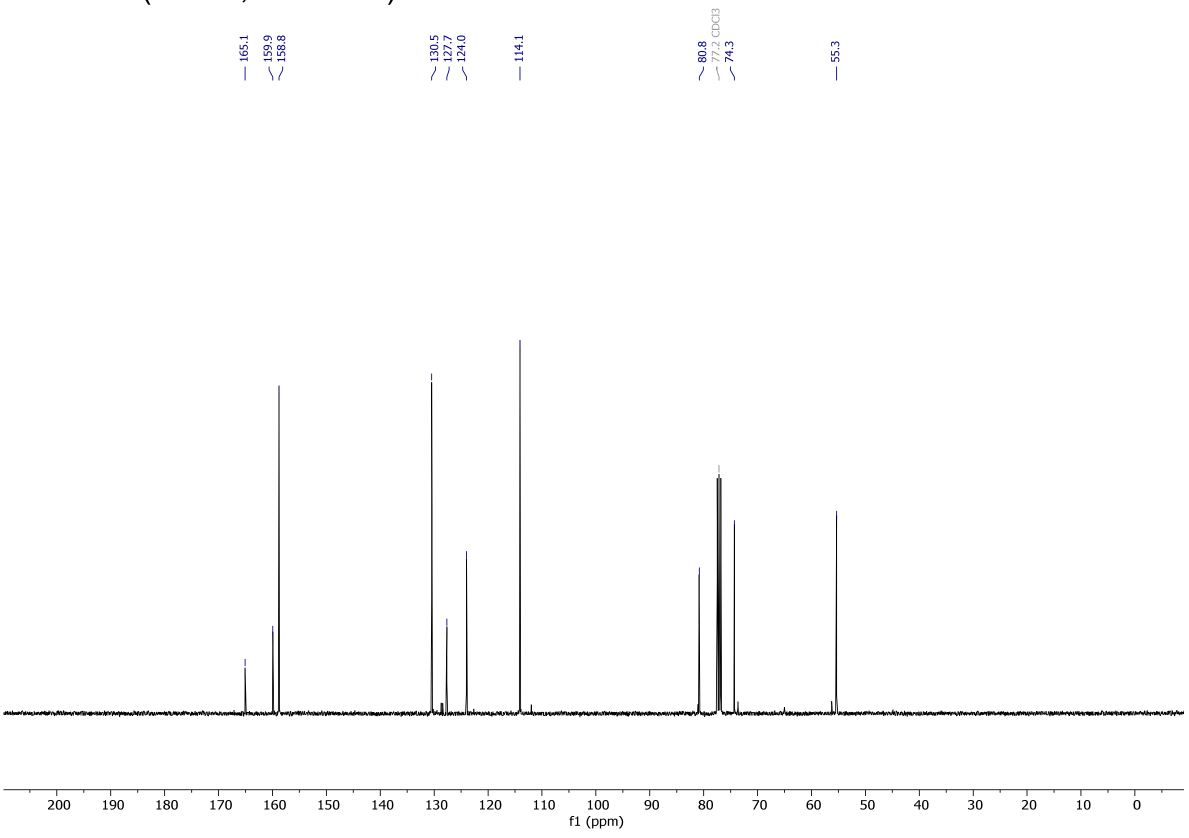

^{1}H NMR (DMSO-d_6, 400 MHz)

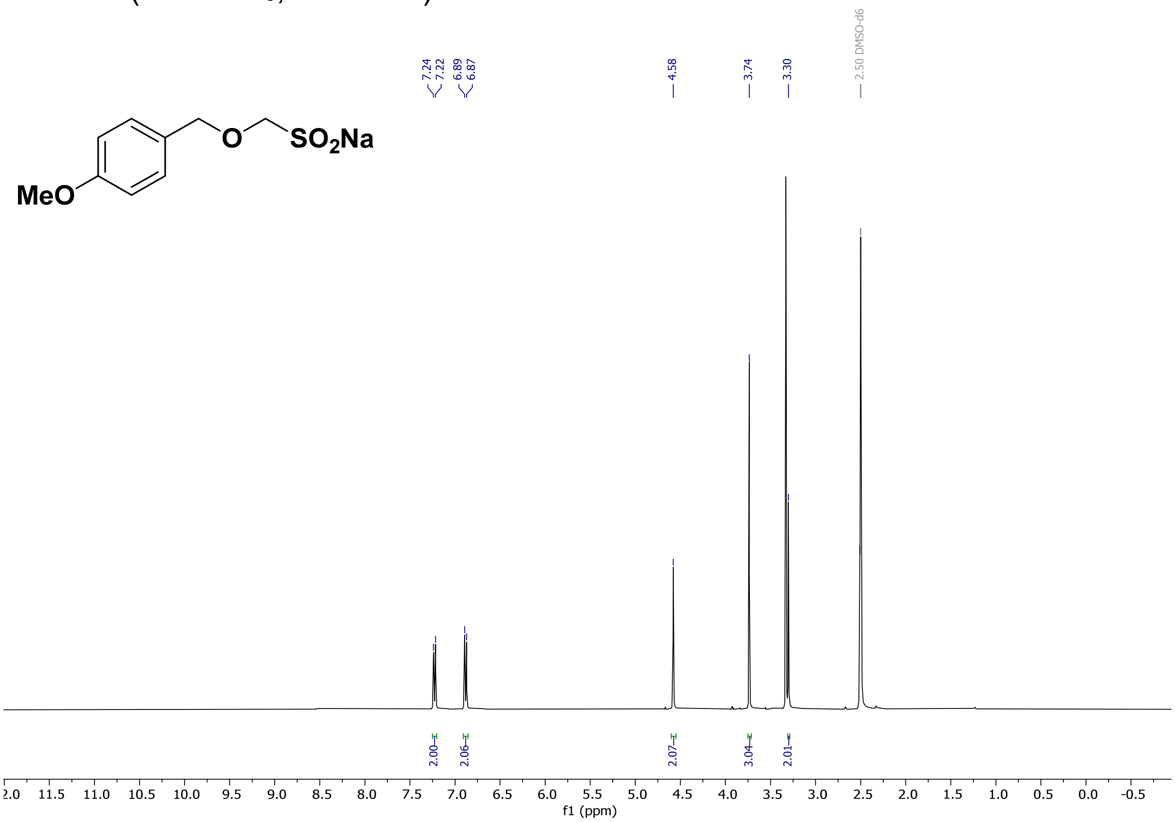

^{13}C NMR (DMSO-d_6; 101 MHz)

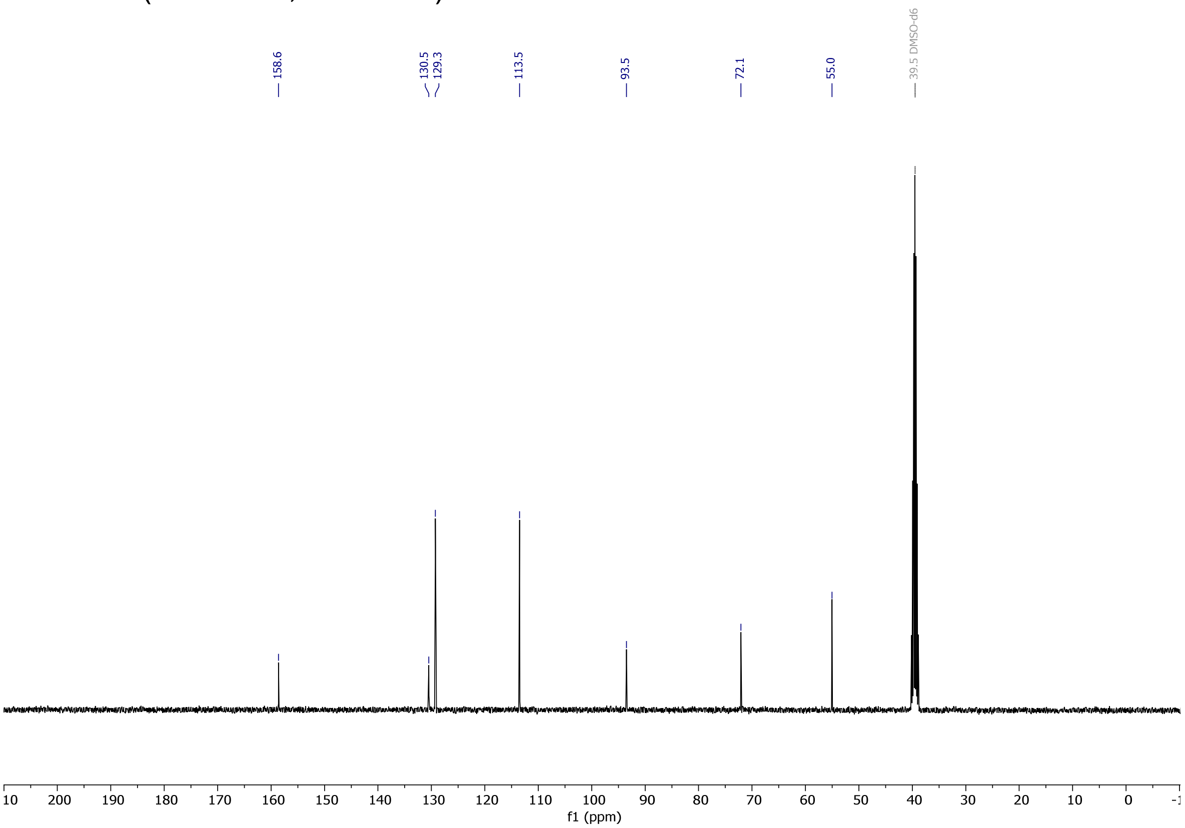

^{1}H NMR ($CDCl_3$, 400 MHz)

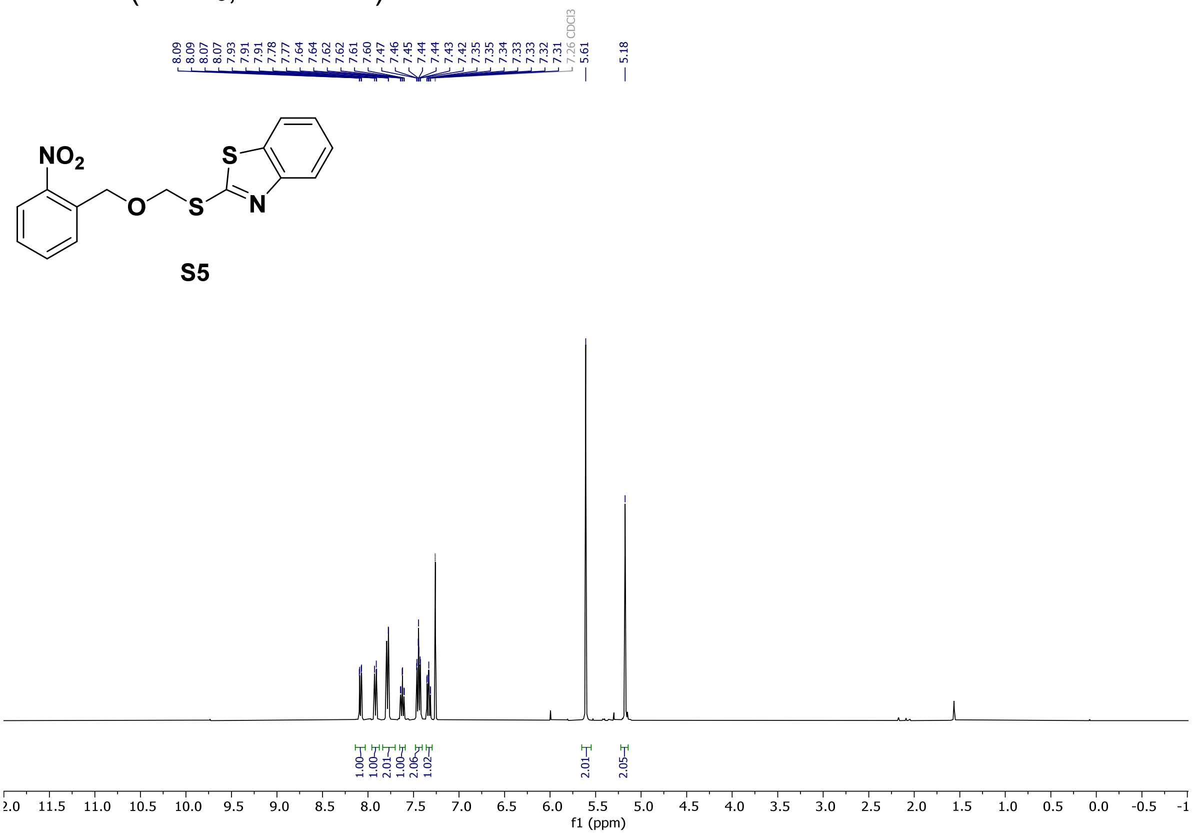

^{13}C NMR ($CDCl_3$; 101 MHz)

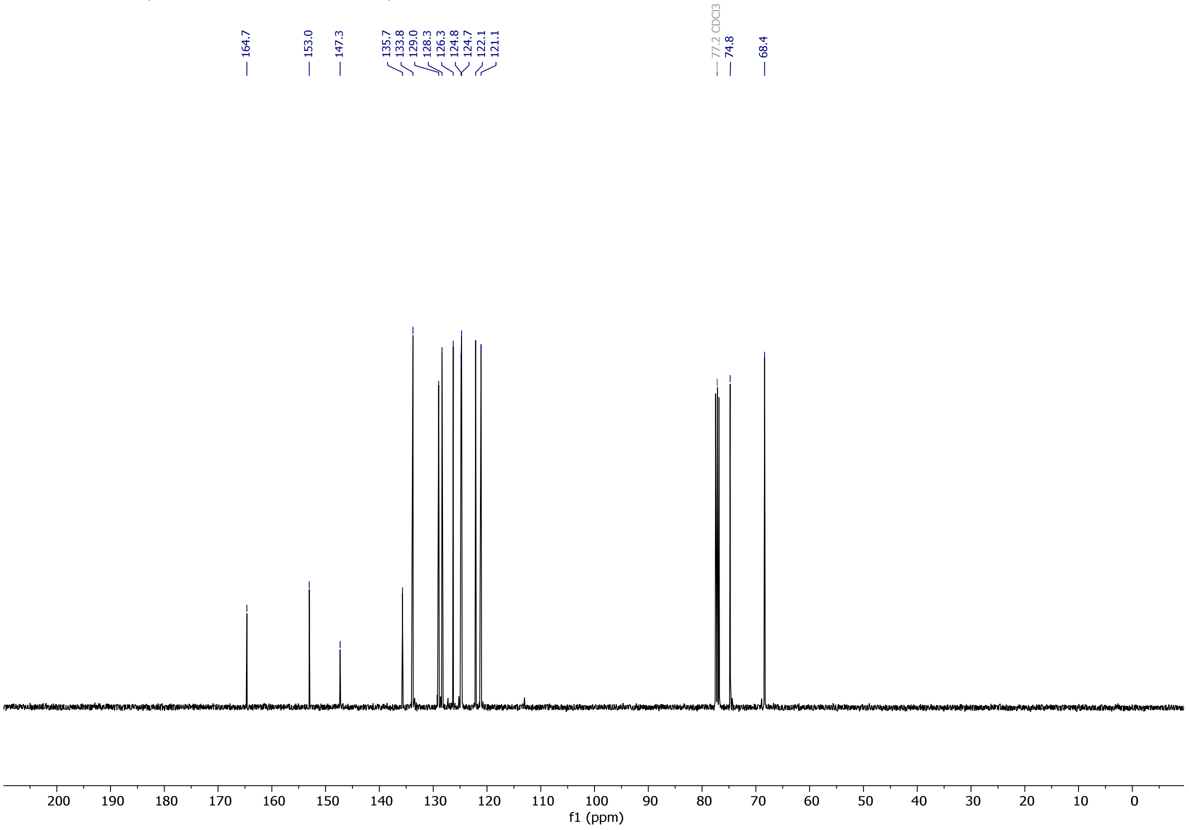

^{1}H NMR ($CDCl_3$, 400 MHz)

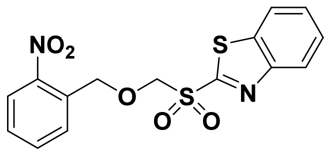

S6

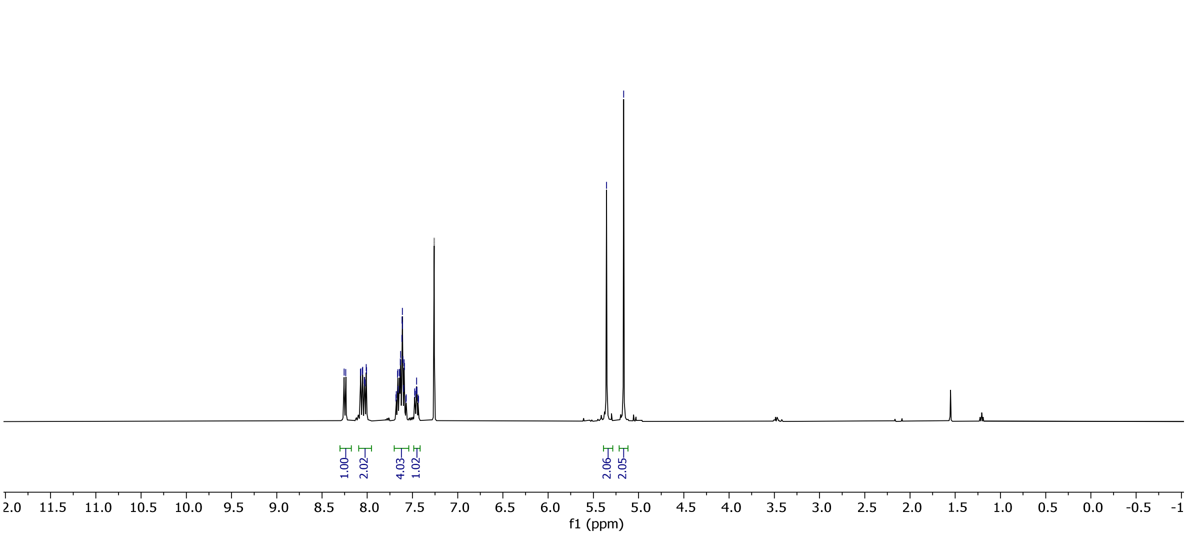

^{13}C NMR ($CDCl_3$; 101 MHz)

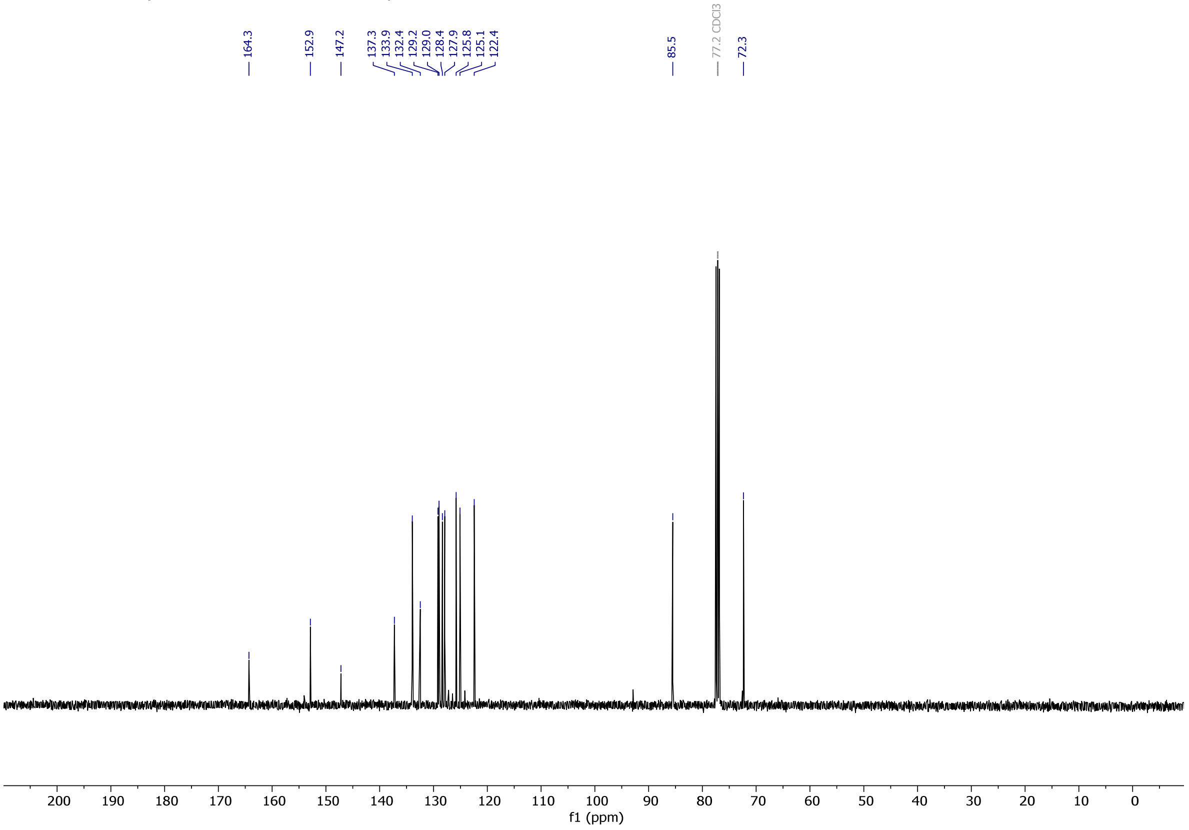

^{1}H NMR (DMSO-d_6, 400 MHz)

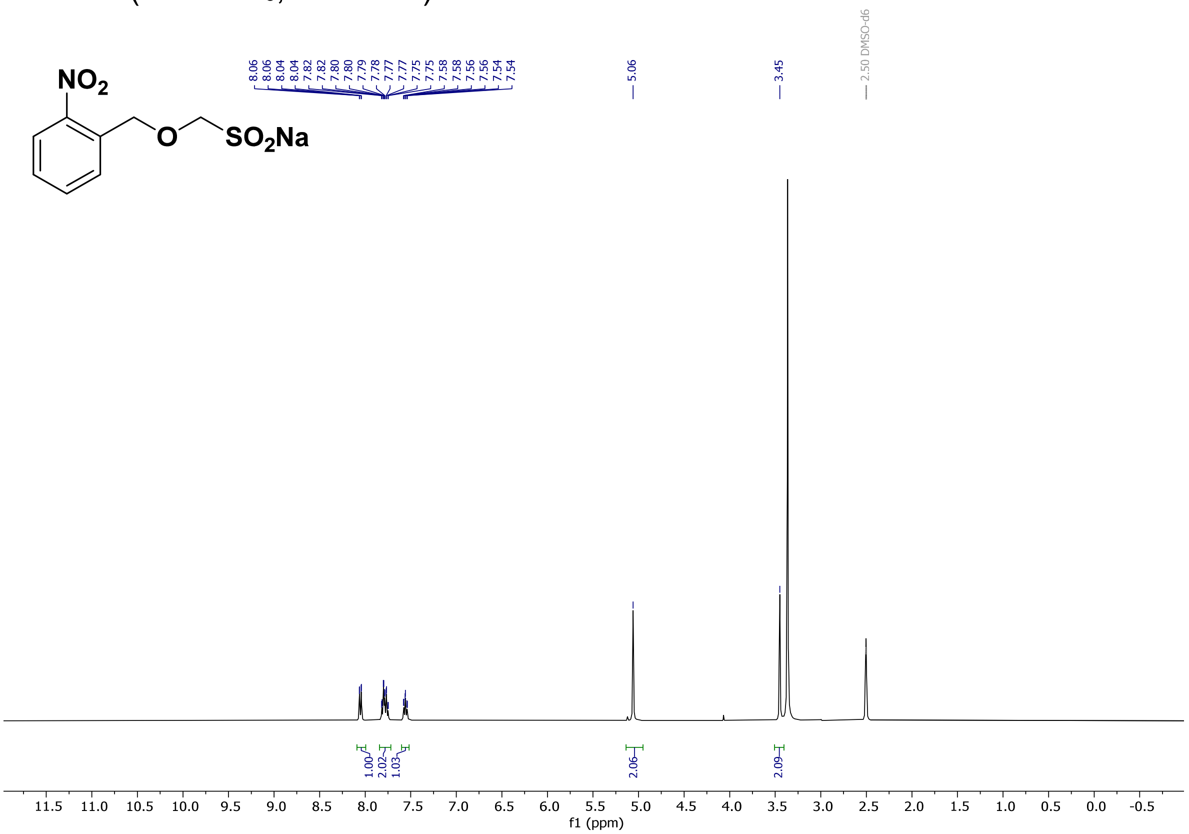

^{13}C NMR (DMSO-d_6; 101 MHz)

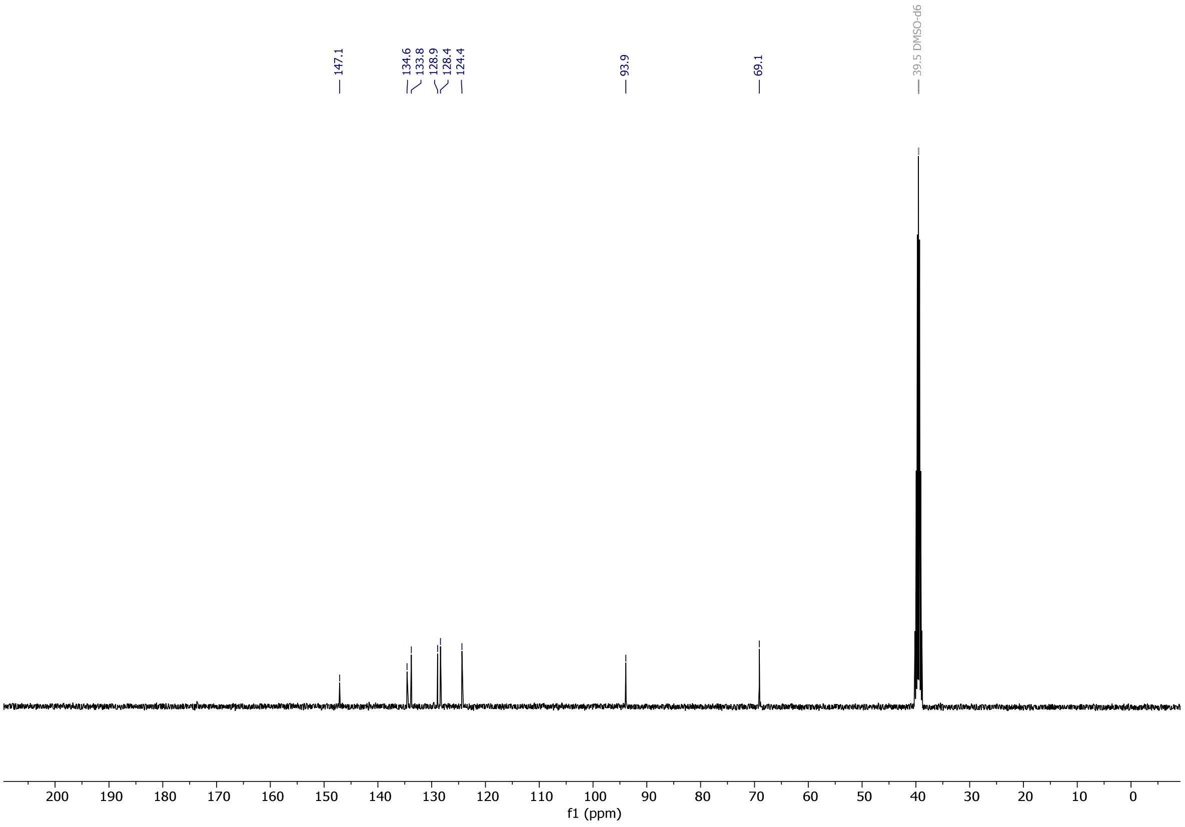

^{1}H NMR ($CDCl_3$, 400 MHz)

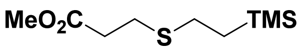

S7

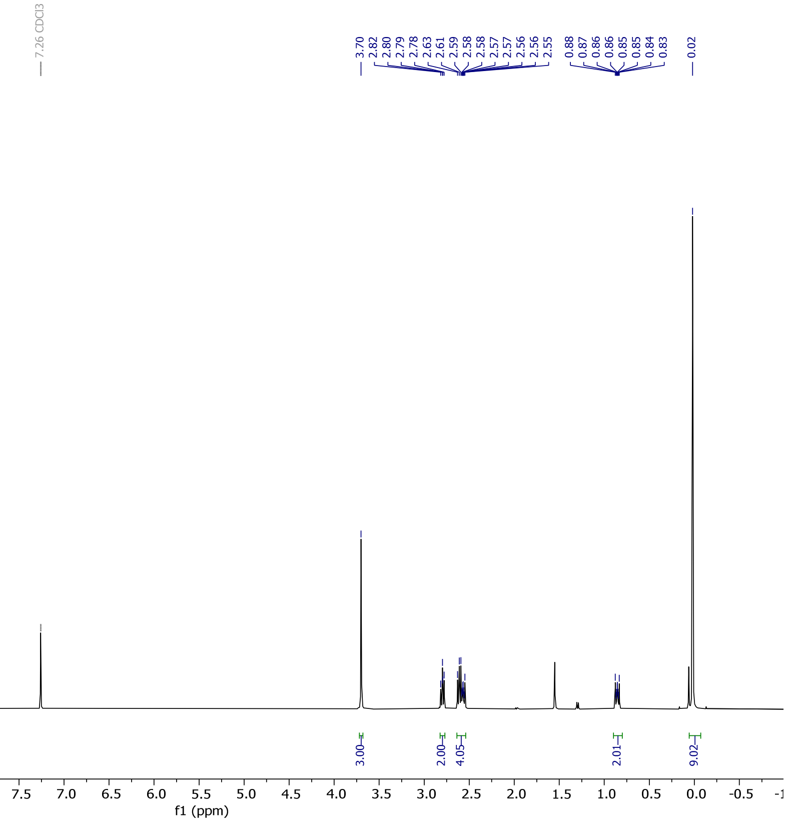

^{13}C NMR ($CDCl_3$; 101 MHz)

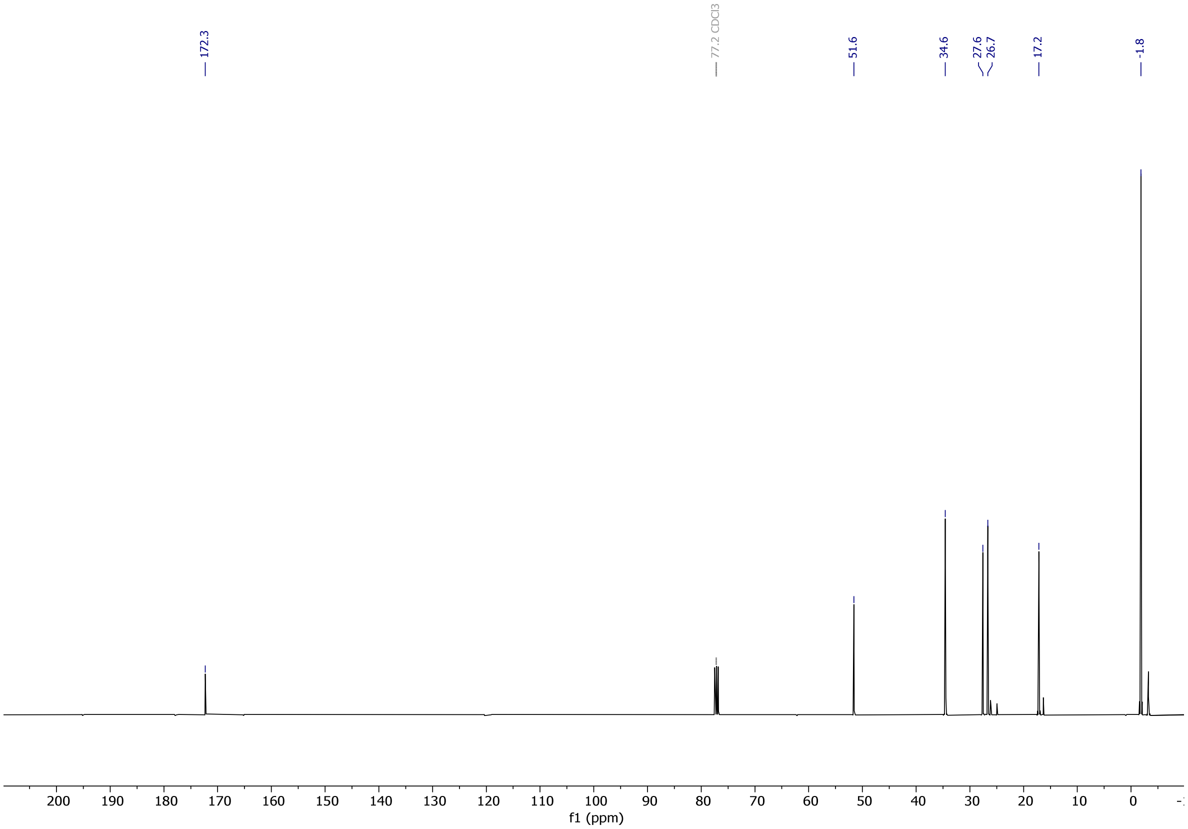

^{1}H NMR ($CDCl_3$, 400 MHz)

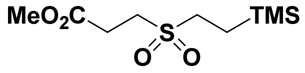

S8

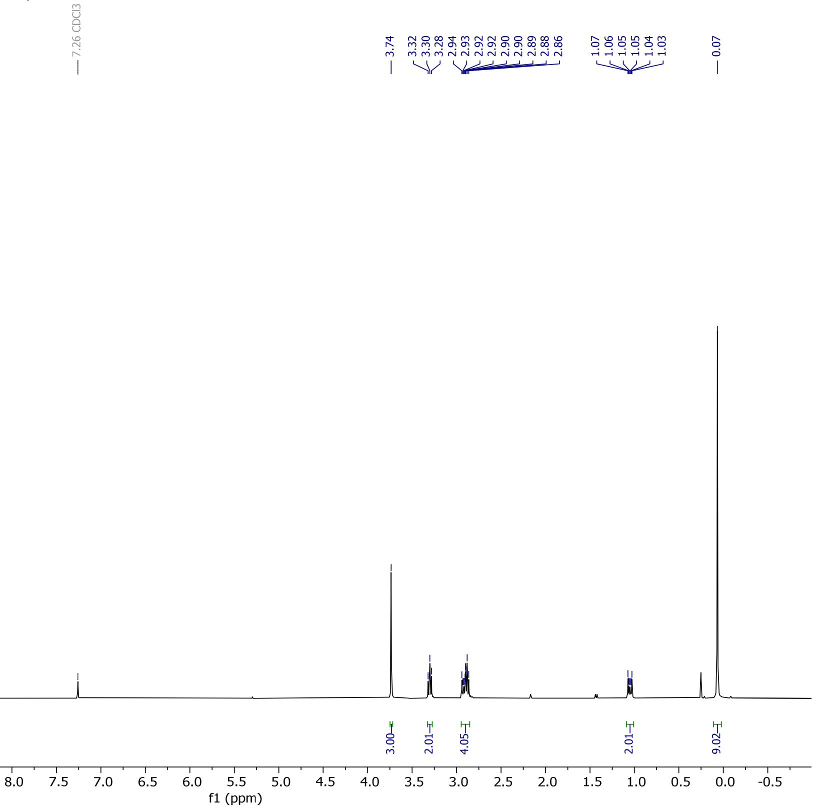

^{13}C NMR ($CDCl_3$; 101 MHz)

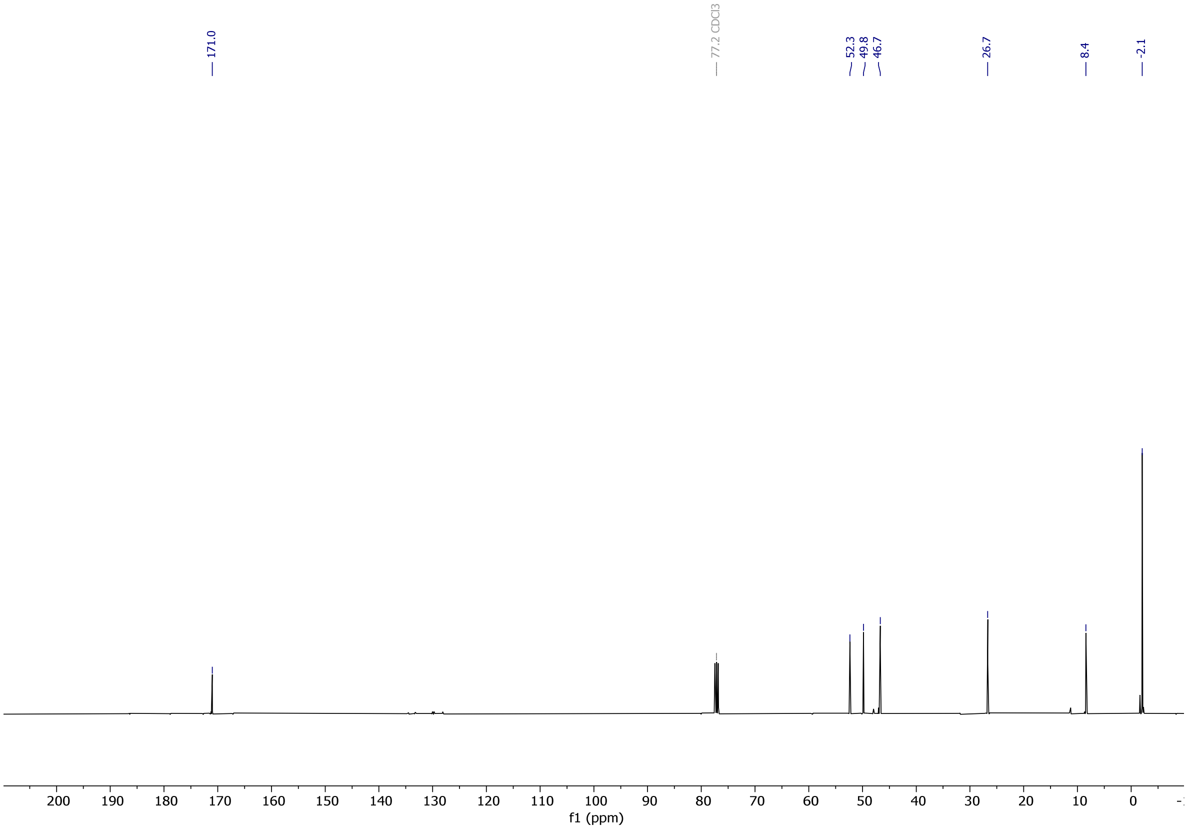

^{1}H NMR (MeOD-d_4, 400 MHz)

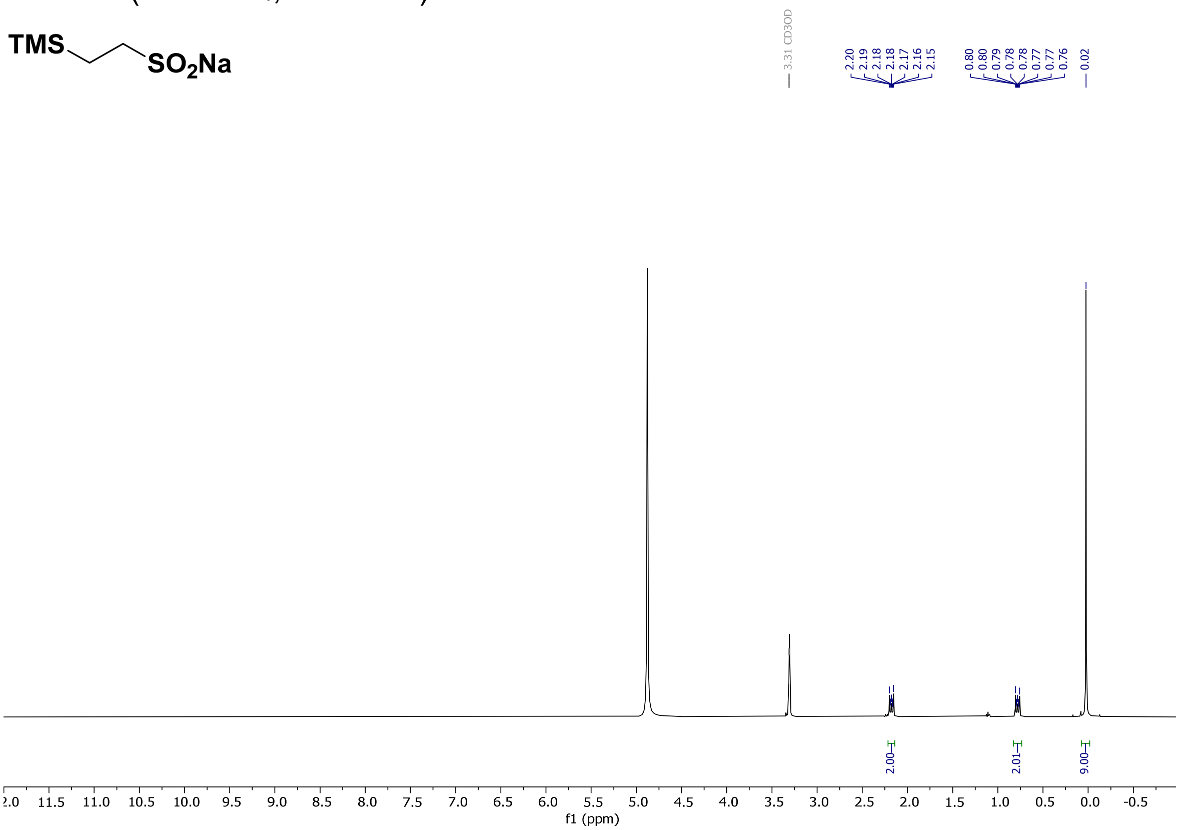

^{13}C NMR (MeOD-d_4; 101 MHz)

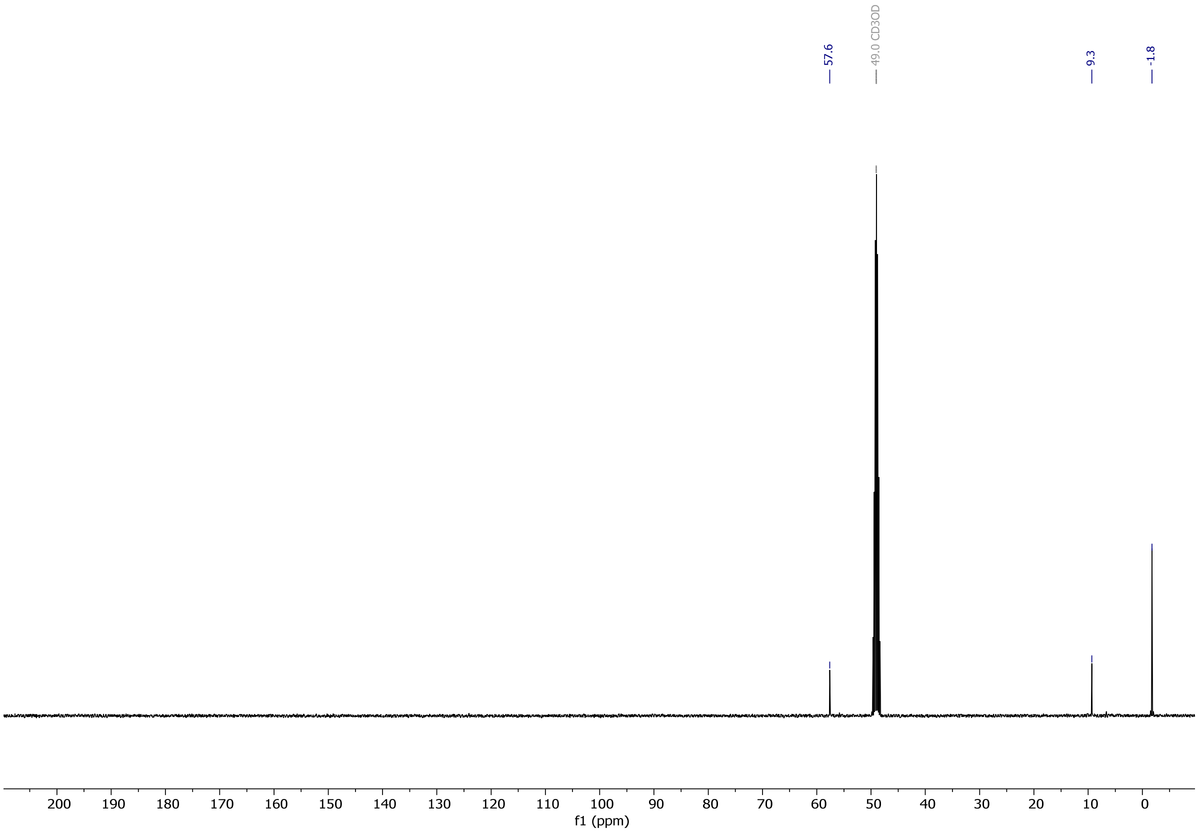

^{1}H NMR ($CDCl_3$, 400 MHz)

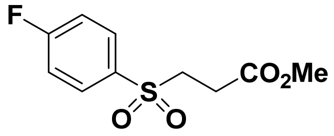

1

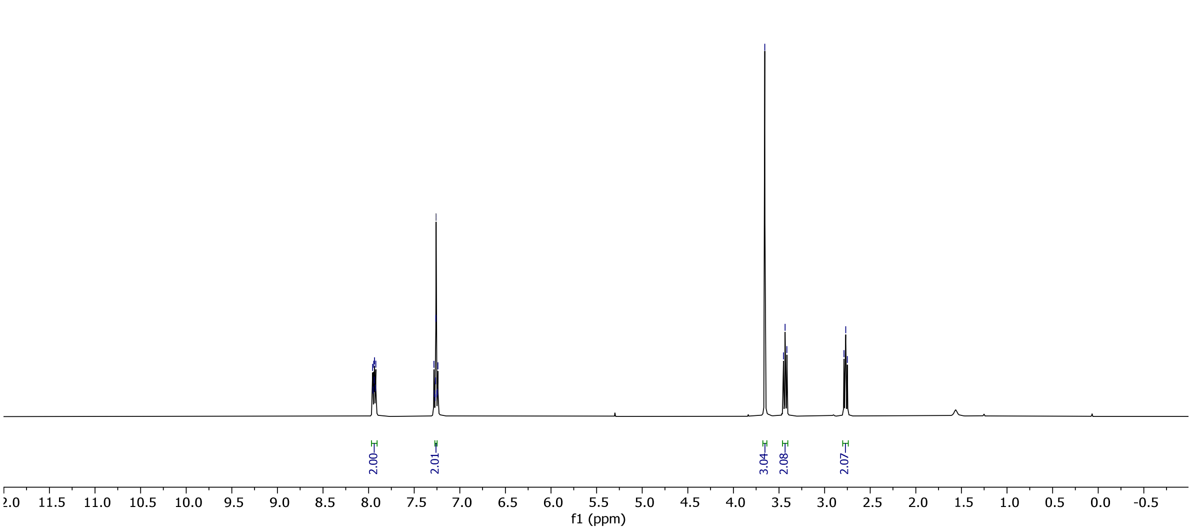

^{13}C NMR ($CDCl_3$; 101 MHz)

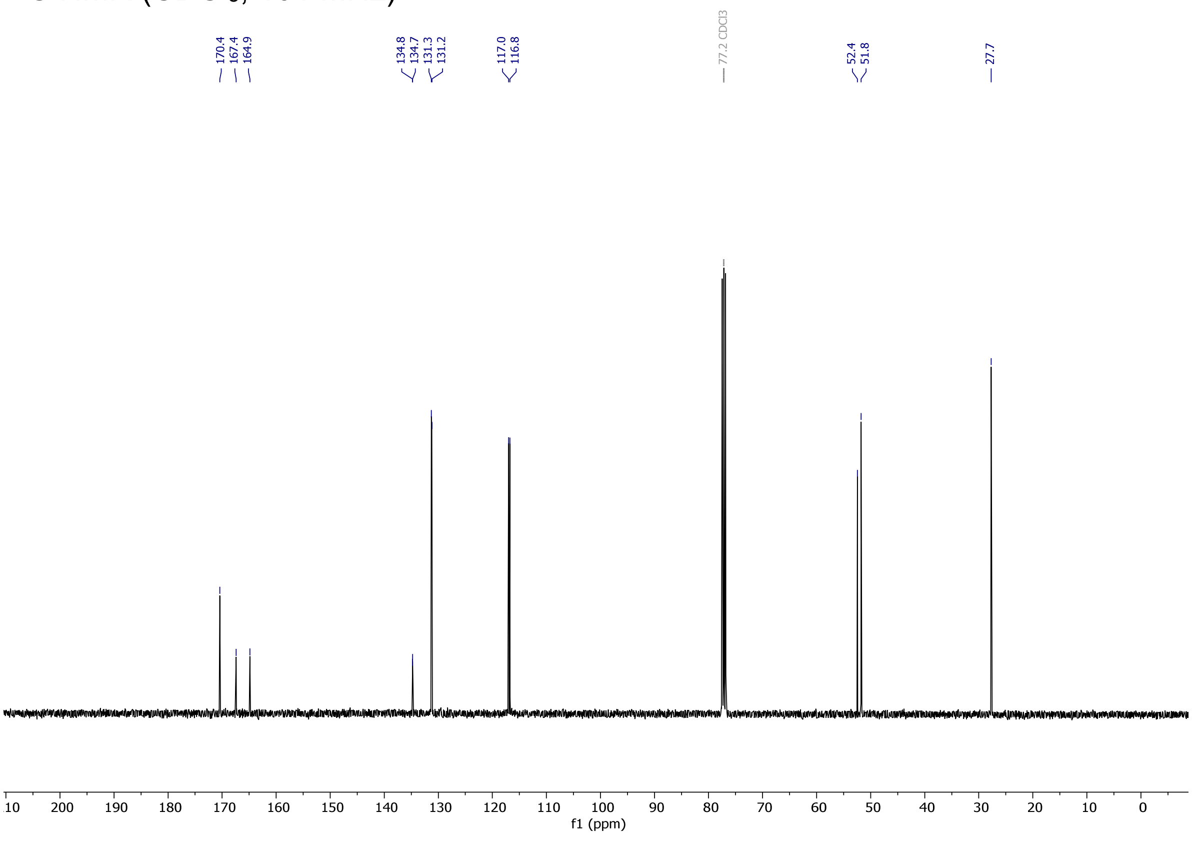

^{19}F NMR ($CDCl_3$; 376 MHz)

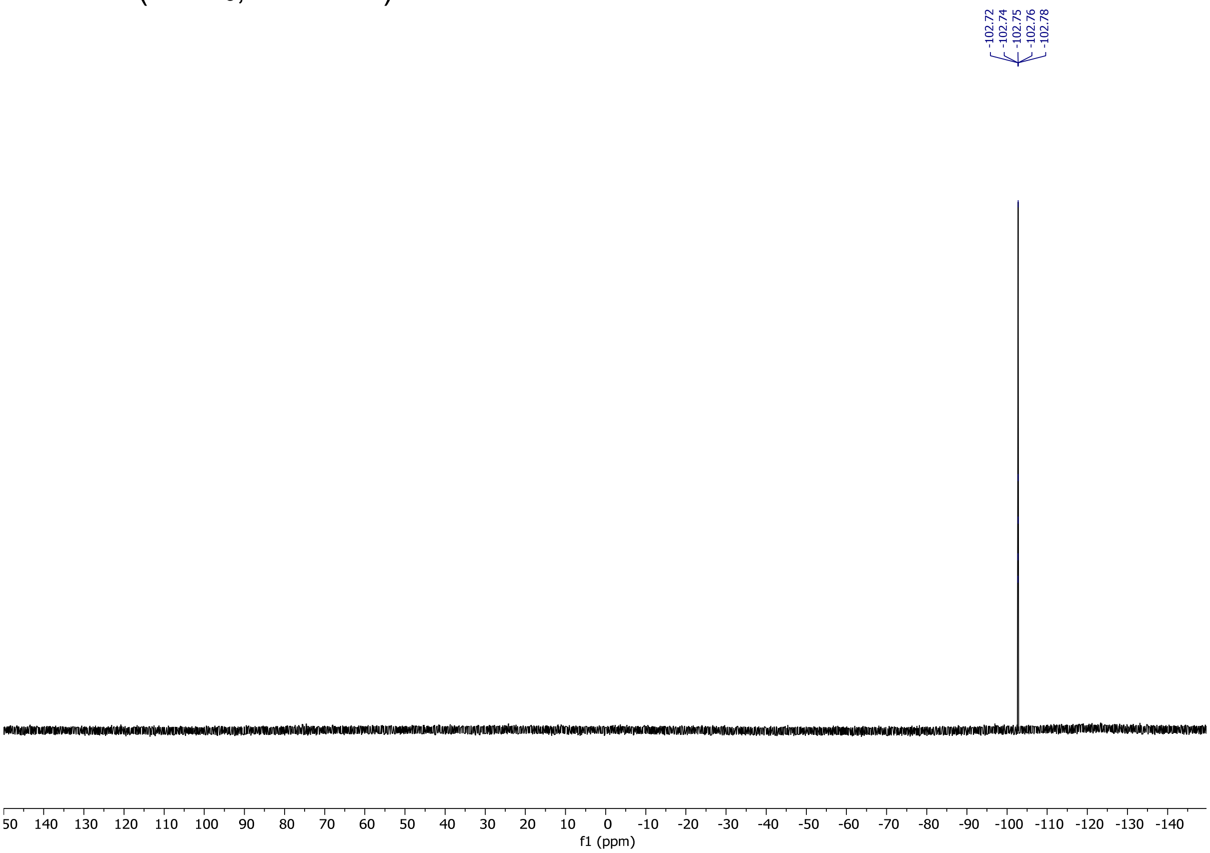

^{1}H NMR ($CDCl_3$, 400 MHz)

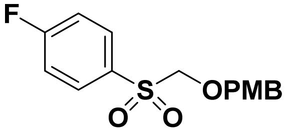

4

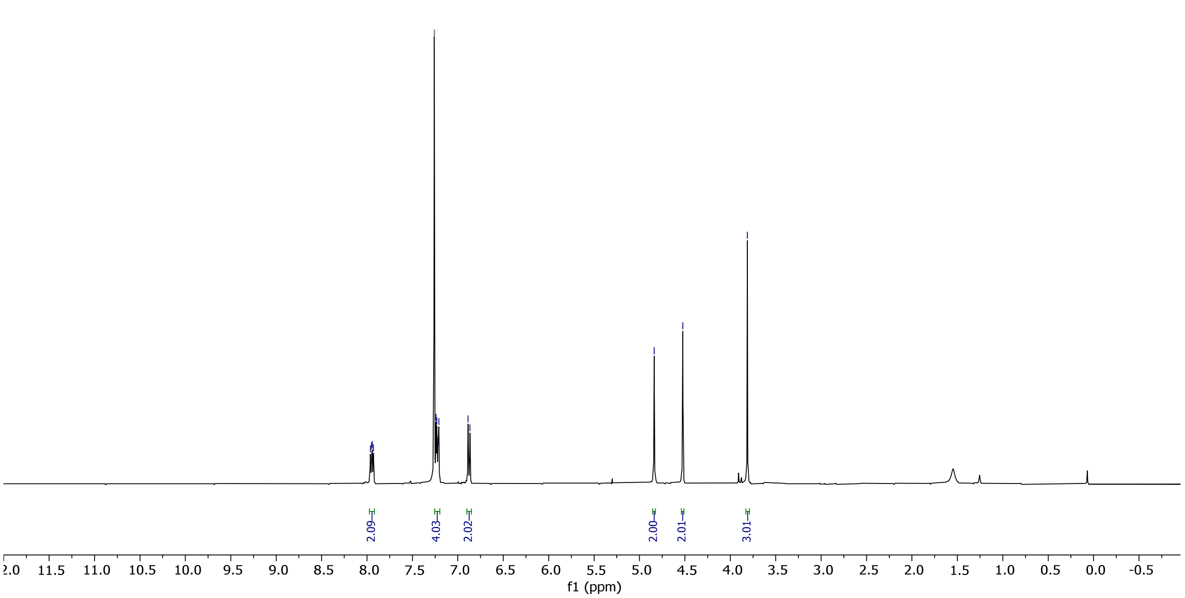

^{13}C NMR ($CDCl_3$; 101 MHz)

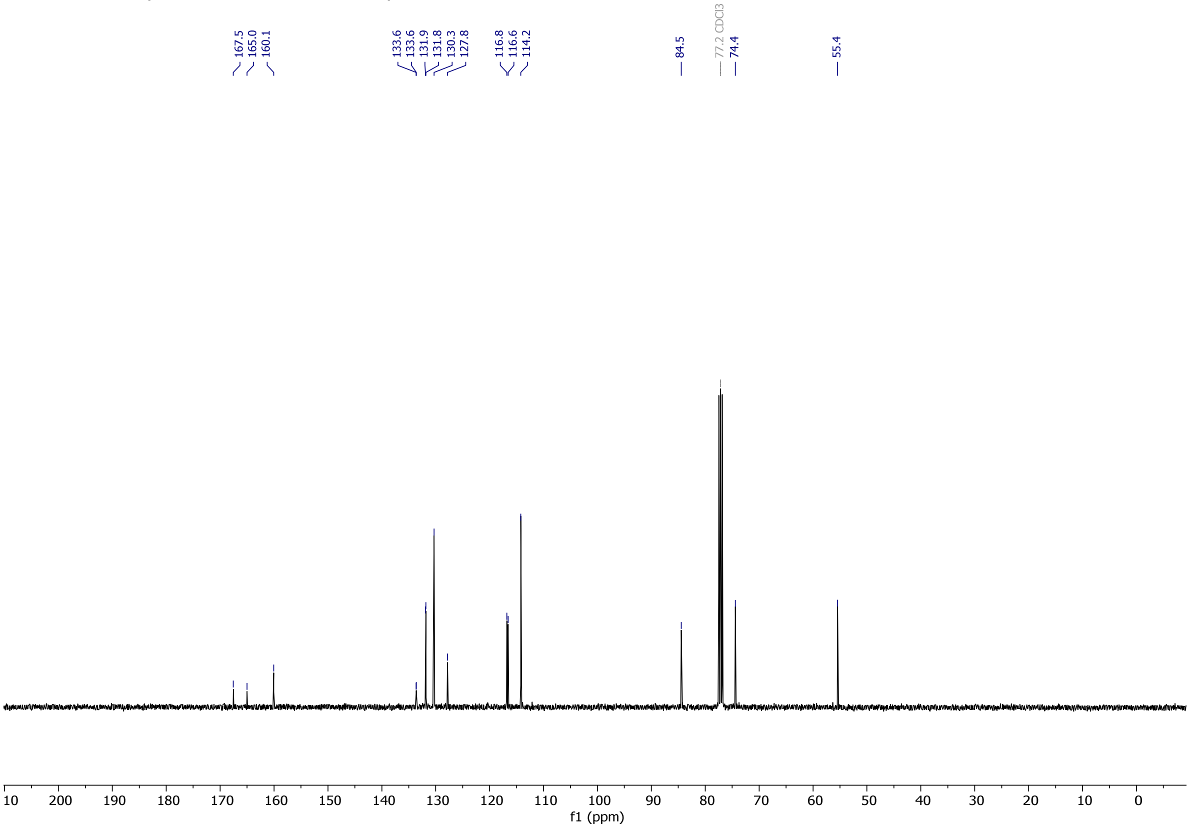

^{19}F NMR ($CDCl_3$; 376 MHz)

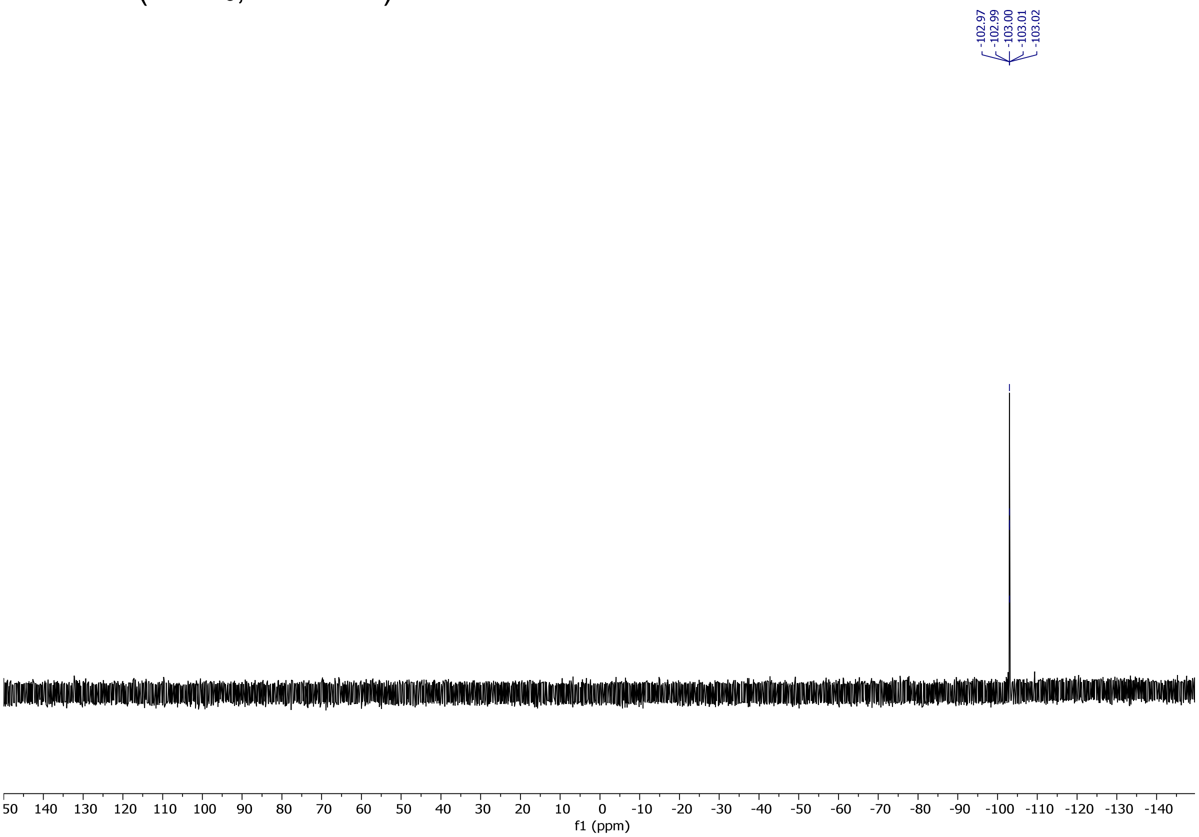

^{1}H NMR ($CDCl_3$, 400 MHz)

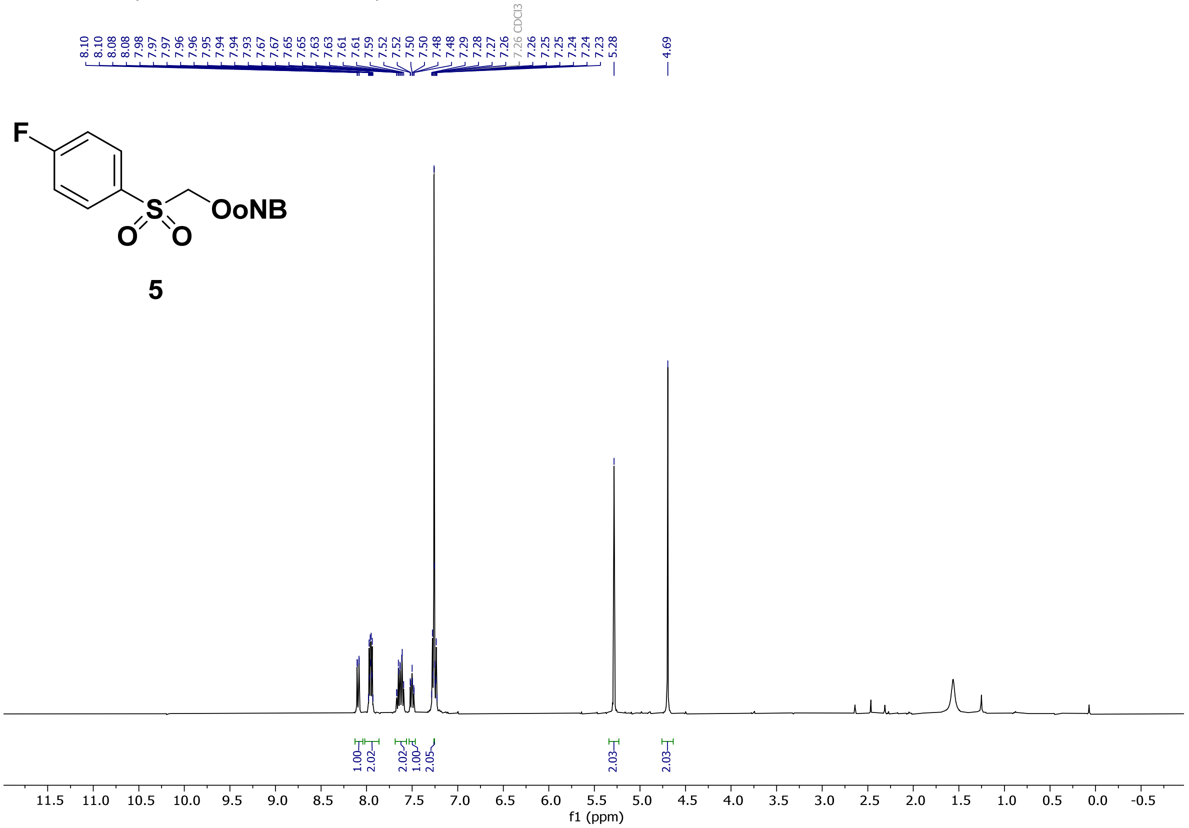

5

^{13}C NMR ($CDCl_3$; 101 MHz)

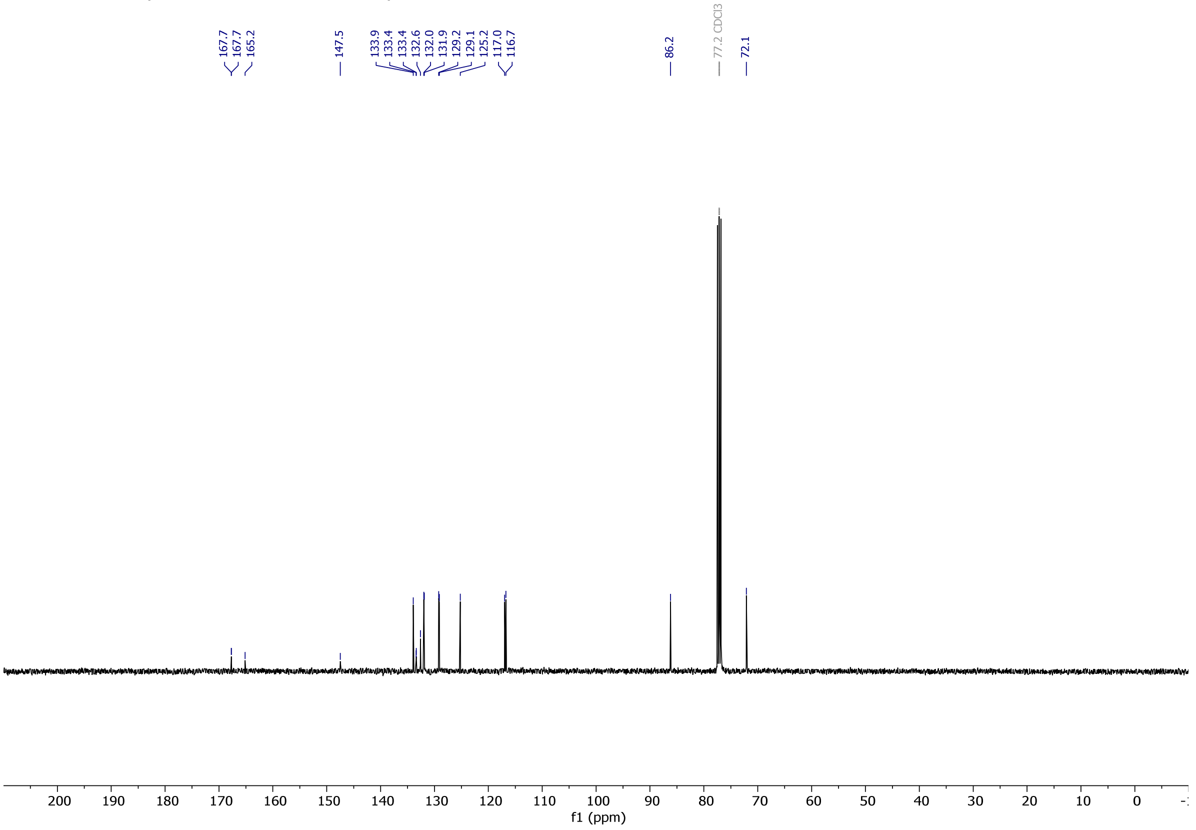

^{19}F NMR ($CDCl_3$; 376 MHz)

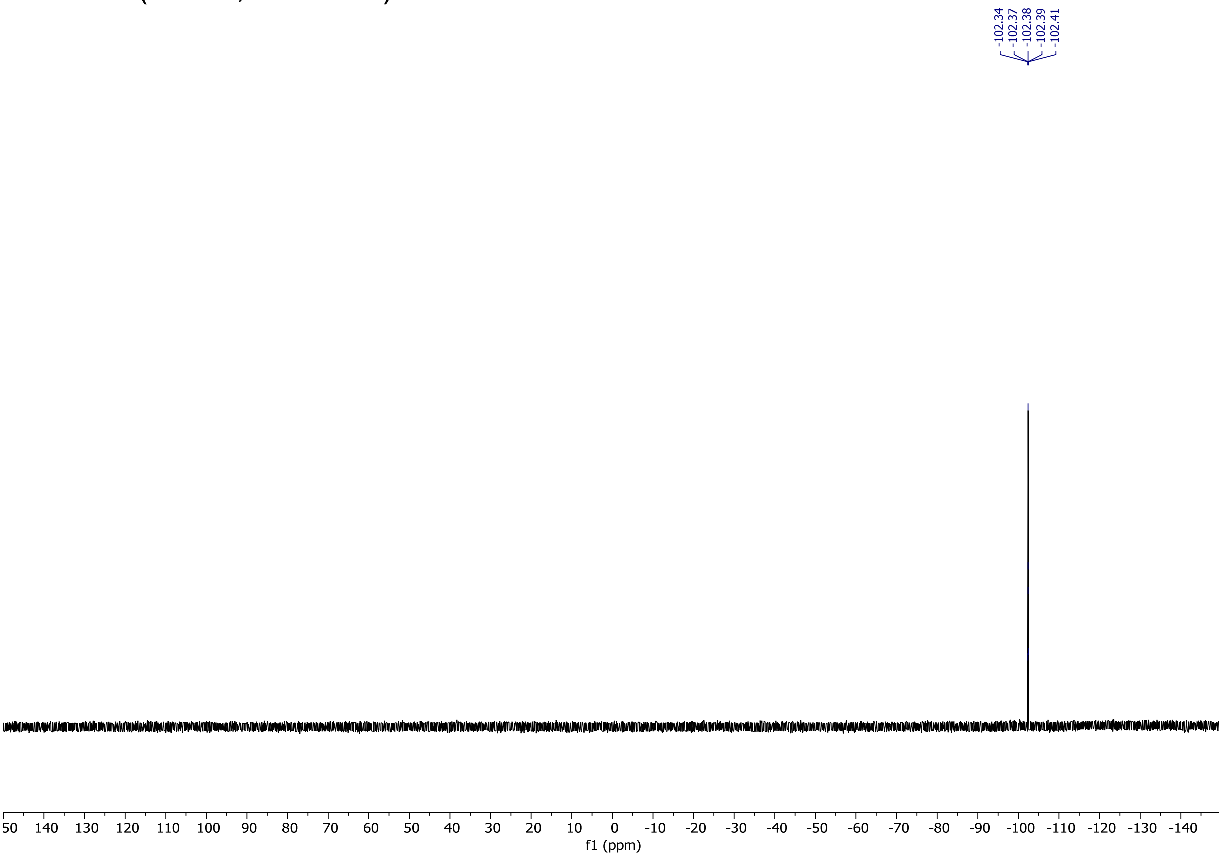

^{1}H NMR ($CDCl_3$, 400 MHz)

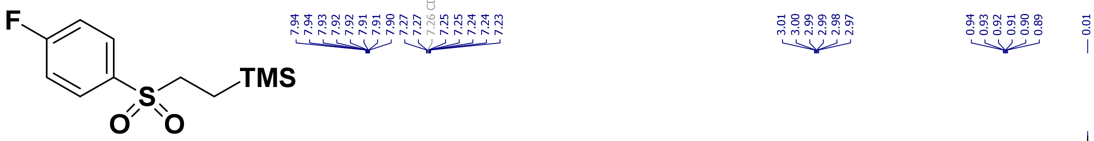

6

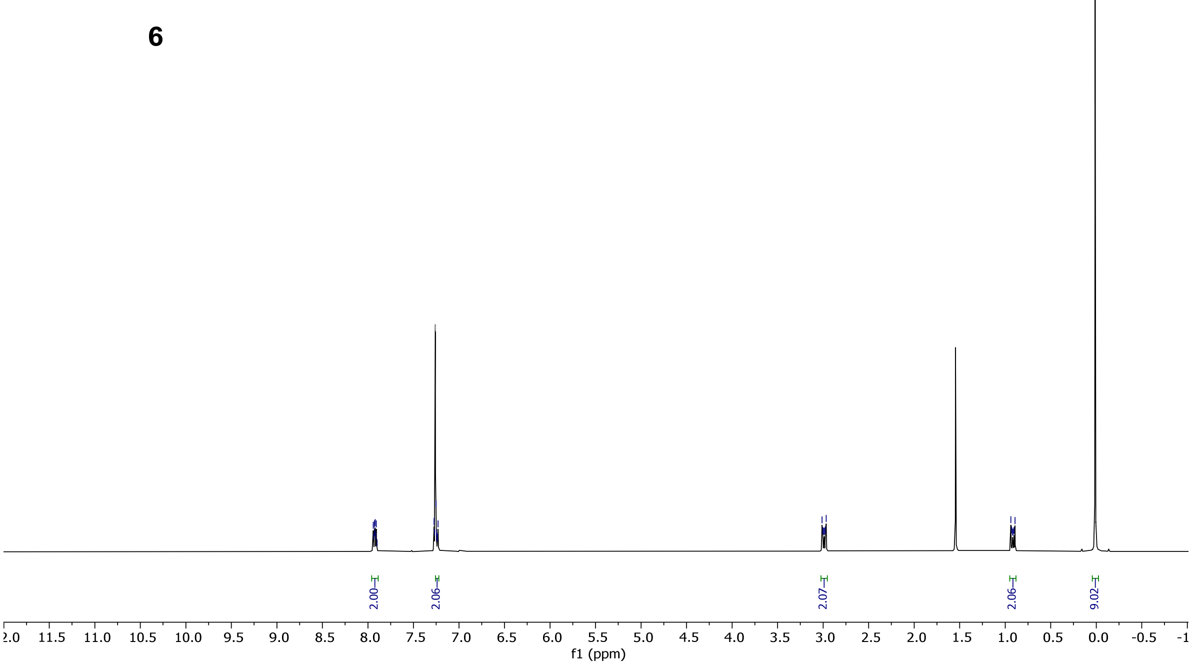

^{13}C NMR ($CDCl_3$; 101 MHz)

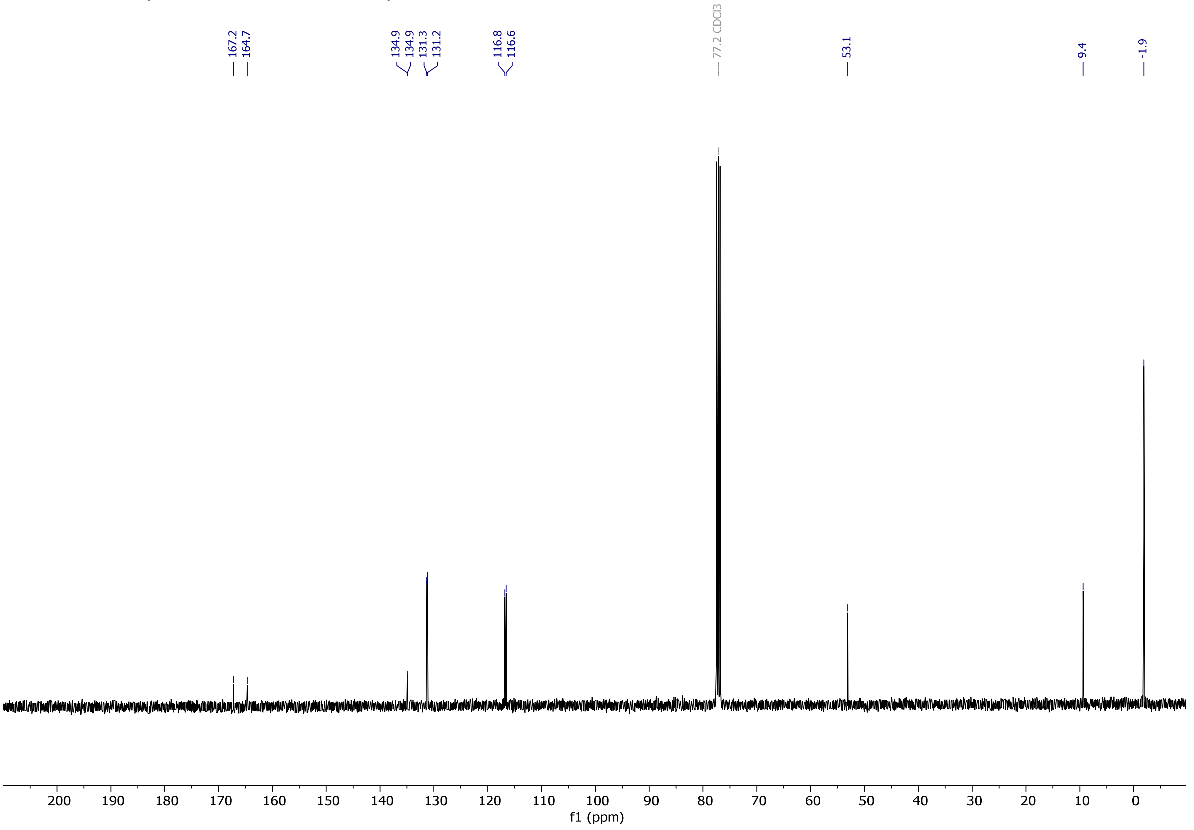

^{19}F NMR ($CDCl_3$; 376 MHz)

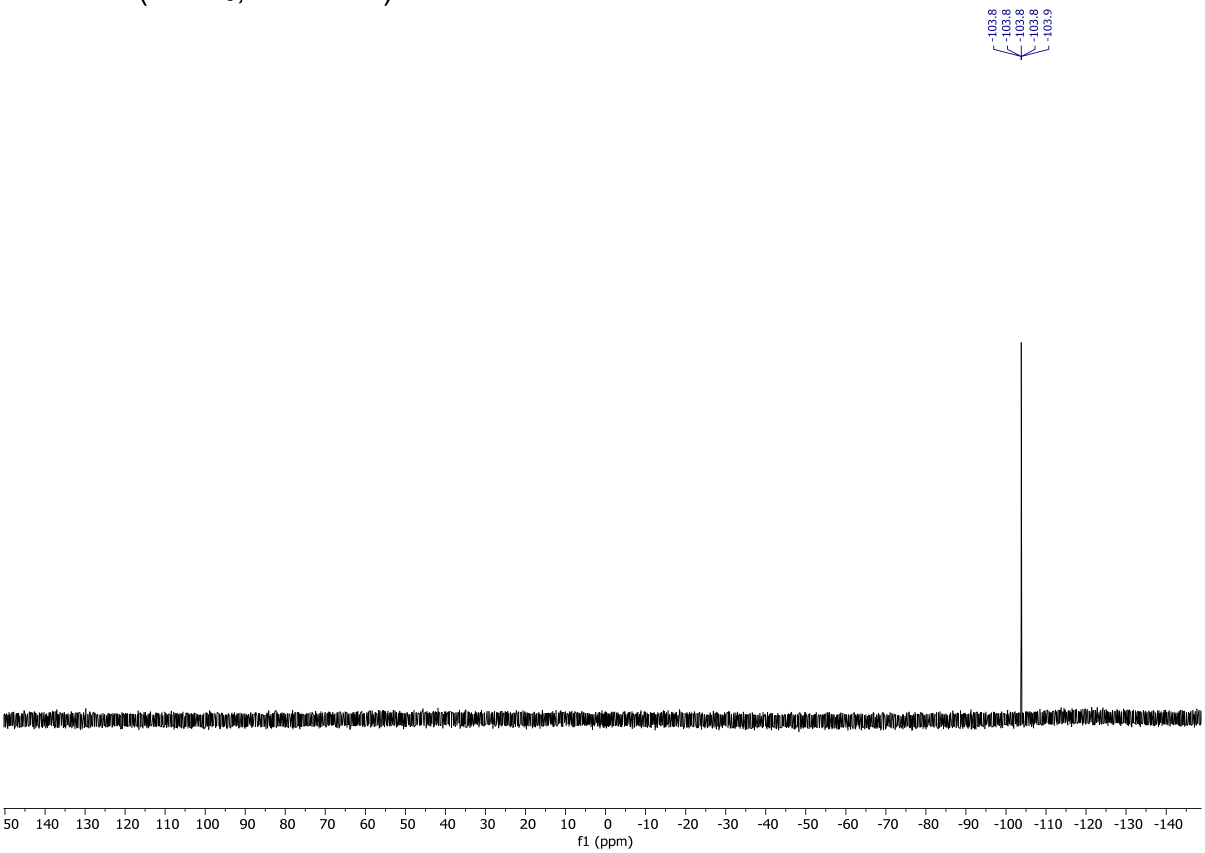

^{1}H NMR ($CDCl_3$, 400 MHz)

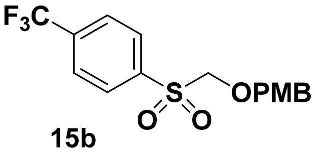

15b

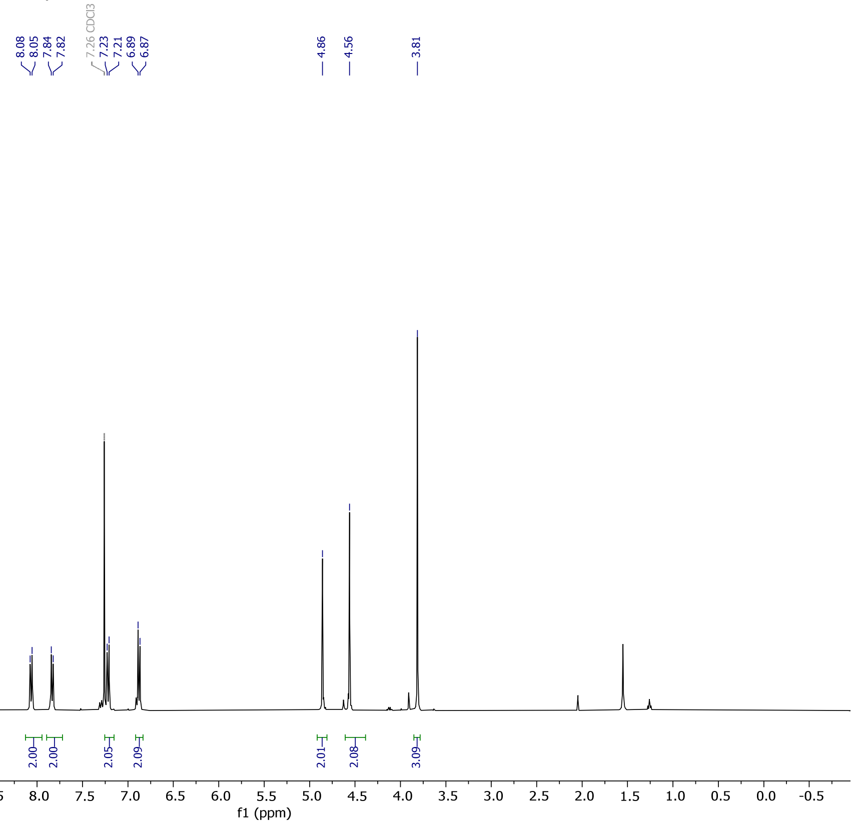

^{13}C NMR ($CDCl_3$; 101 MHz)

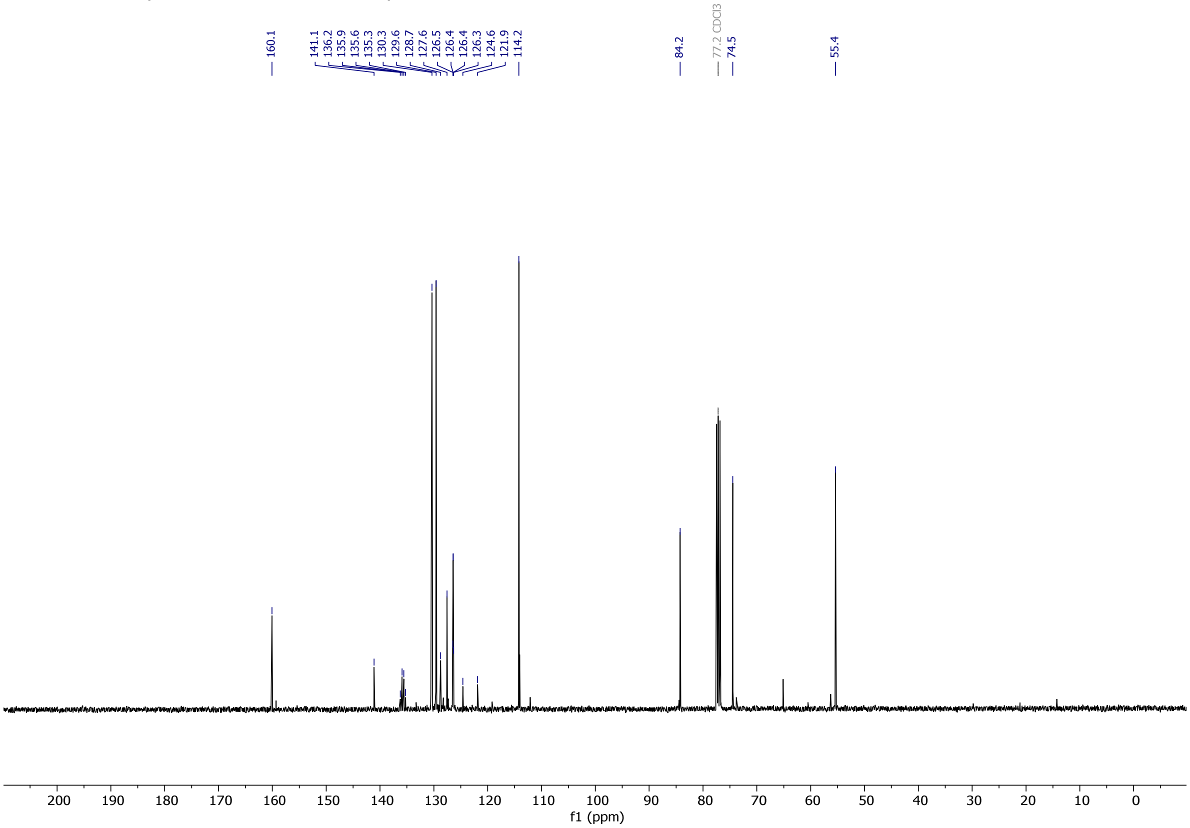

^{19}F NMR ($CDCl_3$; 376 MHz)

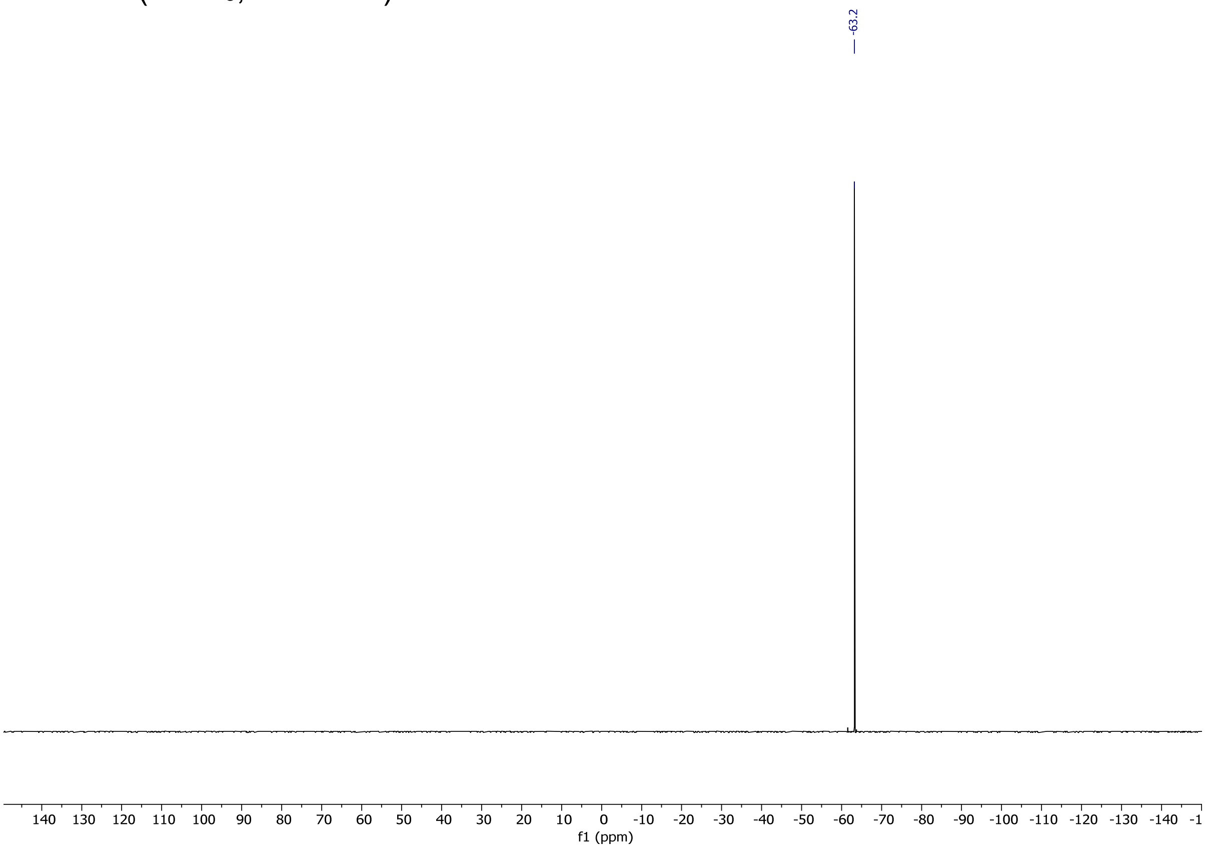

^{1}H NMR ($CDCl_3$, 400 MHz)

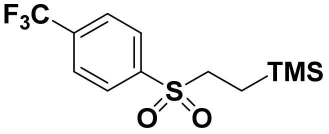

15c

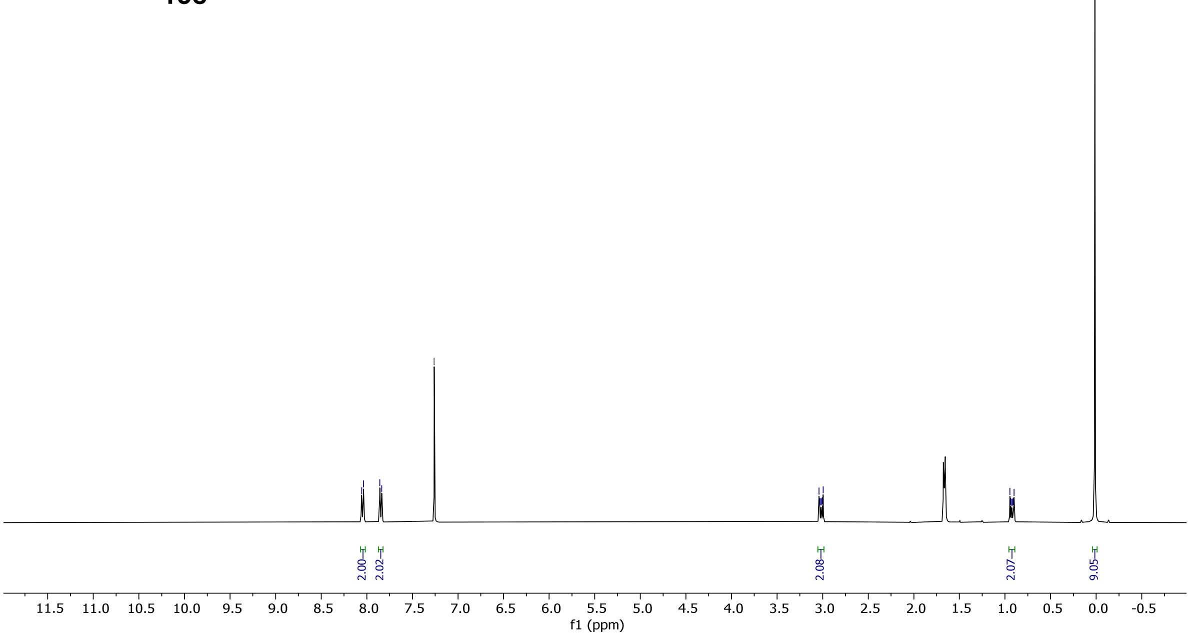

^{13}C NMR ($CDCl_3$; 101 MHz)

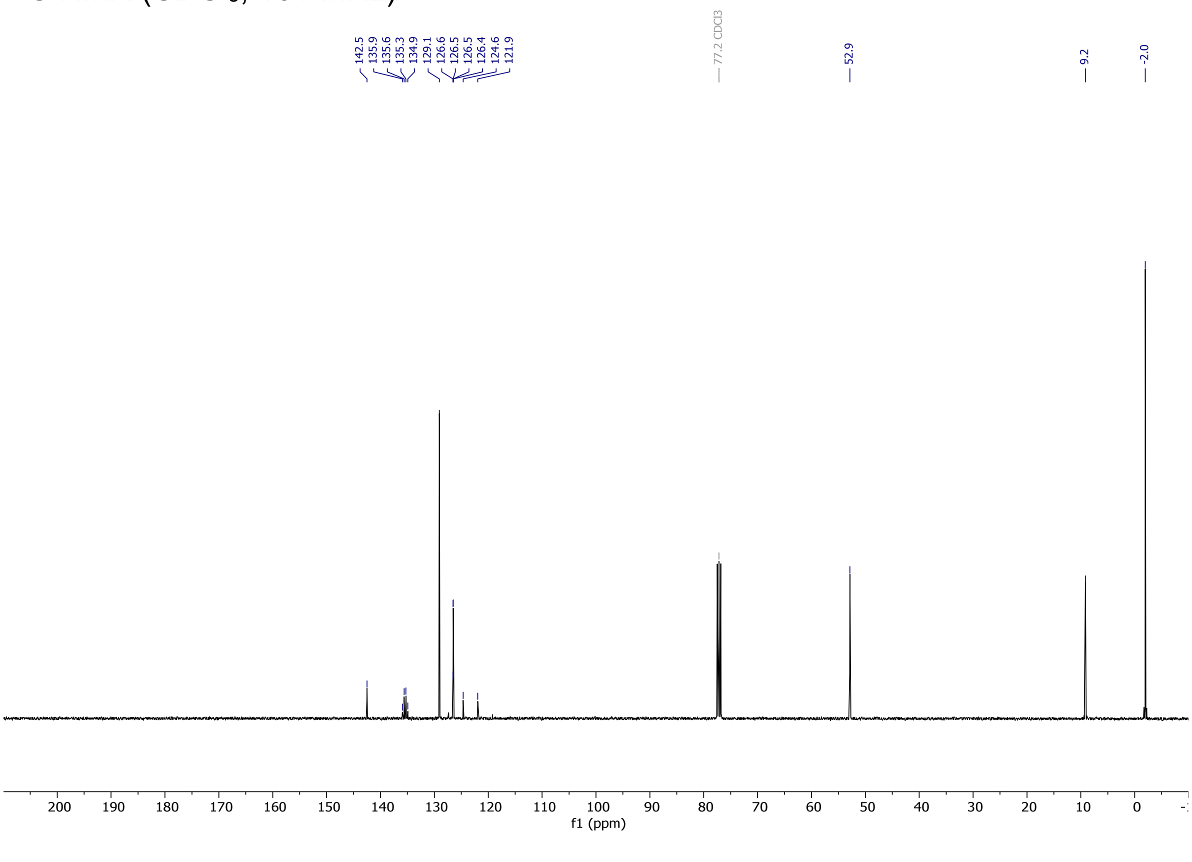

^{19}F NMR ($CDCl_3$; 376 MHz)

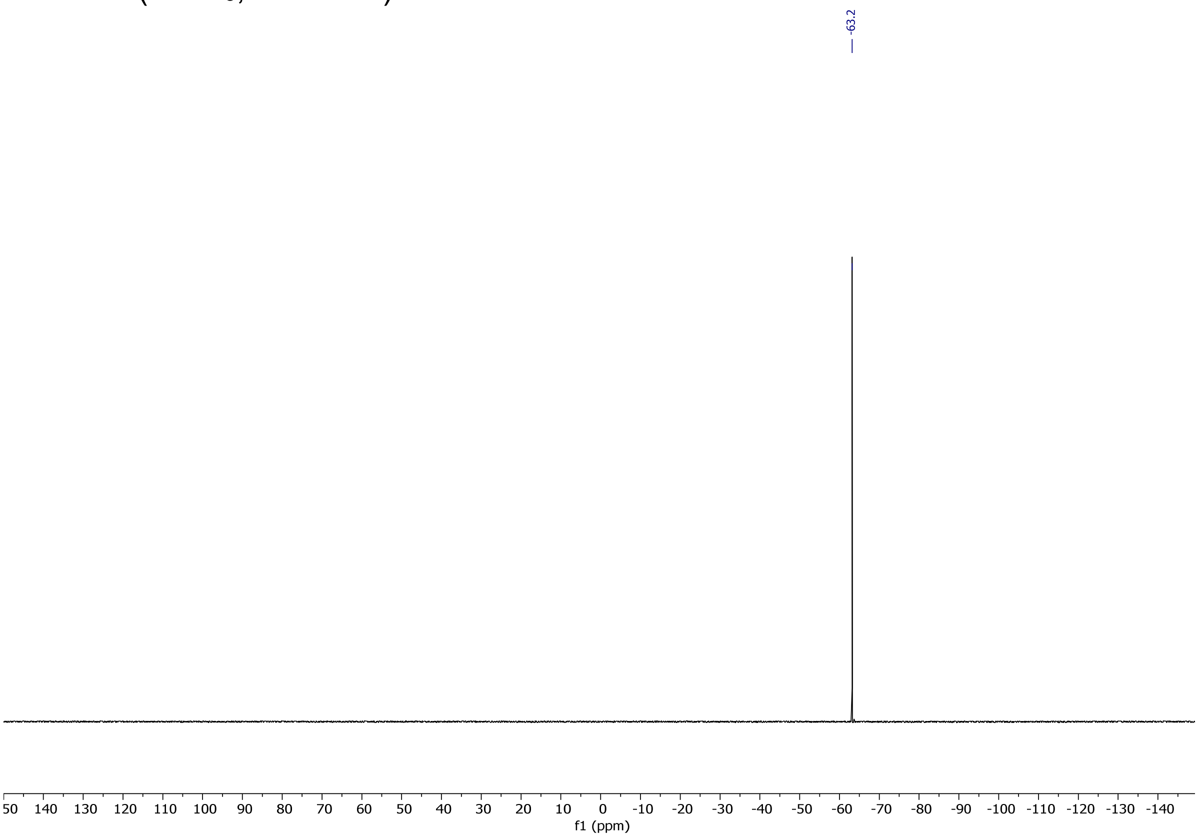

^{1}H NMR ($CDCl_3$, 400 MHz)

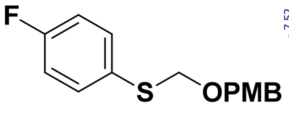

S10

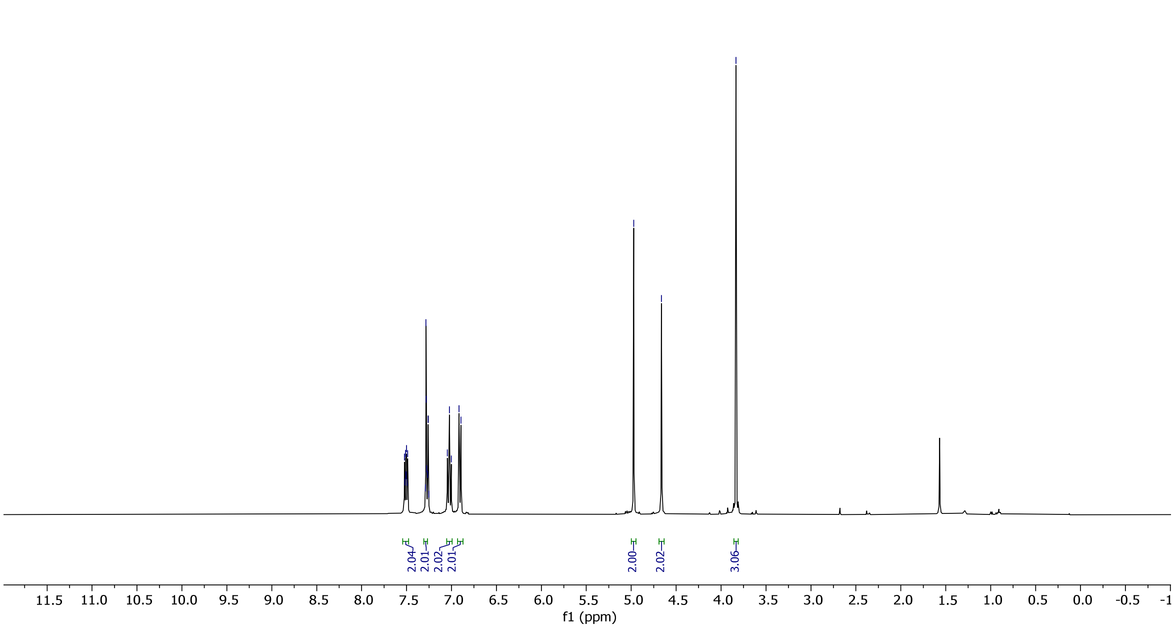

^{13}C NMR ($CDCl_3$; 101 MHz)

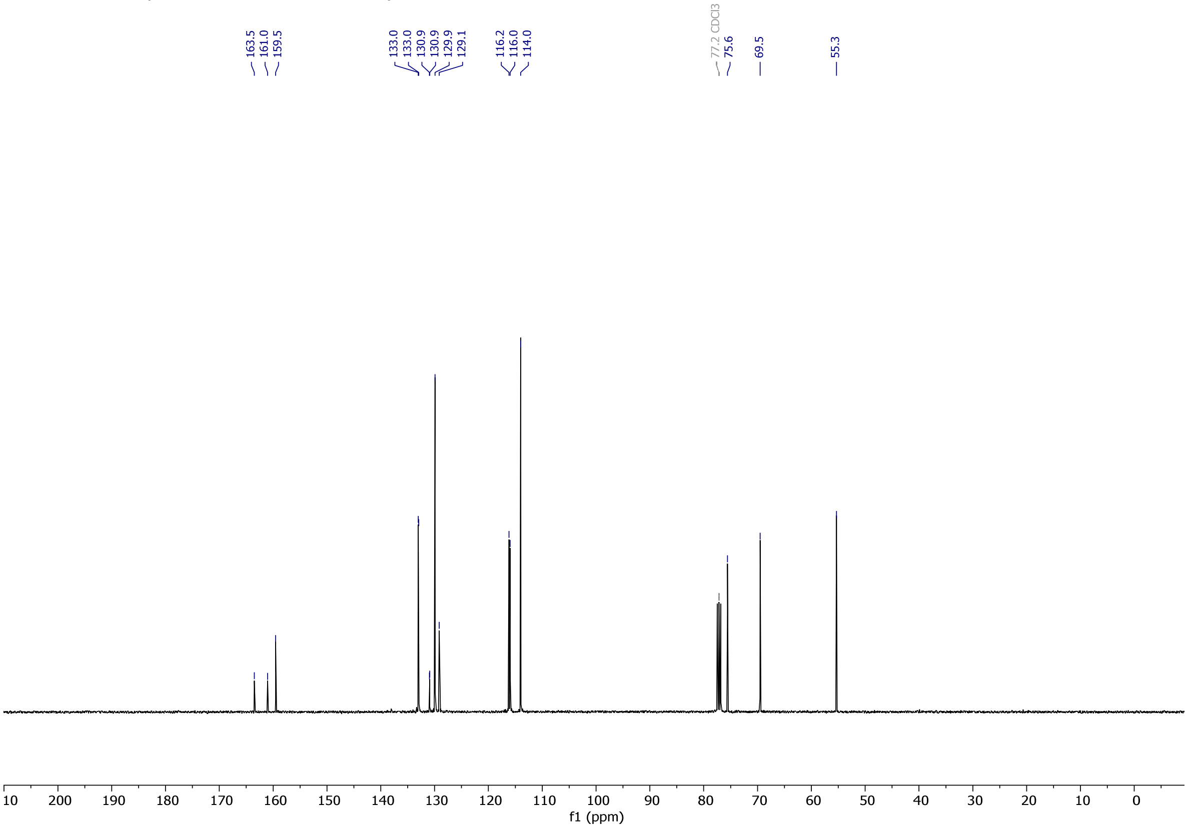

^{19}F NMR ($CDCl_3$; 376 MHz)

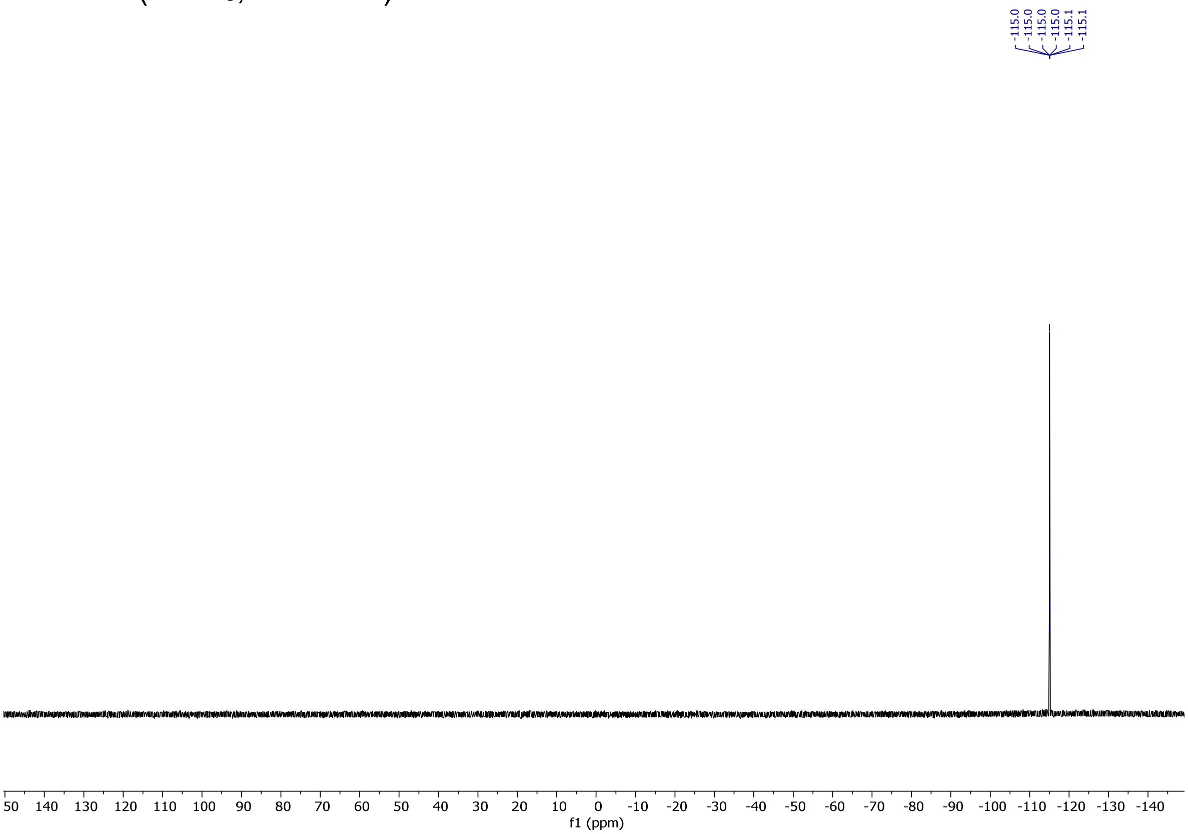

^1H NMR ($CDCl_3$, 400 MHz)

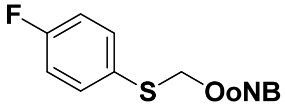

S11

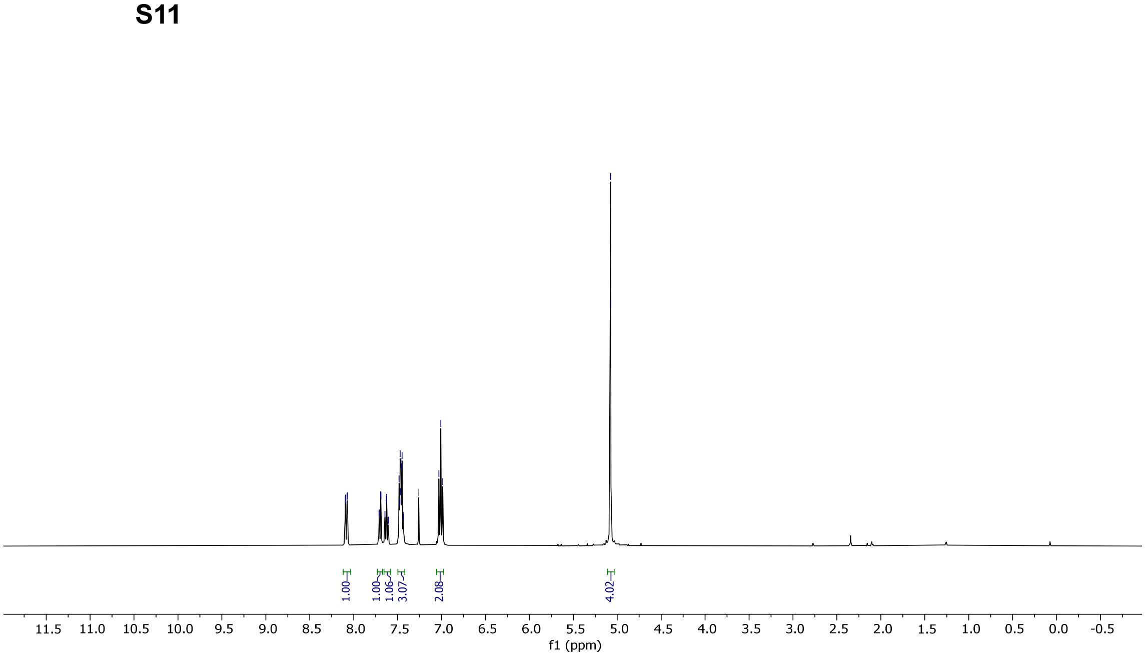

^{13}C NMR ($CDCl_3$; 101 MHz)

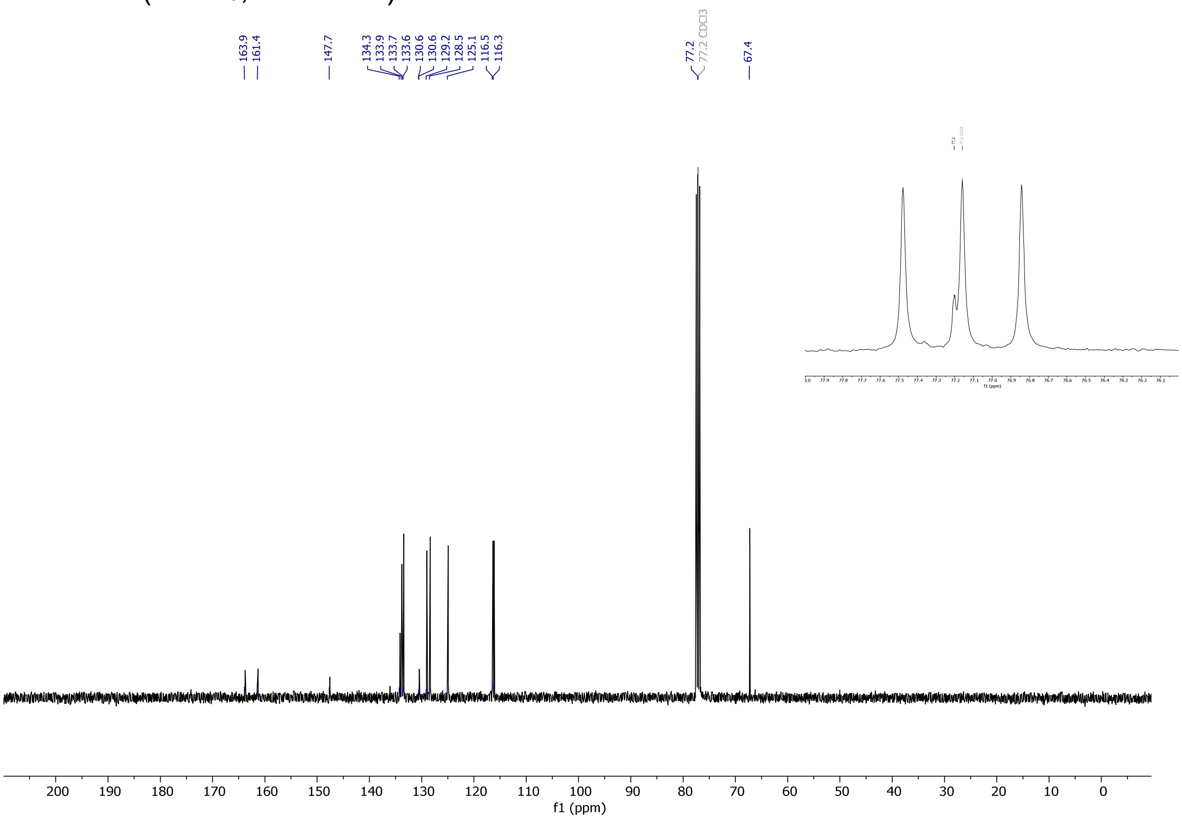

HSQC ($f1$: 101 MHz, $f2$: 400 MHz) NMR

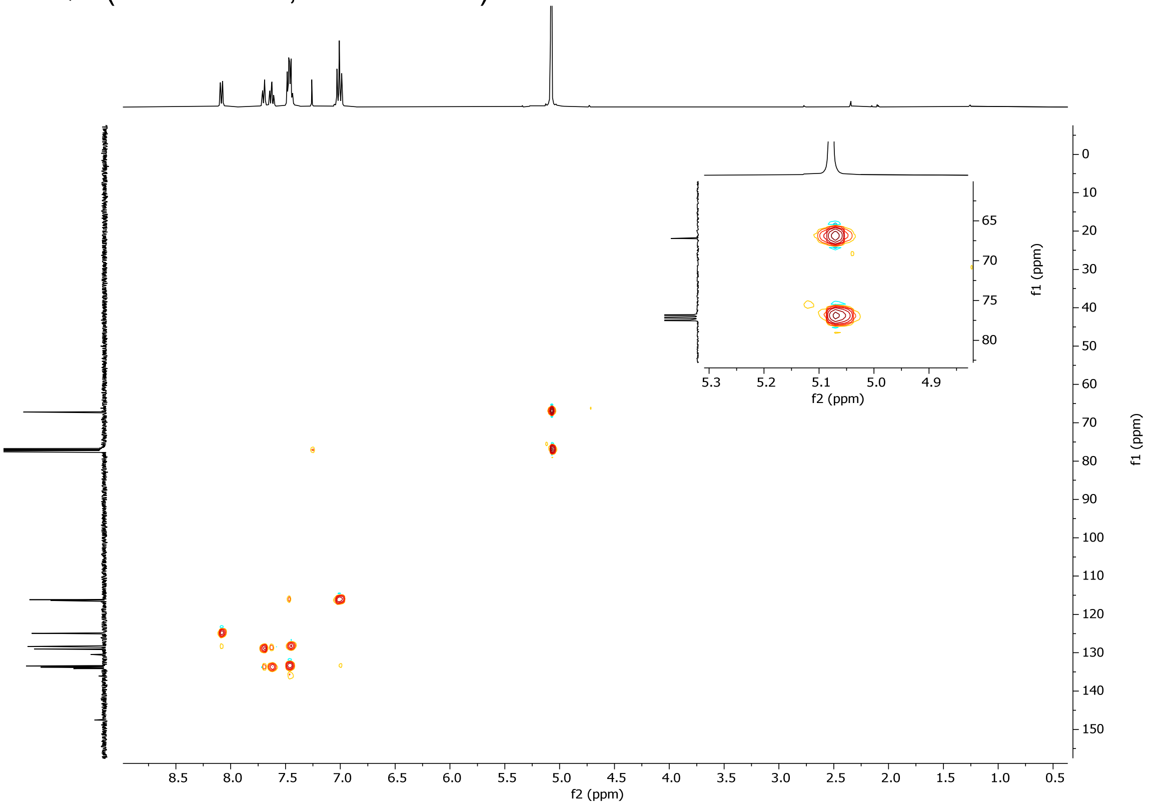

^{19}F NMR ($CDCl_3$; 376 MHz)

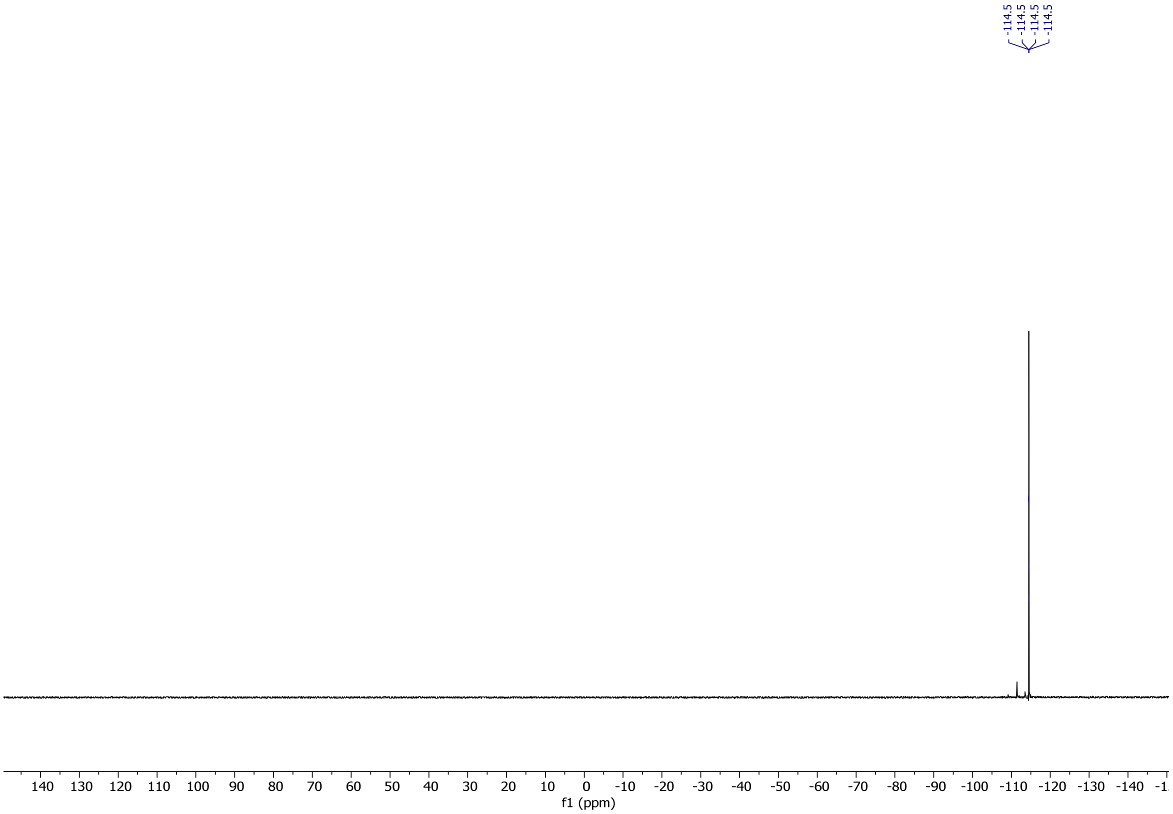

^{1}H NMR ($CDCl_3$, 400 MHz)

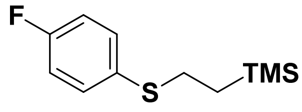

S12

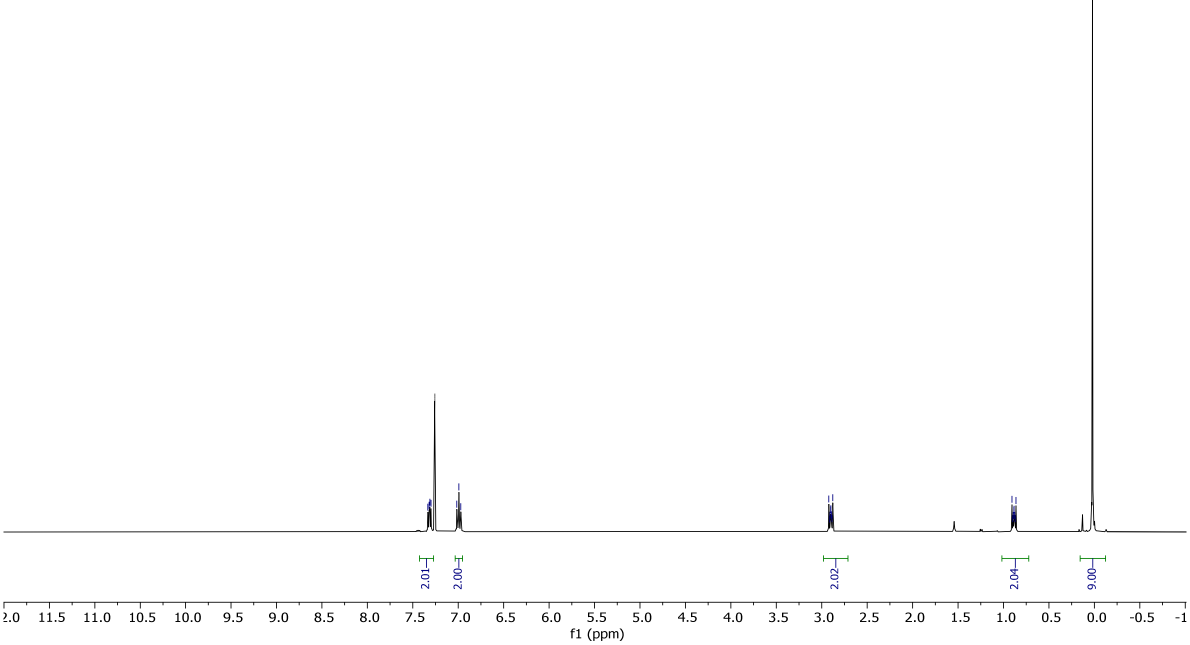

^{13}C NMR ($CDCl_3$; 101 MHz)

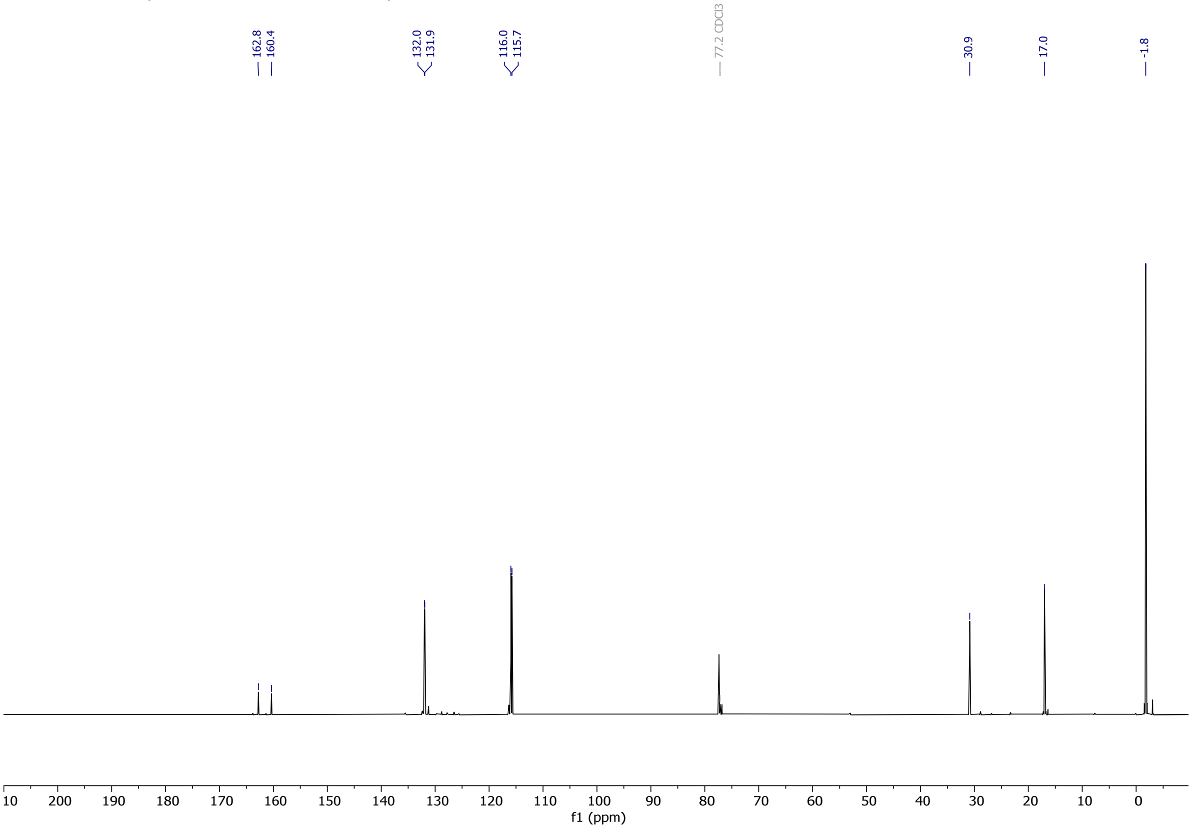

^{19}F NMR ($CDCl_3$; 376 MHz)

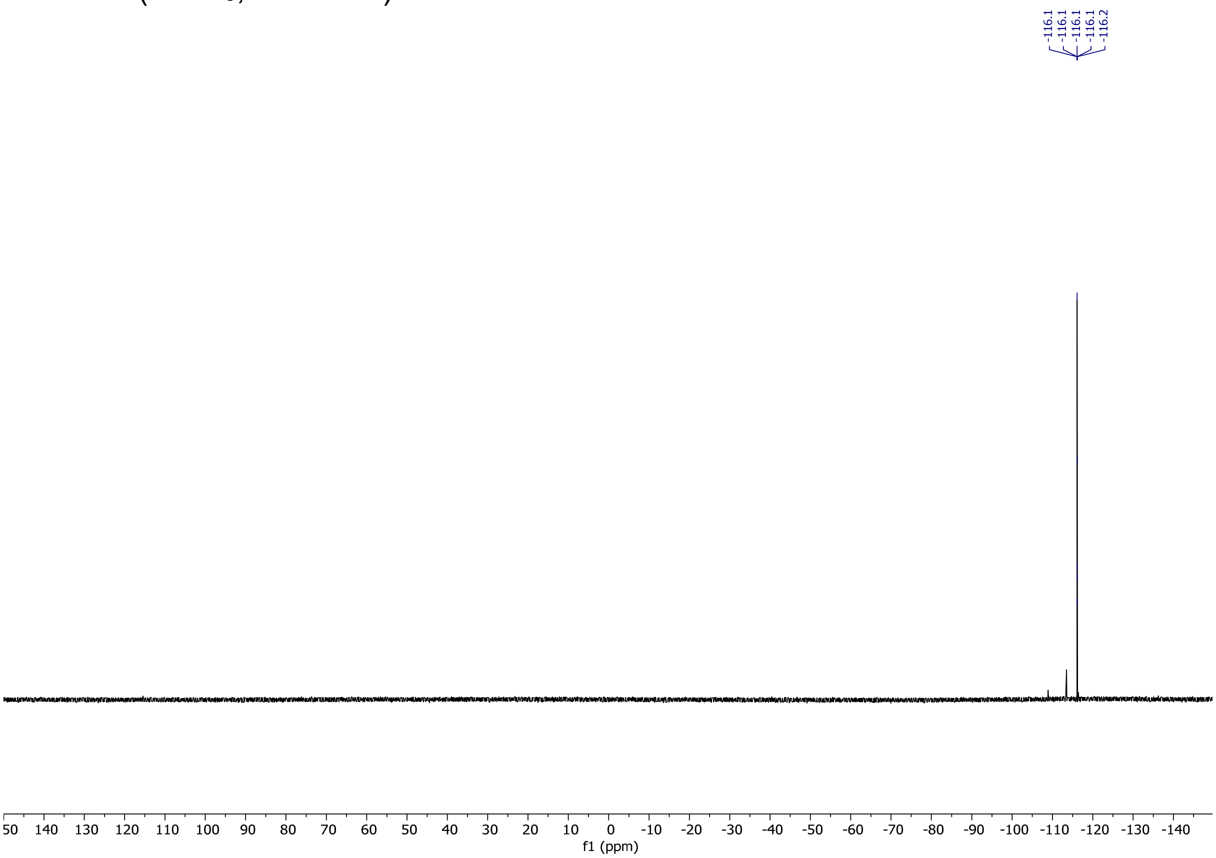

^1H NMR ($CDCl_3$, 400 MHz)

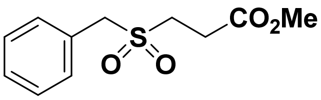

1Bn

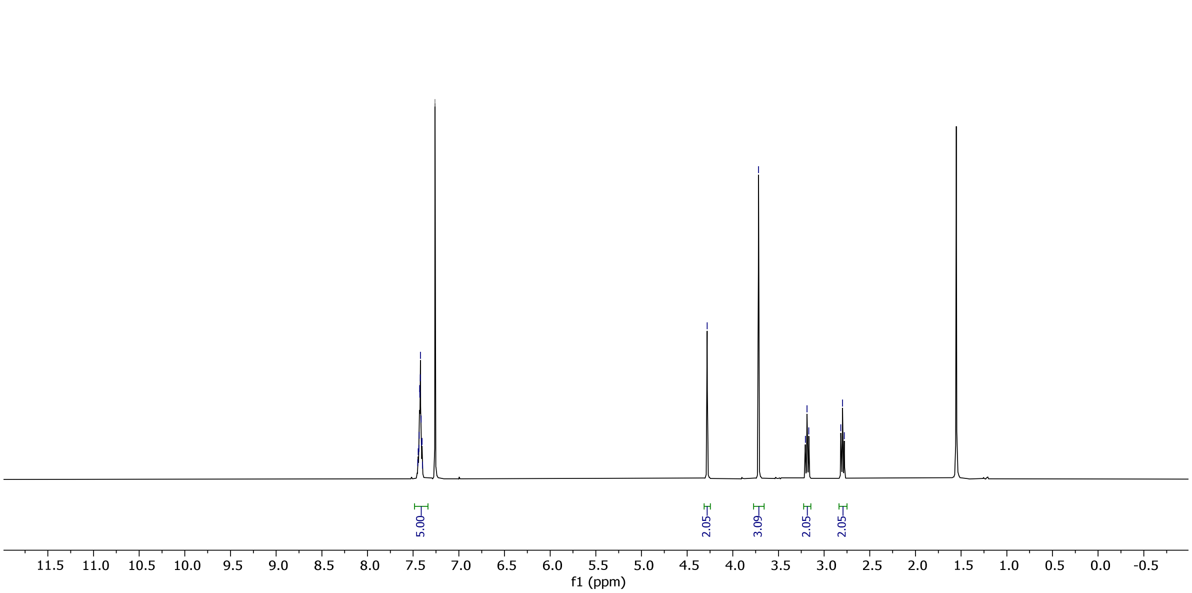

^{13}C NMR ($CDCl_3$; 101 MHz)

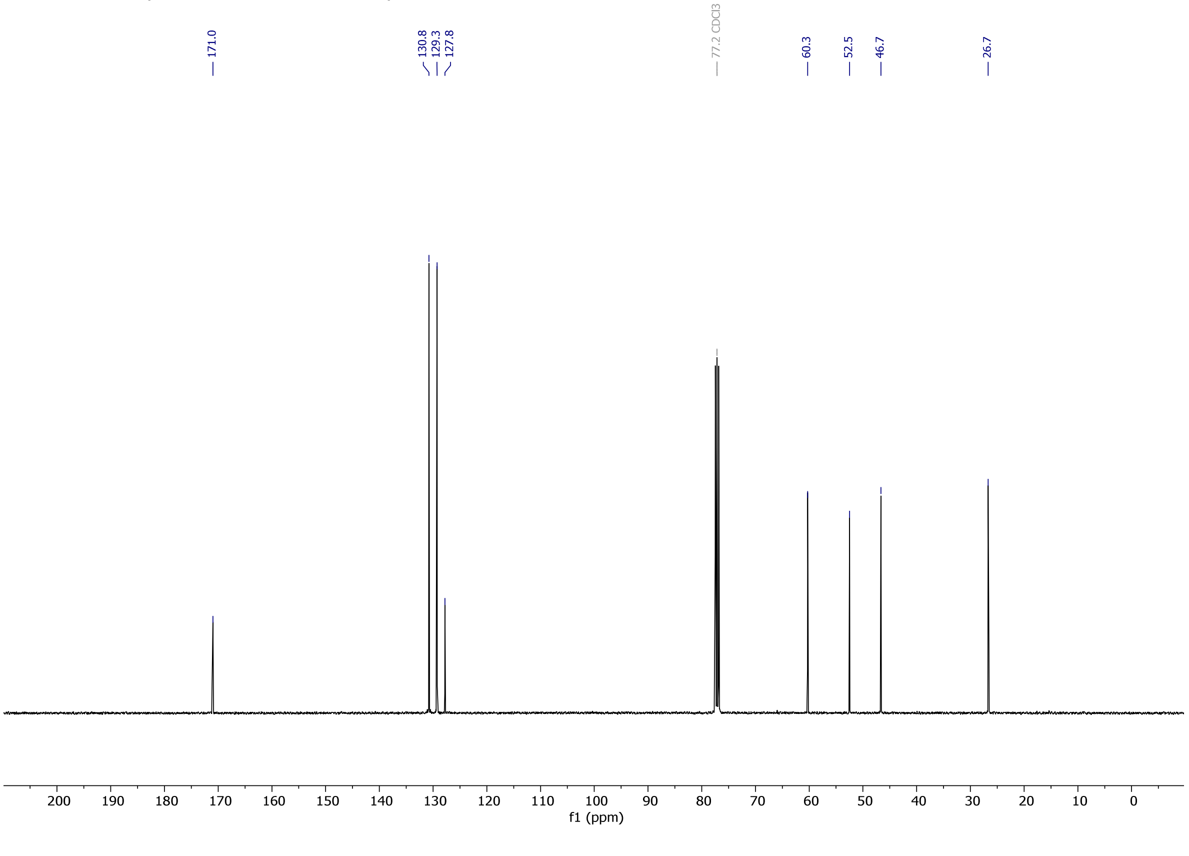

^1H NMR ($CDCl_3$, 400 MHz)

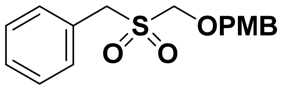

4Bn

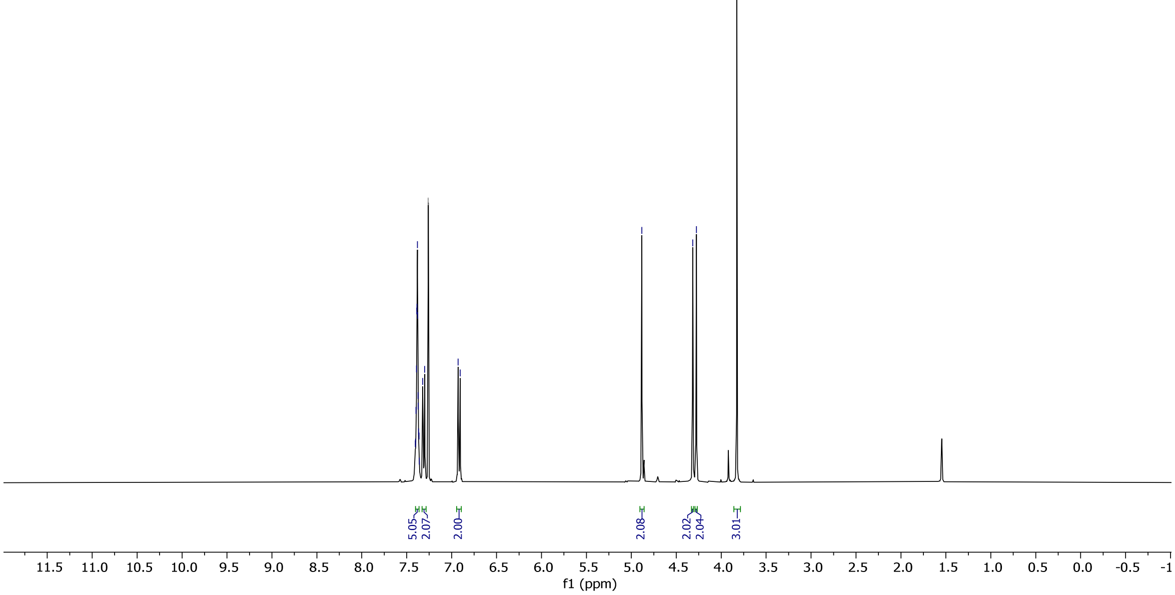

^{13}C NMR ($CDCl_3$; 101 MHz)

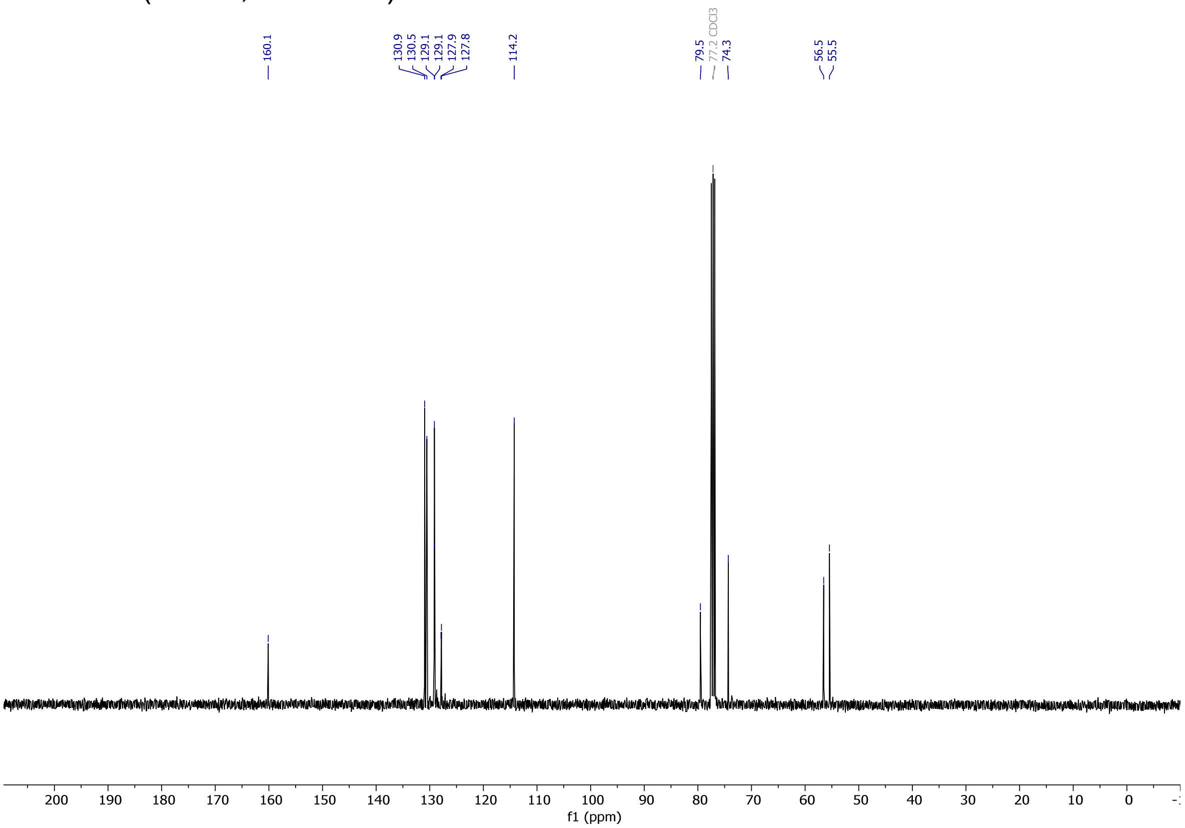

^{1}H NMR ($CDCl_3$, 400 MHz)

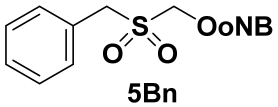

5Bn

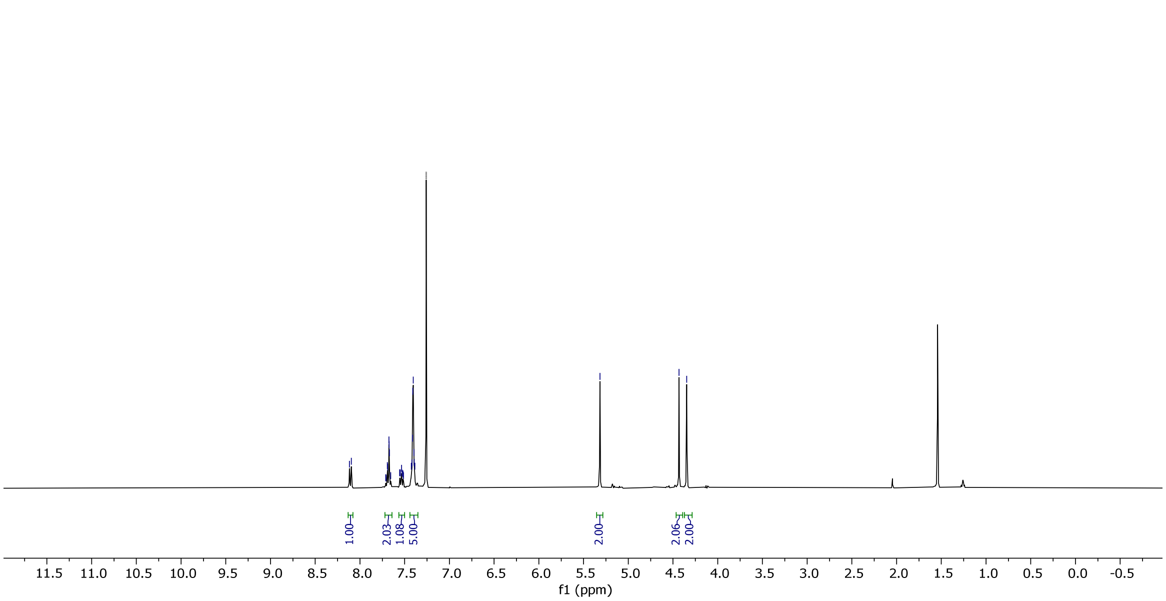

^{13}C NMR ($CDCl_3$; 101 MHz)

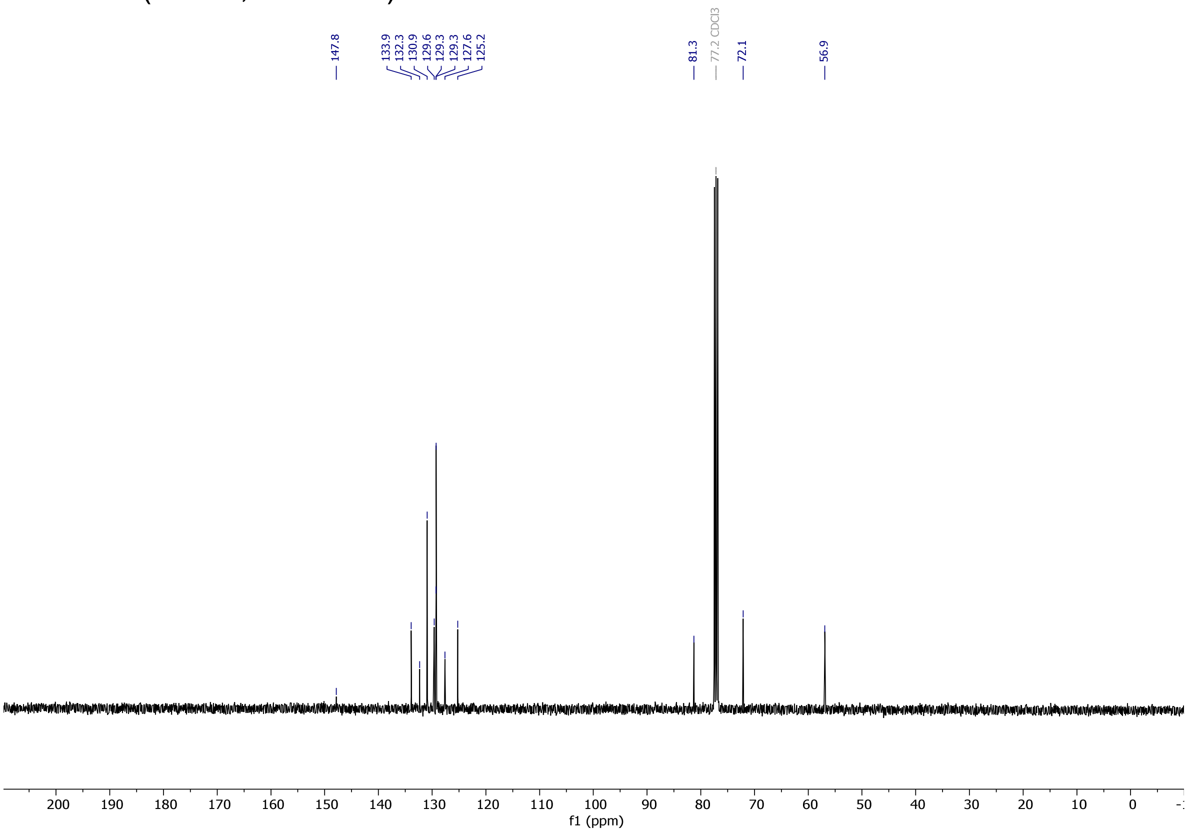

^1H NMR ($CDCl_3$, 400 MHz)

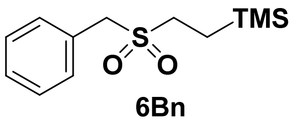

6Bn

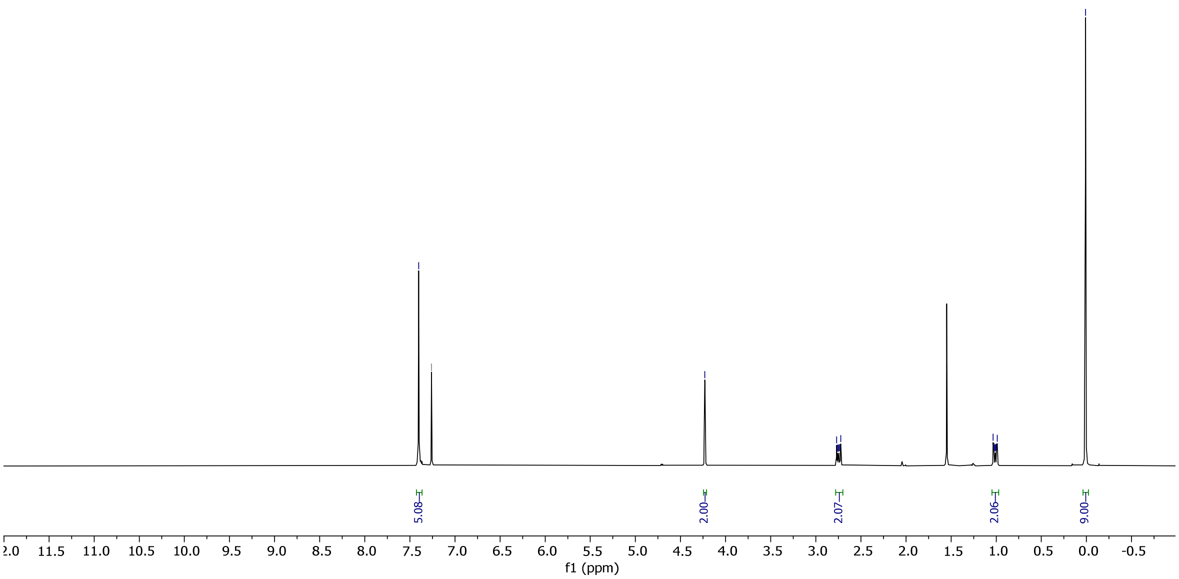

^{13}C NMR ($CDCl_3$; 101 MHz)

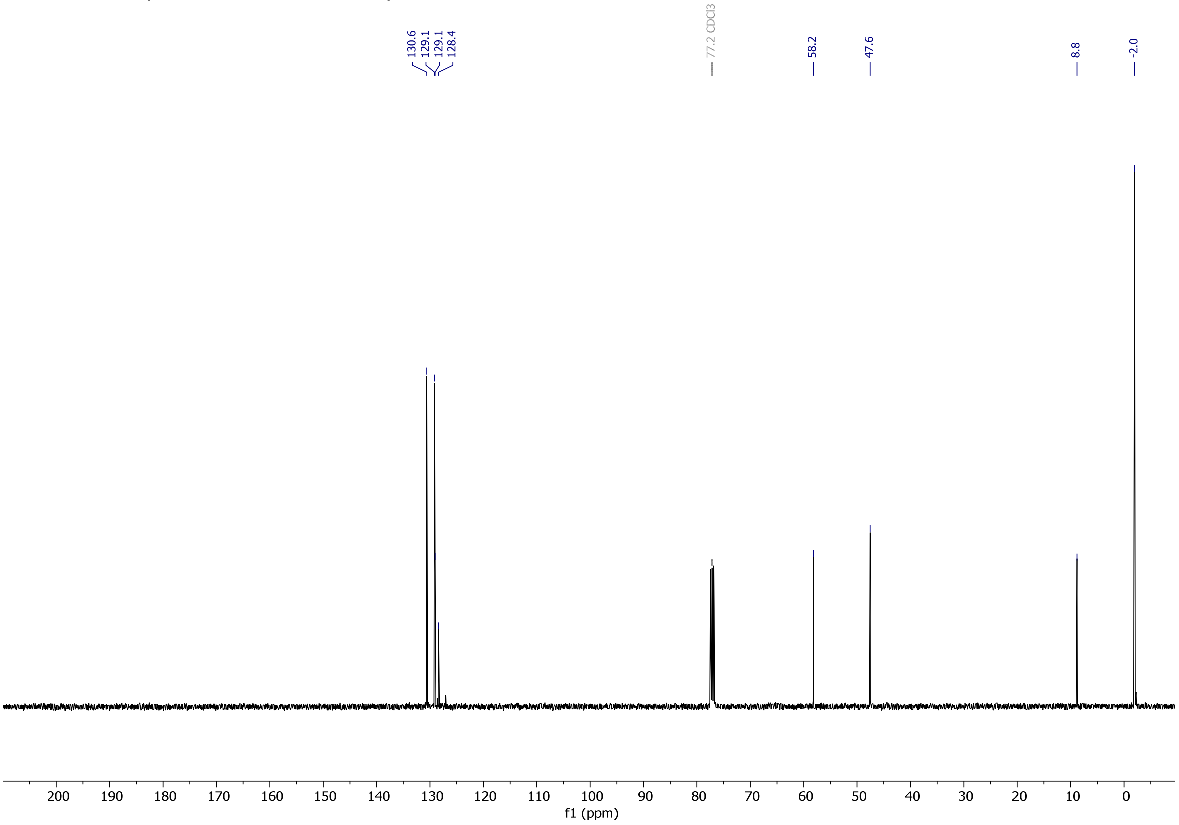

^{1}H NMR ($CDCl_3$, 400 MHz)

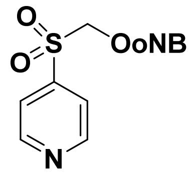

S13a

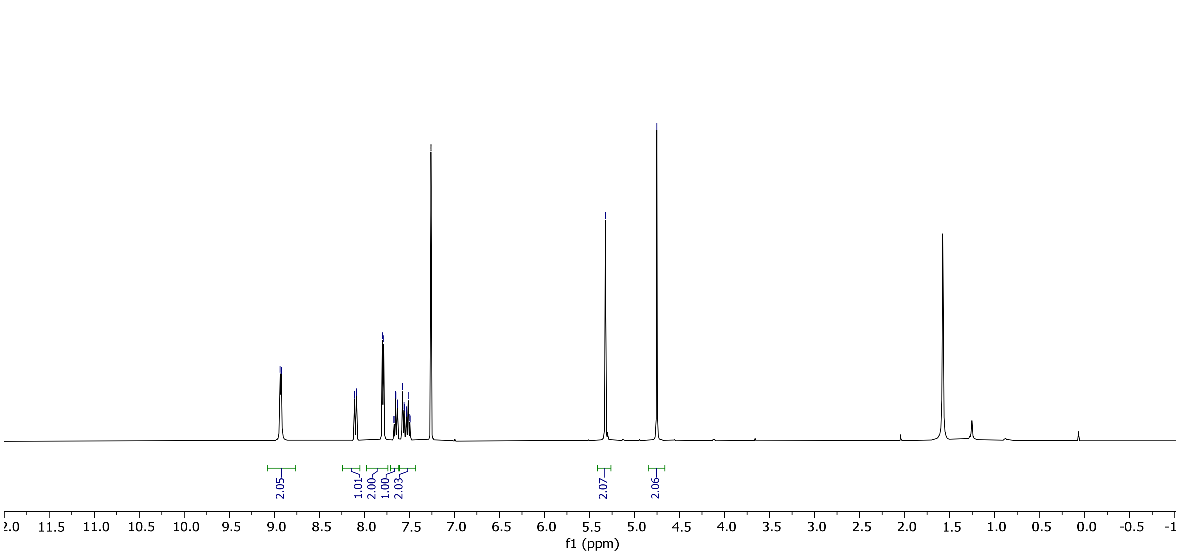

^{13}C NMR ($CDCl_3$; 101 MHz)

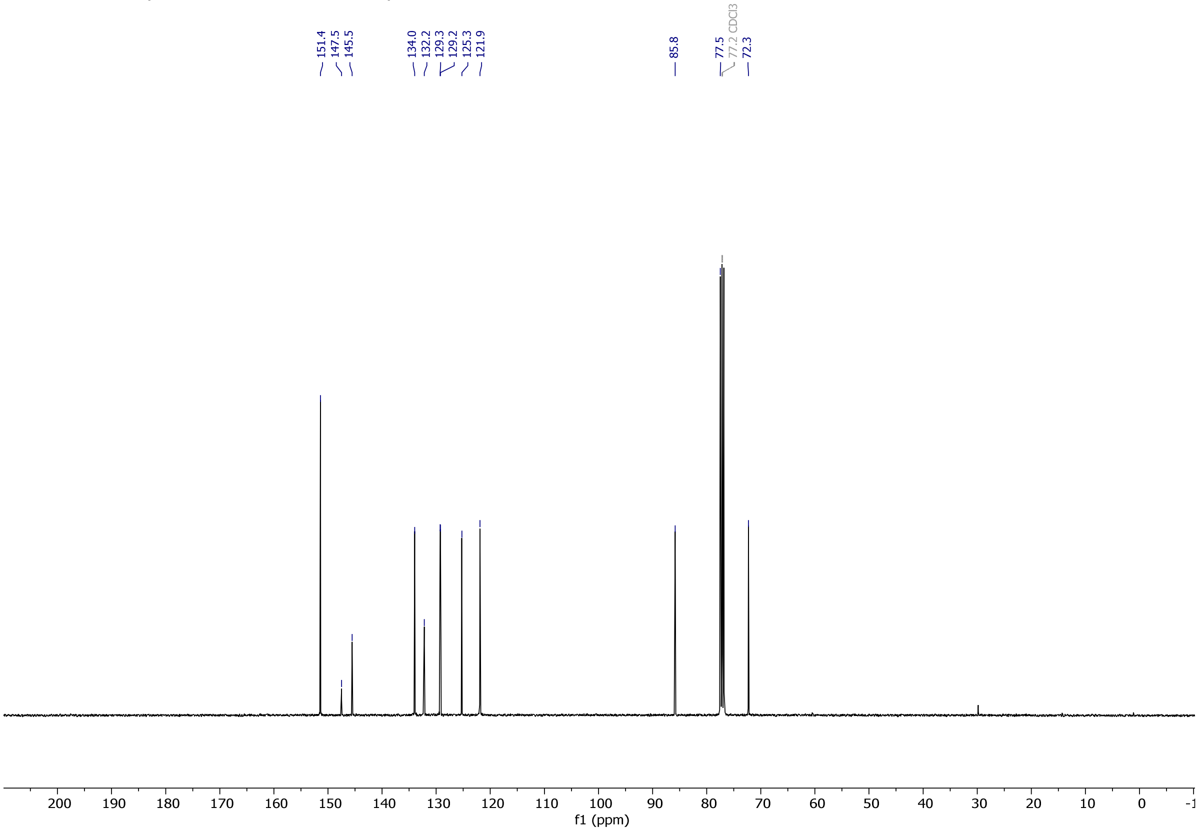

^{1}H NMR ($CDCl_3$, 400 MHz)

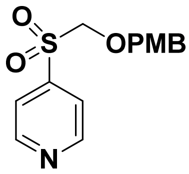

S13b

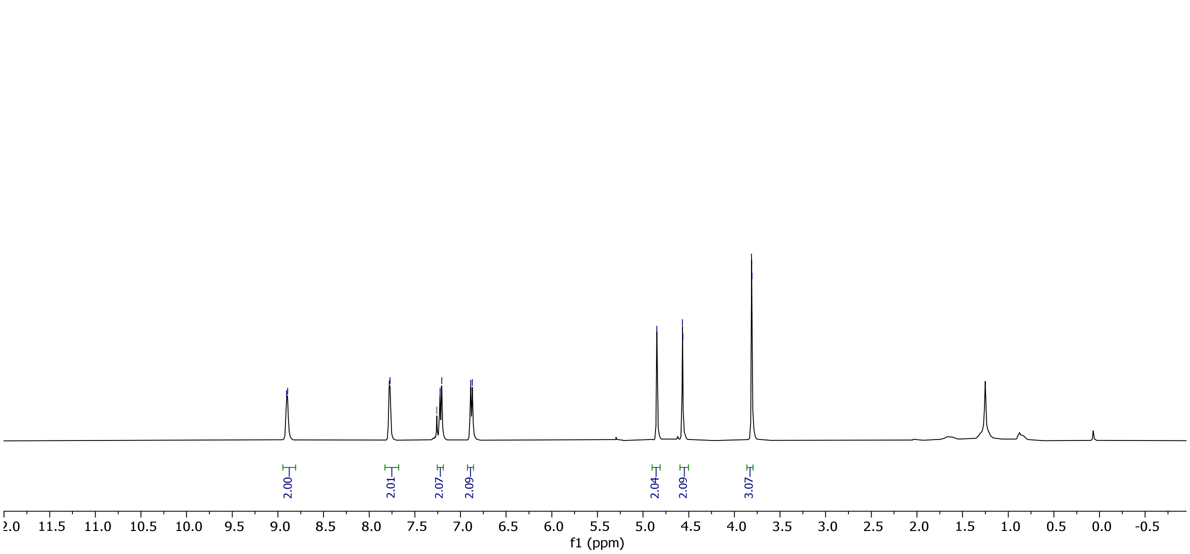

^{13}C NMR ($CDCl_3$; 101 MHz)

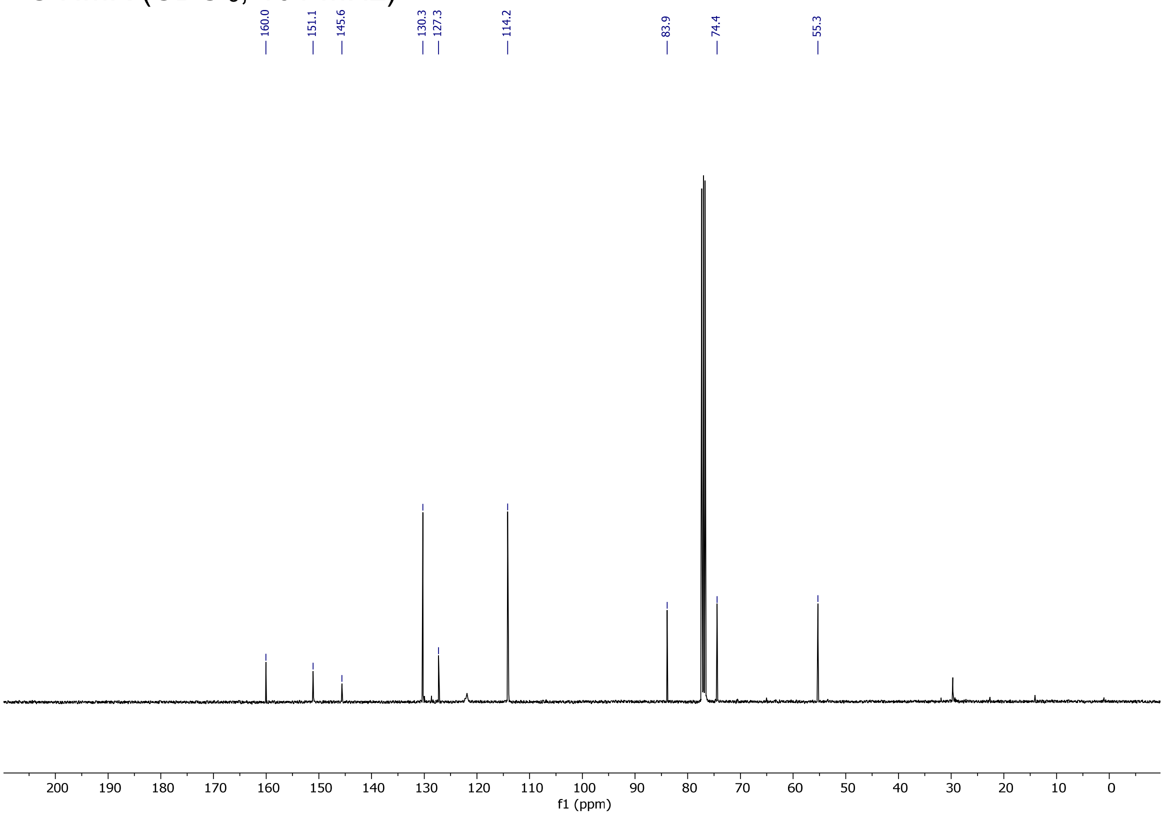

^1H NMR ($CDCl_3$, 400 MHz)

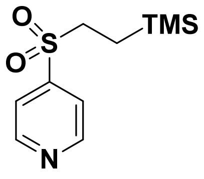

S13c

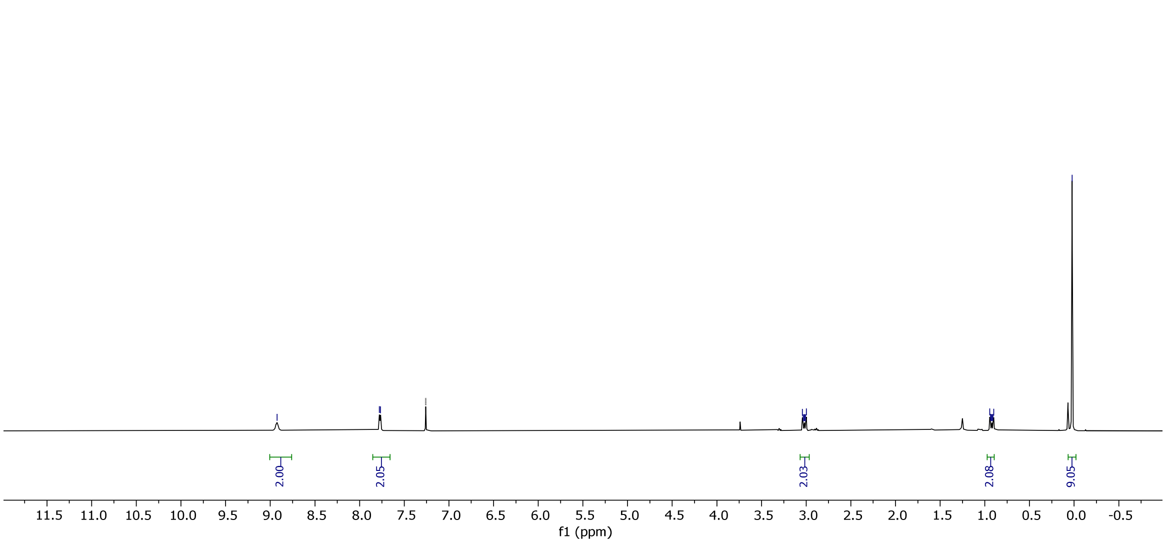

^{13}C NMR ($CDCl_3$; 101 MHz)

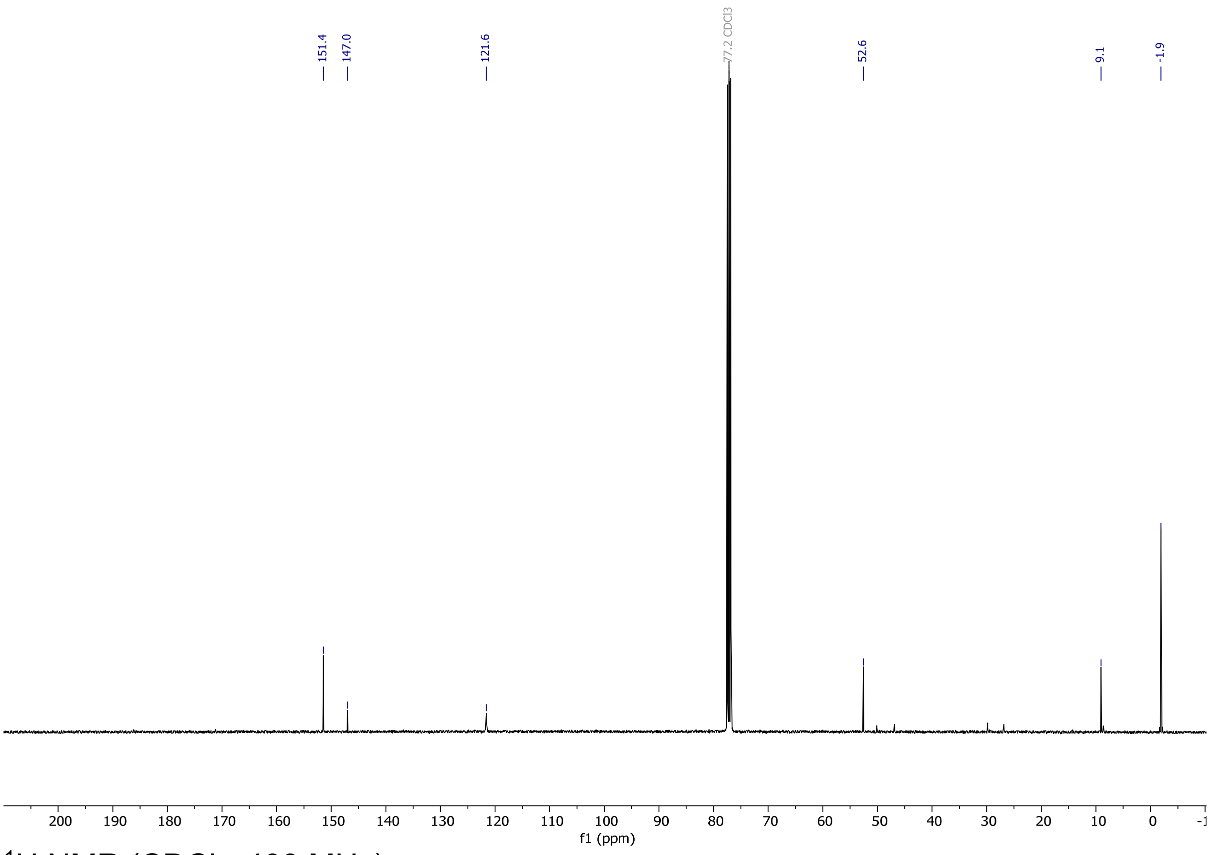

^{1}H NMR ($CDCl_3$, 400 MHz)

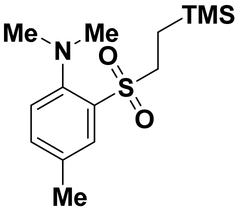

S14

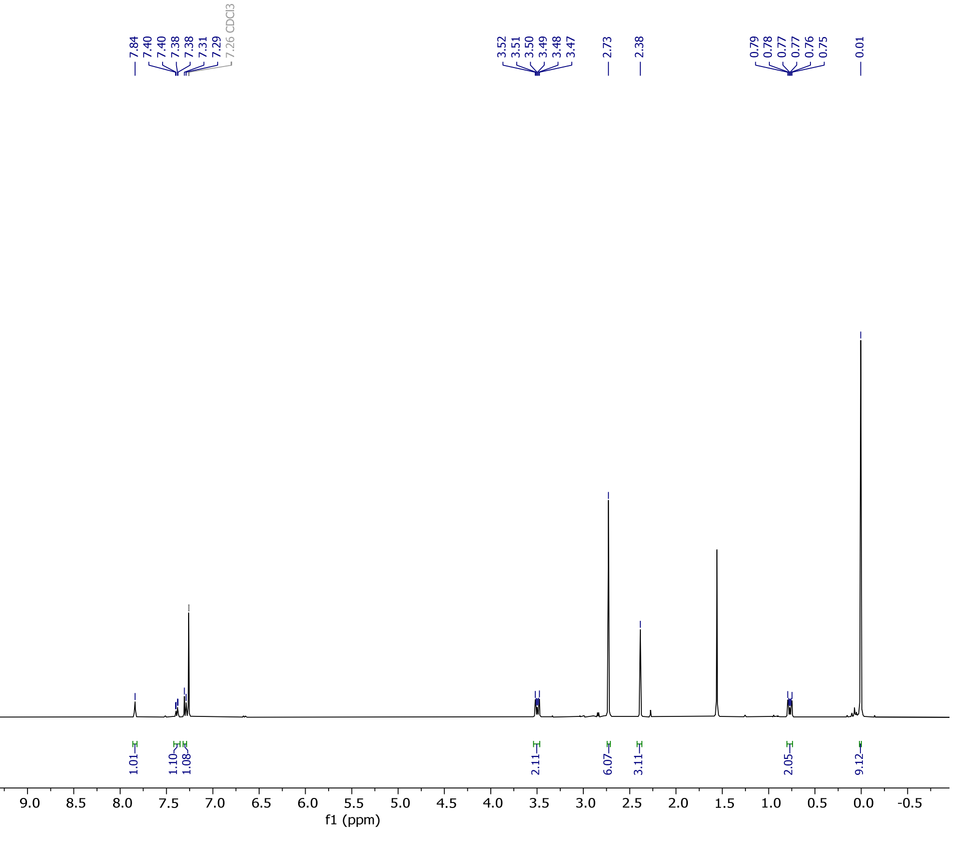

^{13}C NMR ($CDCl_3$; 101 MHz)

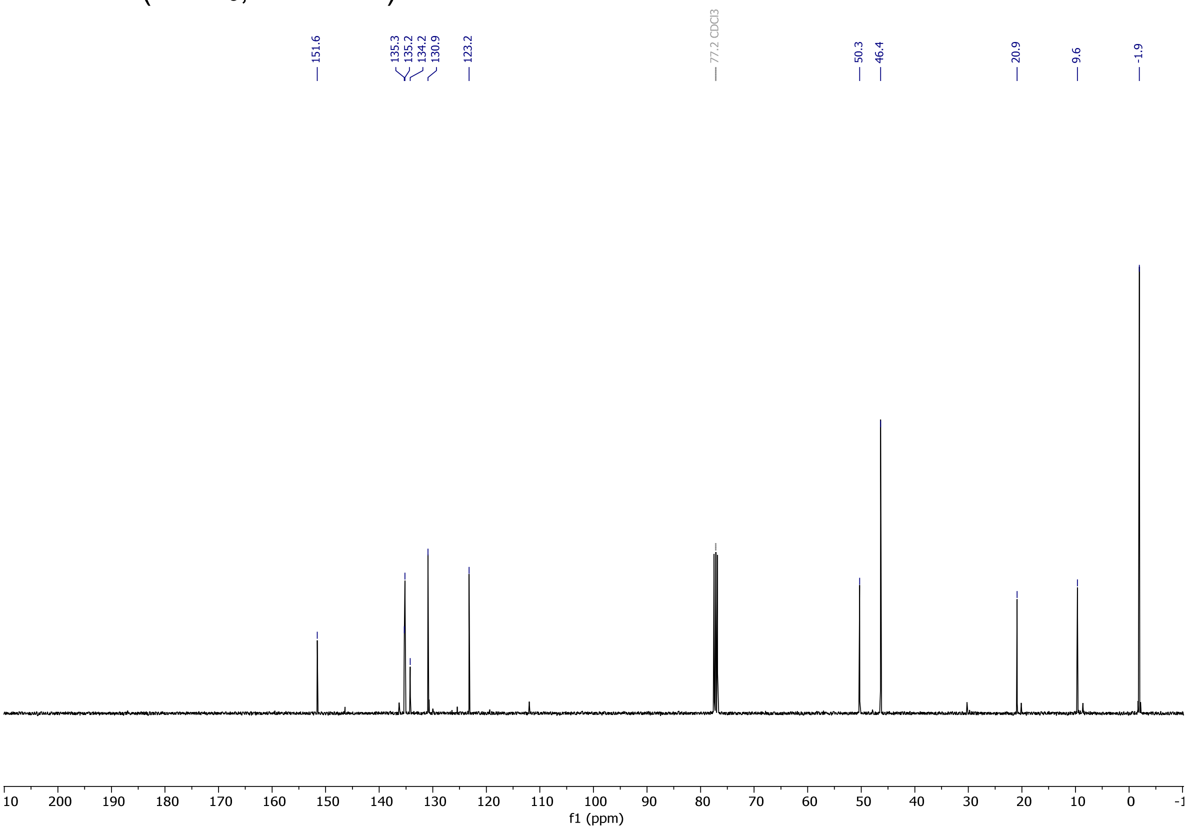

^{1}H NMR ($CDCl_3$, 400 MHz)

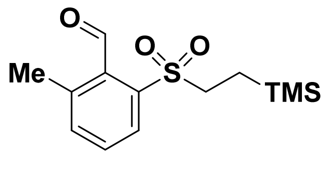

S15

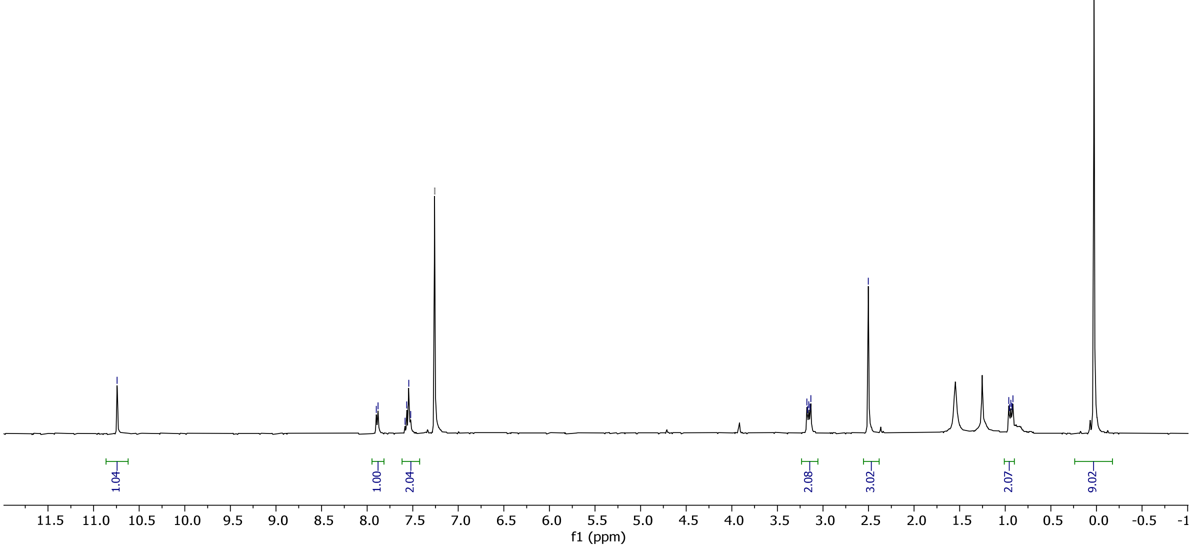

^{13}C NMR ($CDCl_3$; 101 MHz)

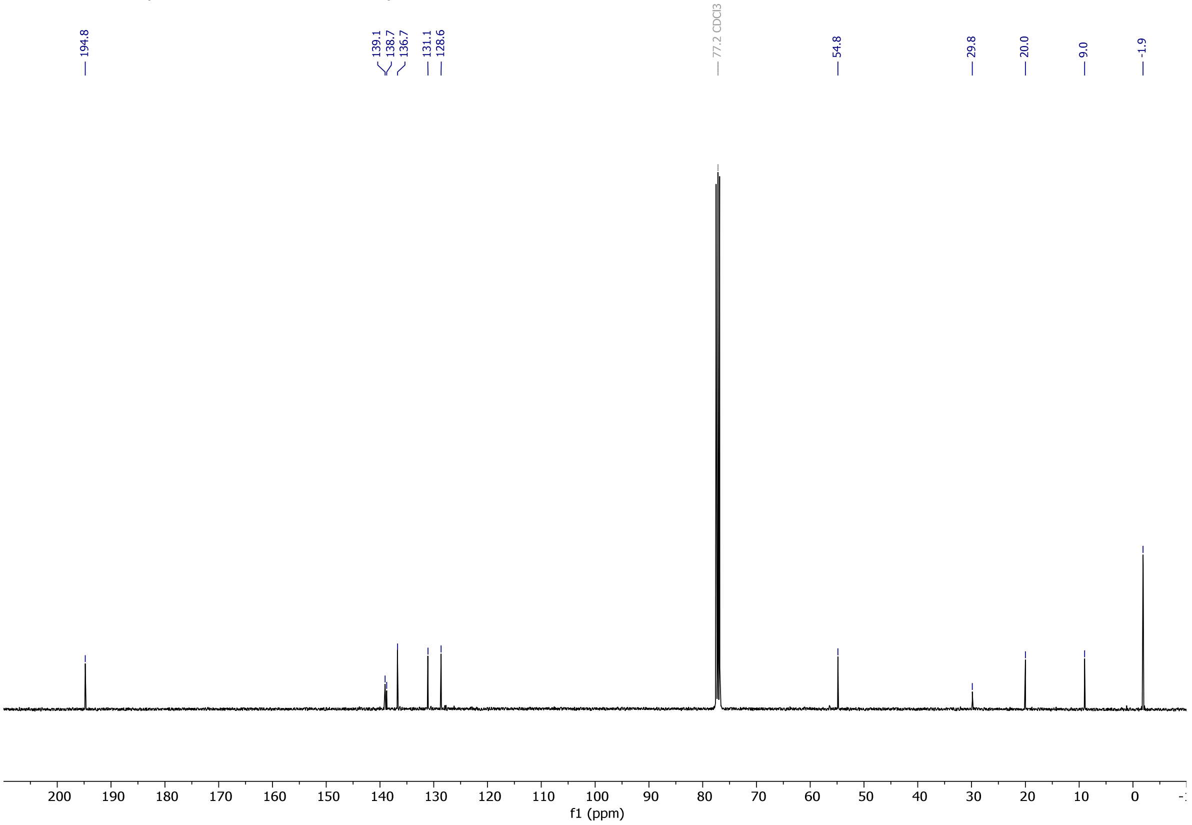

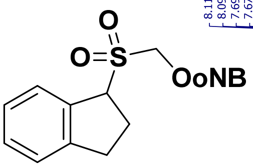

S16a

^{1}H NMR ($CDCl_3$, 400 MHz)

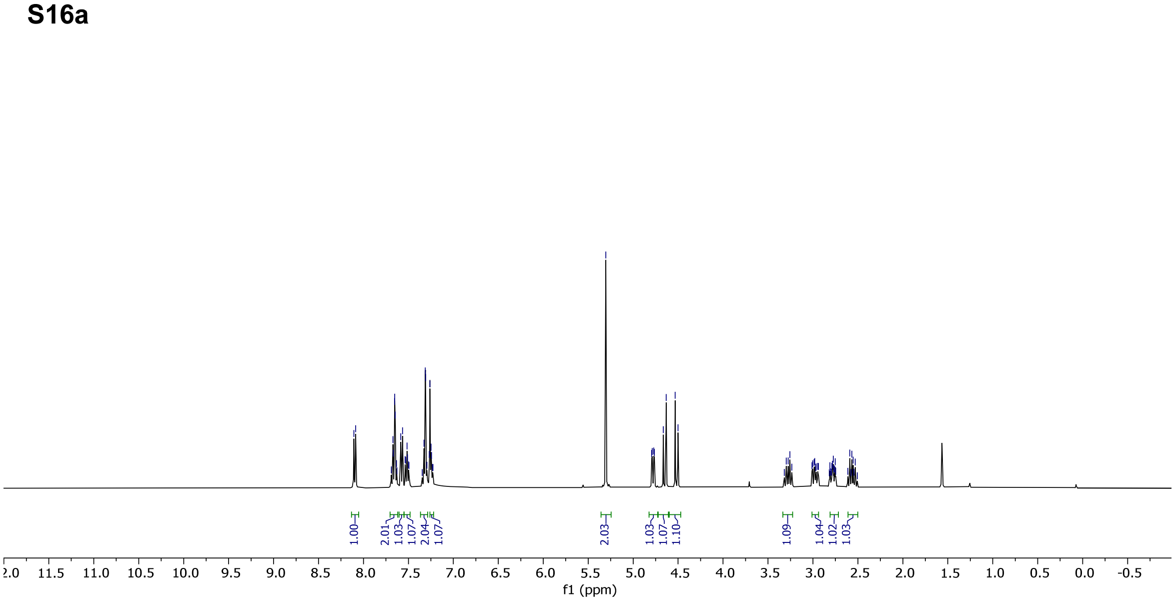

^{13}C NMR ($CDCl_3$; 101 MHz)

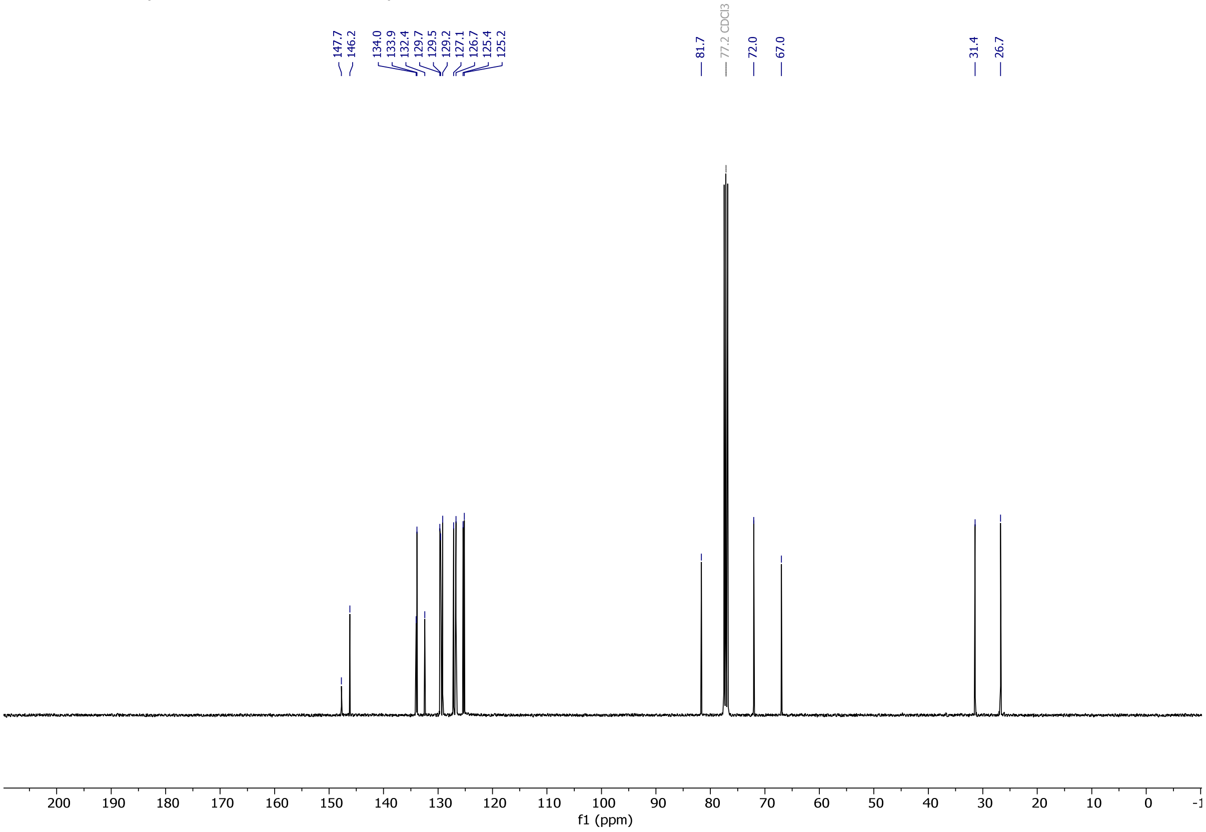

^{1}H NMR ($CDCl_3$, 400 MHz)

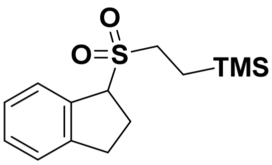

S16b

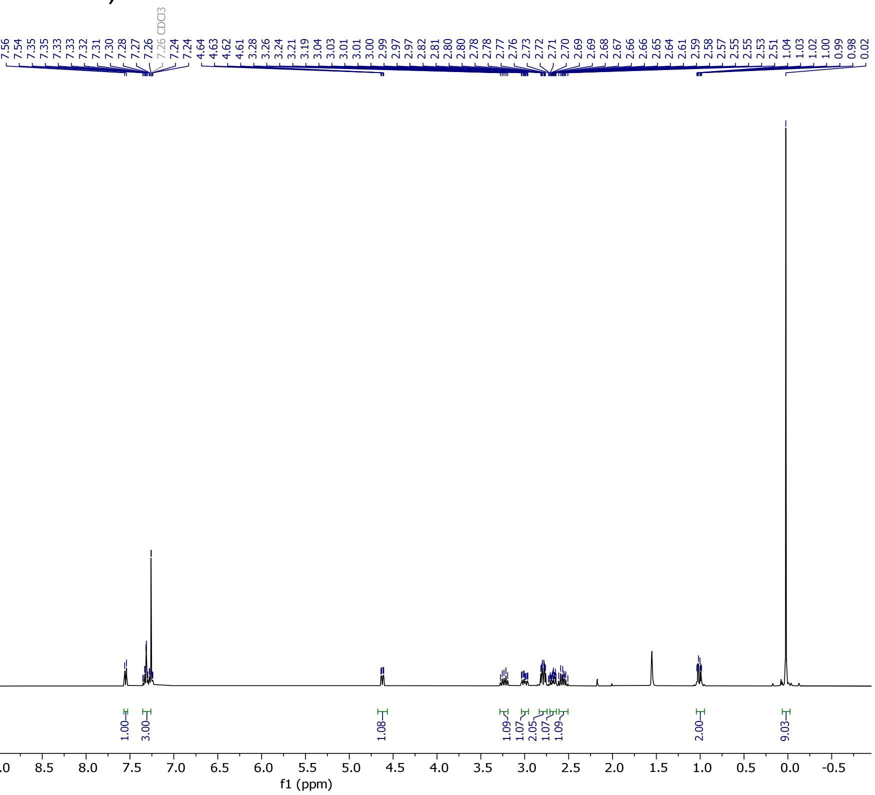

^{13}C NMR ($CDCl_3$; 101 MHz)

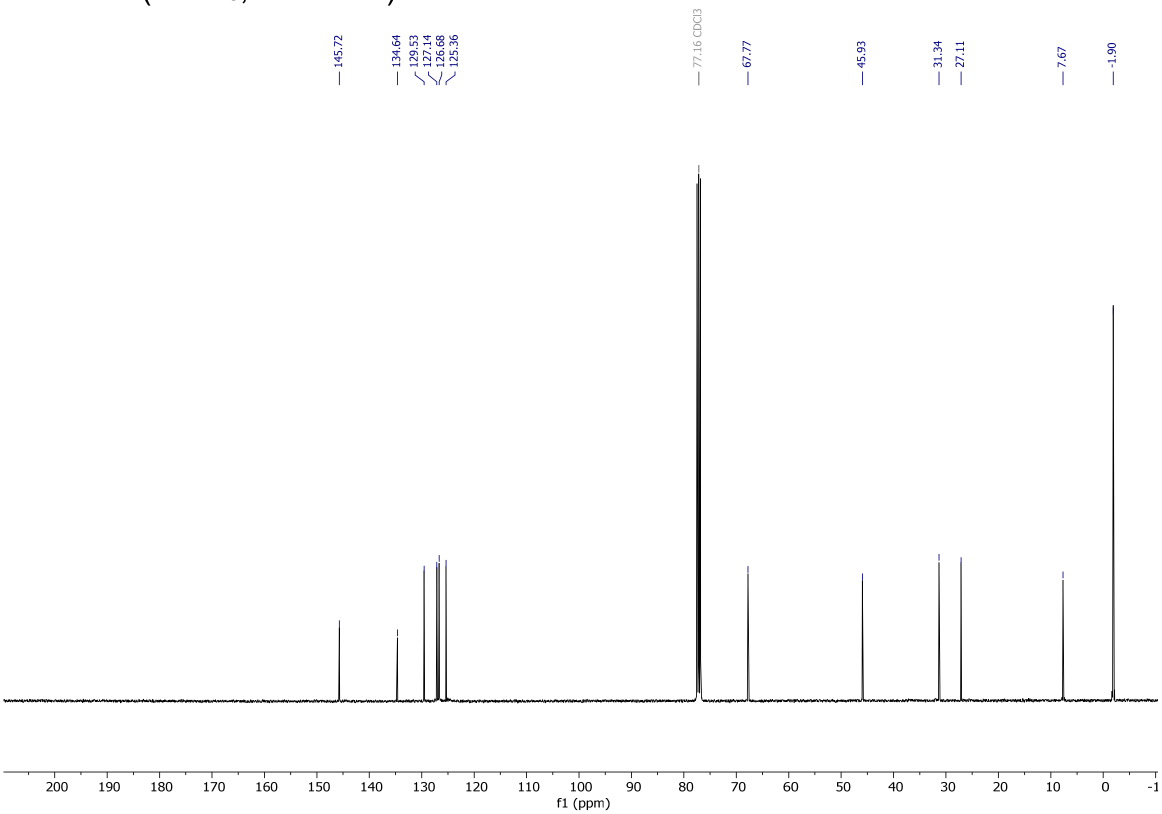

^{1}H NMR (DMSO-d_6, 400 MHz)

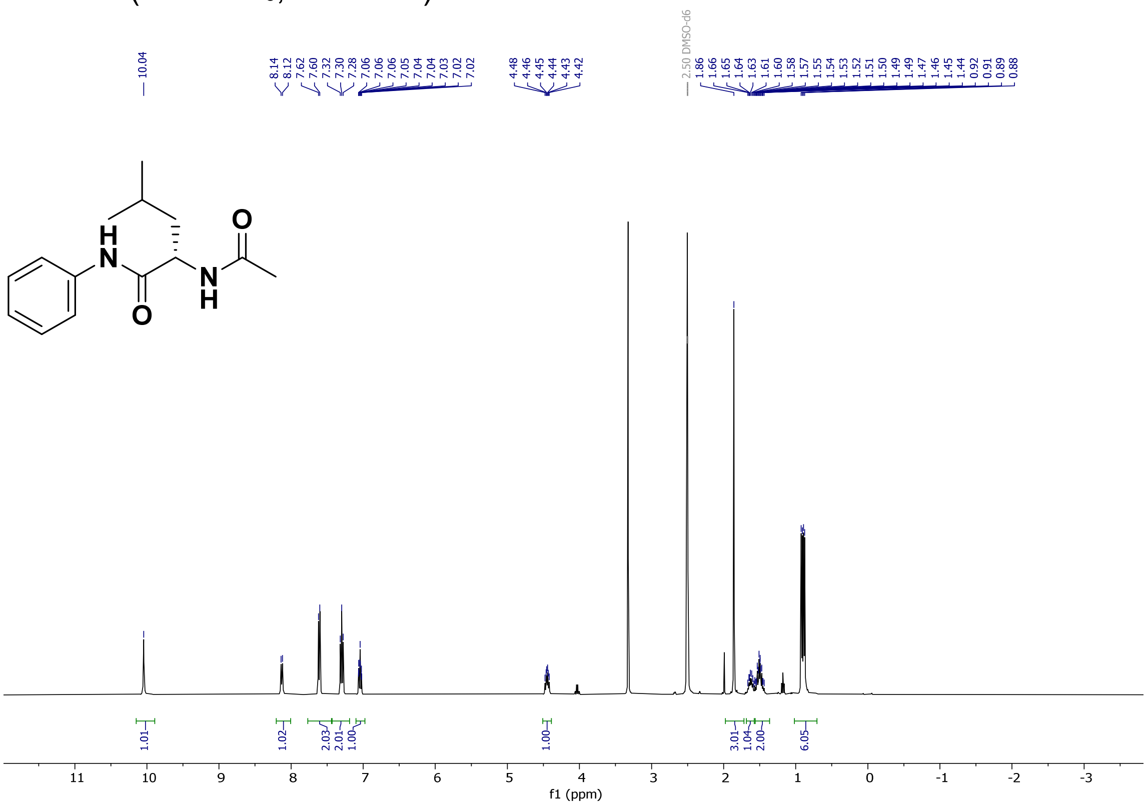

^{13}C NMR (DMSO-d_6; 101 MHz)

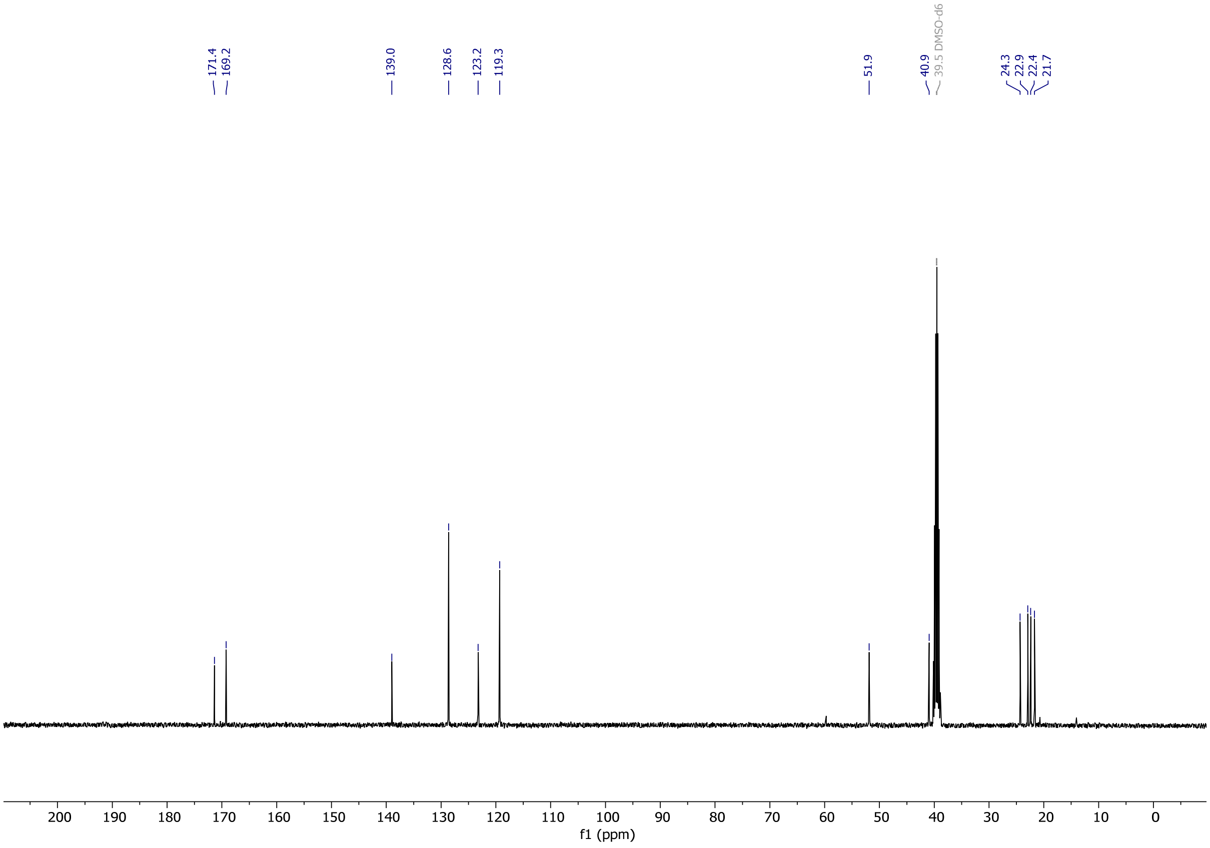

^1H NMR ($CDCl_3$, 400 MHz)

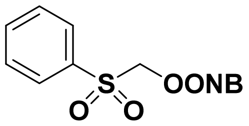

11a

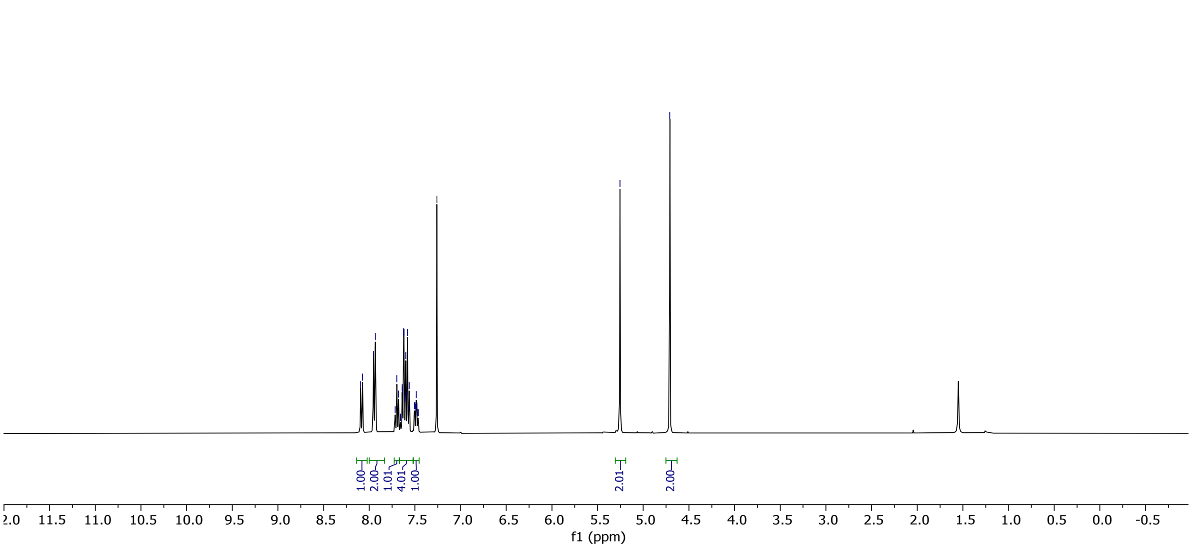

^{13}C NMR ($CDCl_3$; 101 MHz)

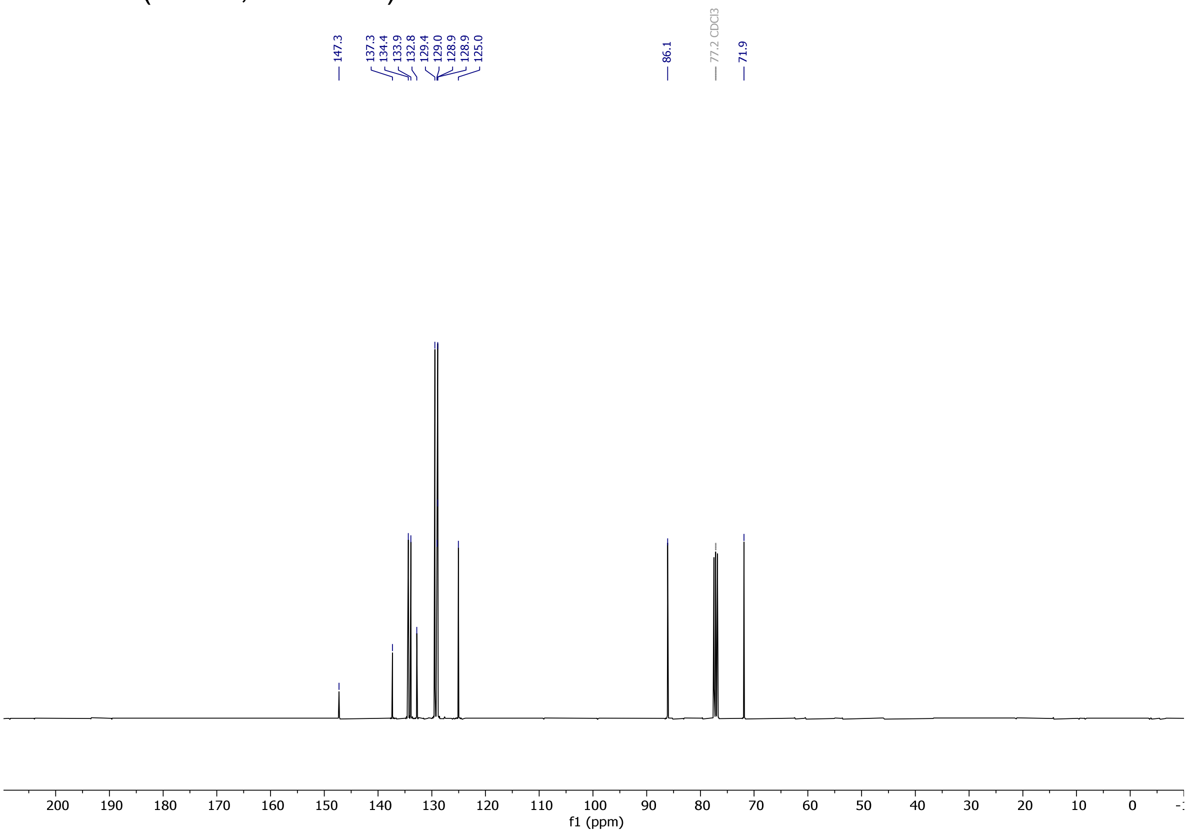

^{1}H NMR ($CDCl_3$, 400 MHz)

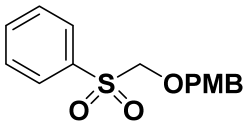

11b

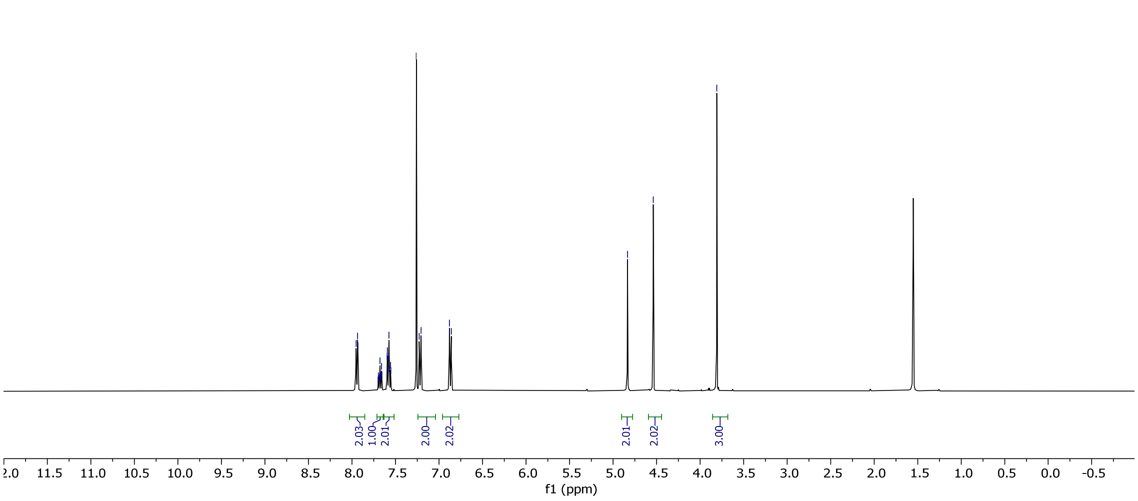

^{13}C NMR ($CDCl_3$; 101 MHz)

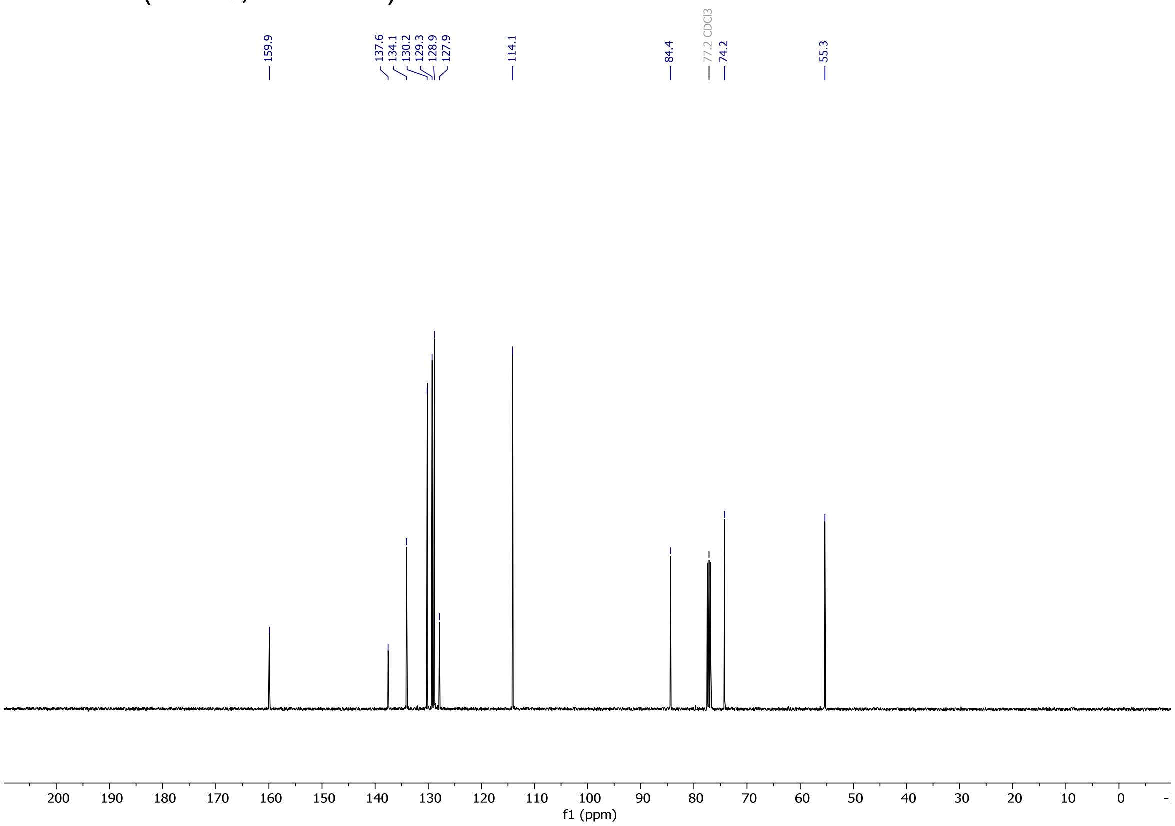

^{1}H NMR ($CDCl_3$, 400 MHz)

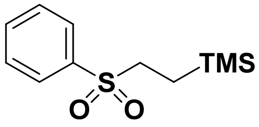

11c

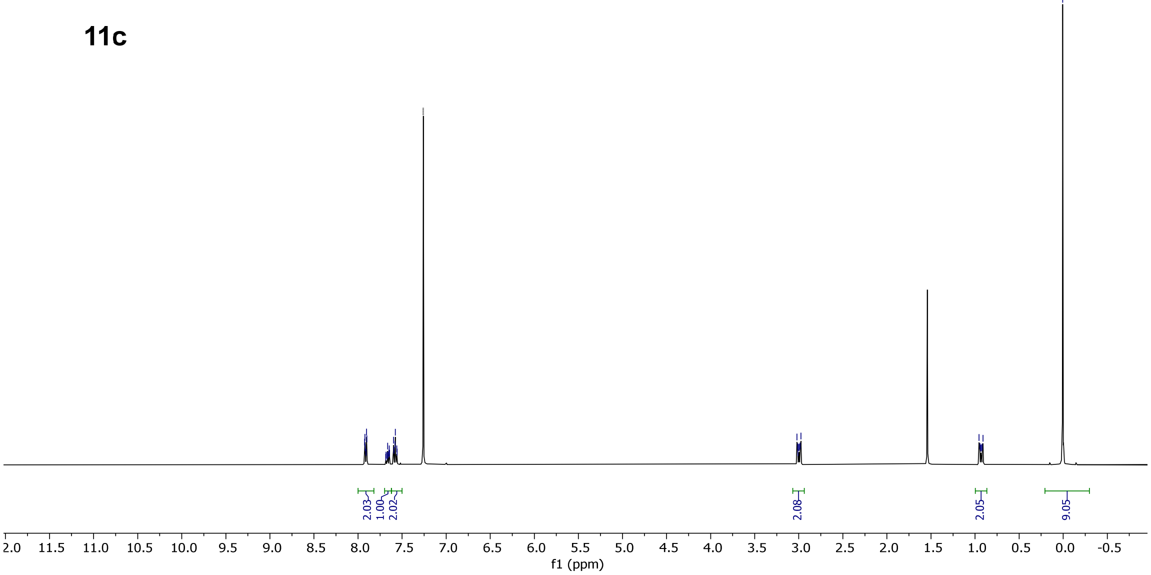

^{13}C NMR ($CDCl_3$; 101 MHz)

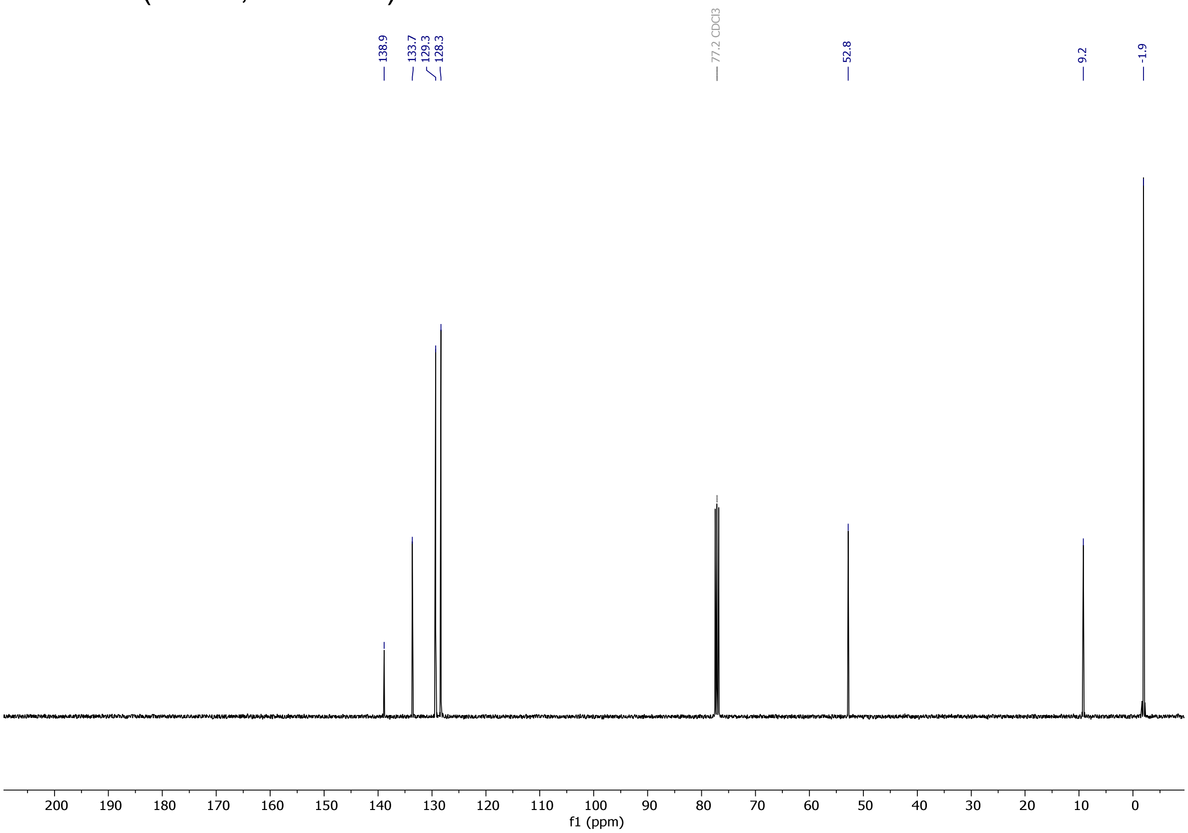

^{1}H NMR ($CDCl_3$, 400 MHz)

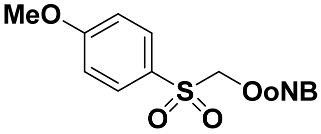

12a

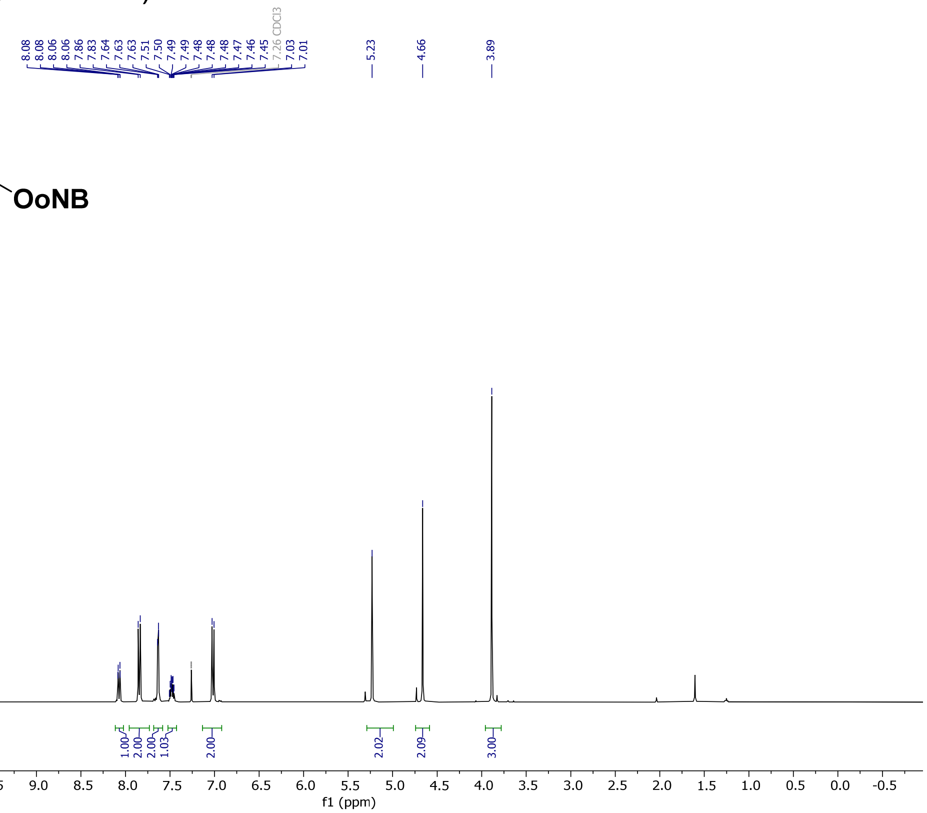

^{13}C NMR ($CDCl_3$; 101 MHz)

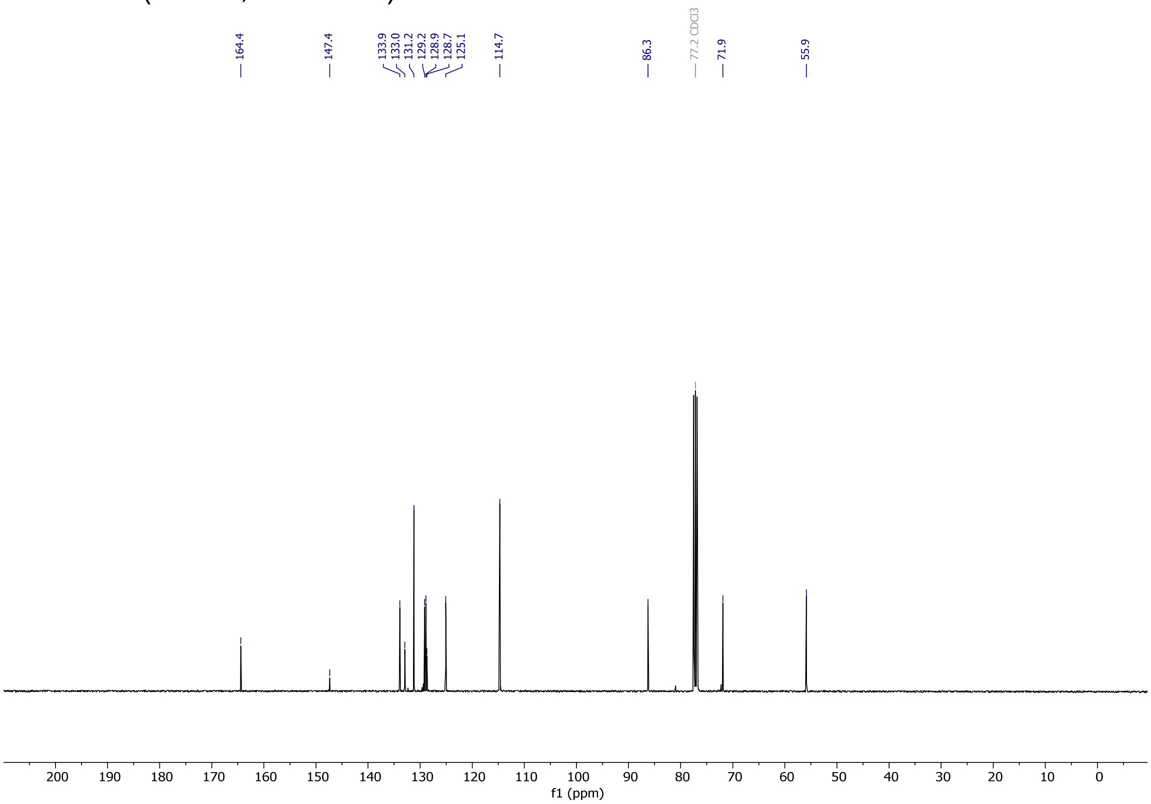

^{1}H NMR ($CDCl_3$, 400 MHz)

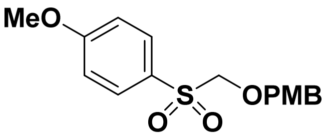

12b

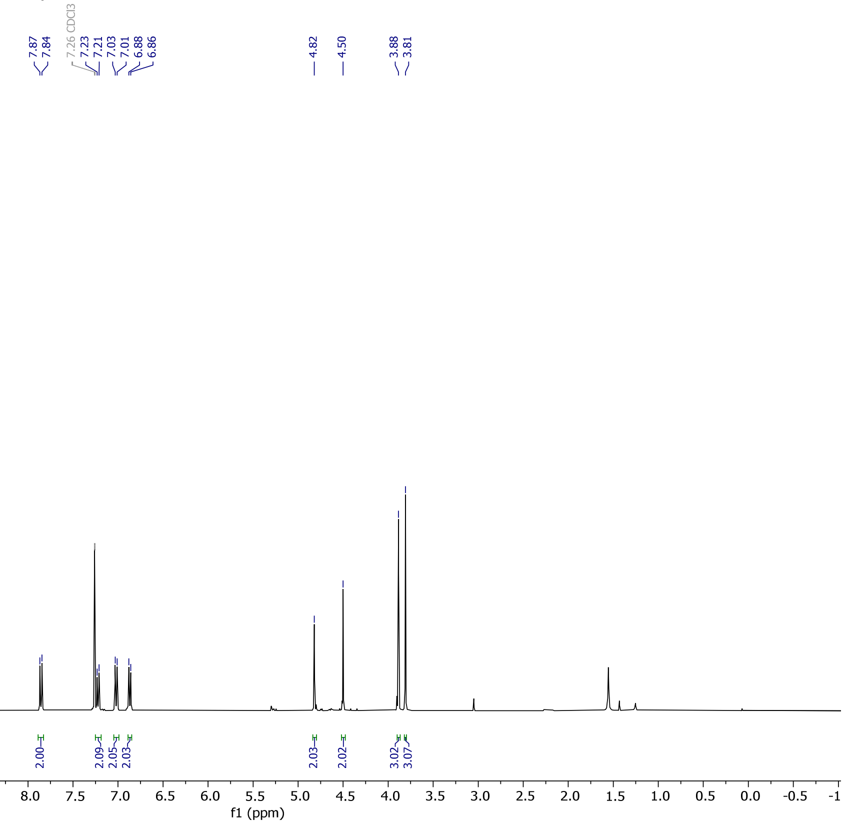

^{13}C NMR ($CDCl_3$; 101 MHz)

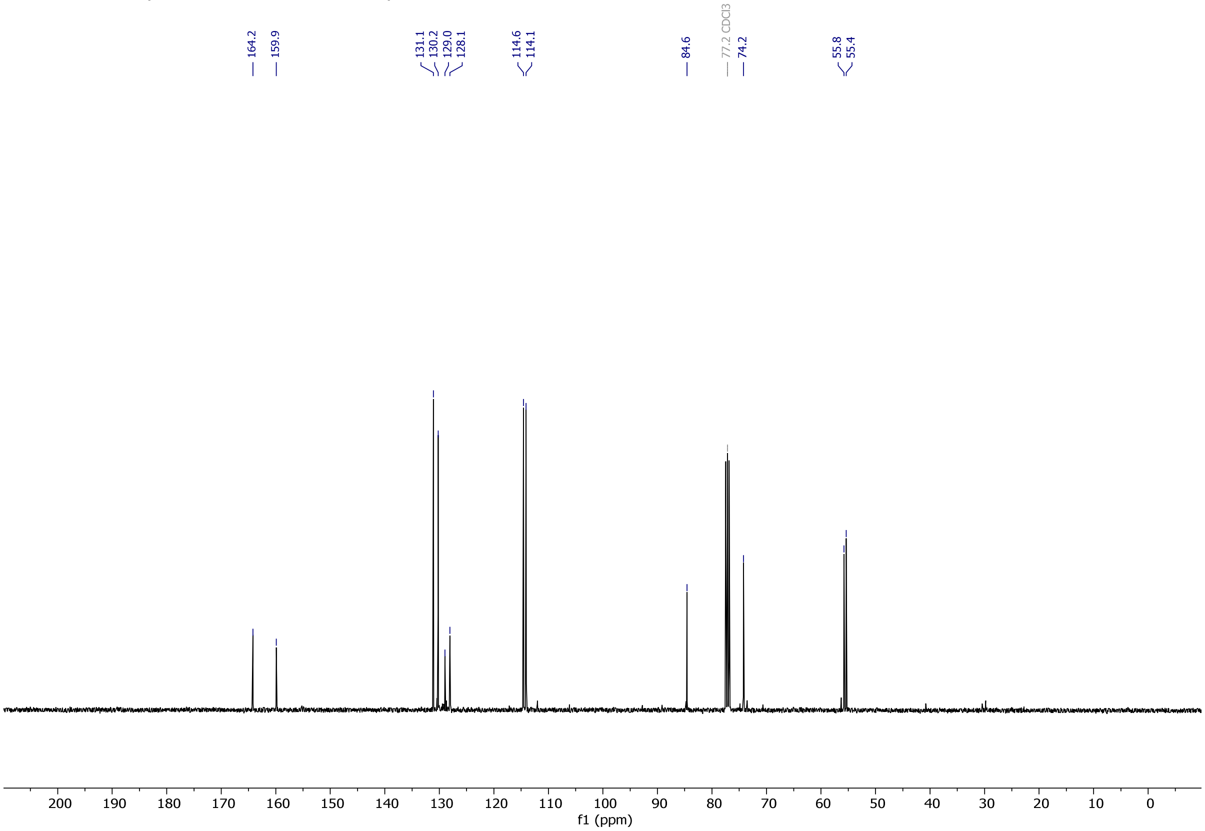

11a ^{1}H NMR ($CDCl_3$, 400 MHz)

12c

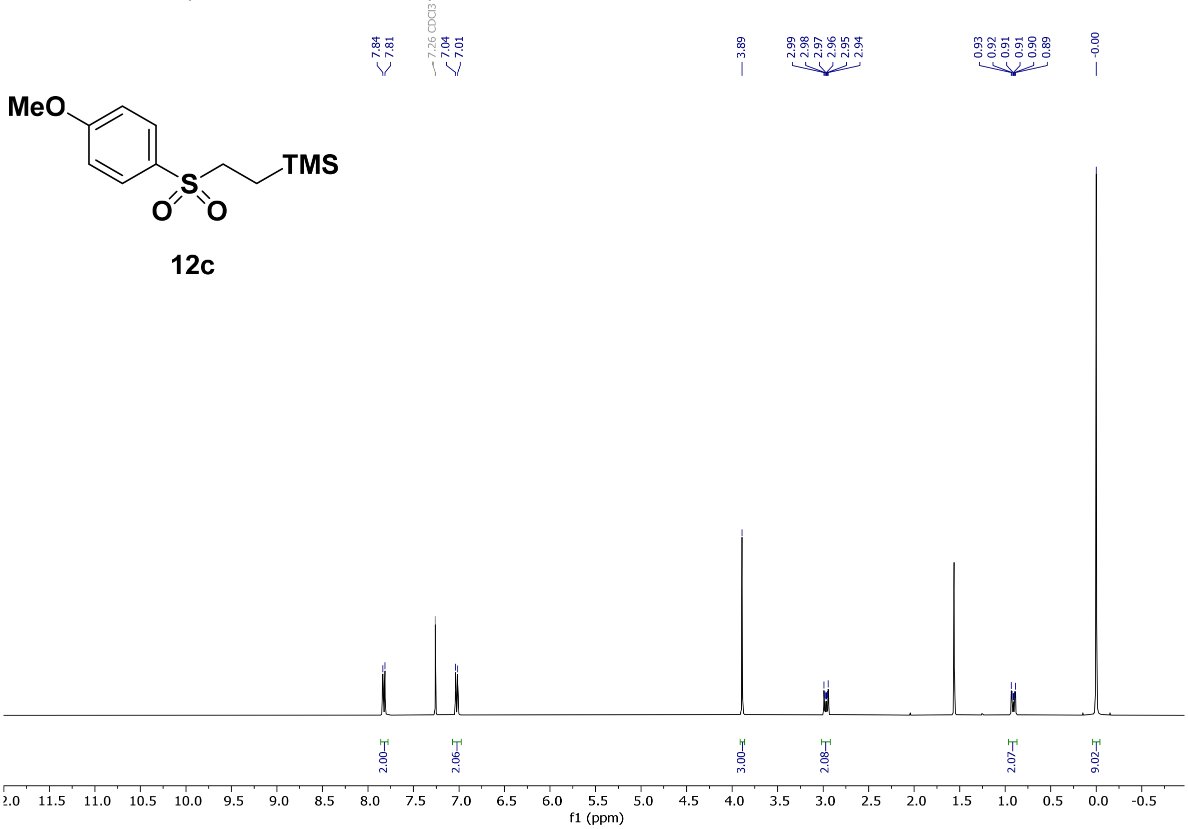

^{13}C NMR ($CDCl_3$; 101 MHz)

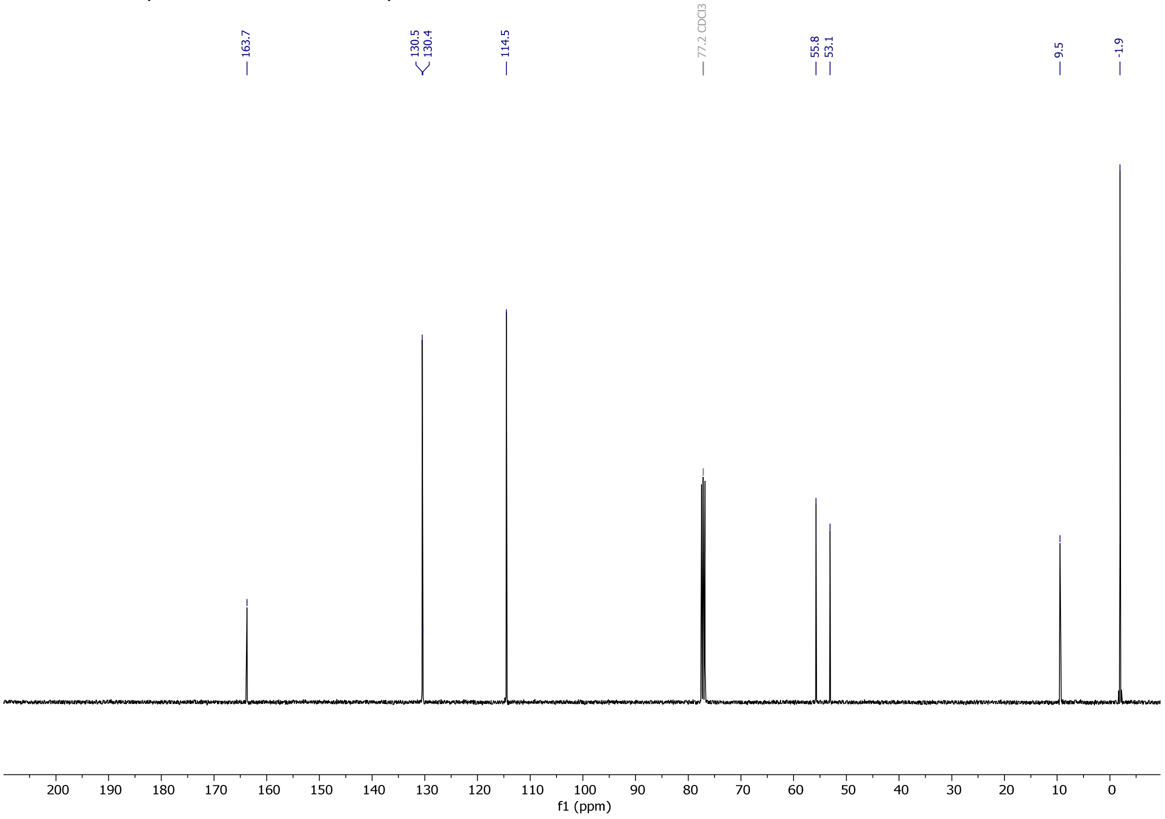

^{1}H NMR ($CDCl_3$, 400 MHz)

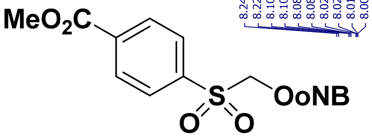

13a

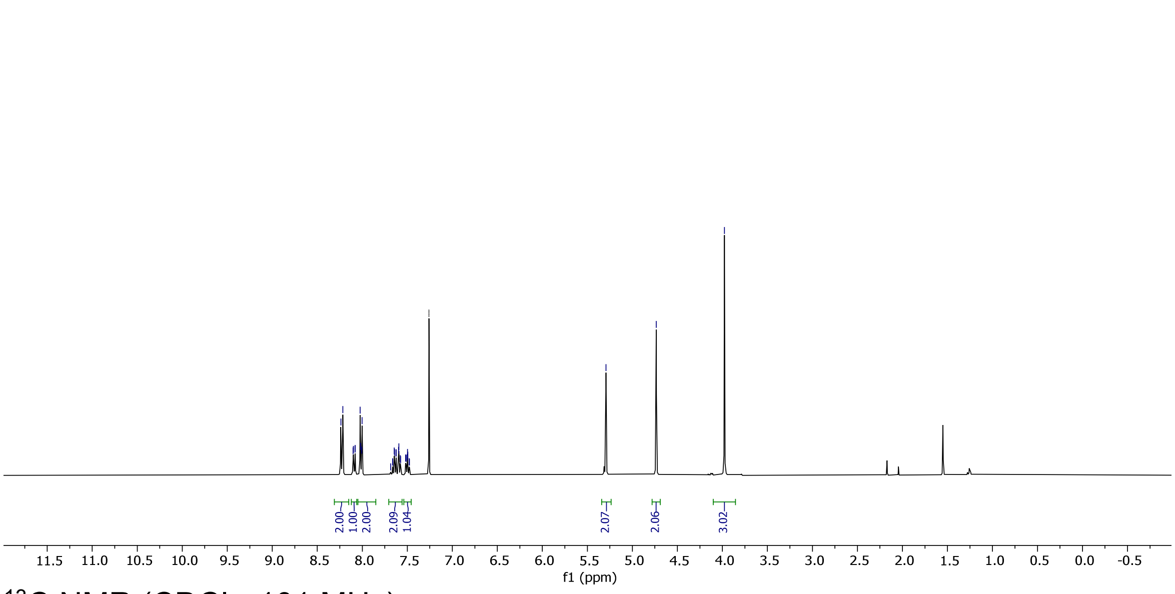

^{13}C NMR ($CDCl_3$; 101 MHz)

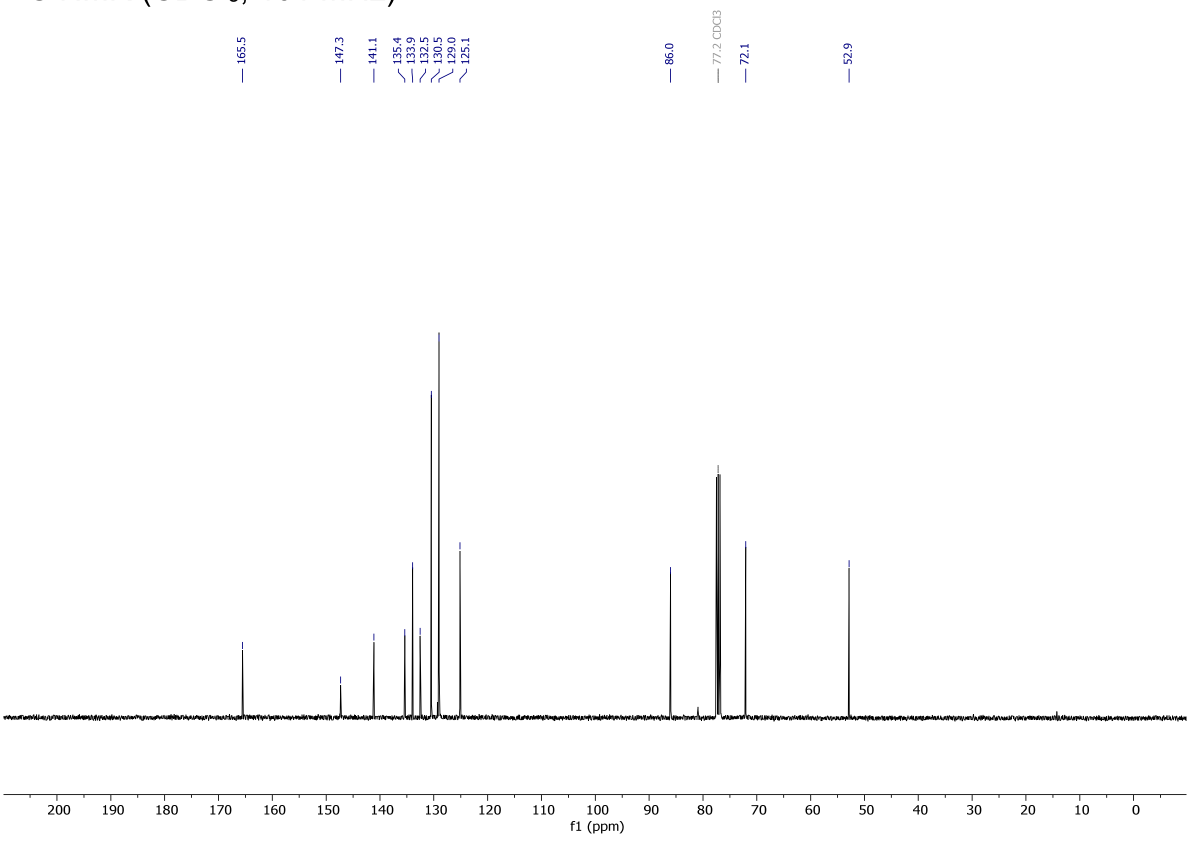

^1H NMR ($CDCl_3$, 400 MHz)

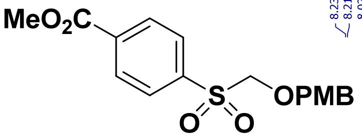

13b

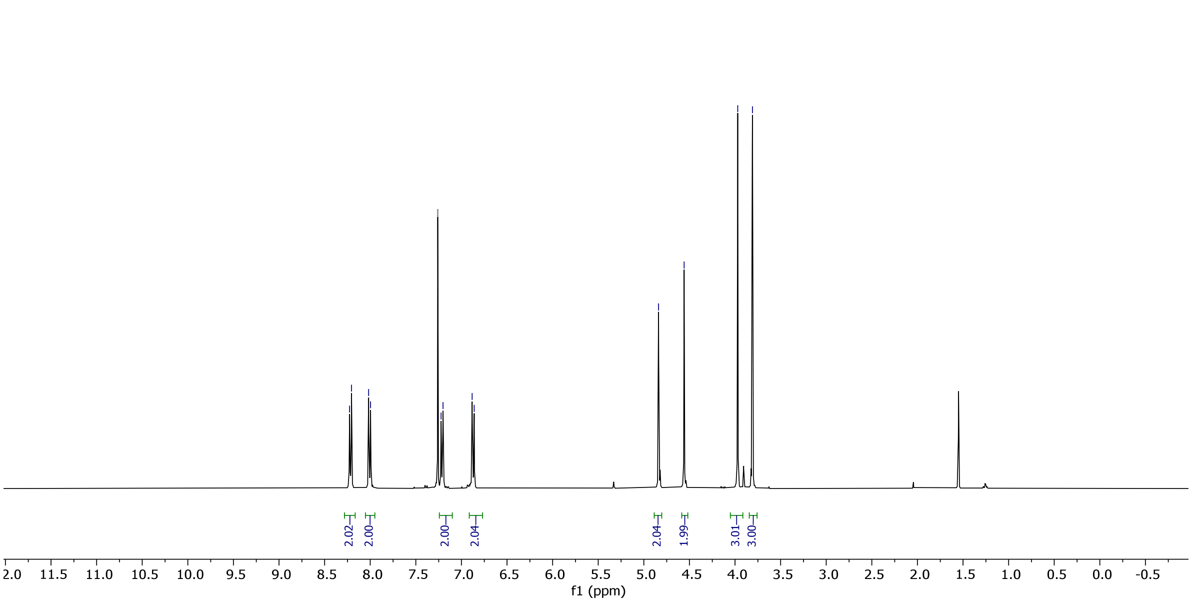

^{13}C NMR ($CDCl_3$; 101 MHz)

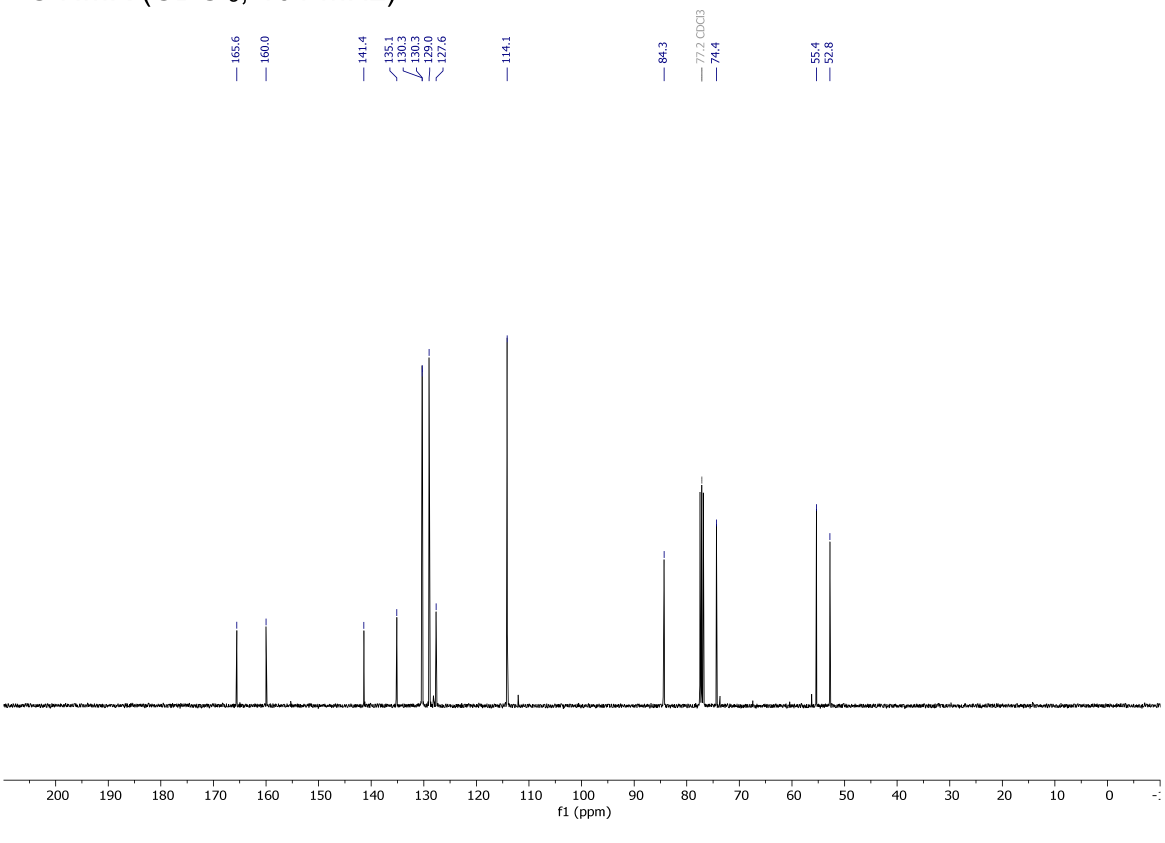

^{1}H NMR (CDCl$_3$, 400 MHz)

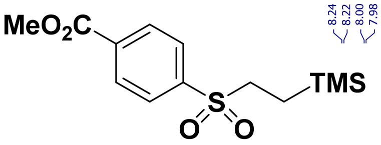

13c

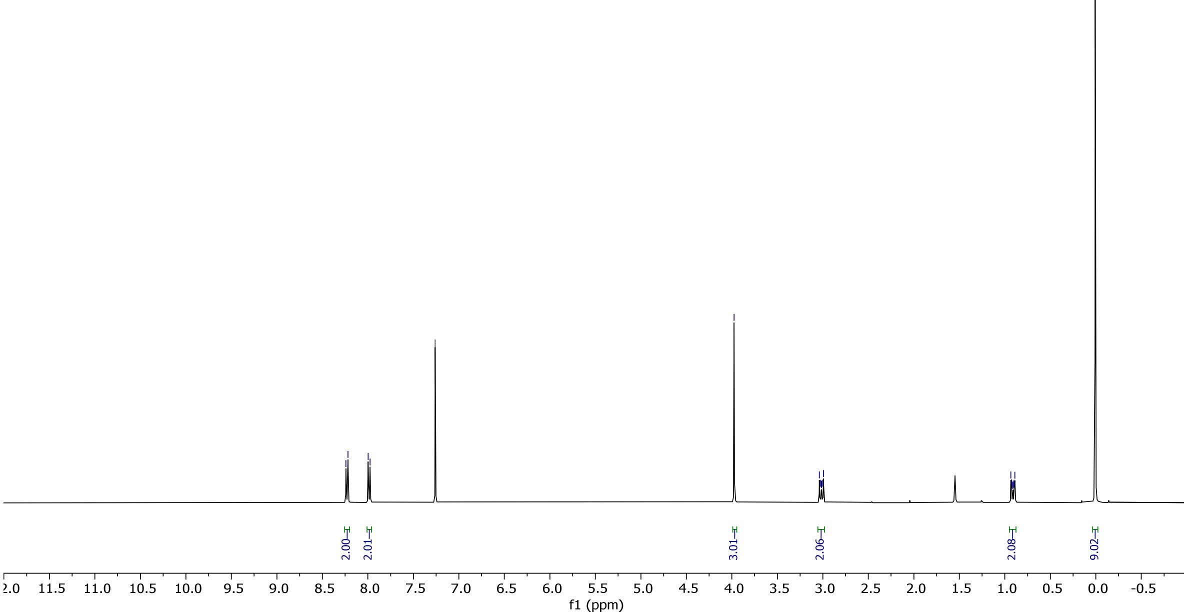

^{13}C NMR (CDCl$_3$; 101 MHz)

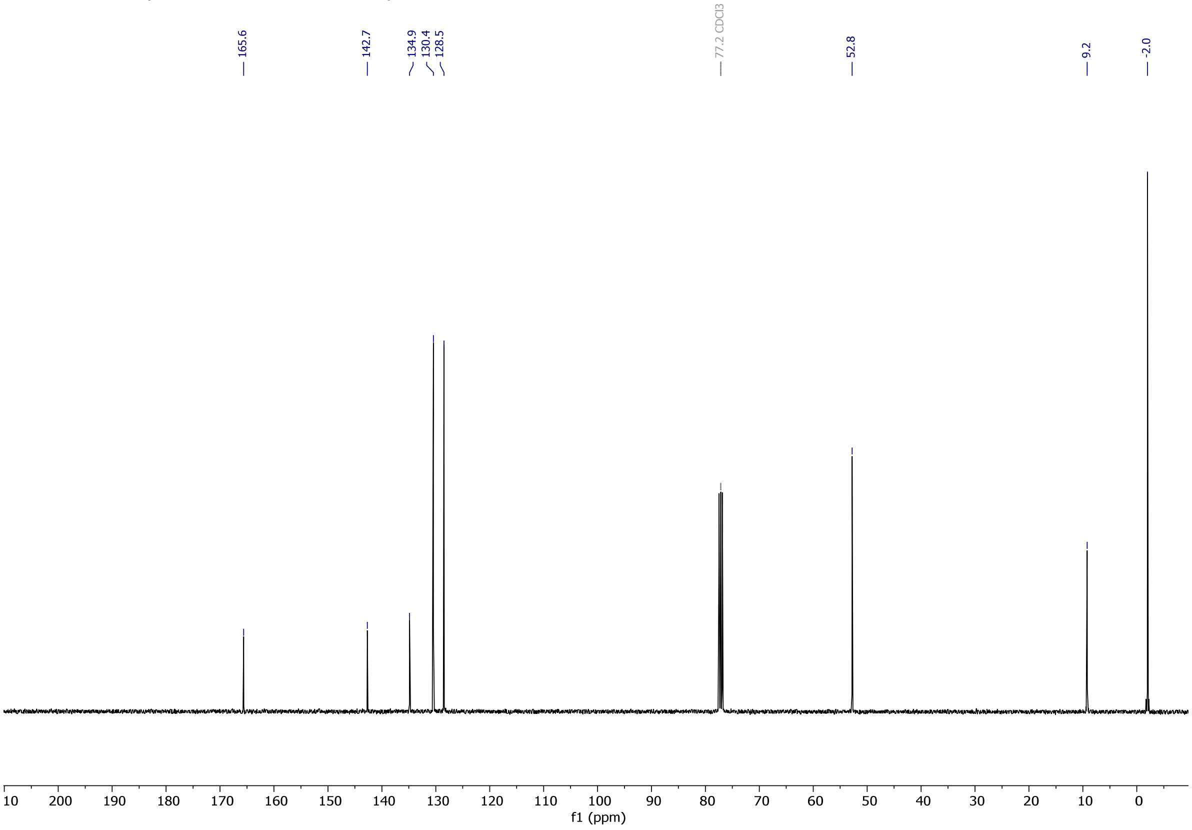

^{1}H NMR ($CDCl_3$, 400 MHz)

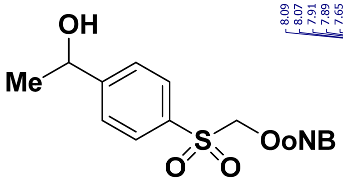

14a

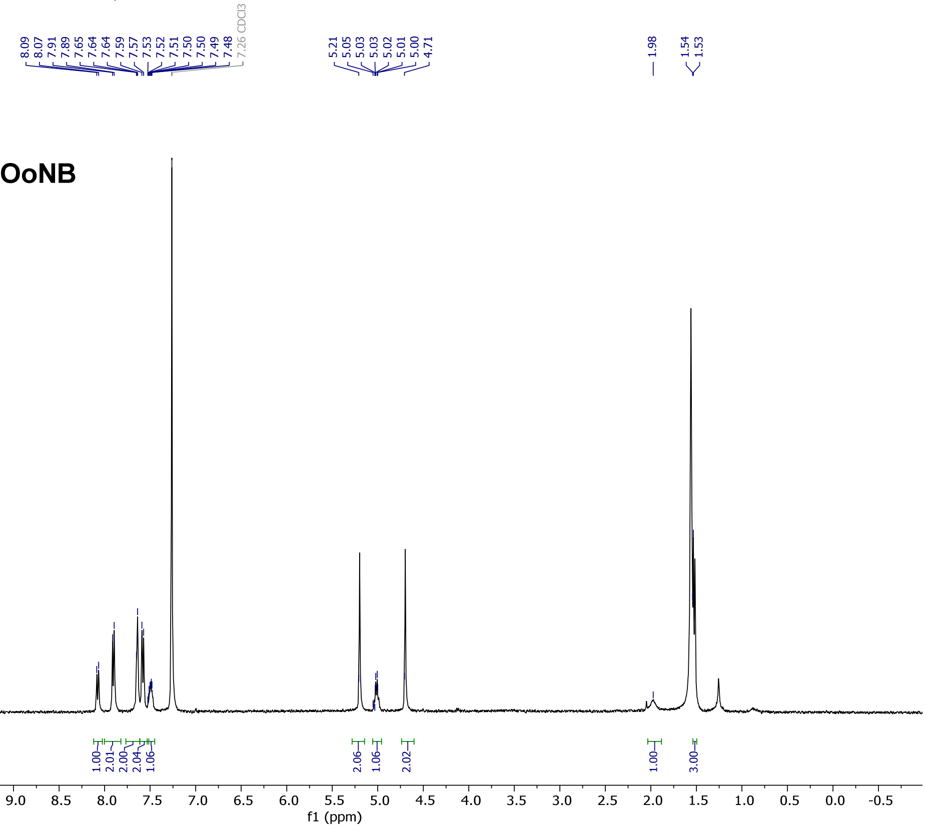

^{13}C NMR ($CDCl_3$; 101 MHz)

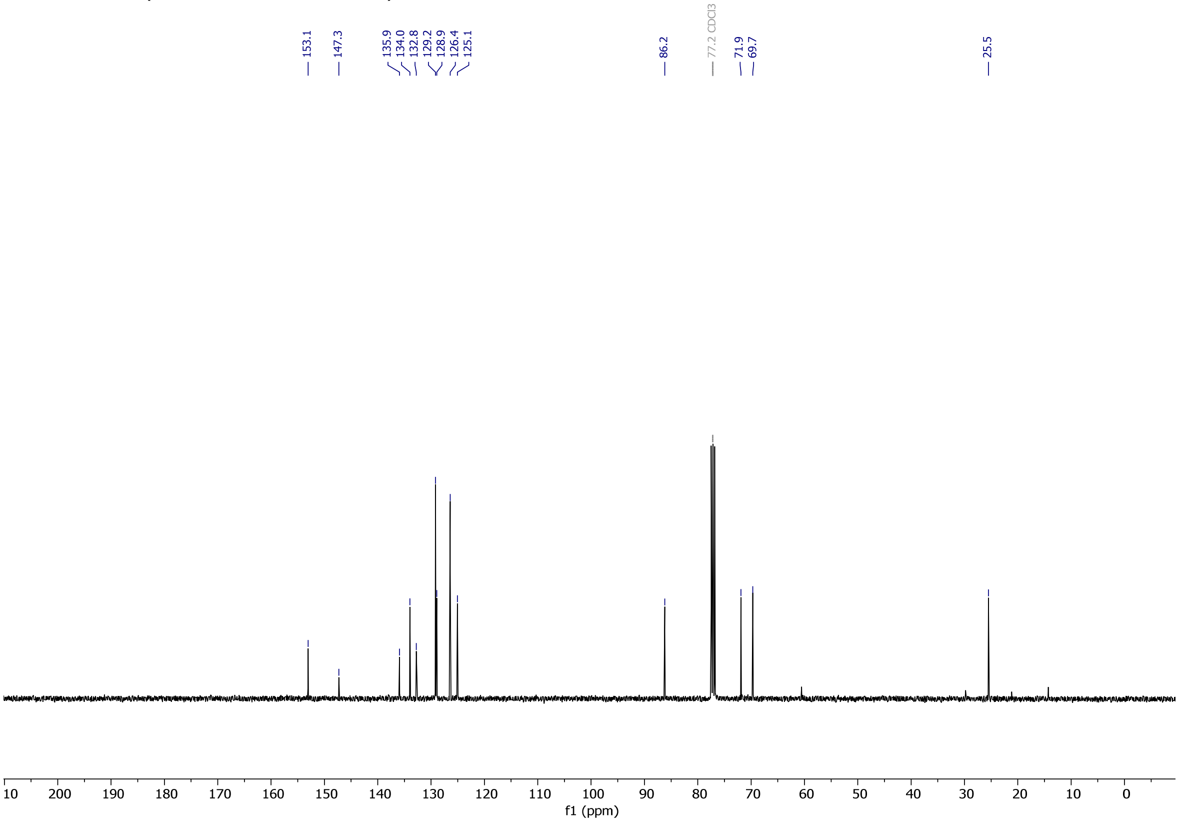

^{1}H NMR ($CDCl_3$, 400 MHz)

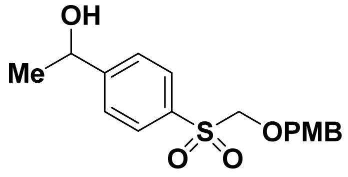

14b

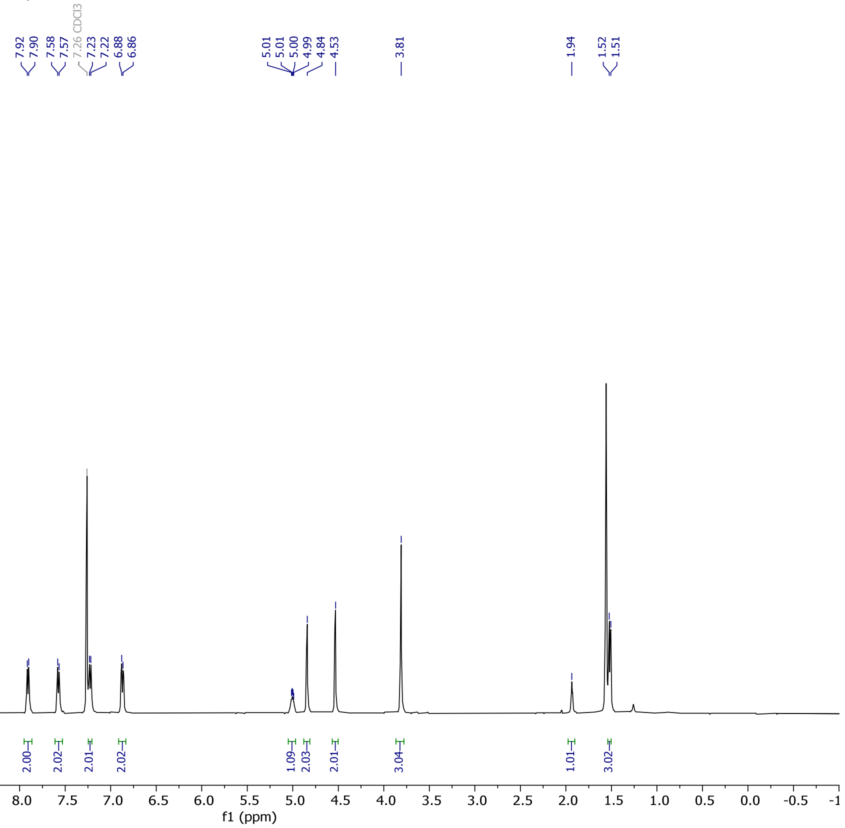

^{13}C NMR ($CDCl_3$; 101 MHz)

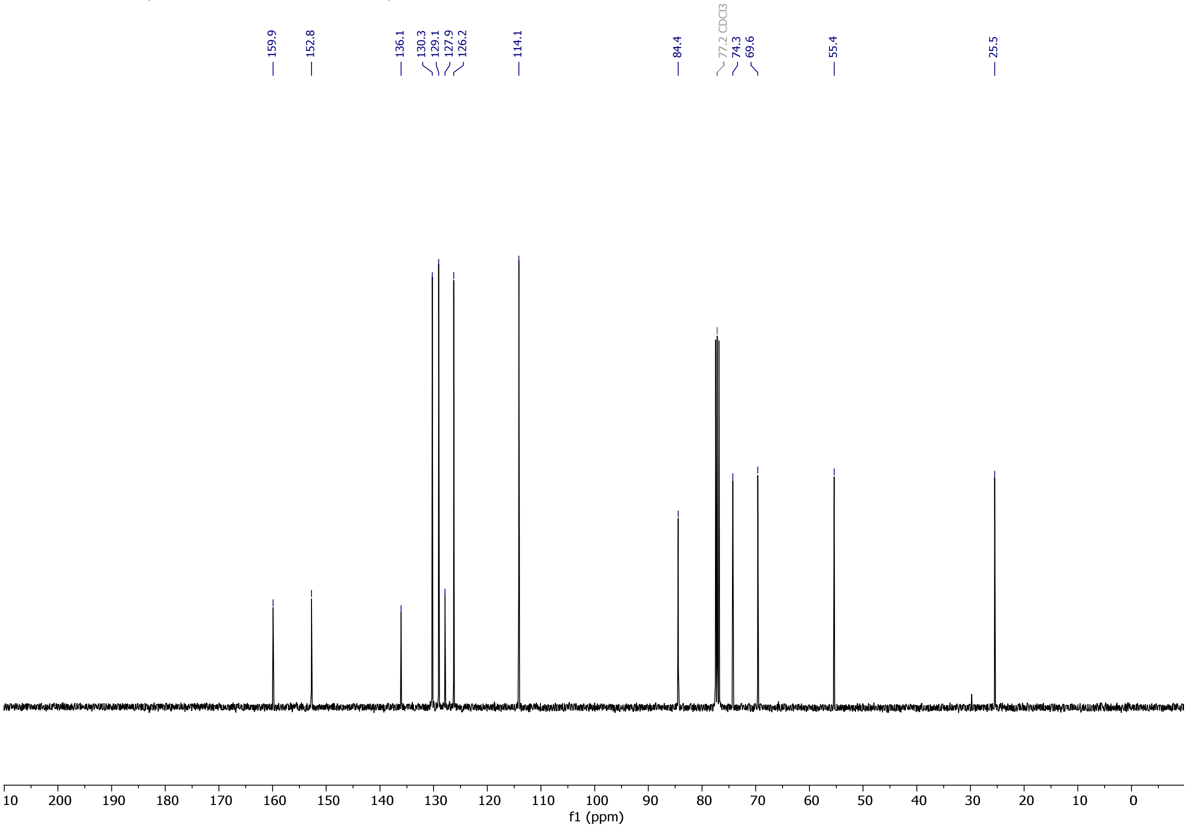

^{1}H NMR ($CDCl_3$, 400 MHz)

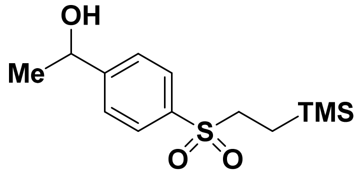

14c

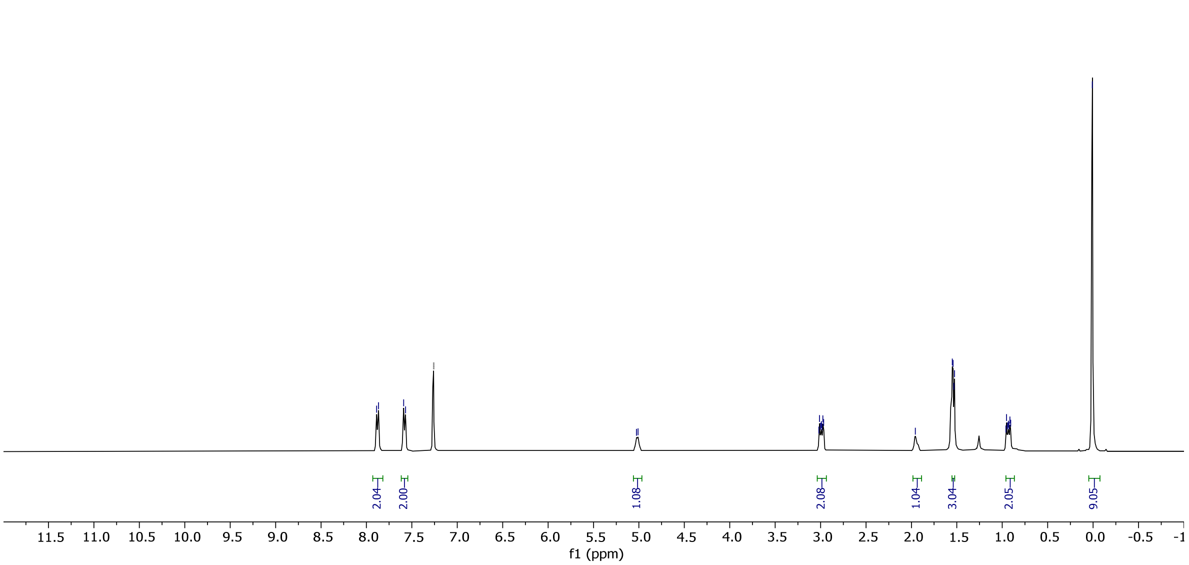

^{13}C NMR ($CDCl_3$; 101 MHz)

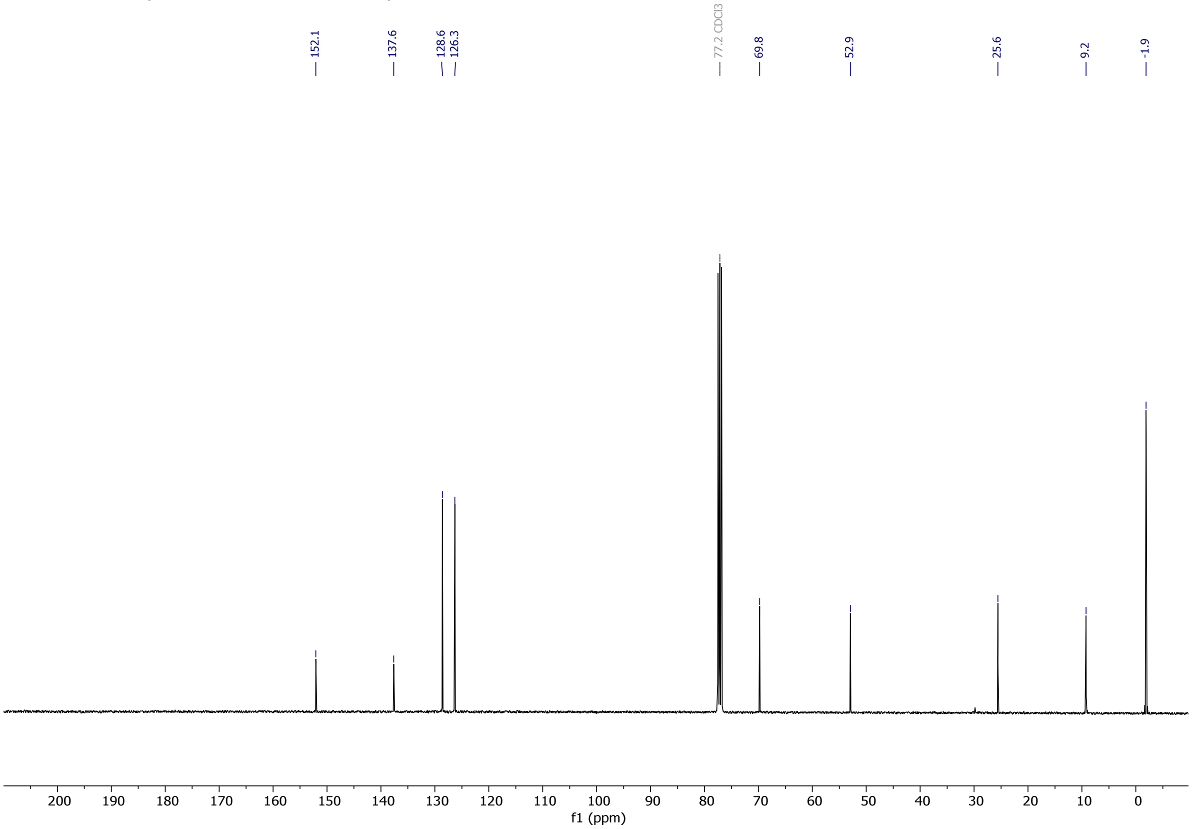

^{1}H NMR ($CDCl_3$, 400 MHz)

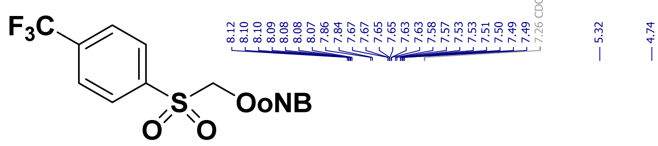

15a

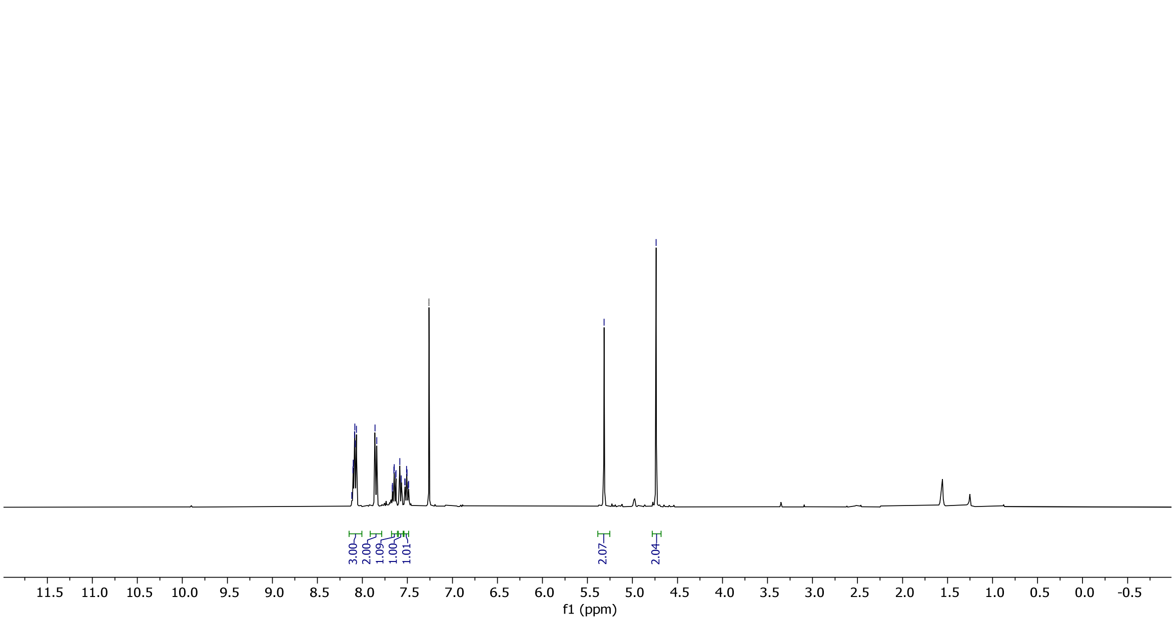

^{13}C NMR ($CDCl_3$; 101 MHz)

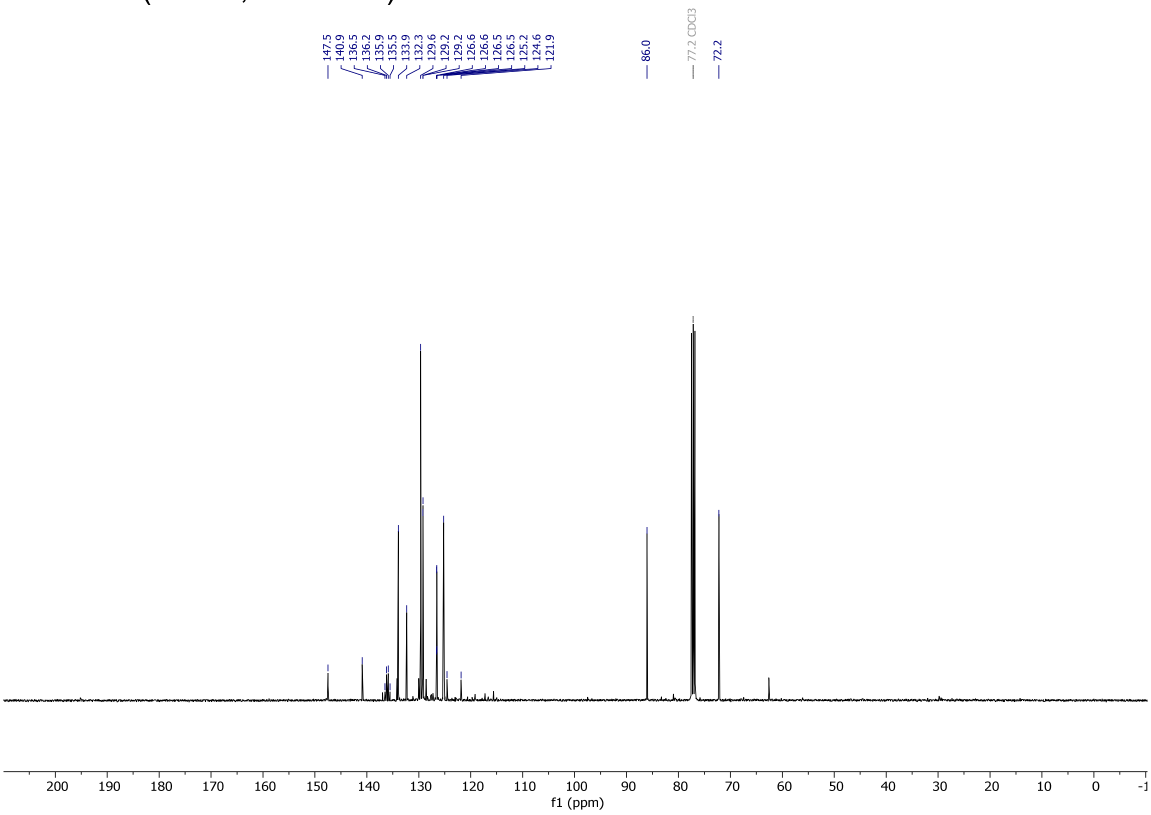

^{19}F NMR ($CDCl_3$; 376 MHz)

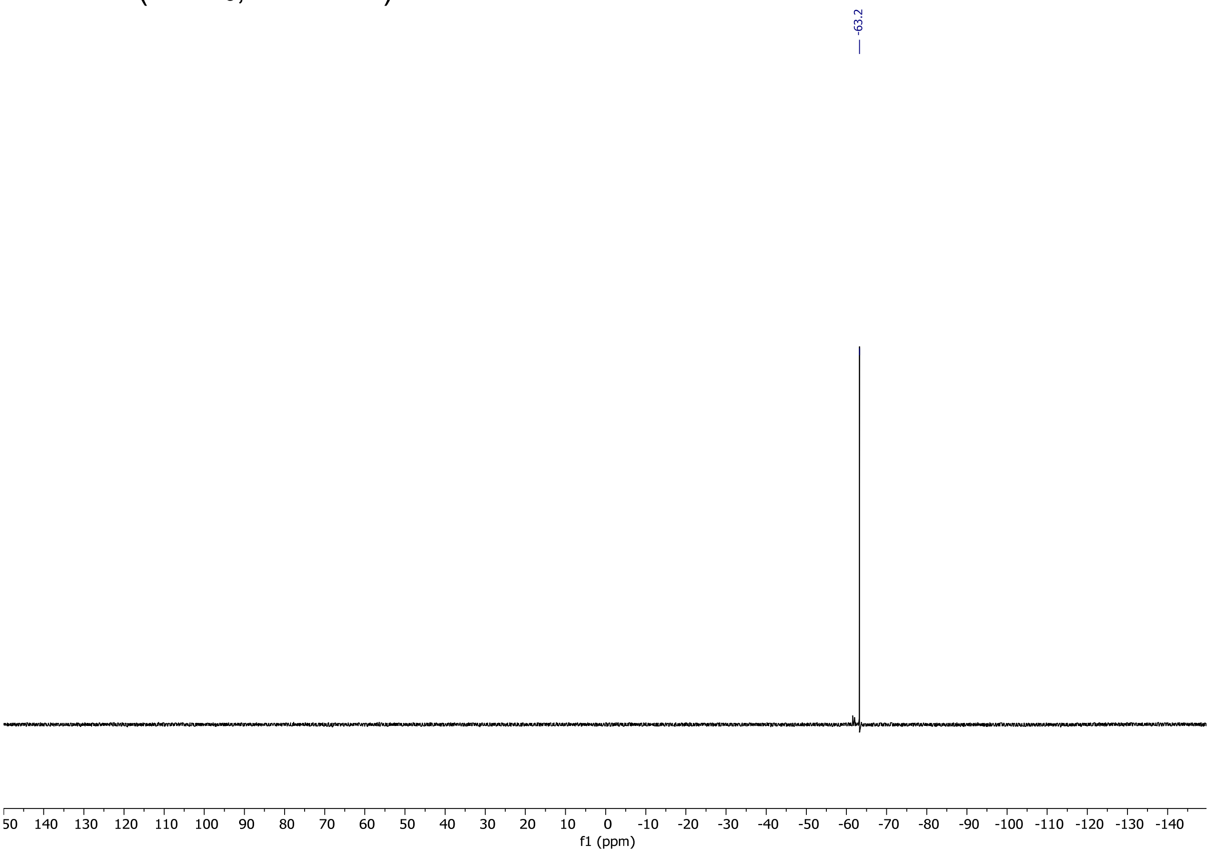

^1H NMR ($CDCl_3$, 400 MHz)

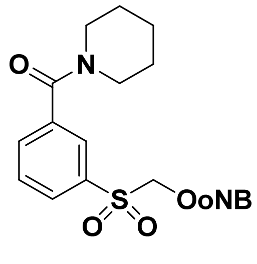

16a

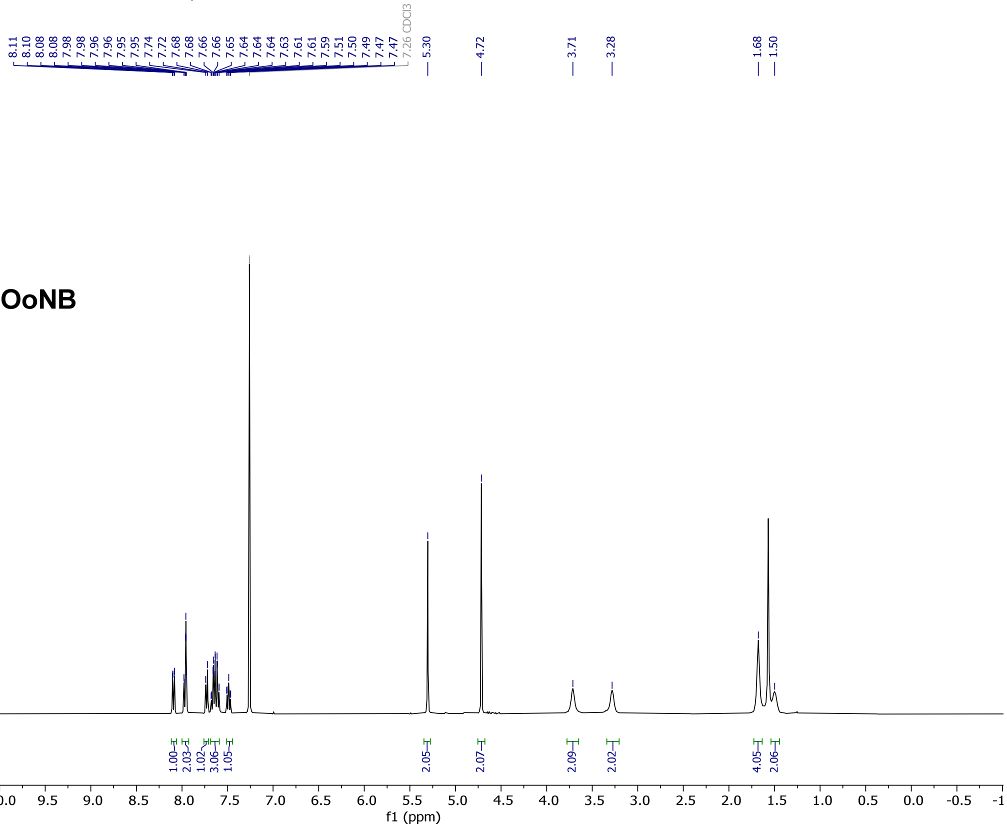

^{13}C NMR ($CDCl_3$; 101 MHz)

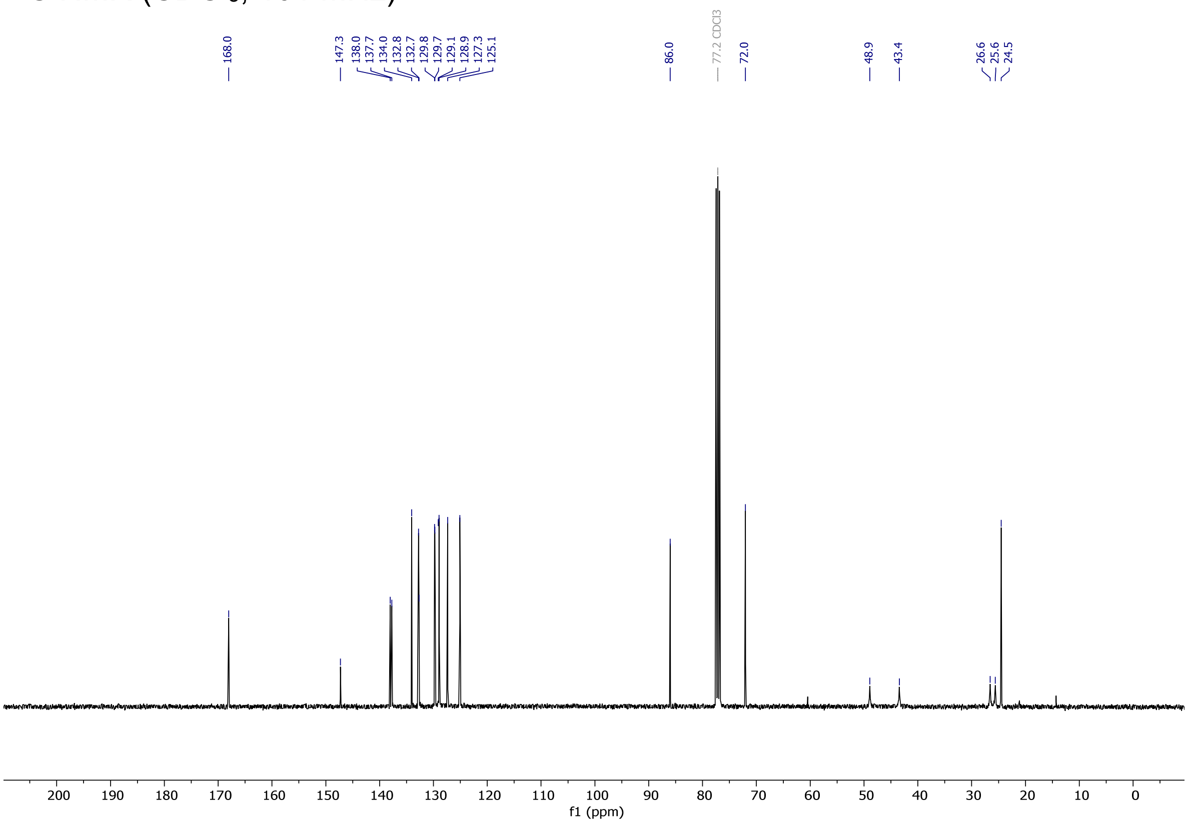

^{1}H NMR ($CDCl_3$, 400 MHz)

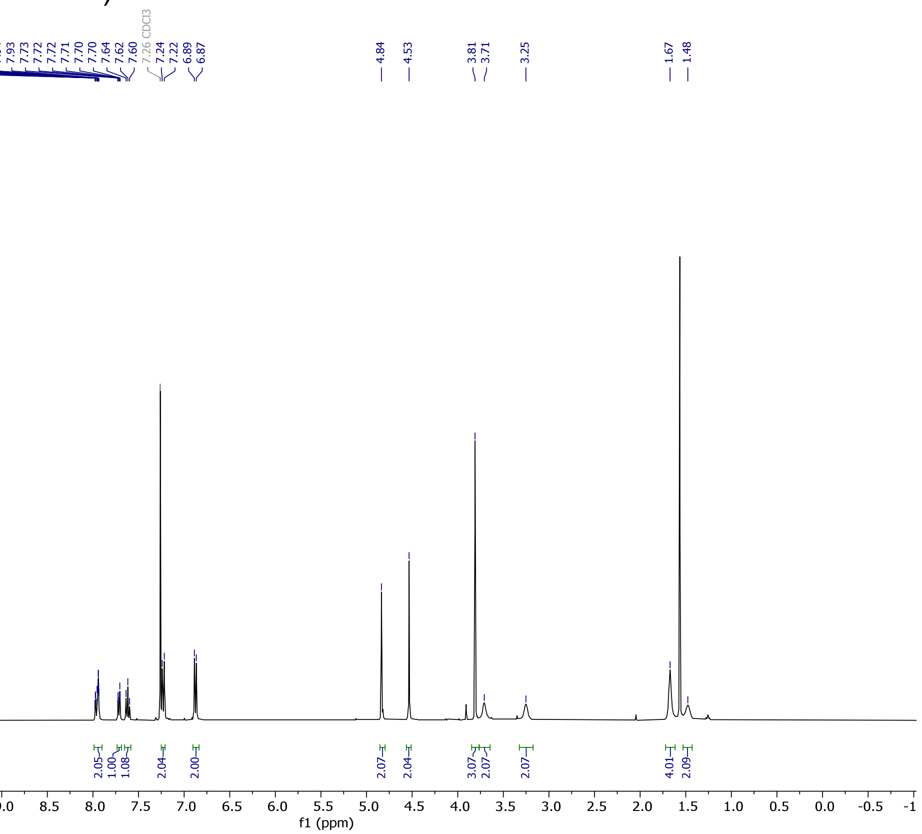

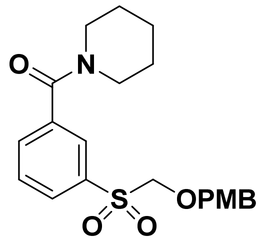

16b

^{13}C NMR ($CDCl_3$; 101 MHz)

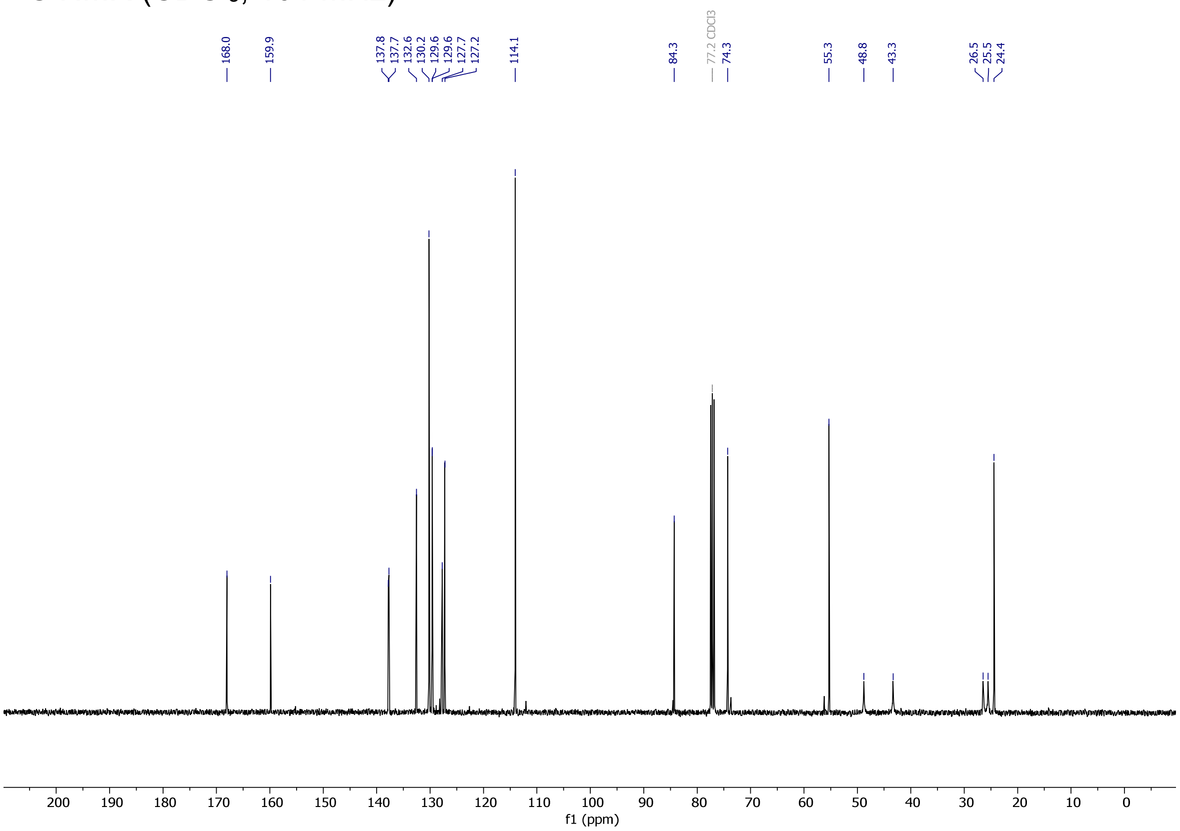

^{1}H NMR ($CDCl_3$, 400 MHz)

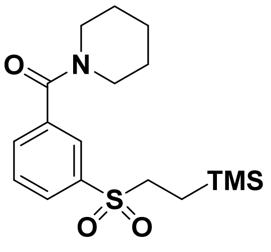

16c

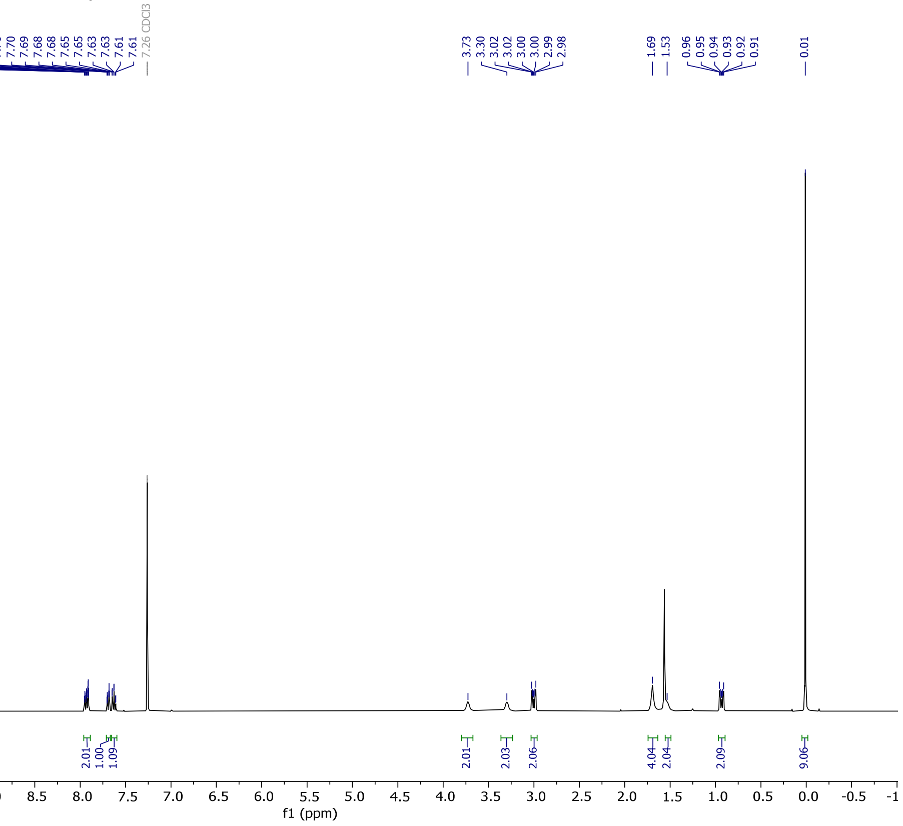

^{13}C NMR ($CDCl_3$; 101 MHz)

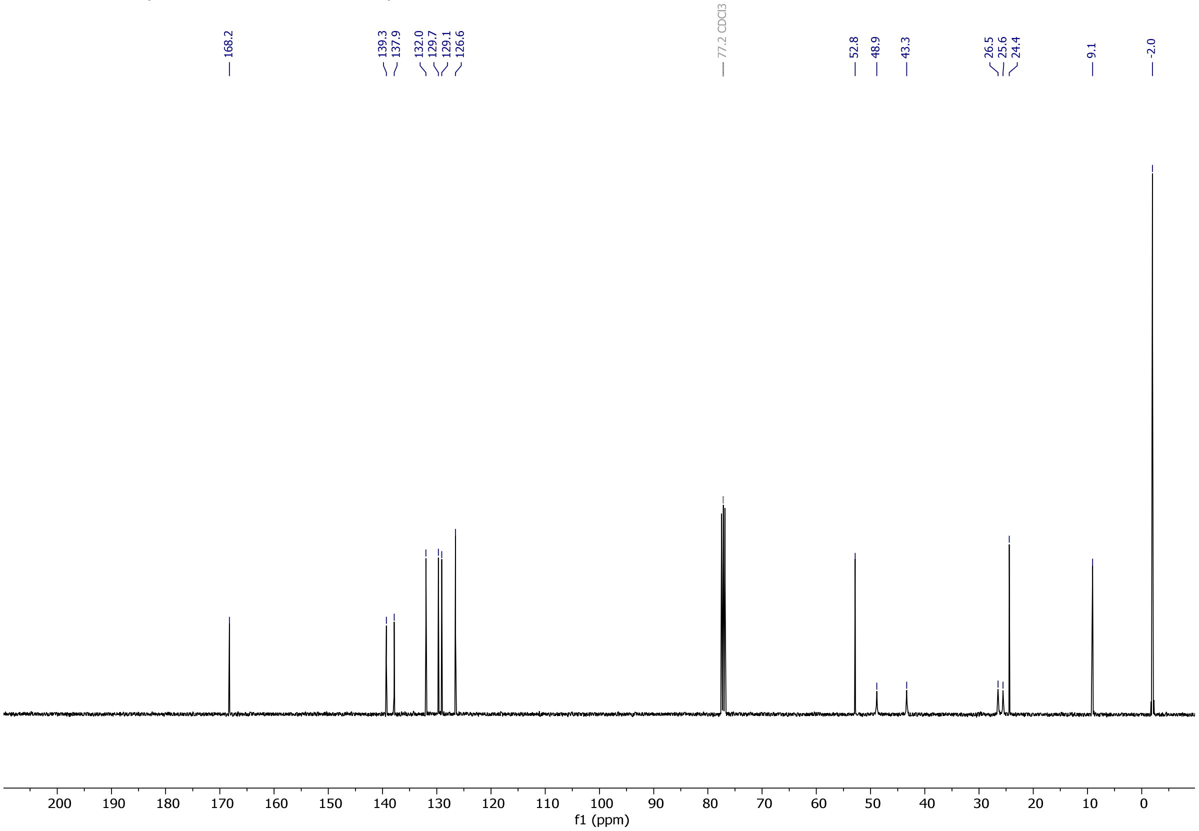

^{1}H NMR ($CDCl_3$, 400 MHz)

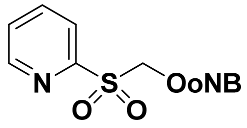

17a

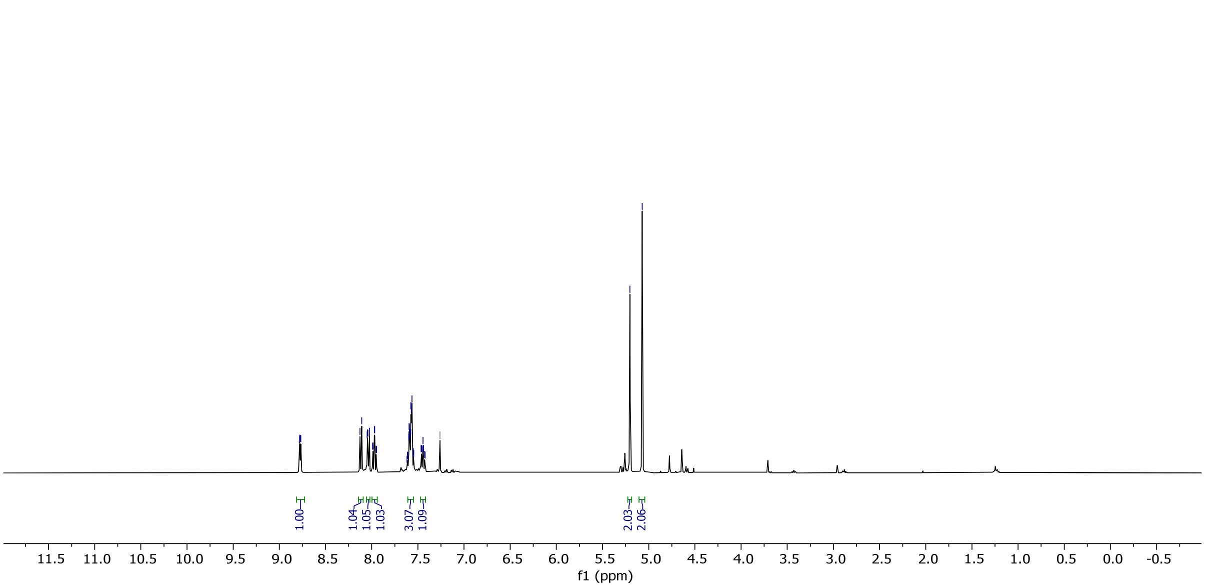

^{13}C NMR ($CDCl_3$; 101 MHz)

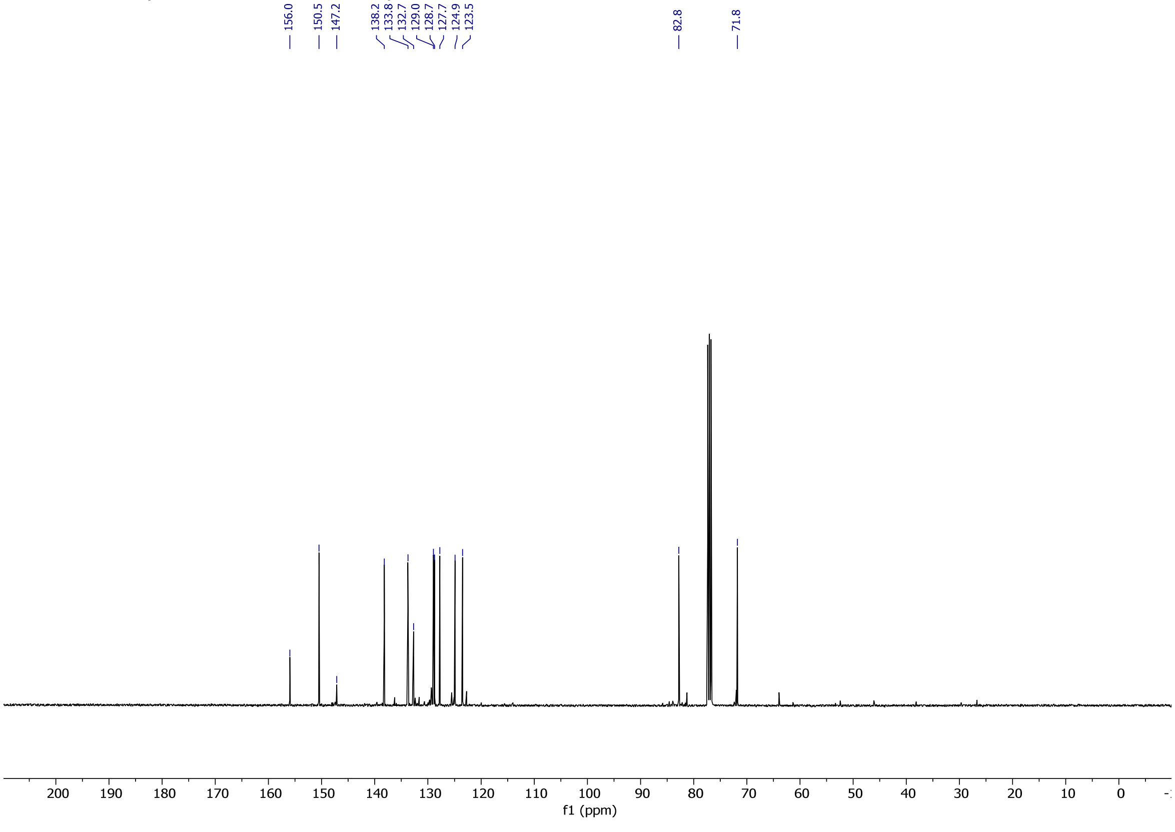

^1H NMR ($CDCl_3$, 400 MHz)

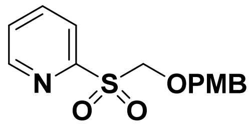

17b

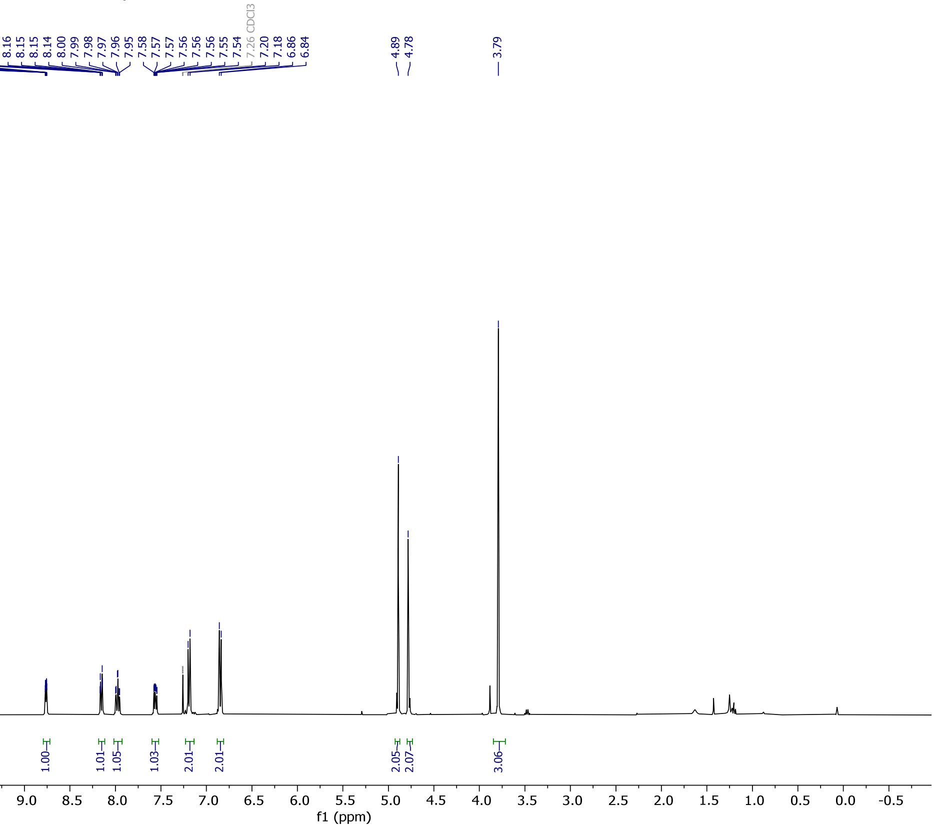

^{13}C NMR ($CDCl_3$; 101 MHz)

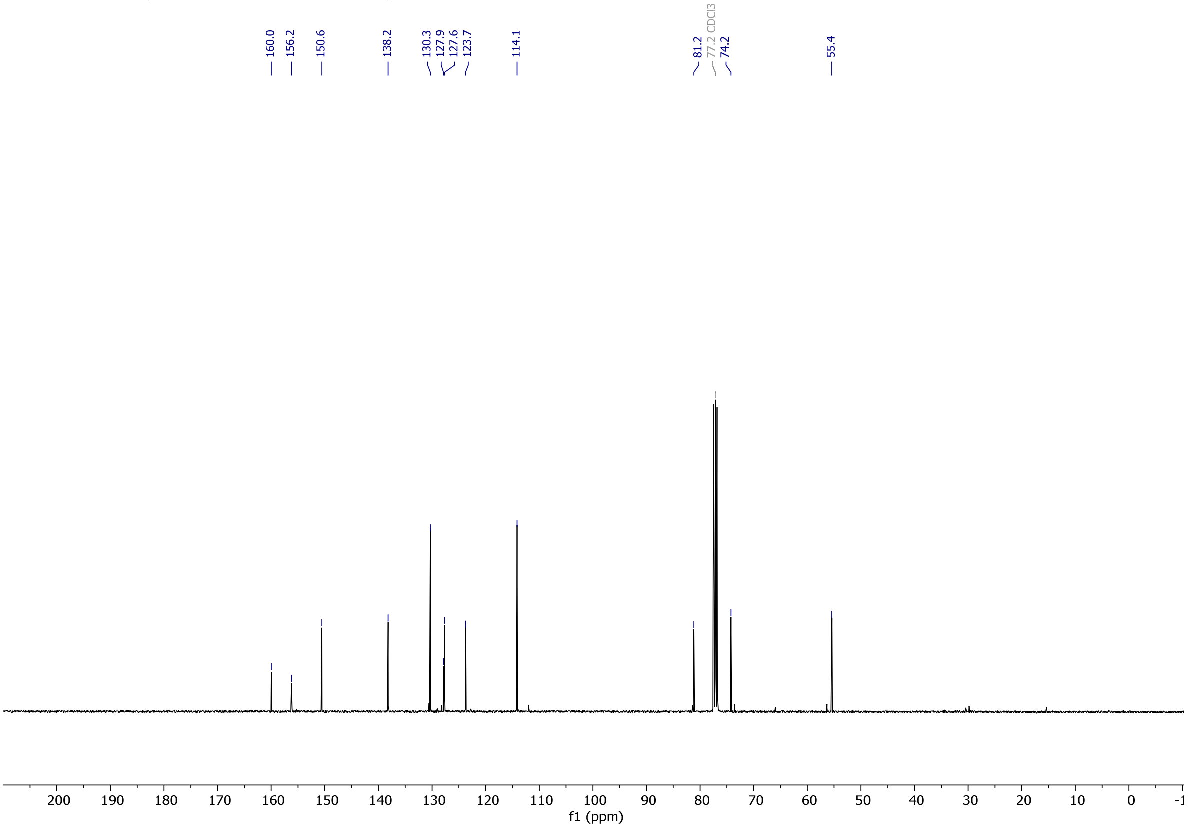

^{1}H NMR ($CDCl_3$, 400 MHz)

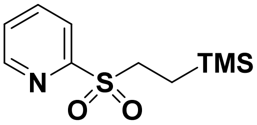

17c

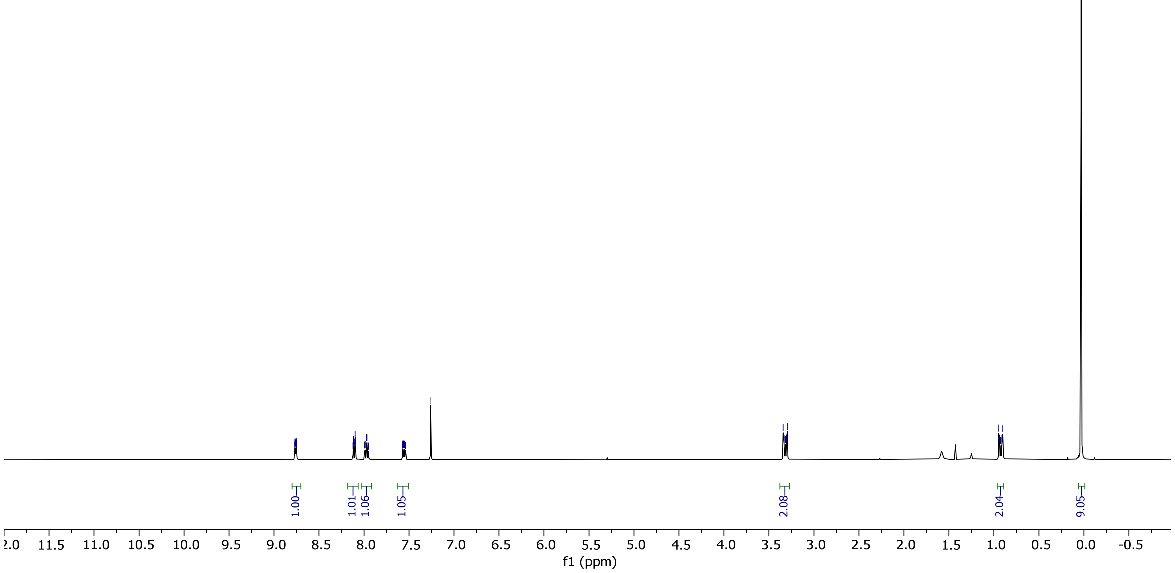

^{13}C NMR ($CDCl_3$; 101 MHz)

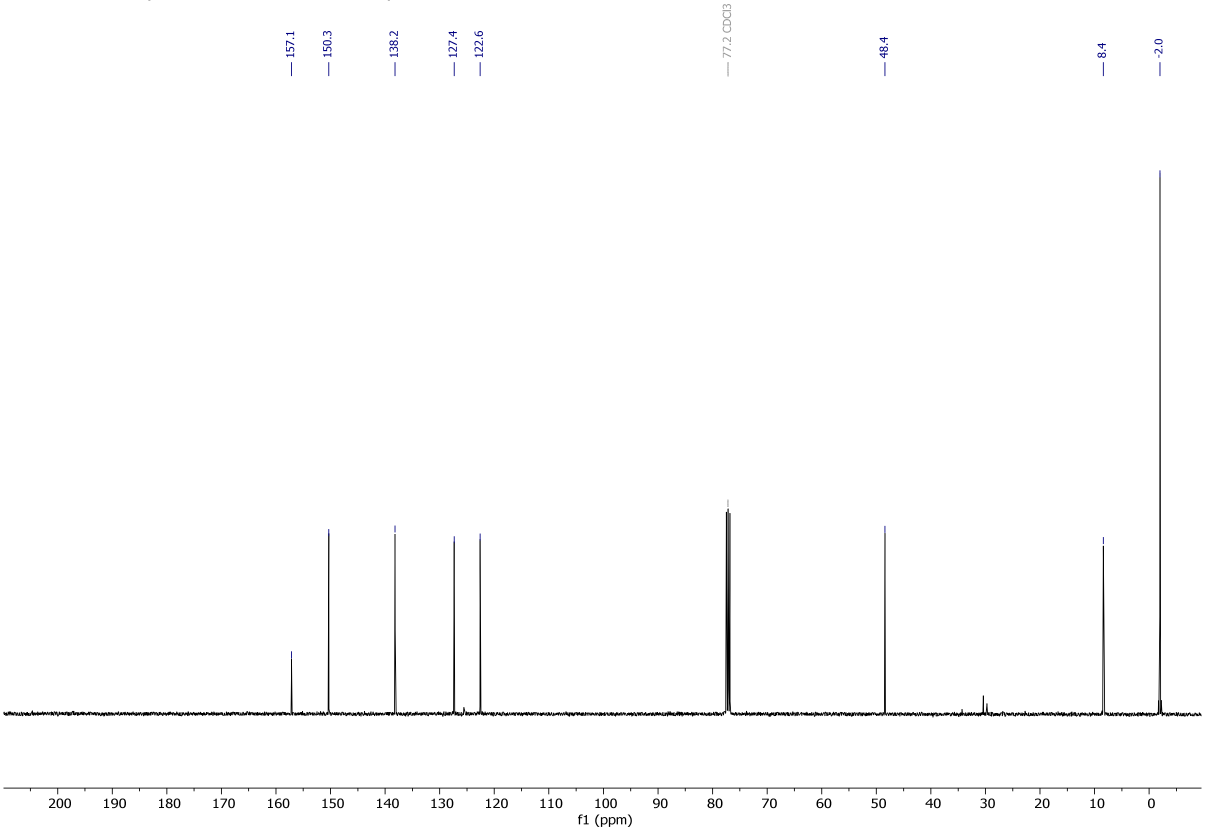

^{1}H NMR ($CDCl_3$, 400 MHz)

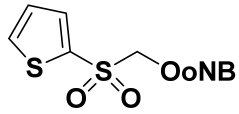

18a

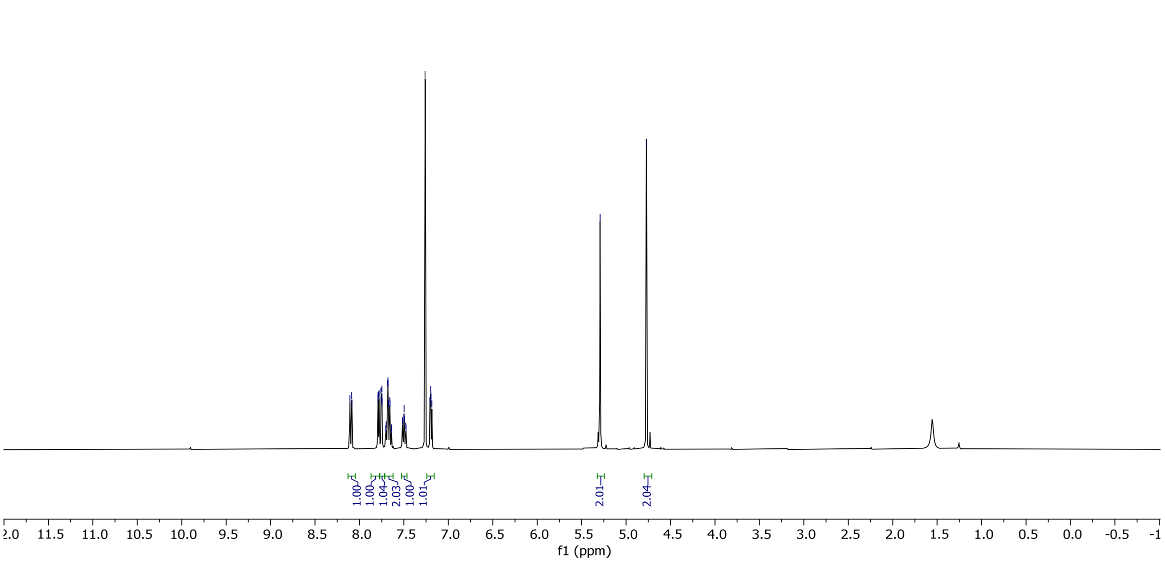

^{13}C NMR ($CDCl_3$; 101 MHz)

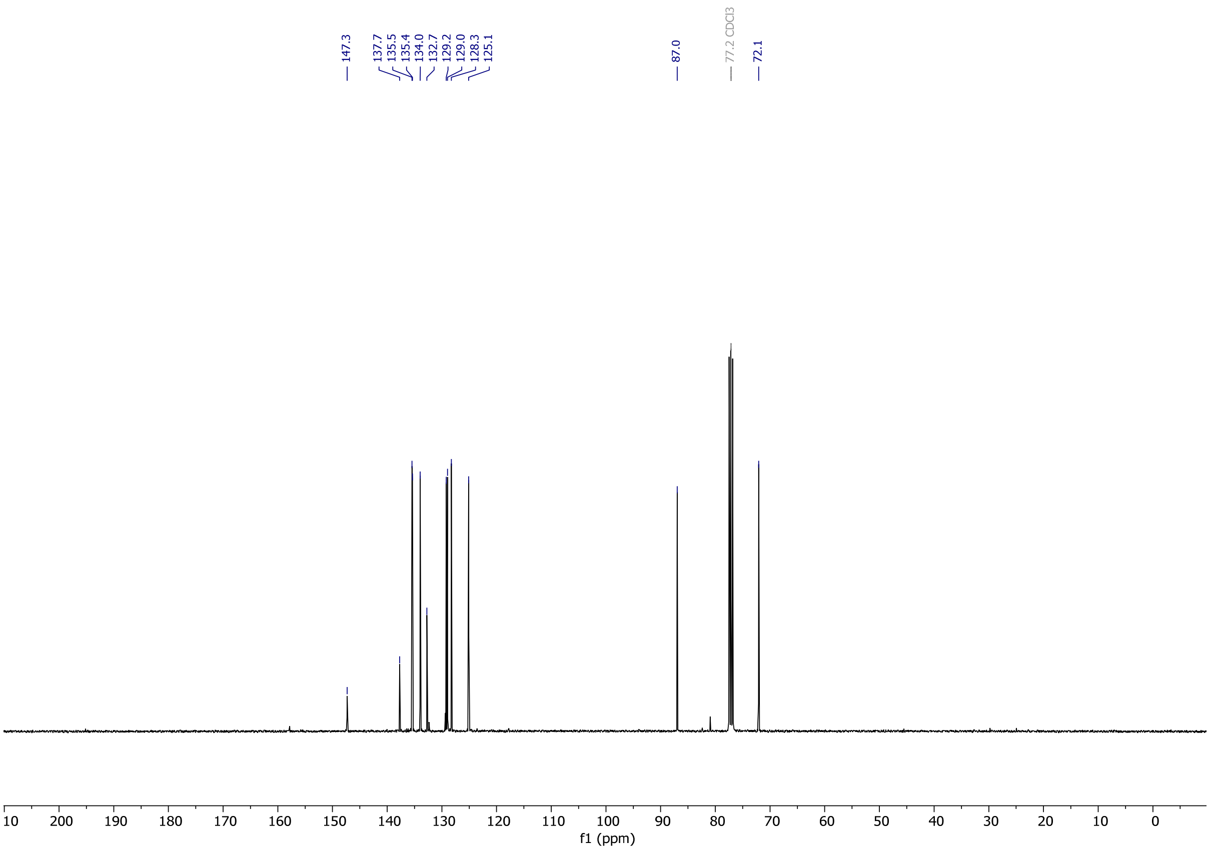

^{1}H NMR ($CDCl_3$, 400 MHz)

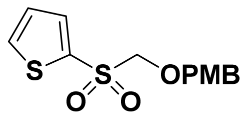

18b

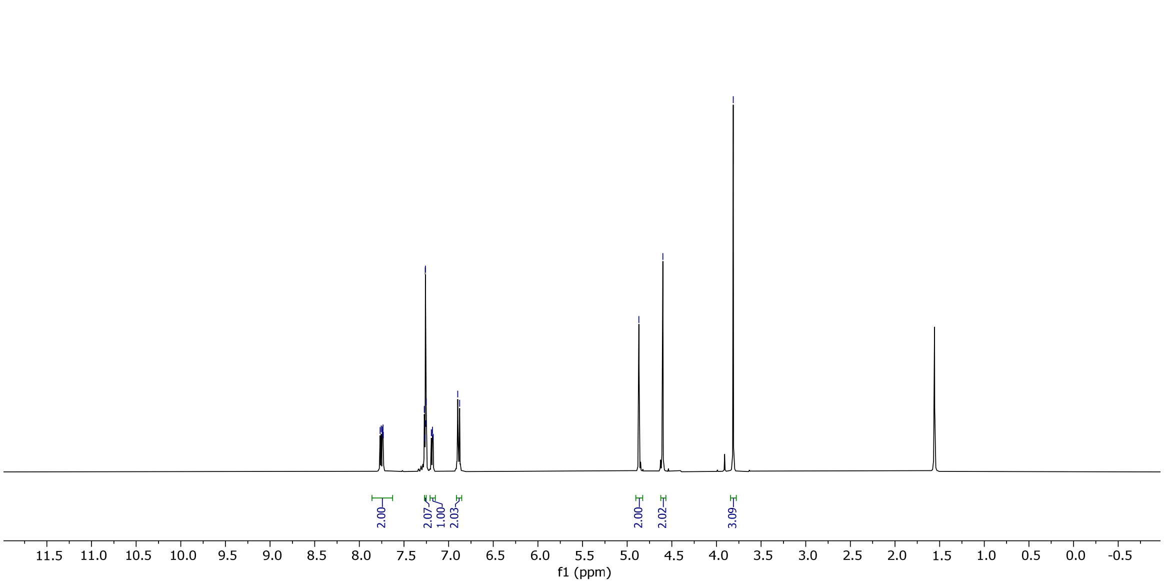

^{13}C NMR (CDCl$_3$; 101 MHz)

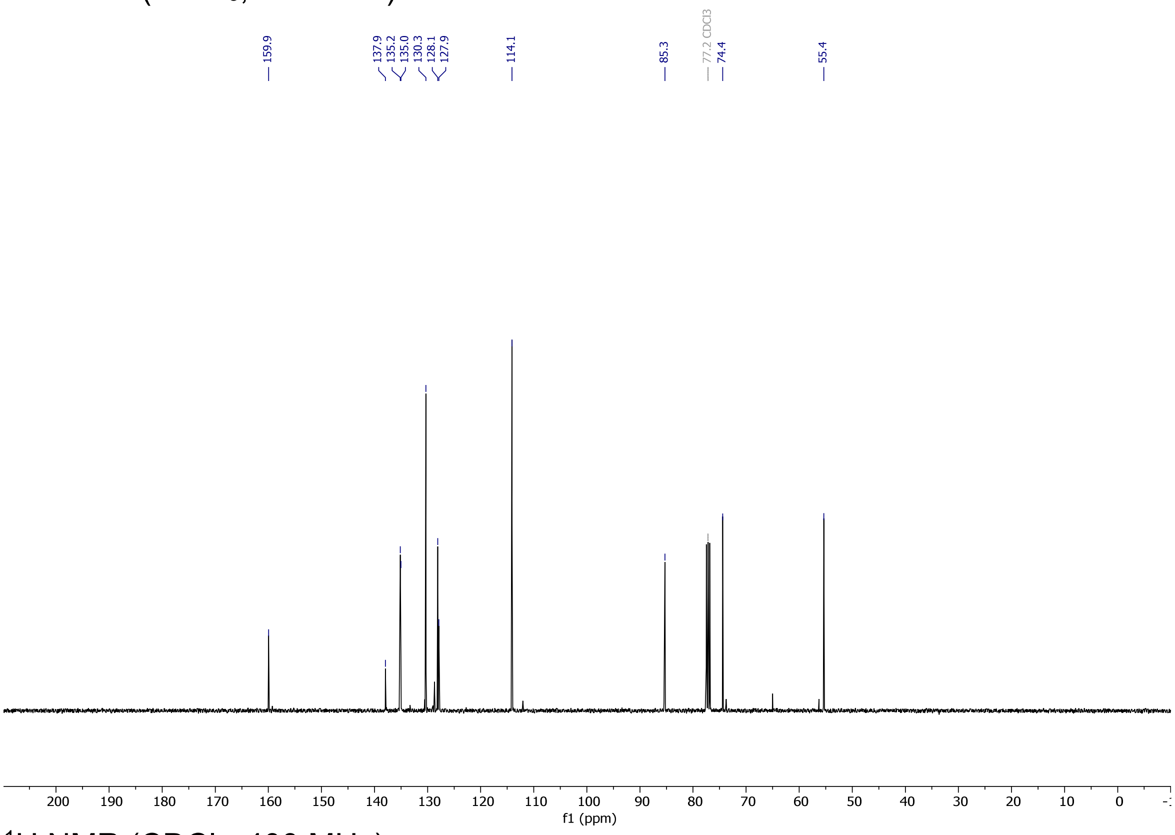

^{1}H NMR (CDCl$_3$, 400 MHz)

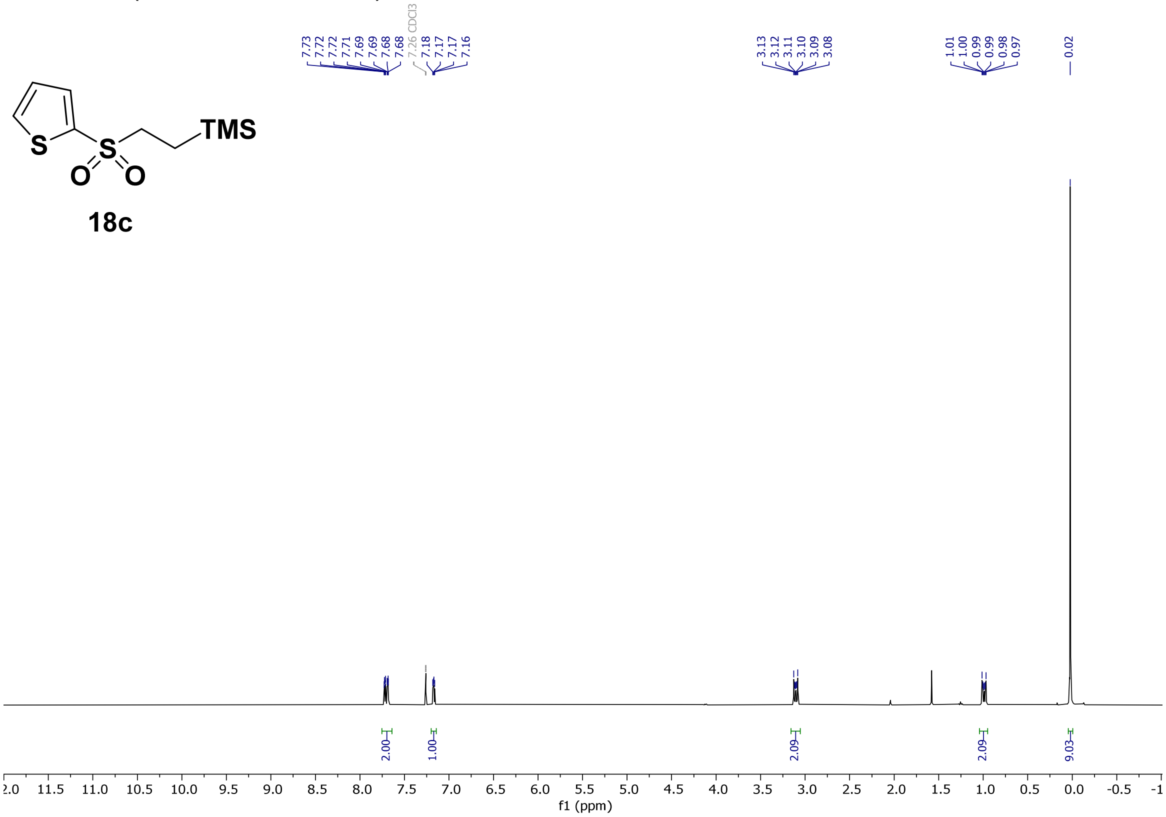

^{13}C NMR ($CDCl_3$; 101 MHz)

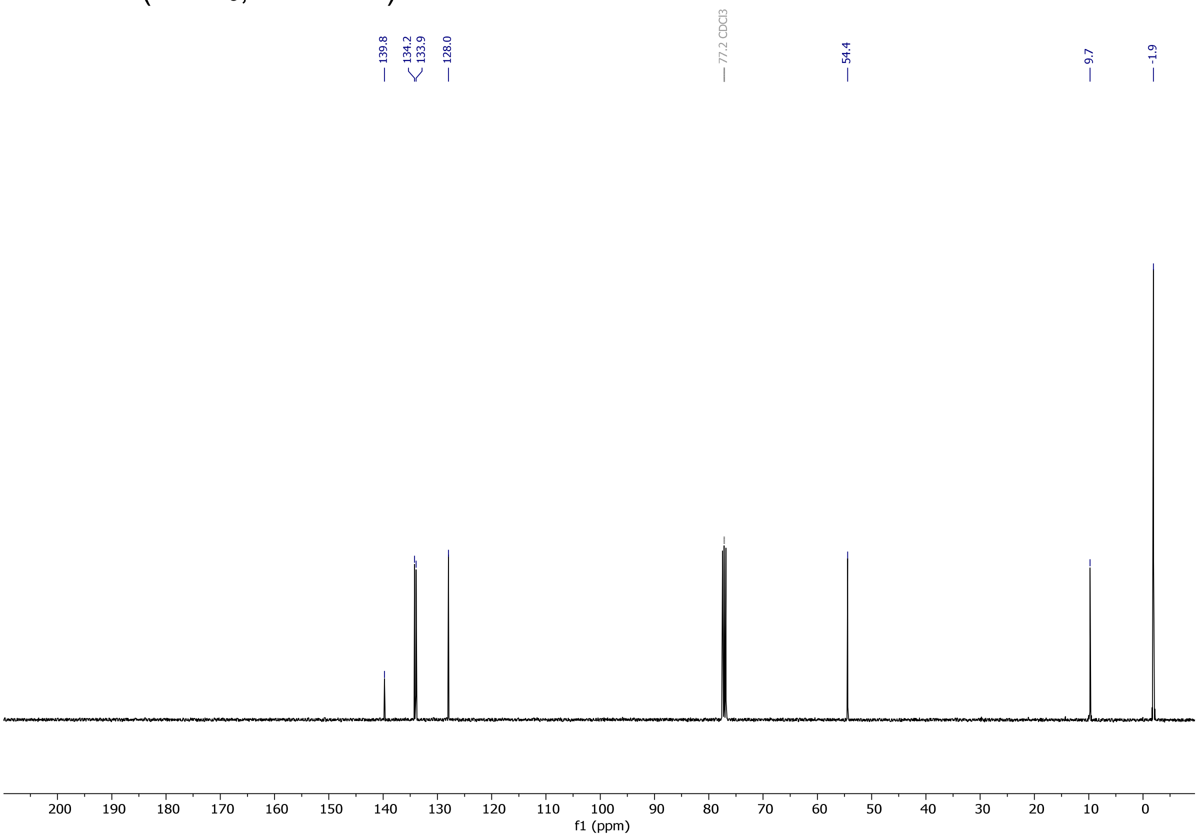

^{1}H NMR ($CDCl_3$, 400 MHz)

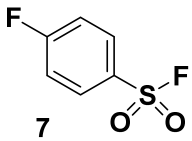

7

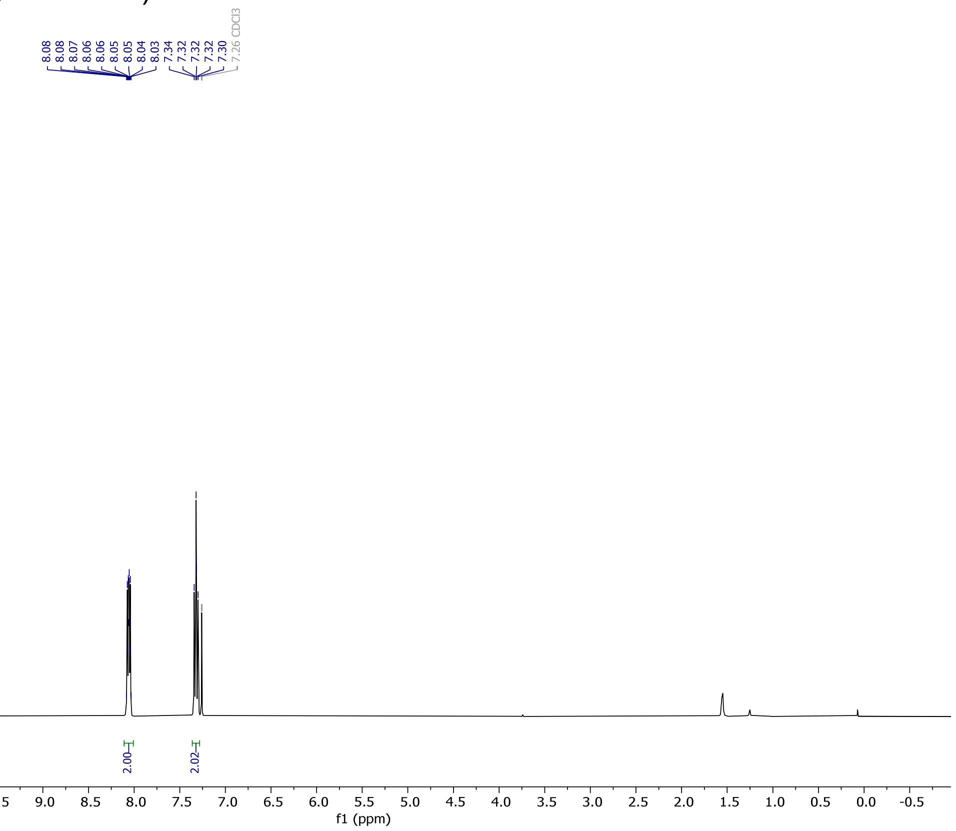

^{1}H NMR ($CDCl_3$, 400 MHz)

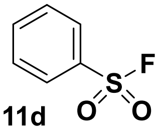

11d

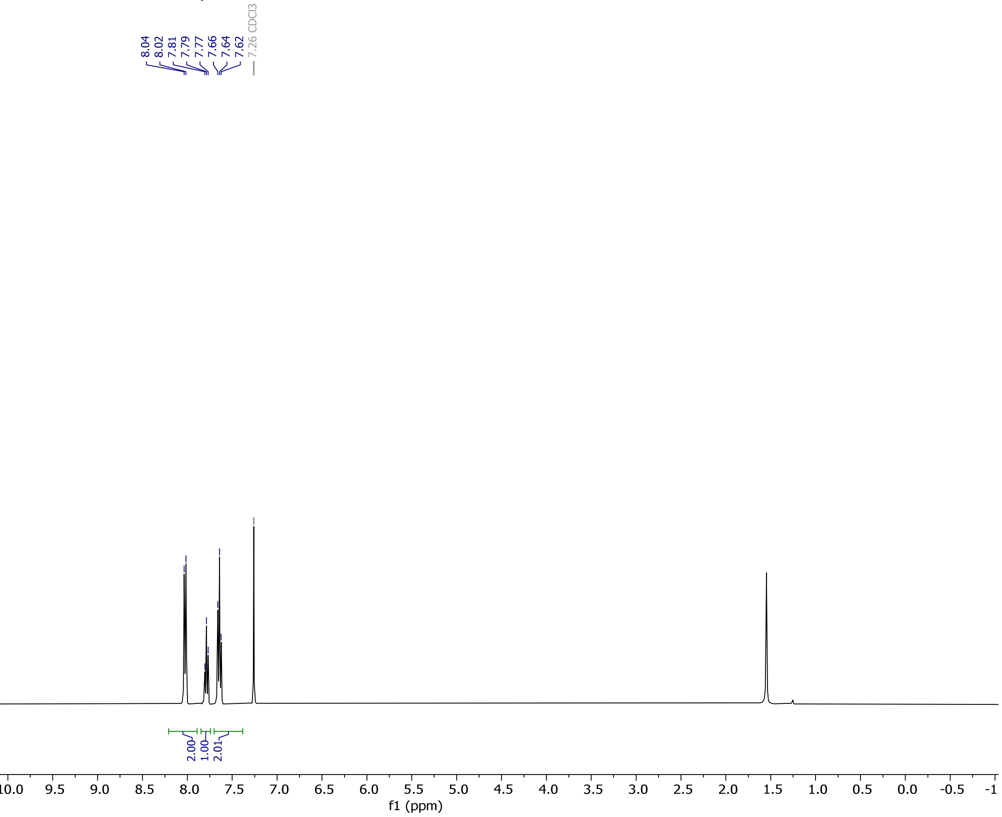

^{1}H NMR ($CDCl_3$, 400 MHz)

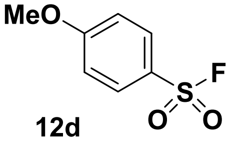

12d

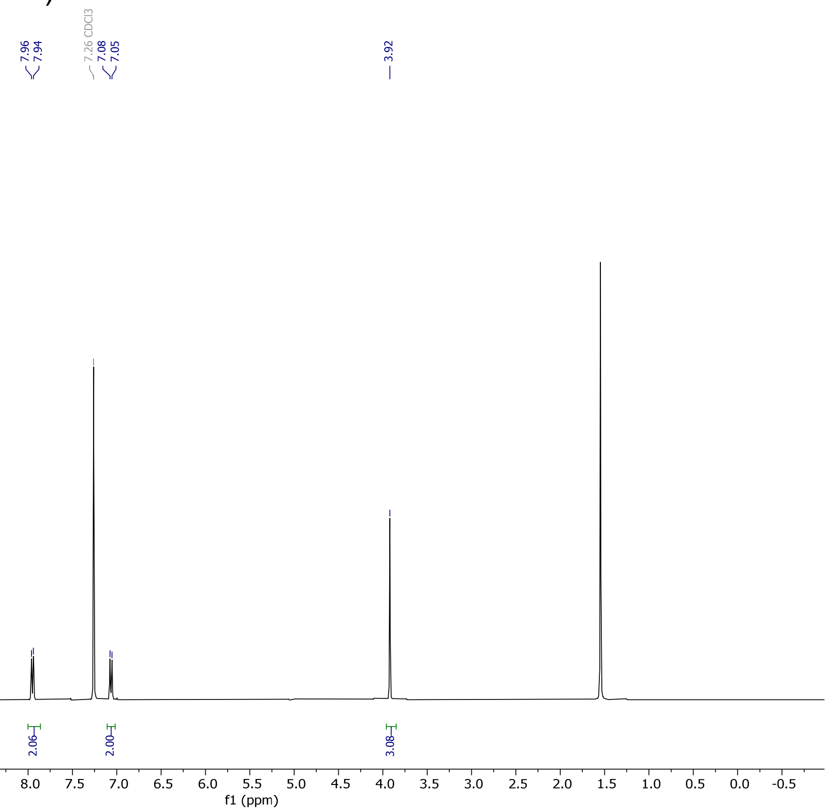

^{1}H NMR ($CDCl_3$, 400 MHz)

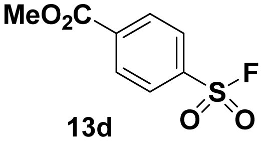

13d

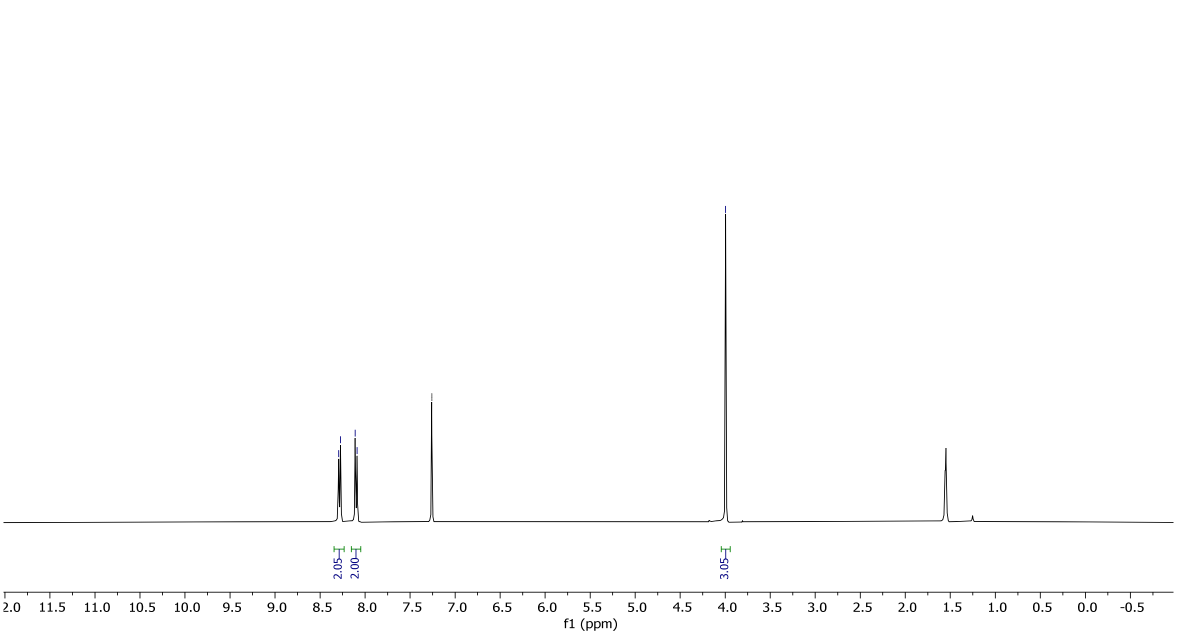

^{1}H NMR ($CDCl_3$, 400 MHz)

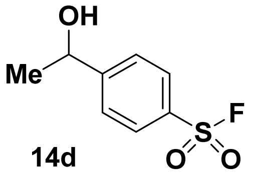

14d

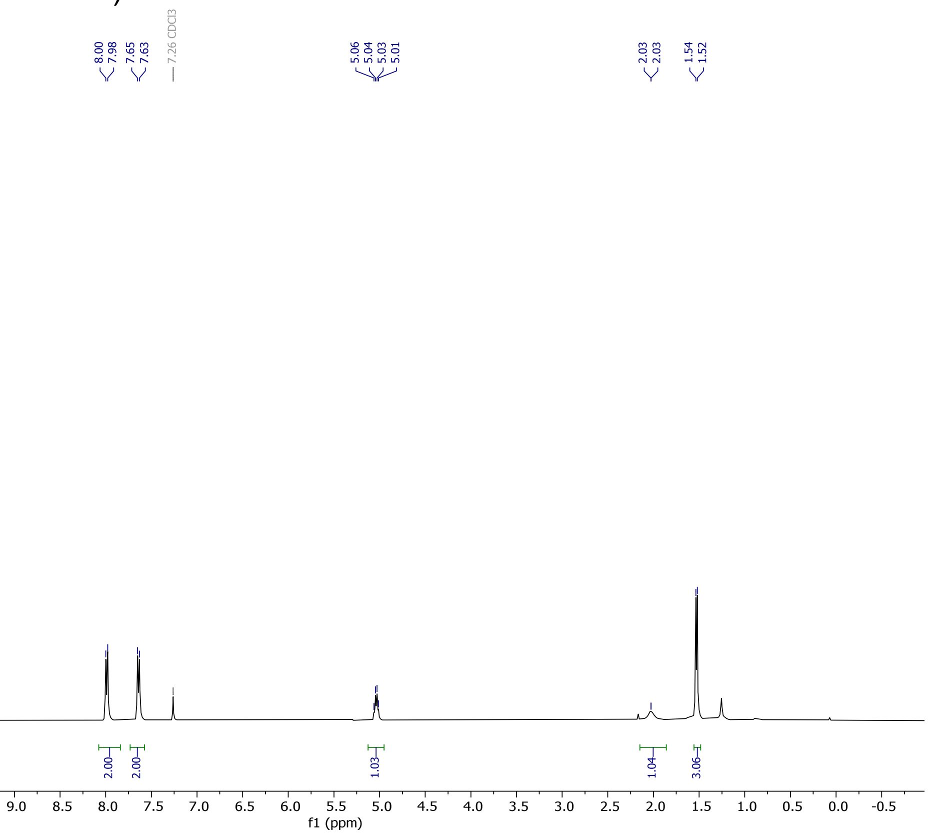

^{13}C NMR ($CDCl_3$; 101 MHz)

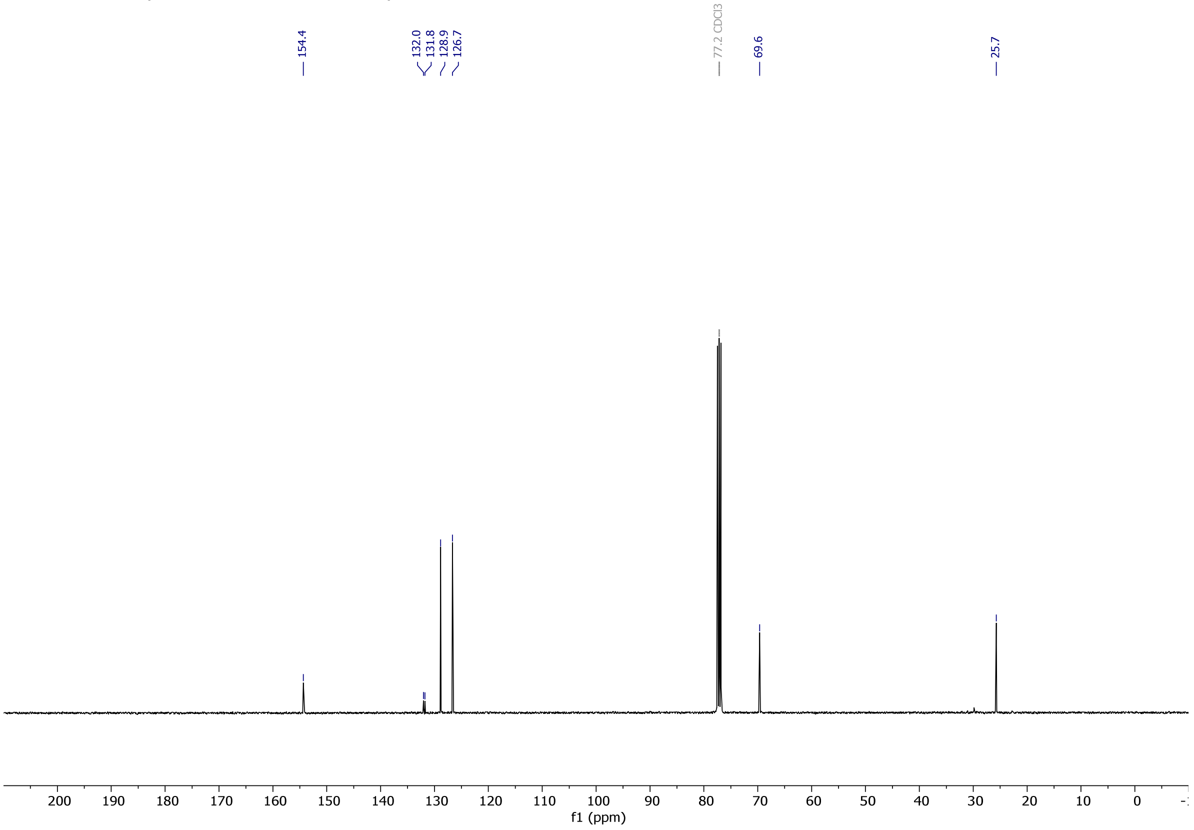

^{19}F NMR ($CDCl_3$; 376 MHz)

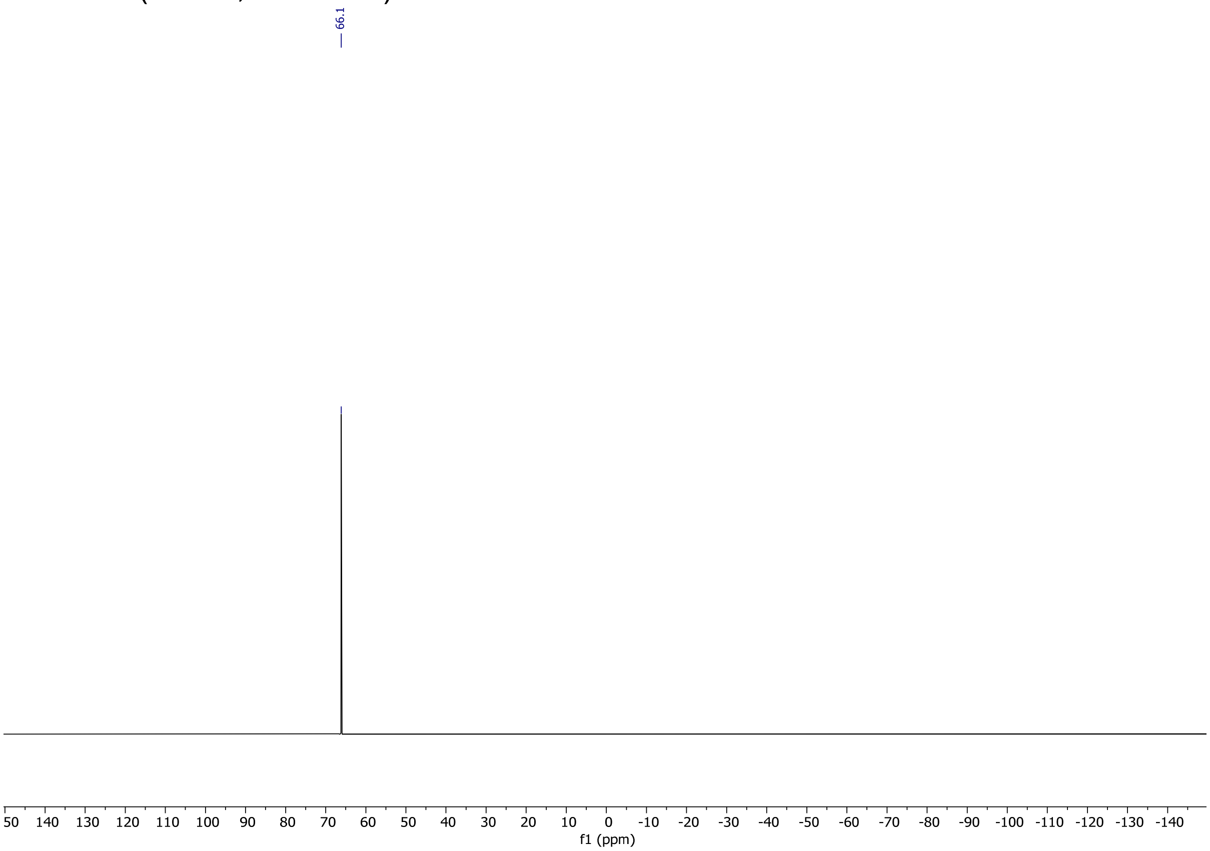

^1H NMR ($CDCl_3$, 400 MHz)

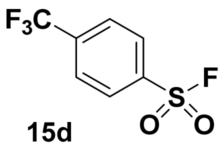

15d

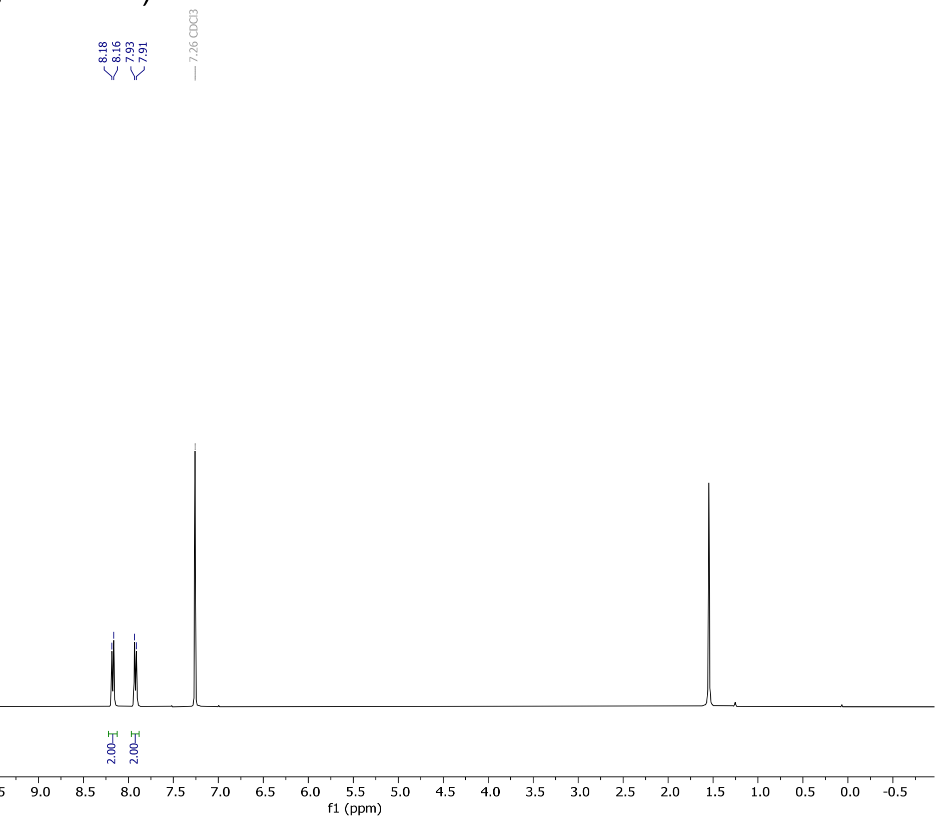

^{1}H NMR ($CDCl_3$, 400 MHz)

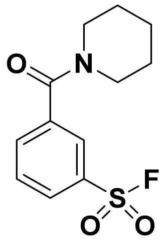

16d

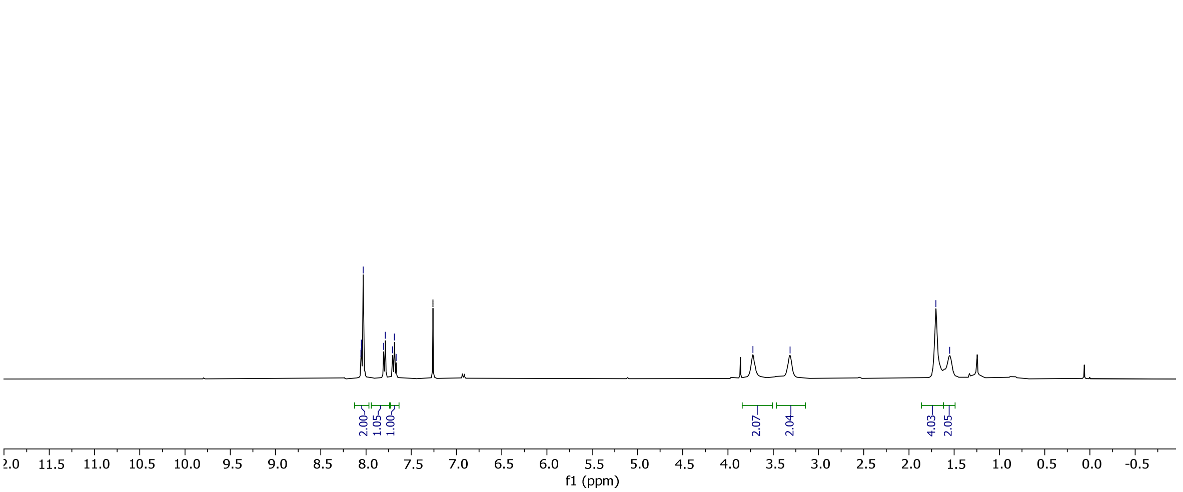

^{13}C NMR ($CDCl_3$; 101 MHz)

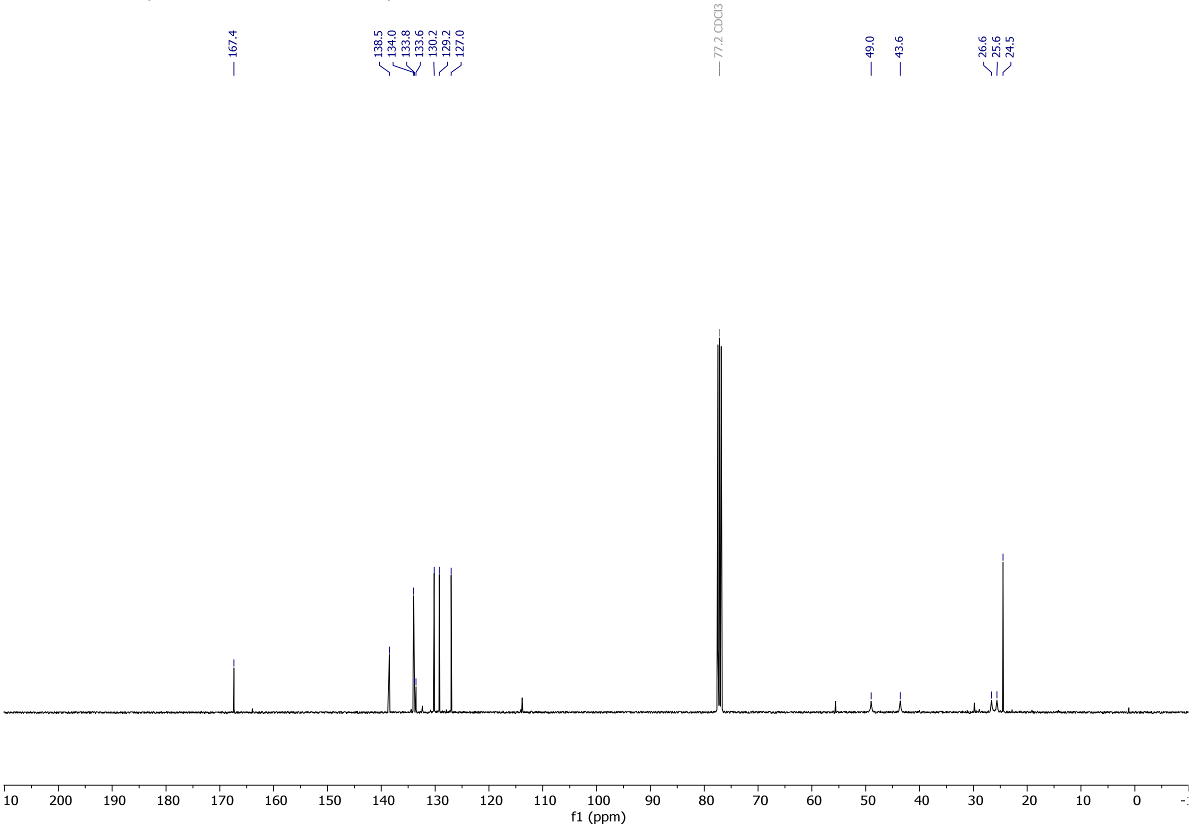

^{19}F NMR ($CDCl_3$; 376 MHz)

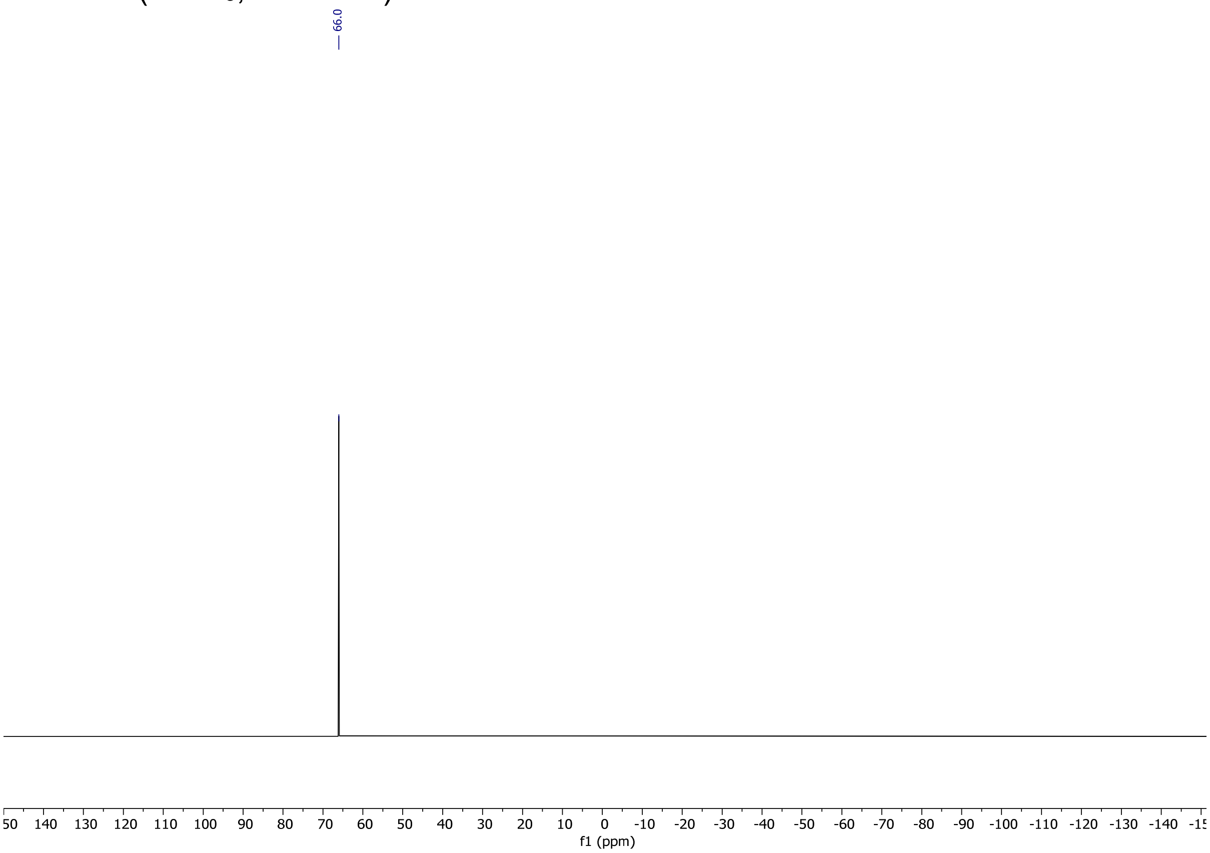

^1H NMR ($CDCl_3$, 400 MHz)

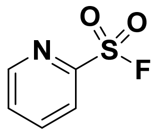

17d

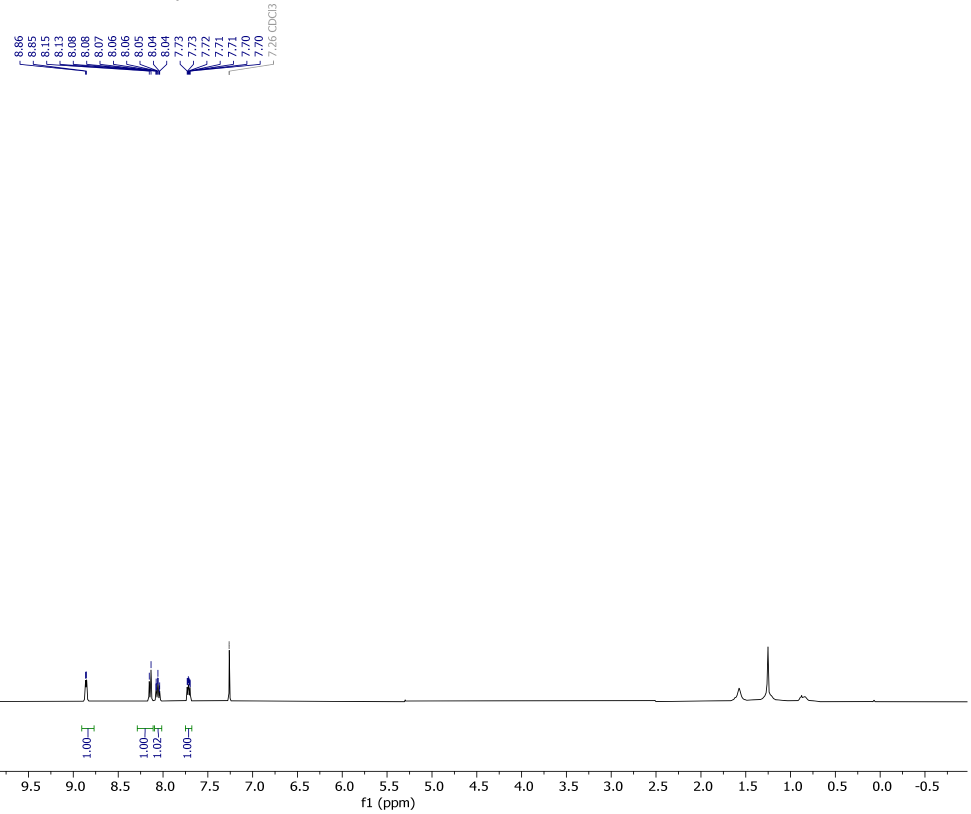

^{1}H NMR ($CDCl_3$, 400 MHz)

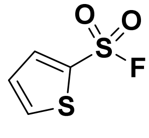

18d

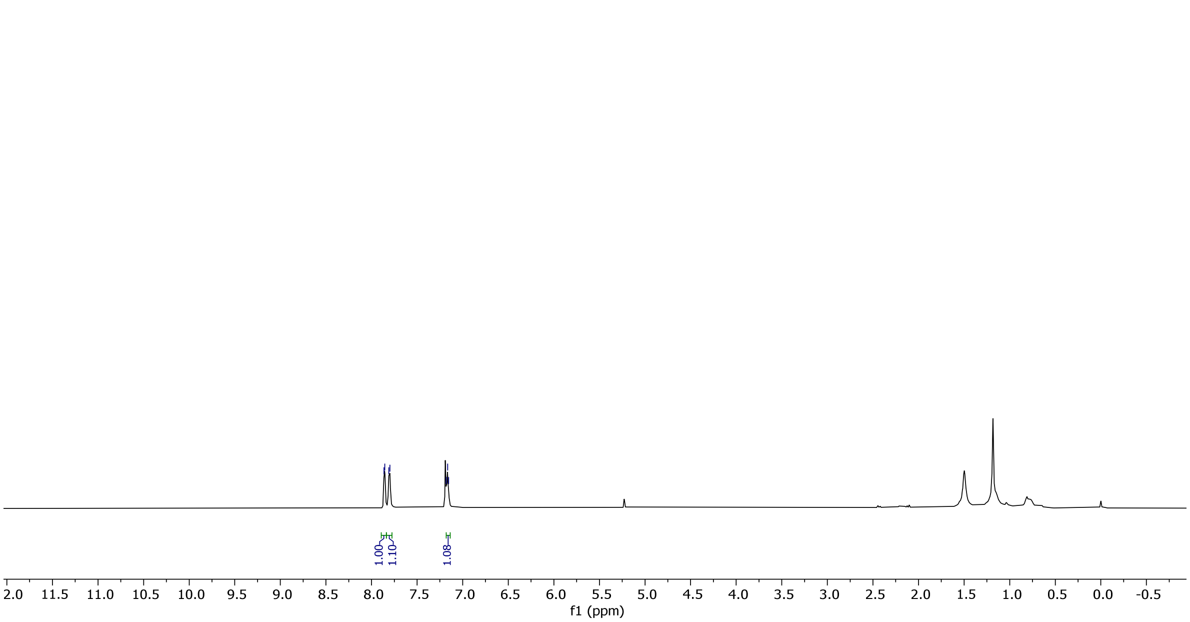

^1H NMR ($CDCl_3$, 400 MHz)

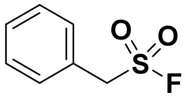

19d

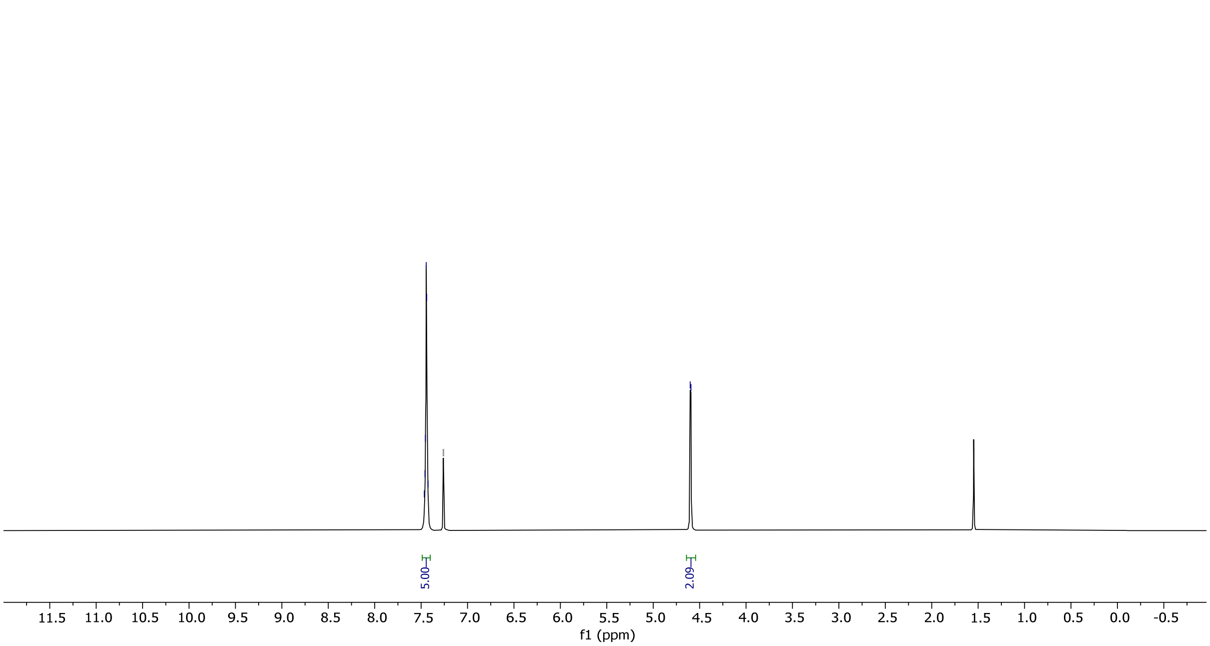

^{1}H NMR ($CDCl_3$, 400 MHz)

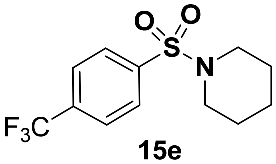

15e

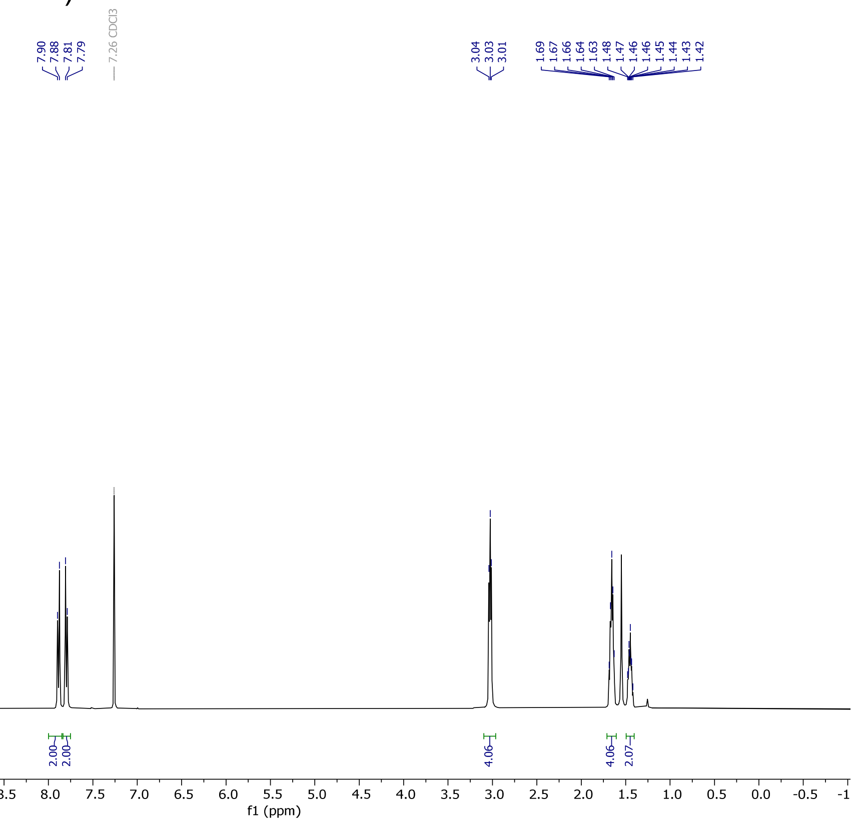

^{13}C NMR ($CDCl_3$; 101 MHz)

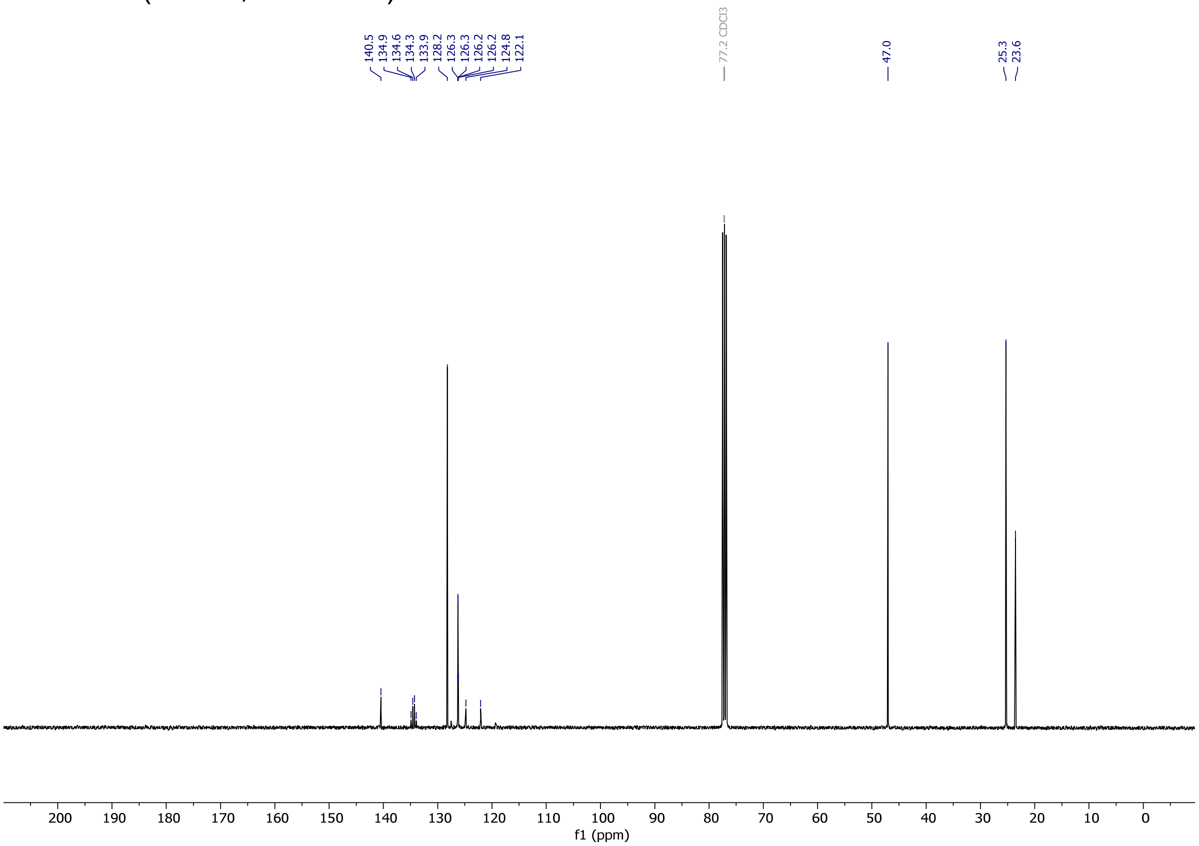

^{19}F NMR ($CDCl_3$; 376 MHz)

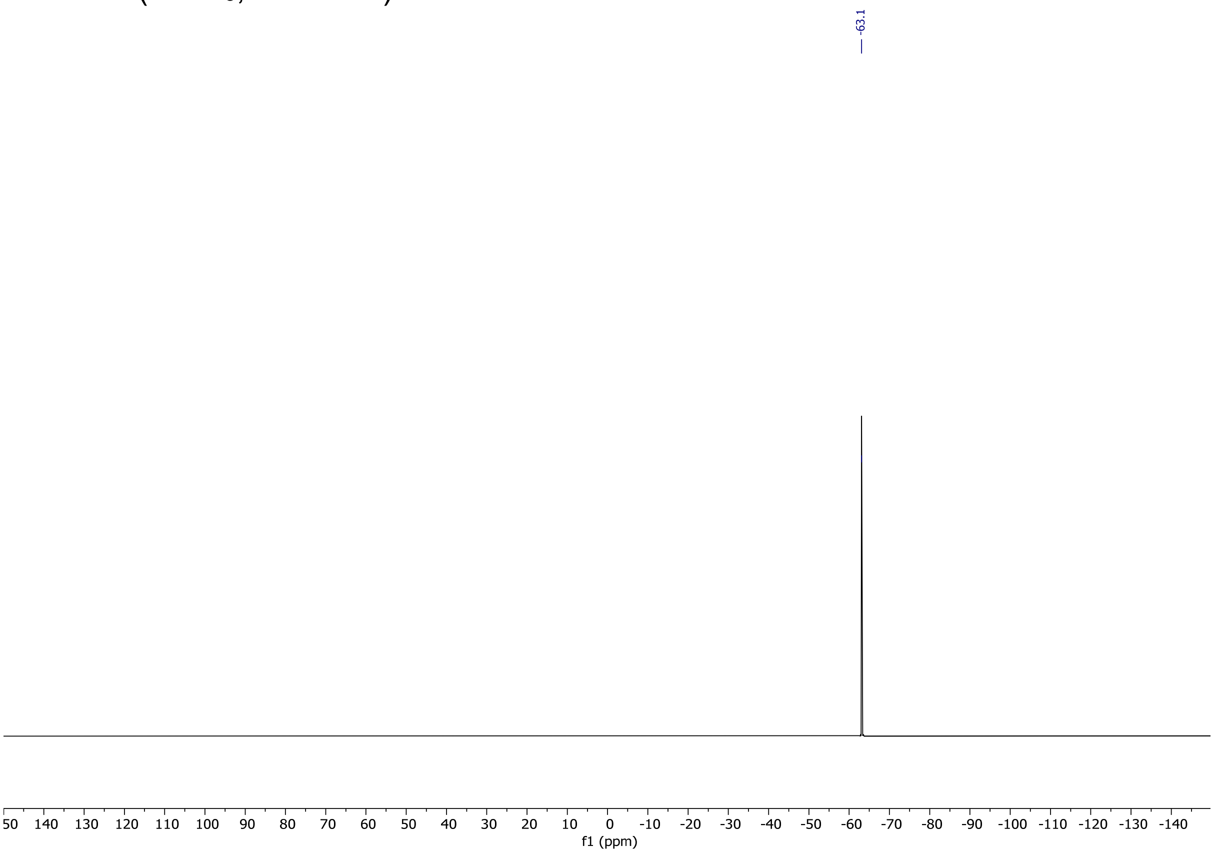

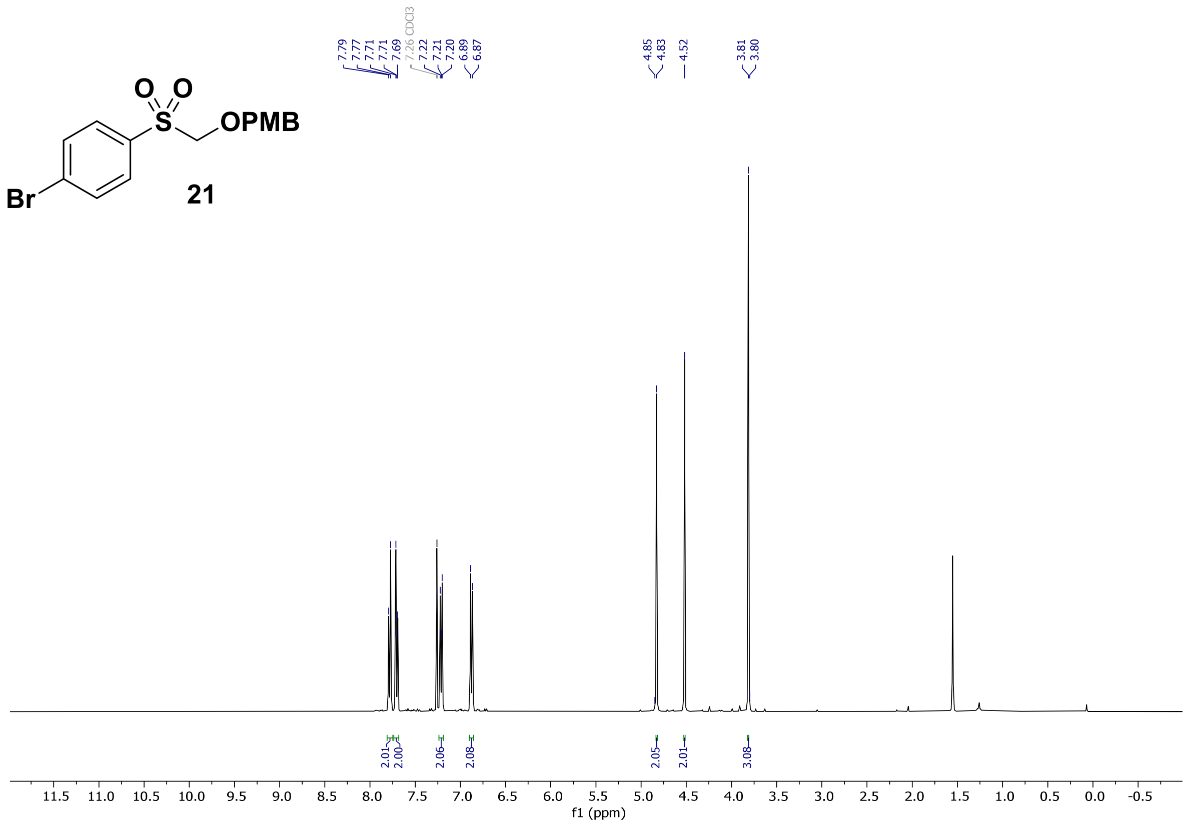

21

^{13}C NMR ($CDCl_3$; 101 MHz)

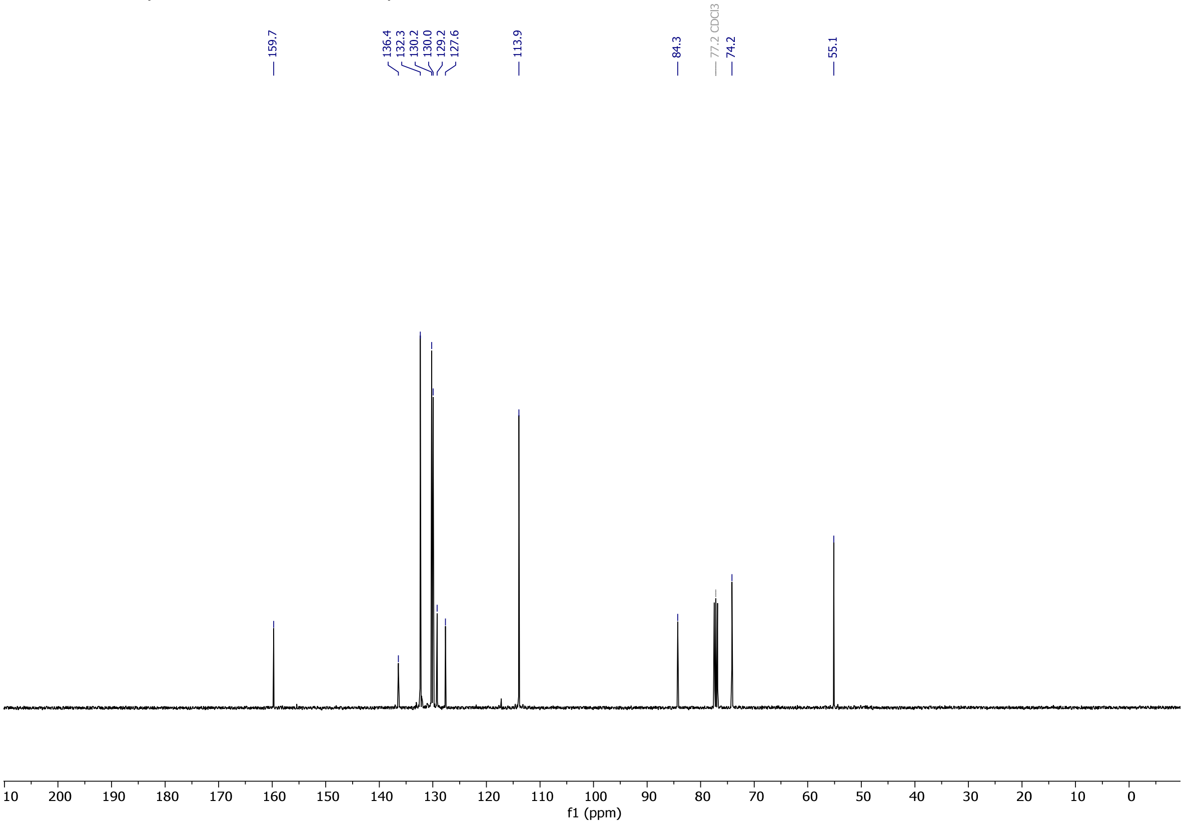

^{1}H NMR ($CDCl_3$, 400 MHz)

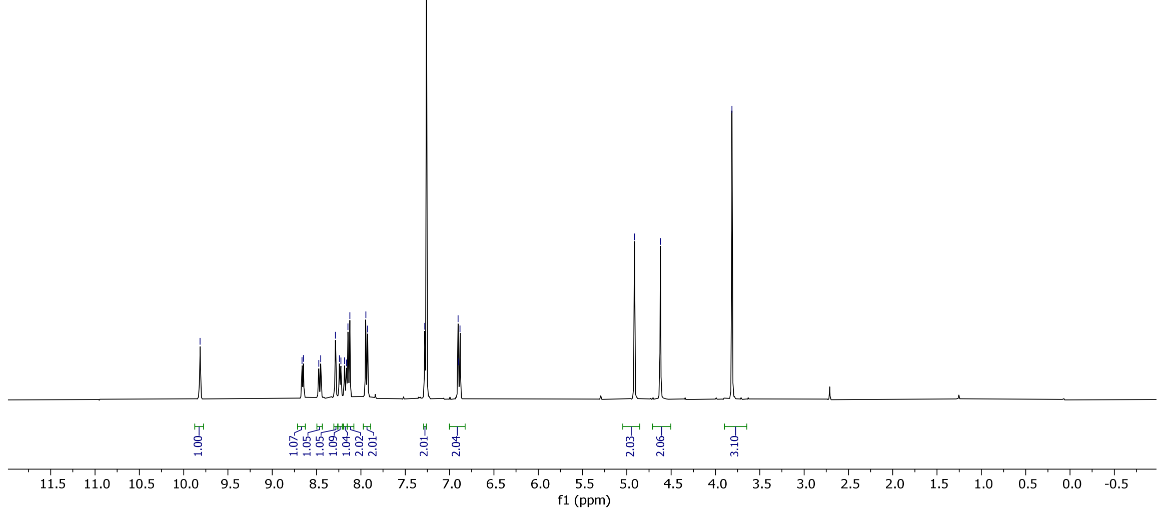

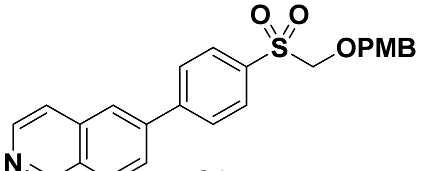

S17a

^{13}C NMR ($CDCl_3$; 101 MHz)

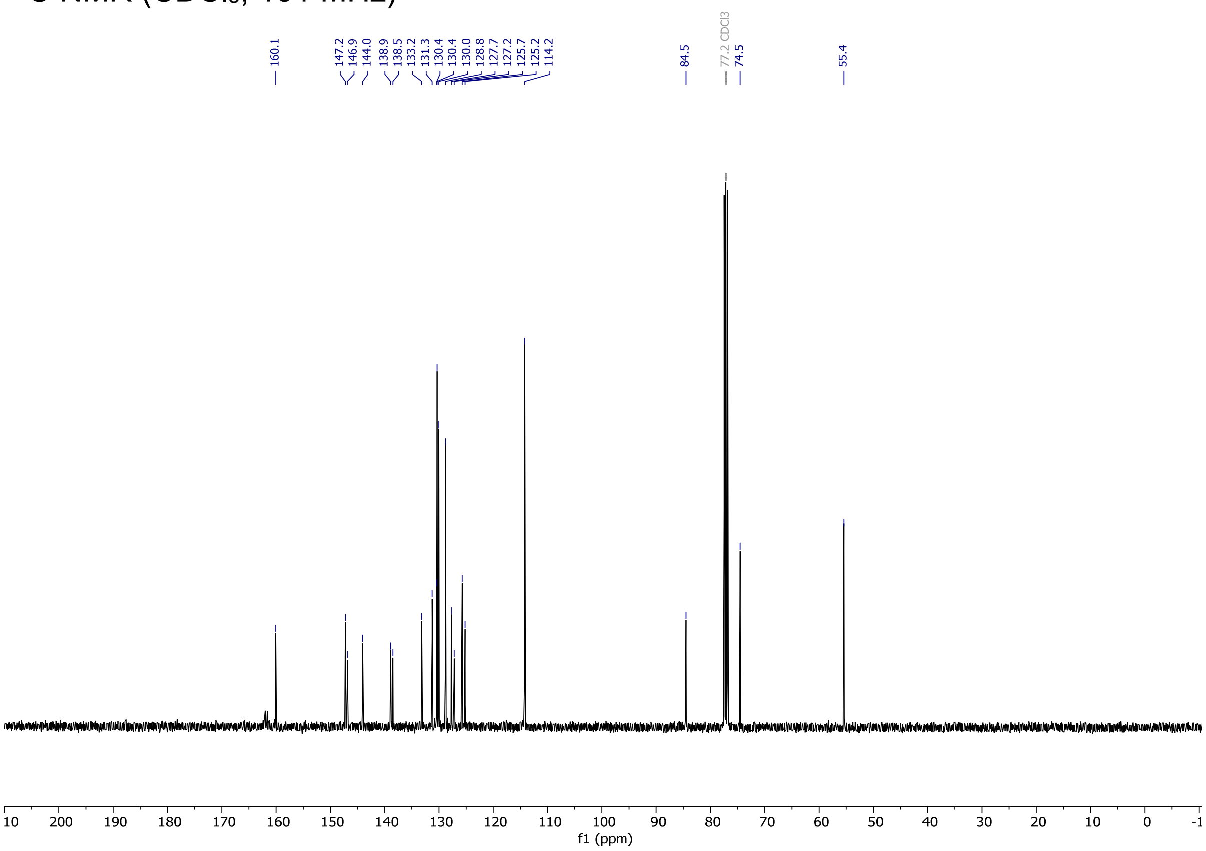

^{1}H NMR (CD_3CN, 400 MHz)

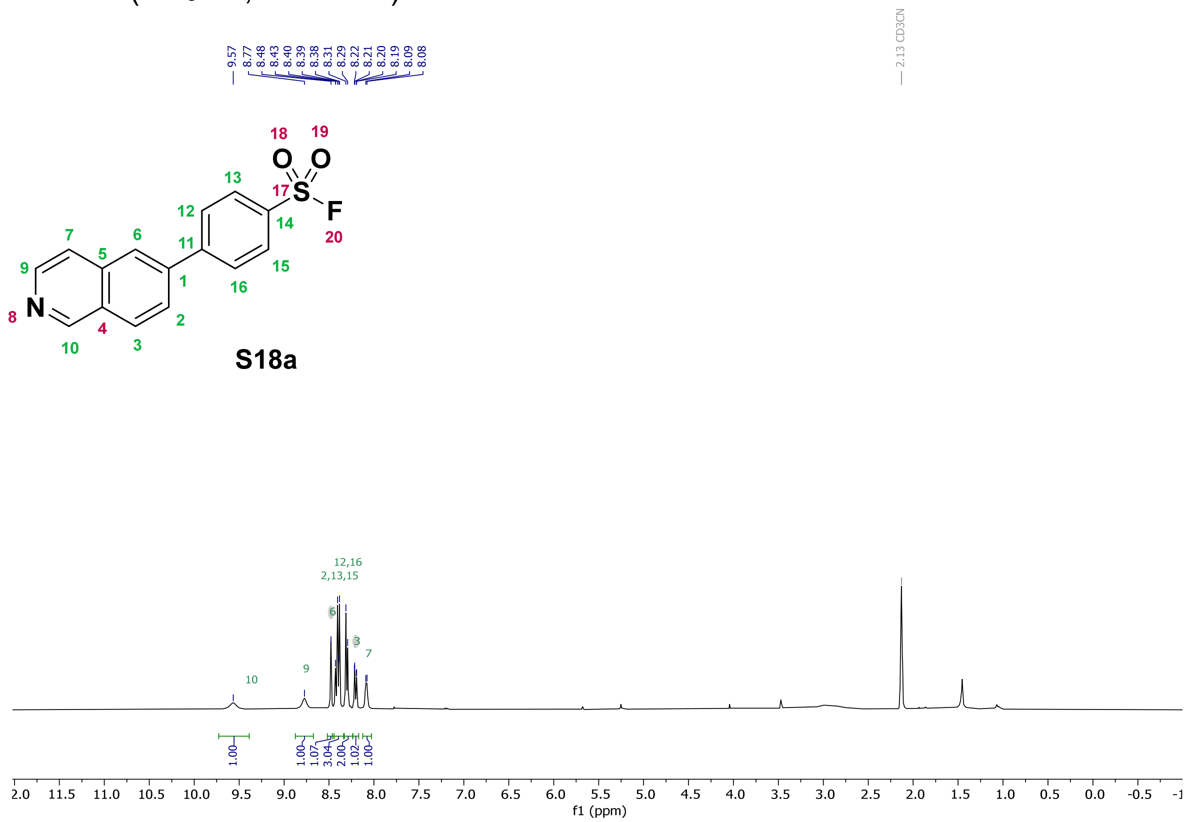

^{13}C NMR (CD_3CN; 101 MHz)

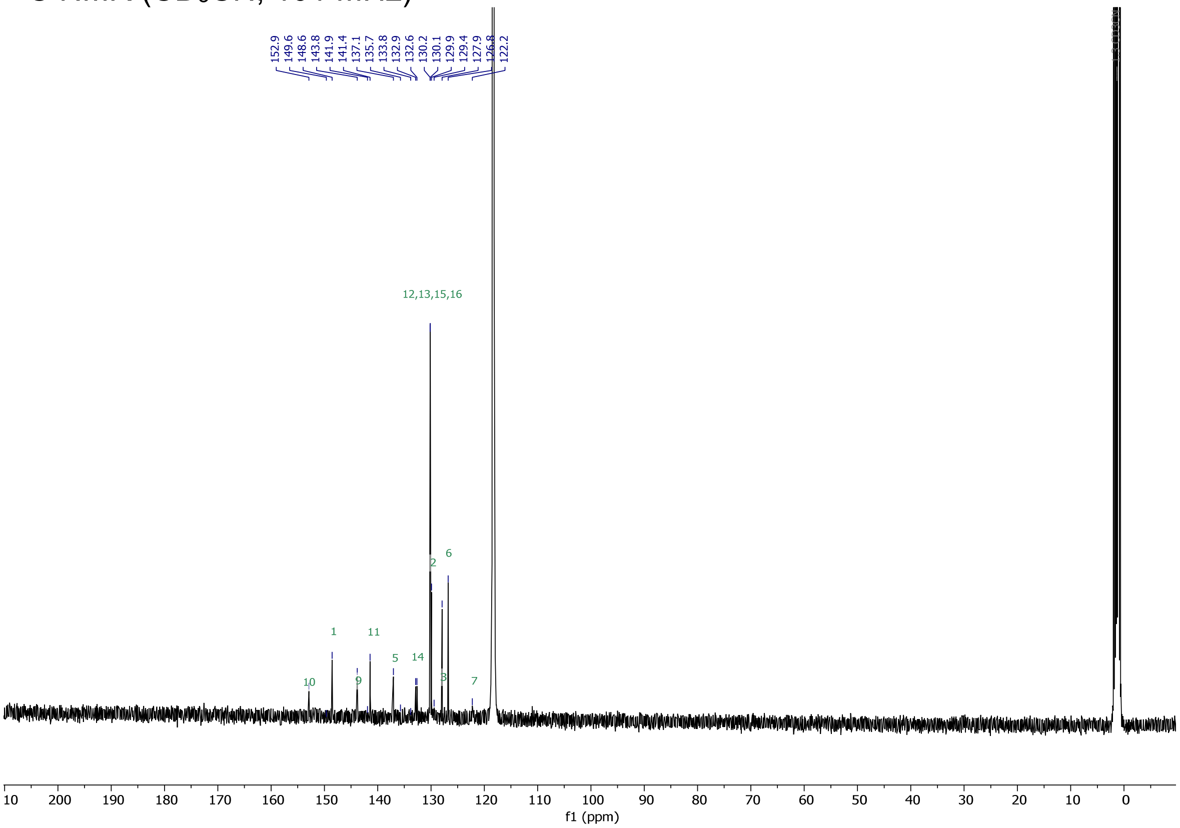

HMQC ($f1$: 101 MHz, $f2$: 400 MHz)

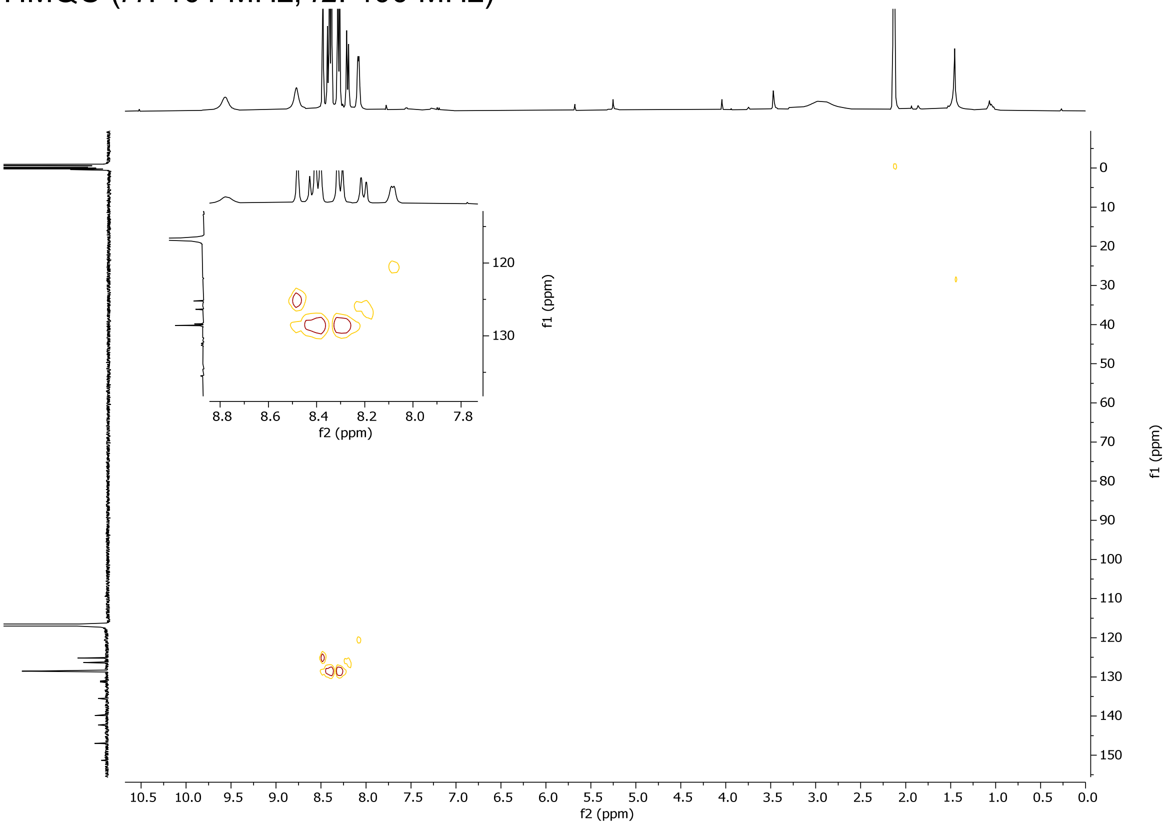

HSQC ($f1$: 101 MHz, $f2$: 400 MHz)

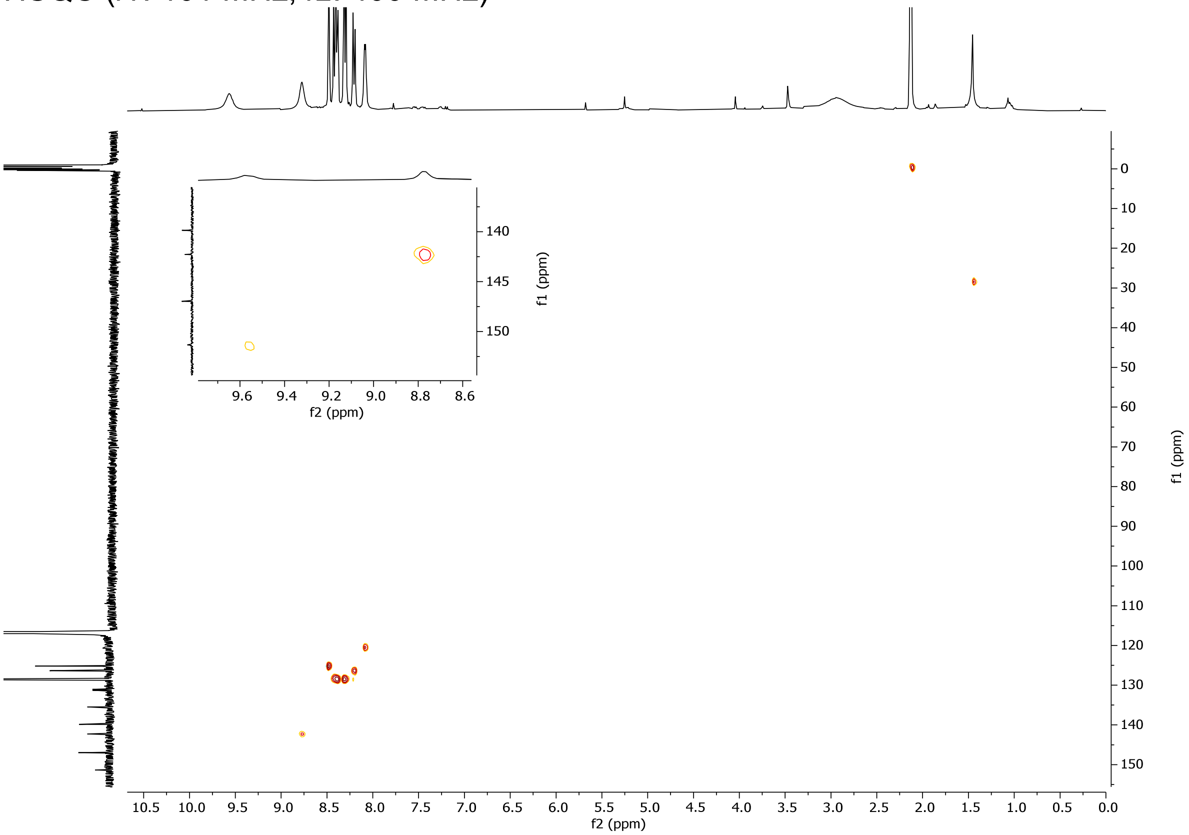

^{1}H NMR ($CDCl_3$, 400 MHz)

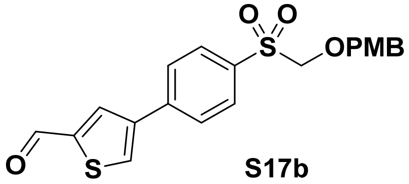

S17b

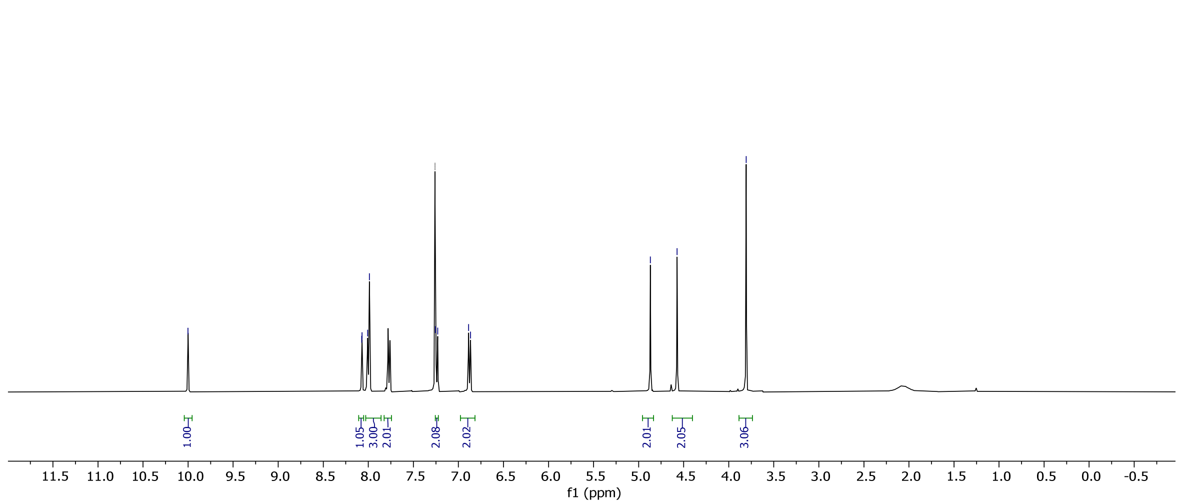

^{13}C NMR ($CDCl_3$; 101 MHz)

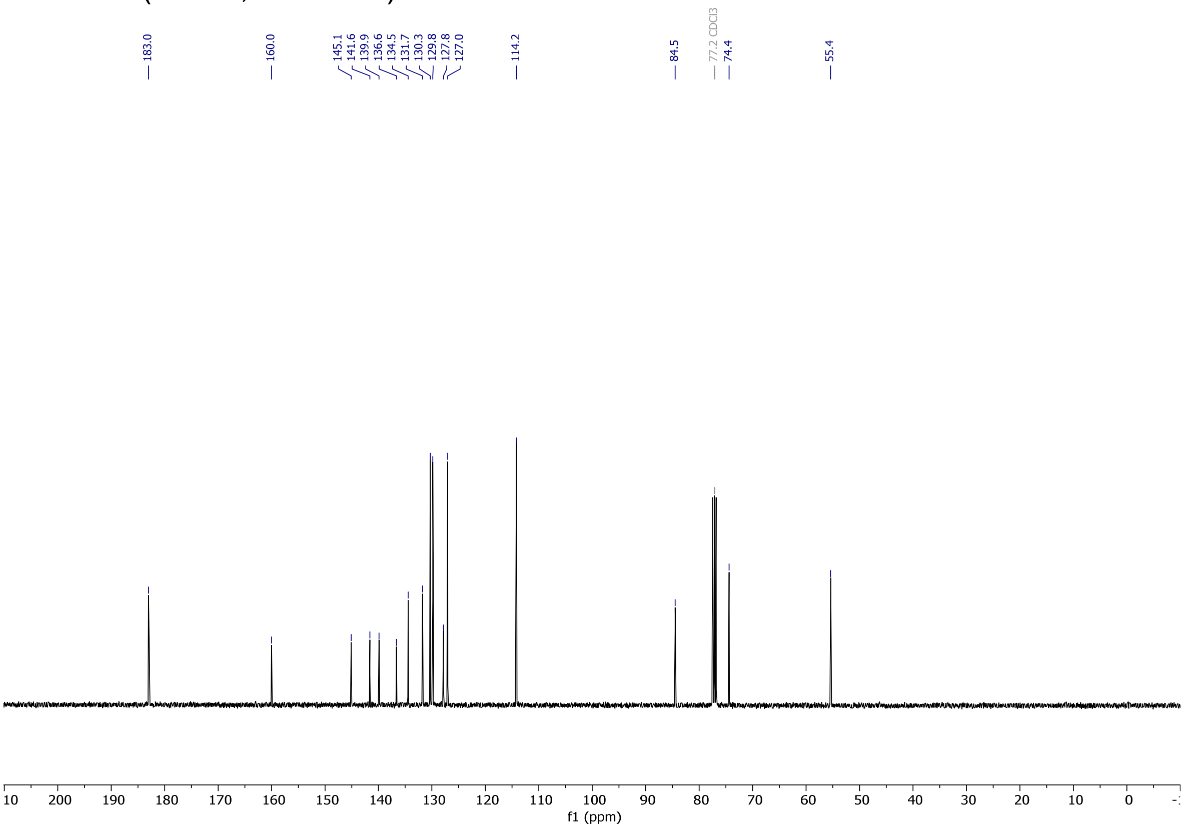

^{1}H NMR ($CDCl_3$, 400 MHz)

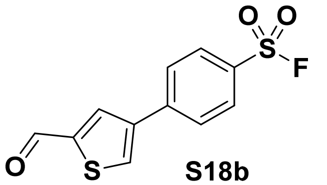

S18b

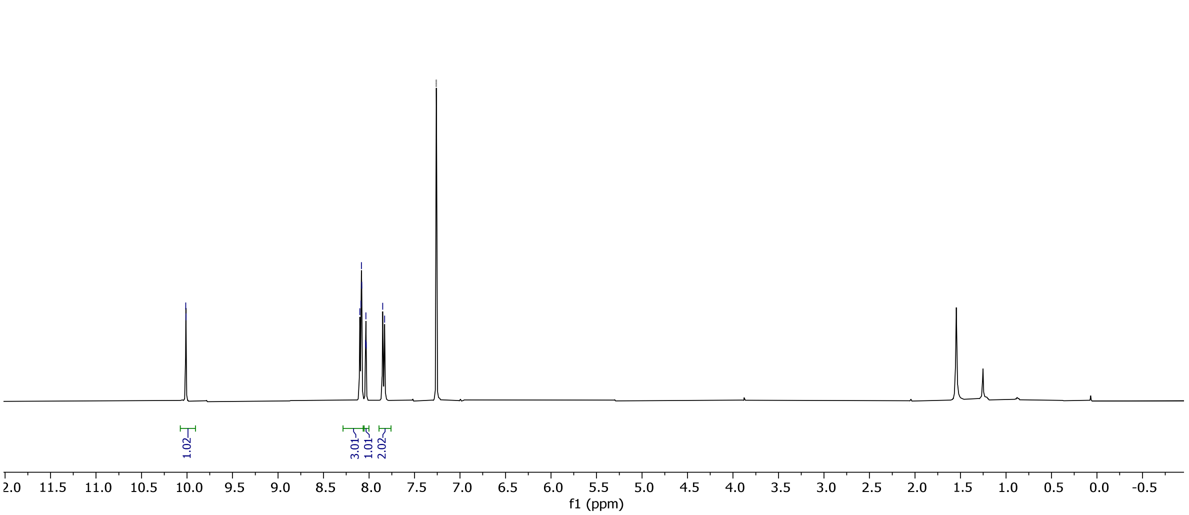

^{13}C NMR ($CDCl_3$; 101 MHz)

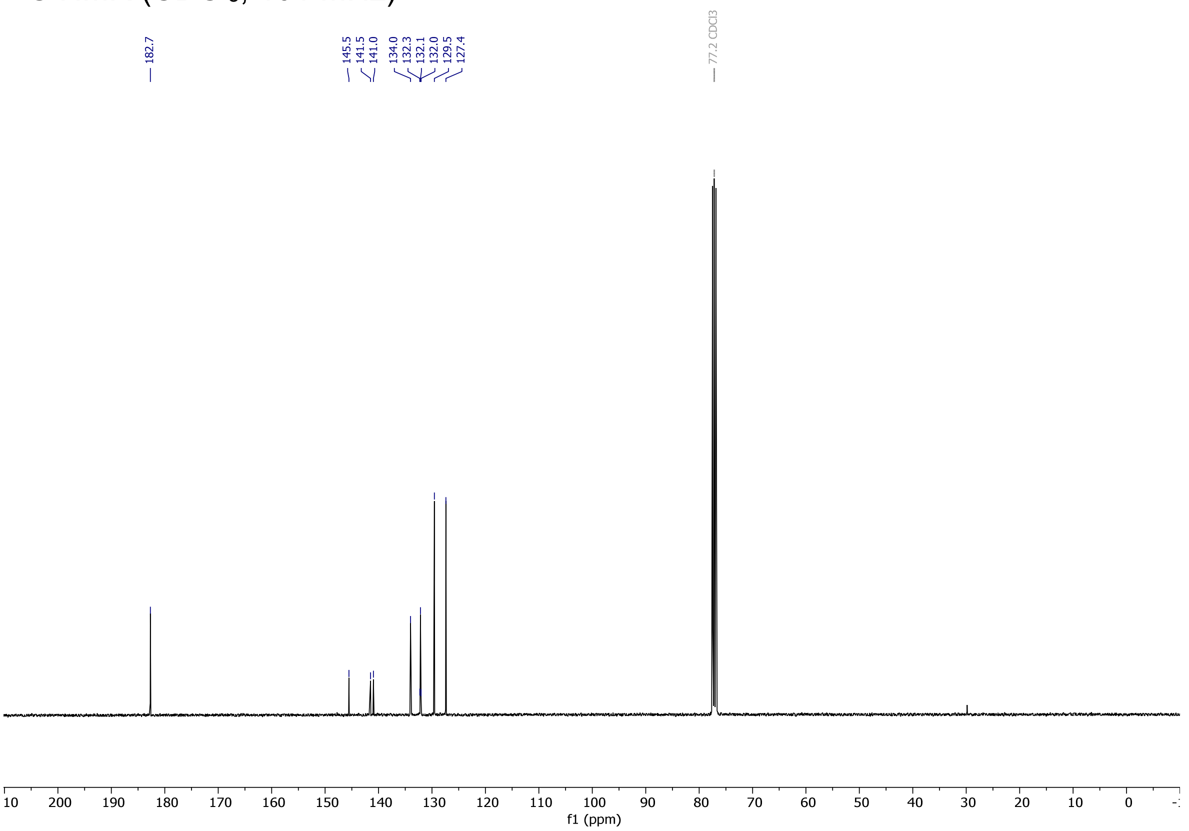

^{19}F NMR ($CDCl_3$; 376 MHz)

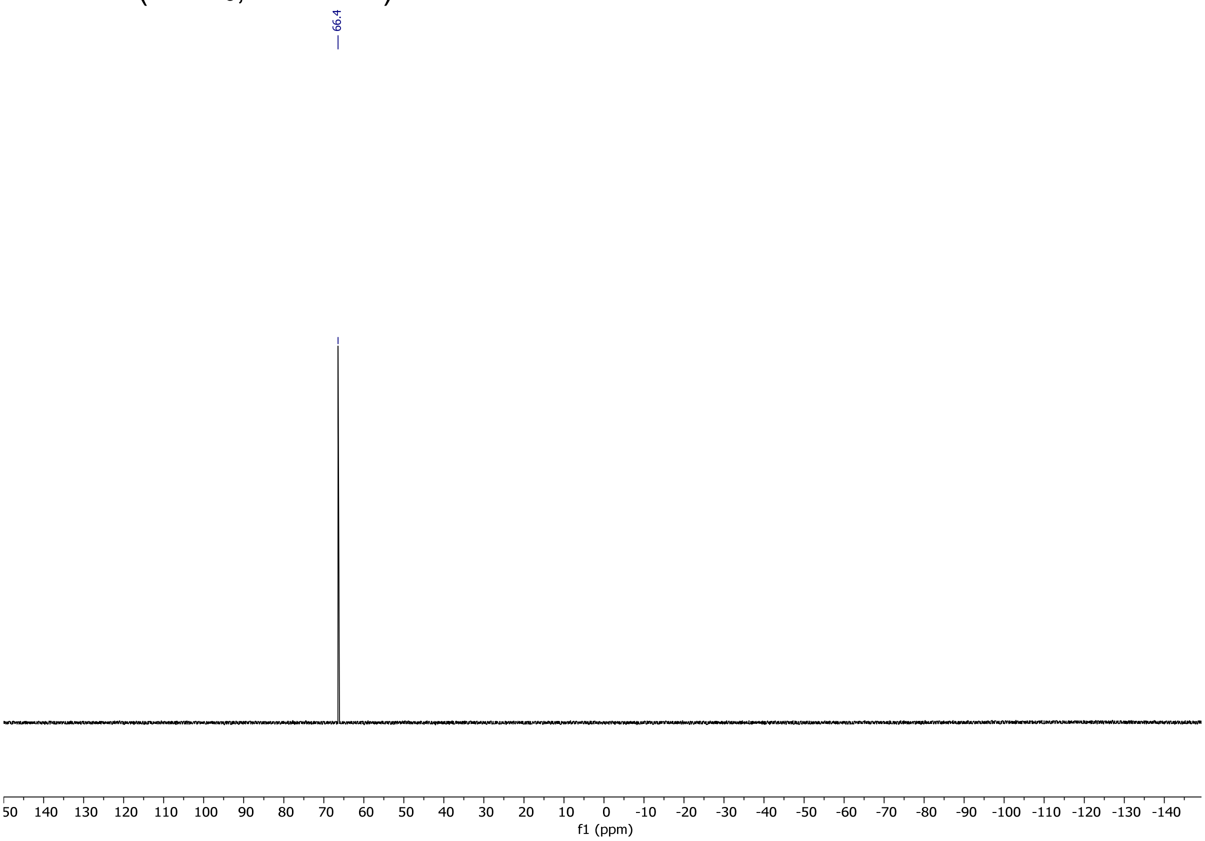

^{1}H NMR ($CDCl_3$, 400 MHz)

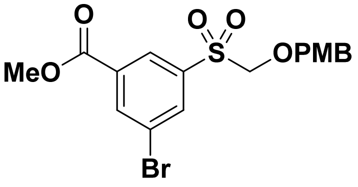

27

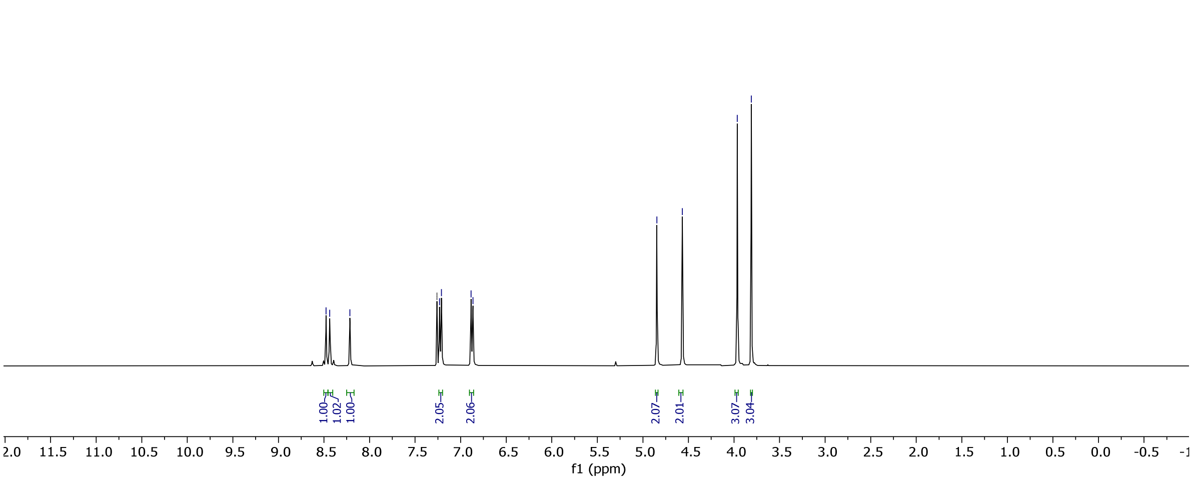

^{13}C NMR ($CDCl_3$; 101 MHz)

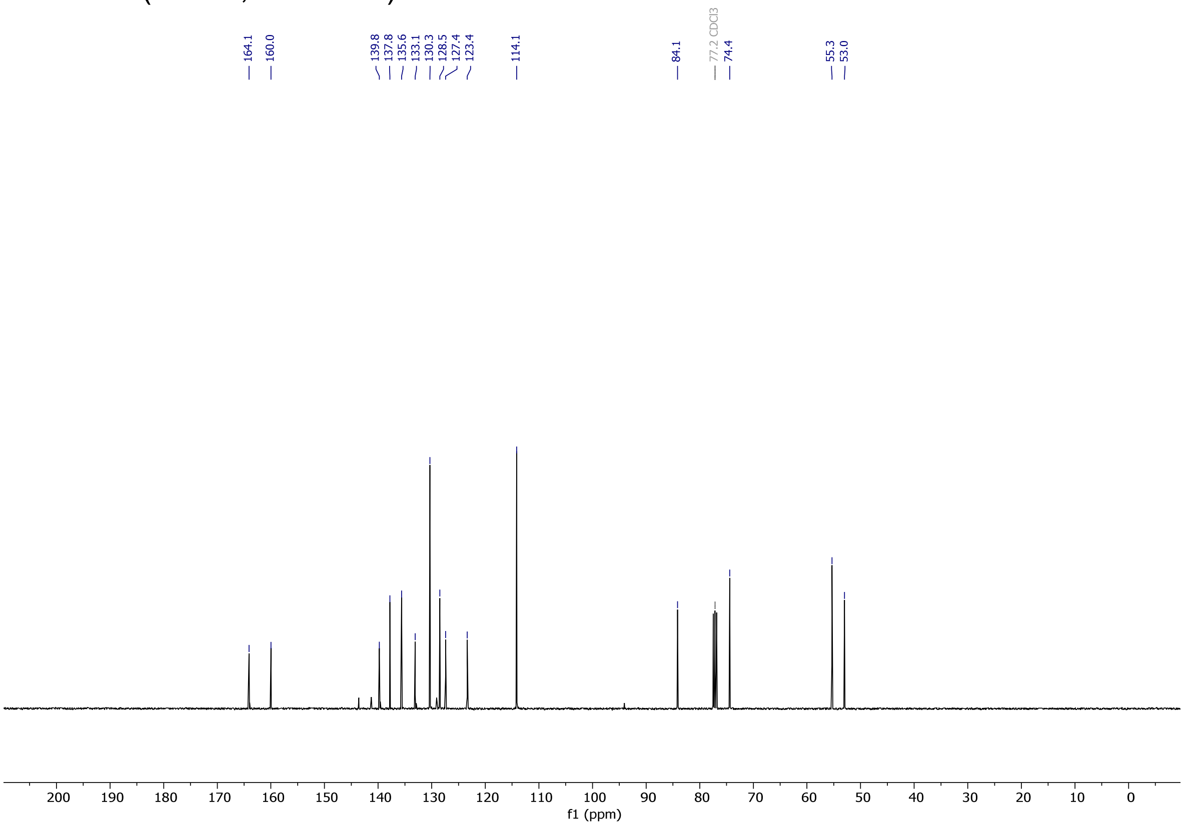

^{1}H NMR ($CDCl_3$, 400 MHz)

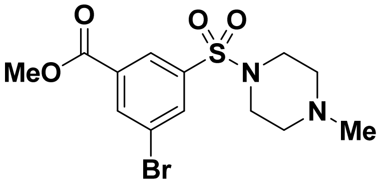

28

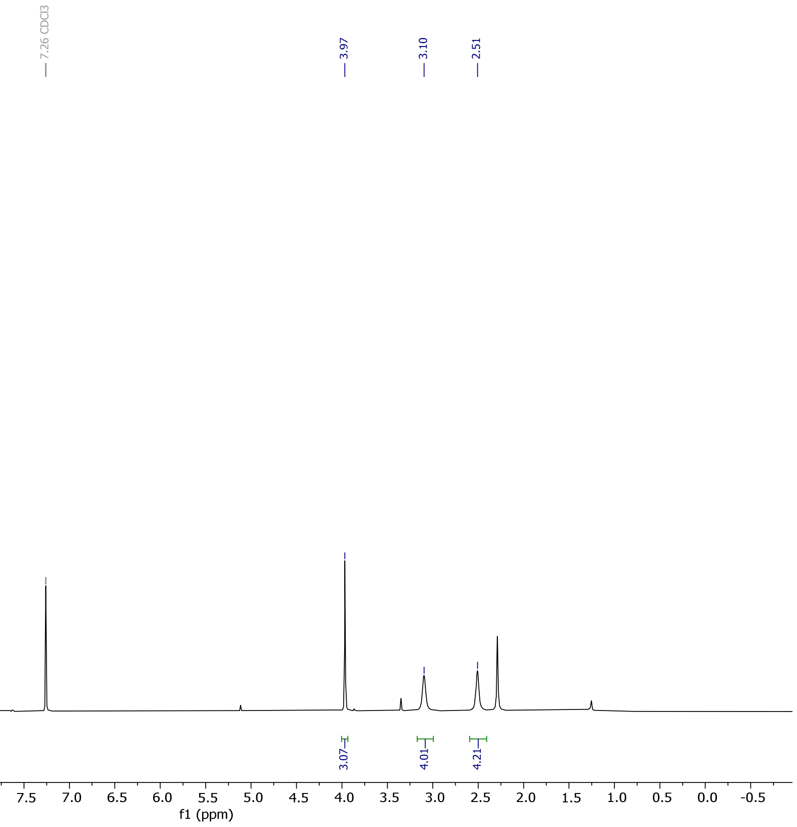

^{13}C NMR ($CDCl_3$; 101 MHz)

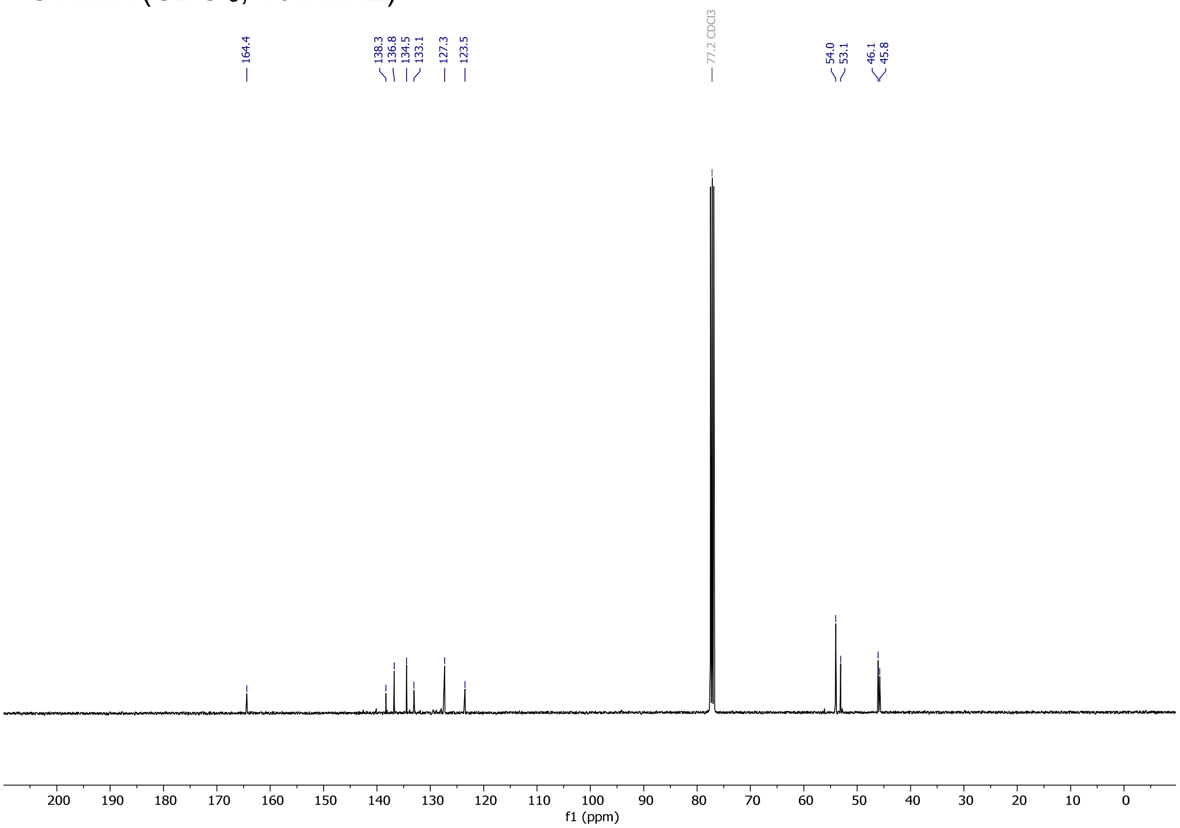

^{1}H NMR ($CDCl_3$, 400 MHz)

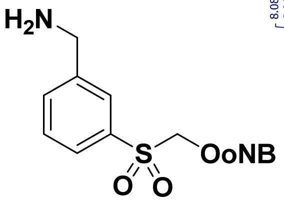

32

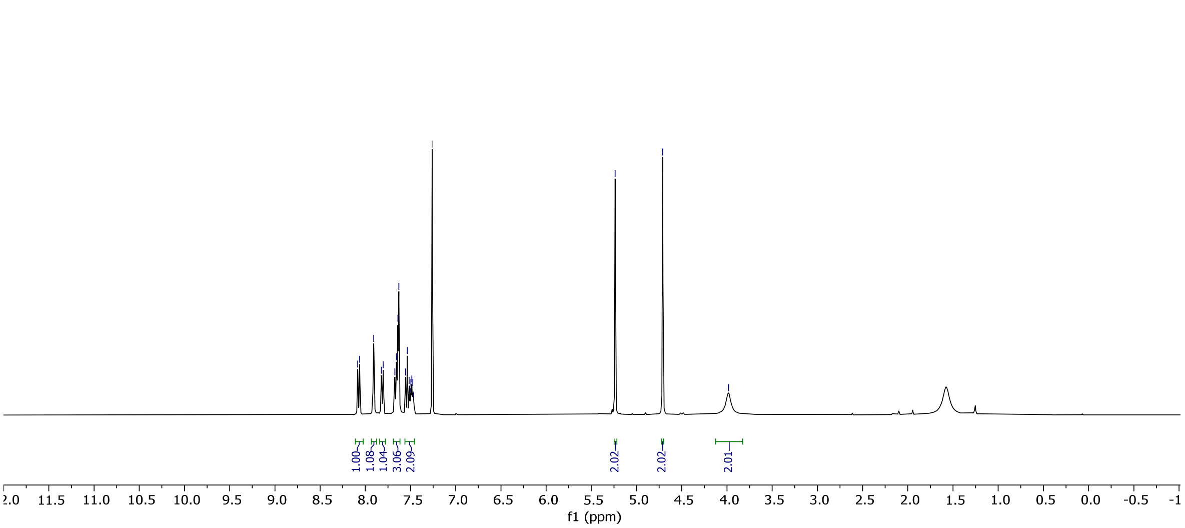

^{13}C NMR ($CDCl_3$; 101 MHz)

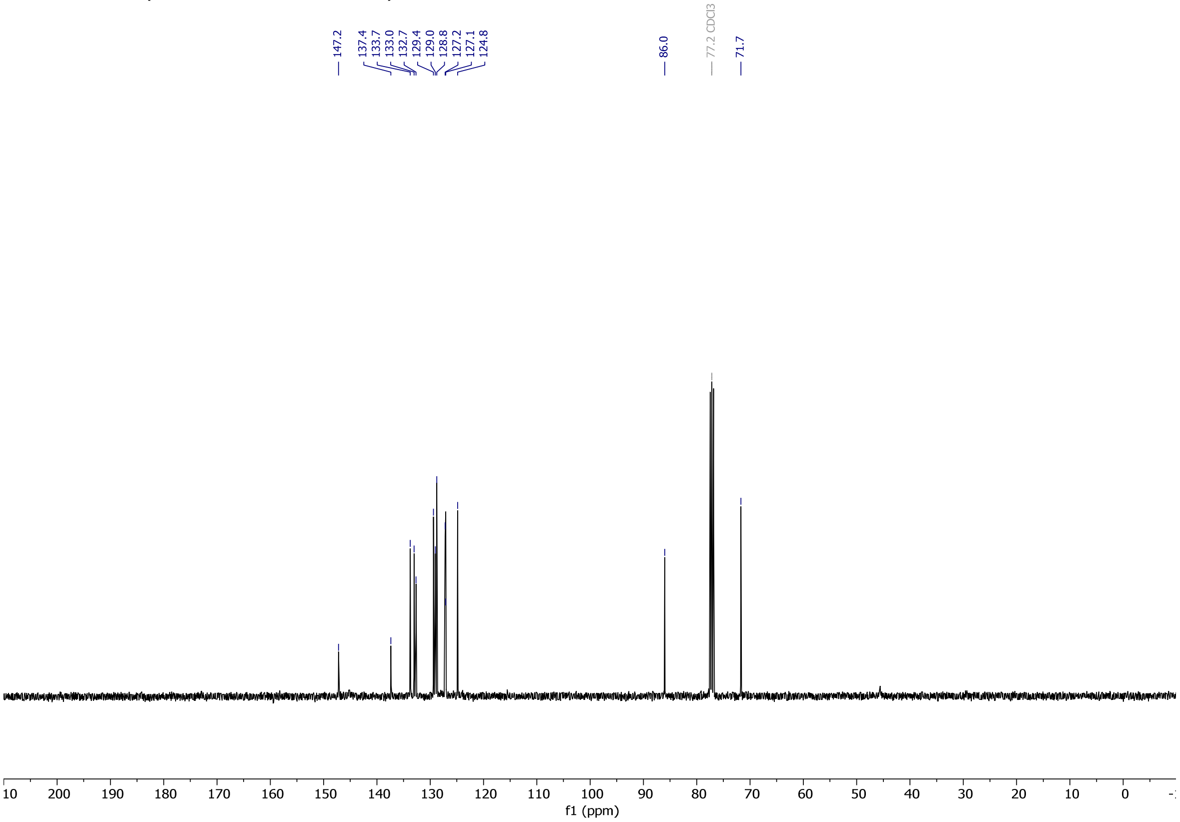

^{1}H NMR ($CDCl_3$, 400 MHz)

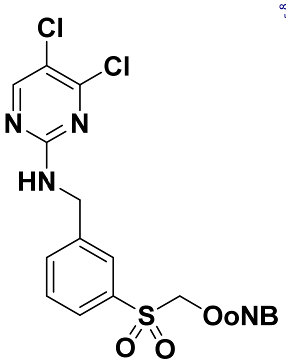

34

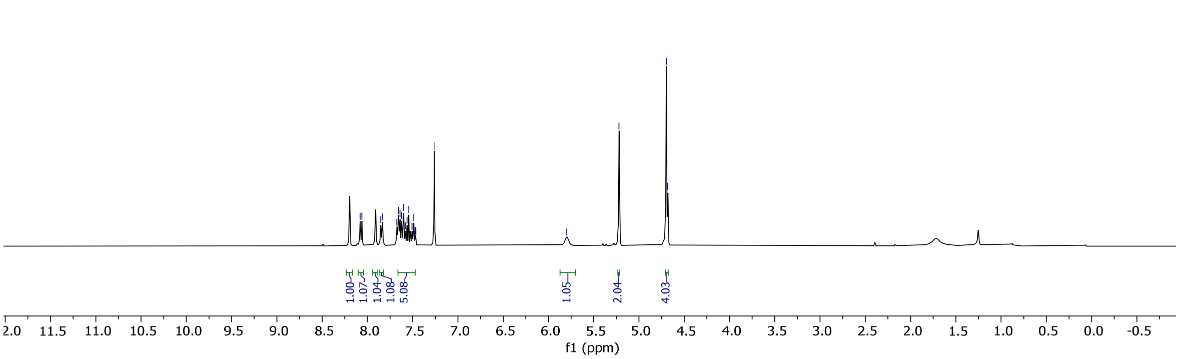

^{13}C NMR ($CDCl_3$; 101 MHz)

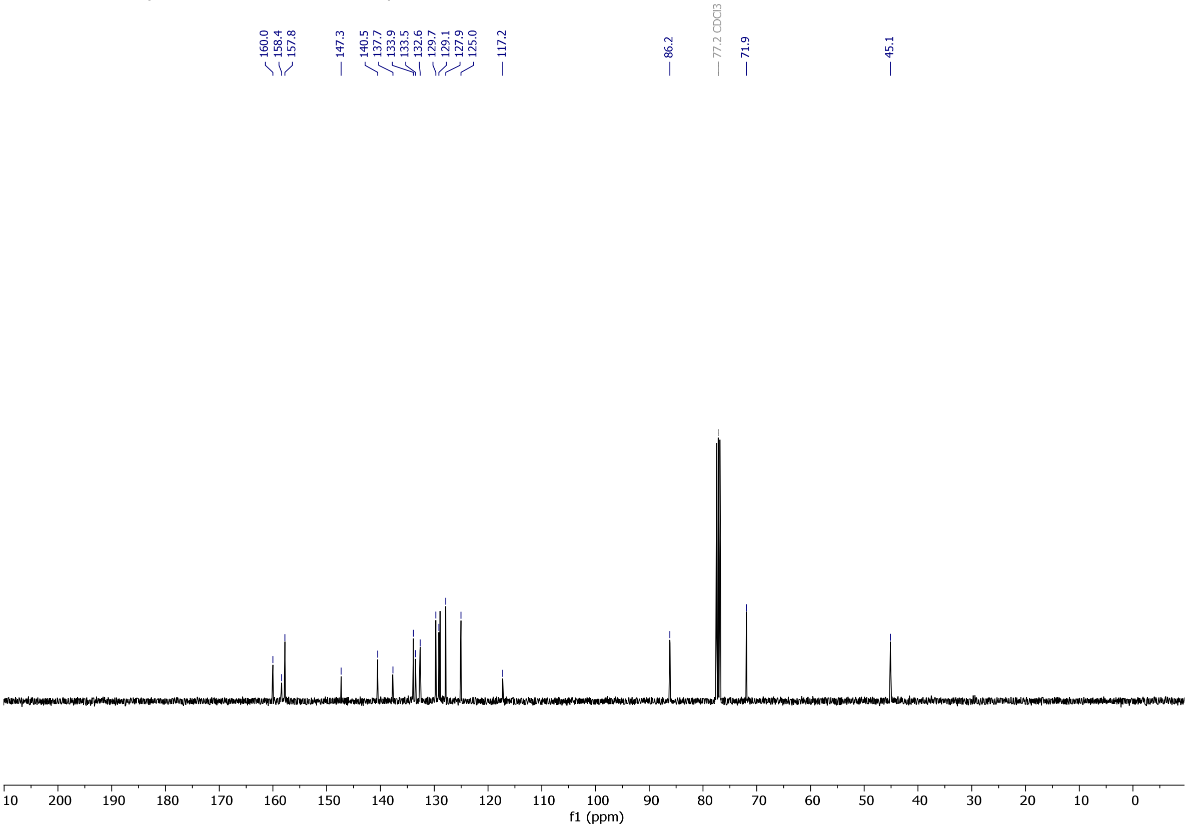

^{1}H NMR ($CDCl_3$, 400 MHz)

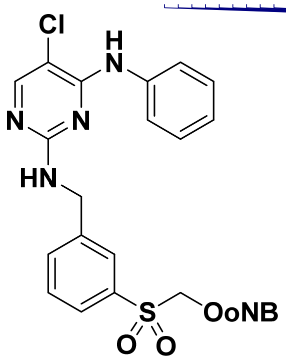

S19

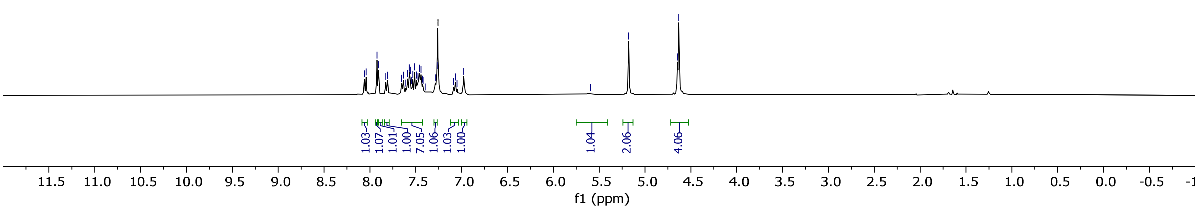

^{13}C NMR ($CDCl_3$; 101 MHz)

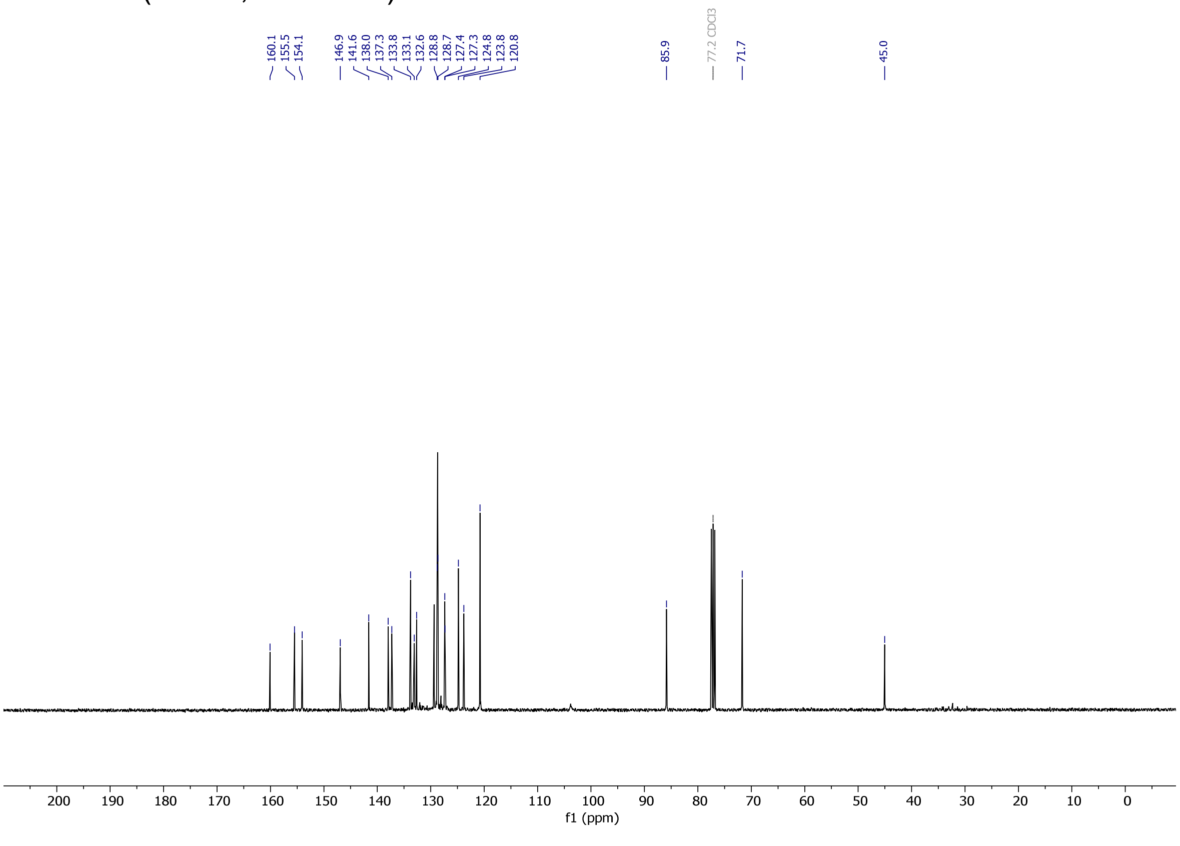

^{1}H NMR ($CDCl_3$, 400 MHz)

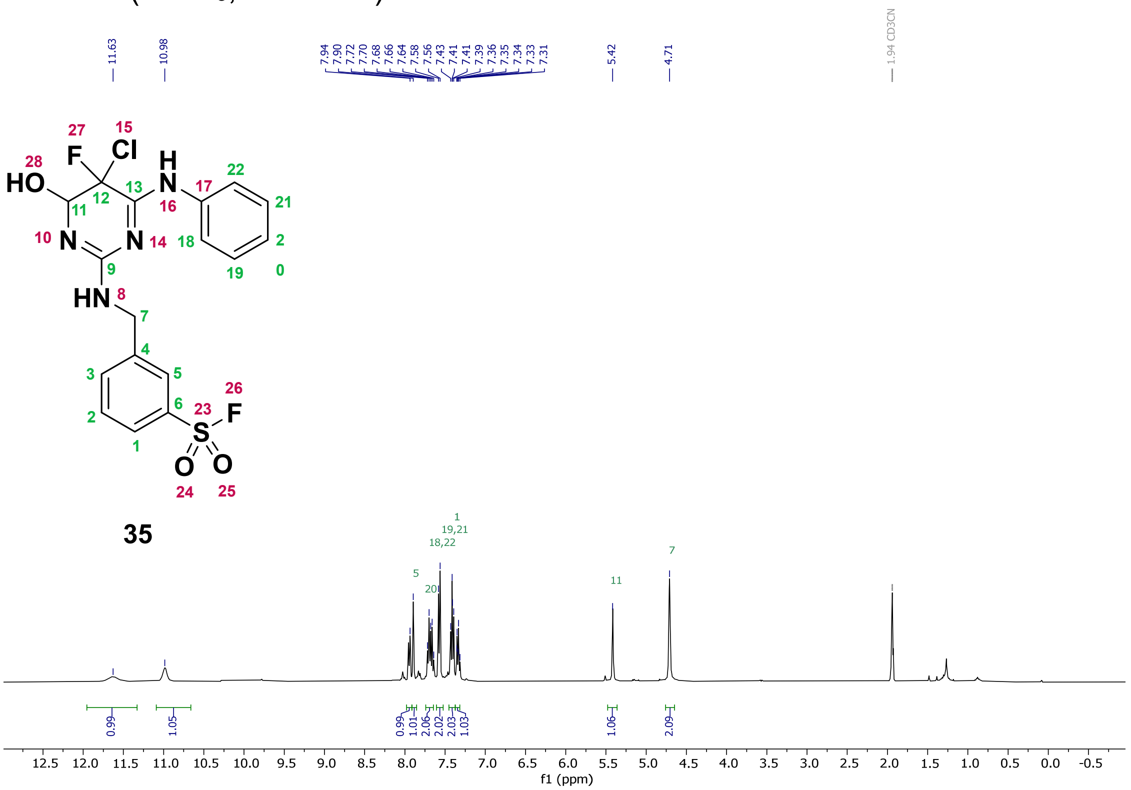

^{13}C NMR ($CDCl_3$; 101 MHz)

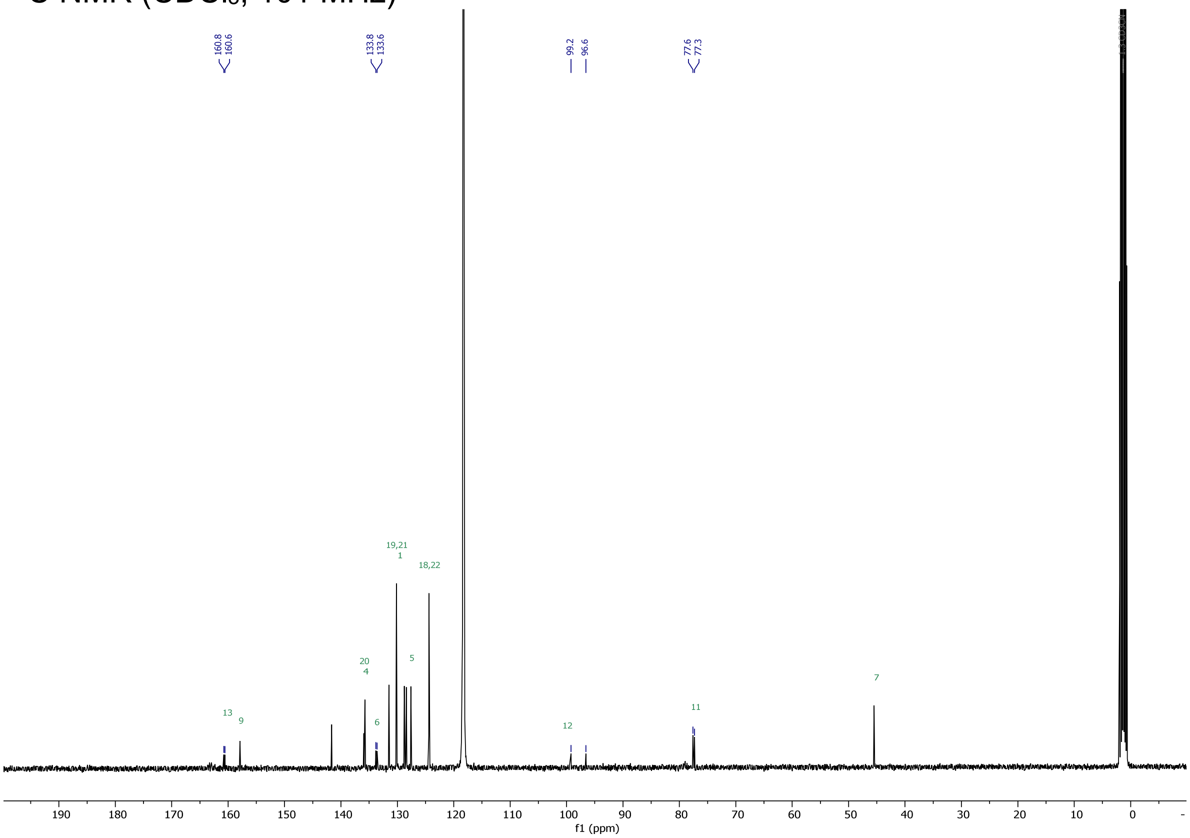

^{19}F NMR ($CDCl_3$; 376 MHz)

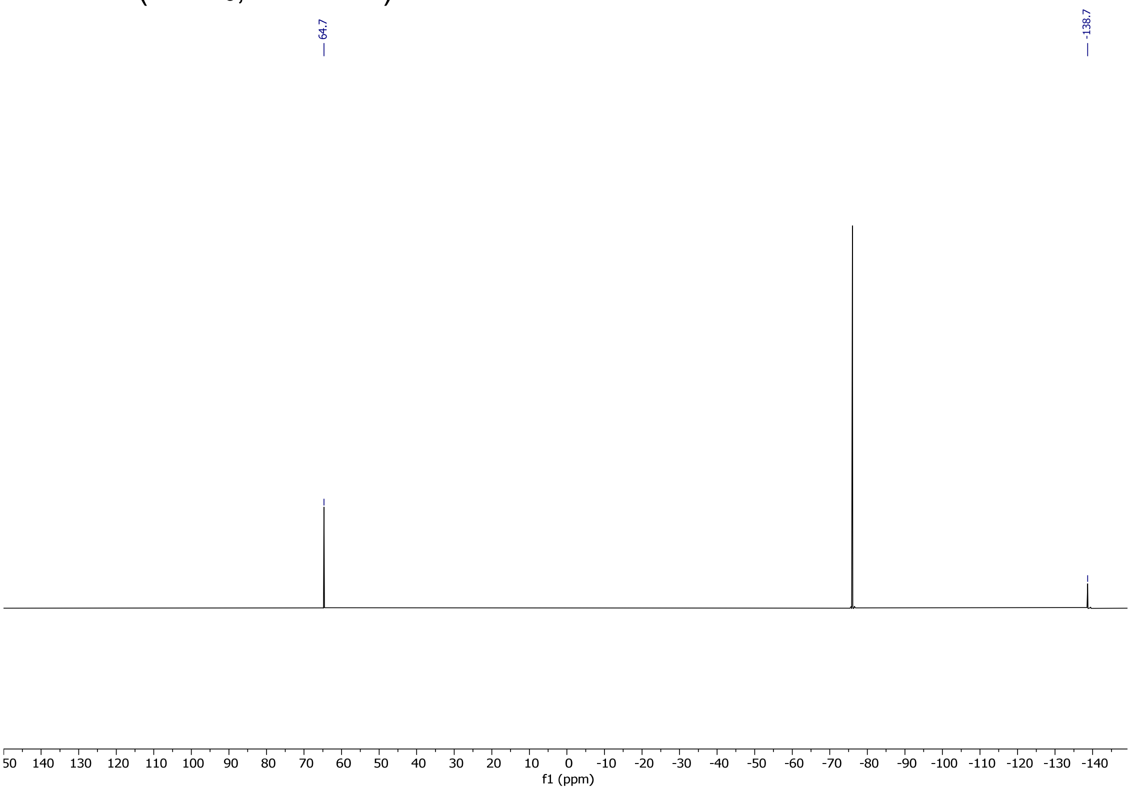

COSY (*f1*: 400 MHz, *f2*: 400 MHz)

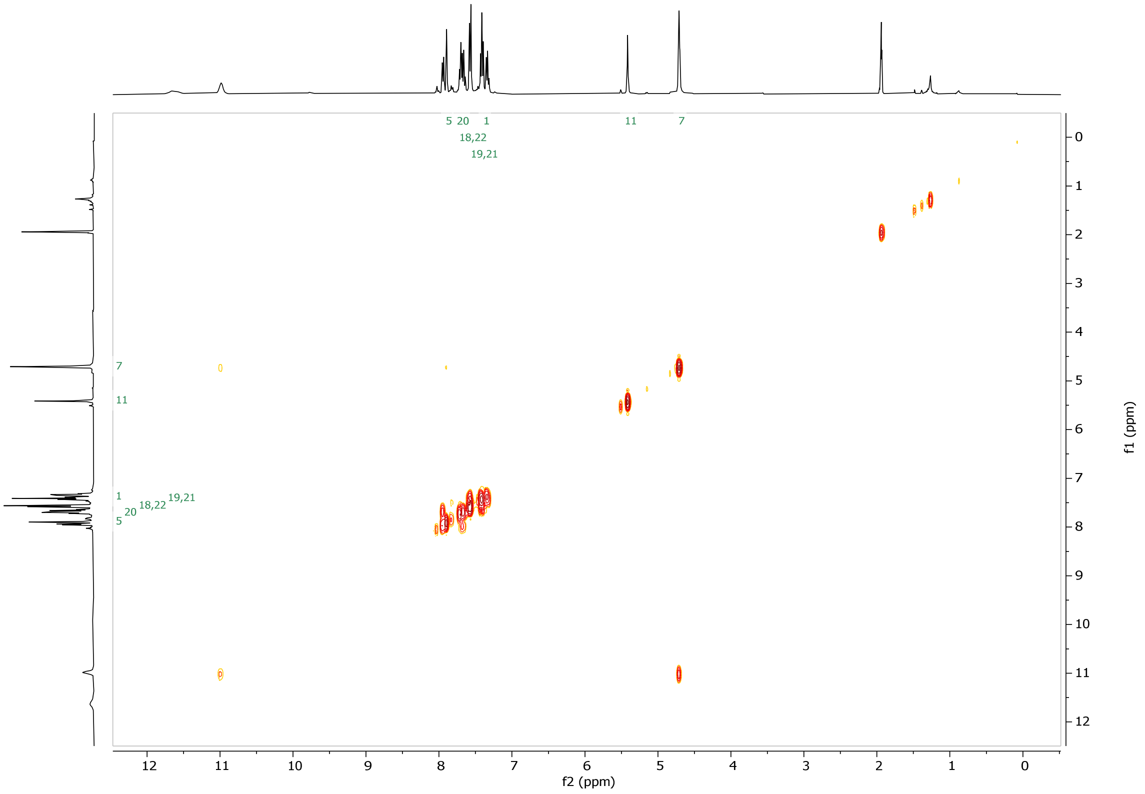

DEPT-135 ($CDCl_3$; 101 MHz)

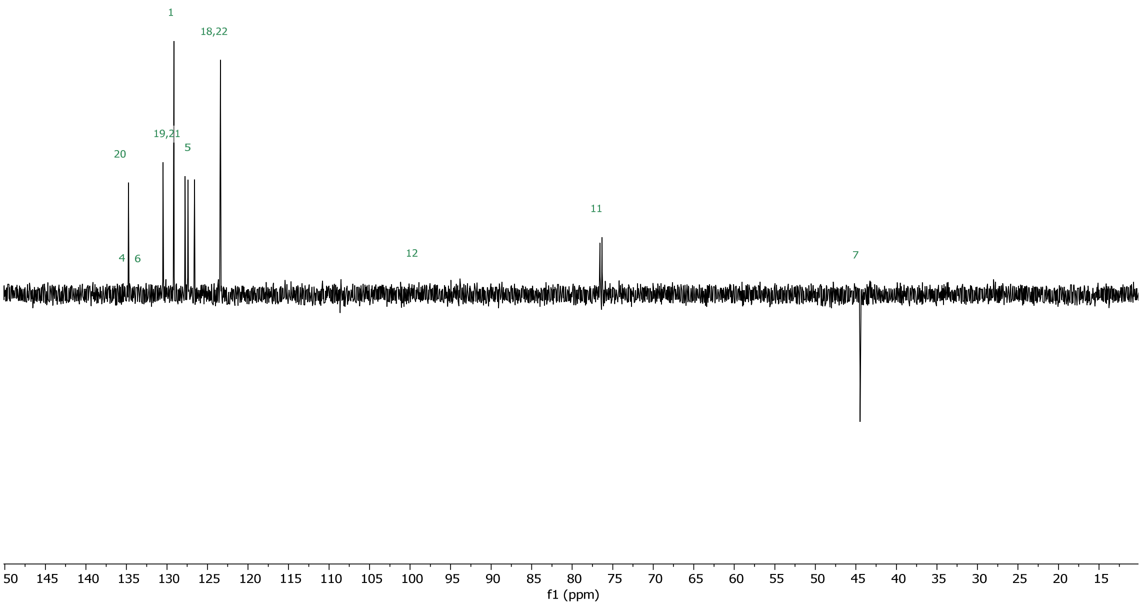

DEPT-90 ($CDCl_3$; 101 MHz)

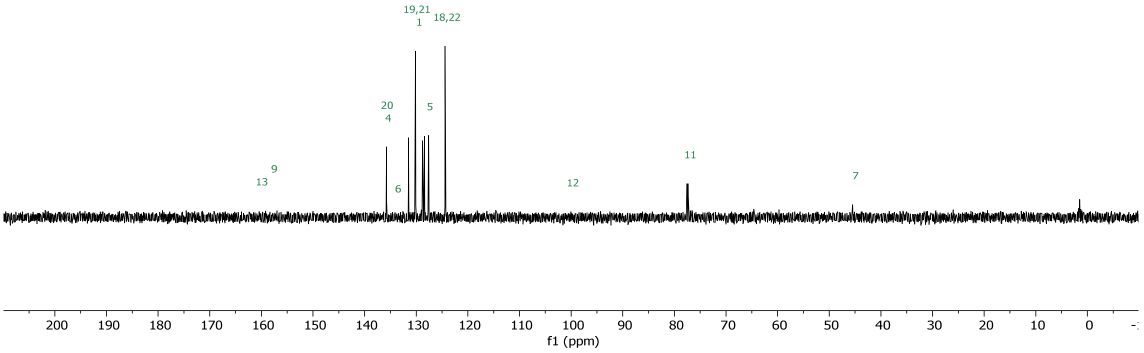

HSQC (*f1*: 101 MHz, *f2*: 400 MHz)

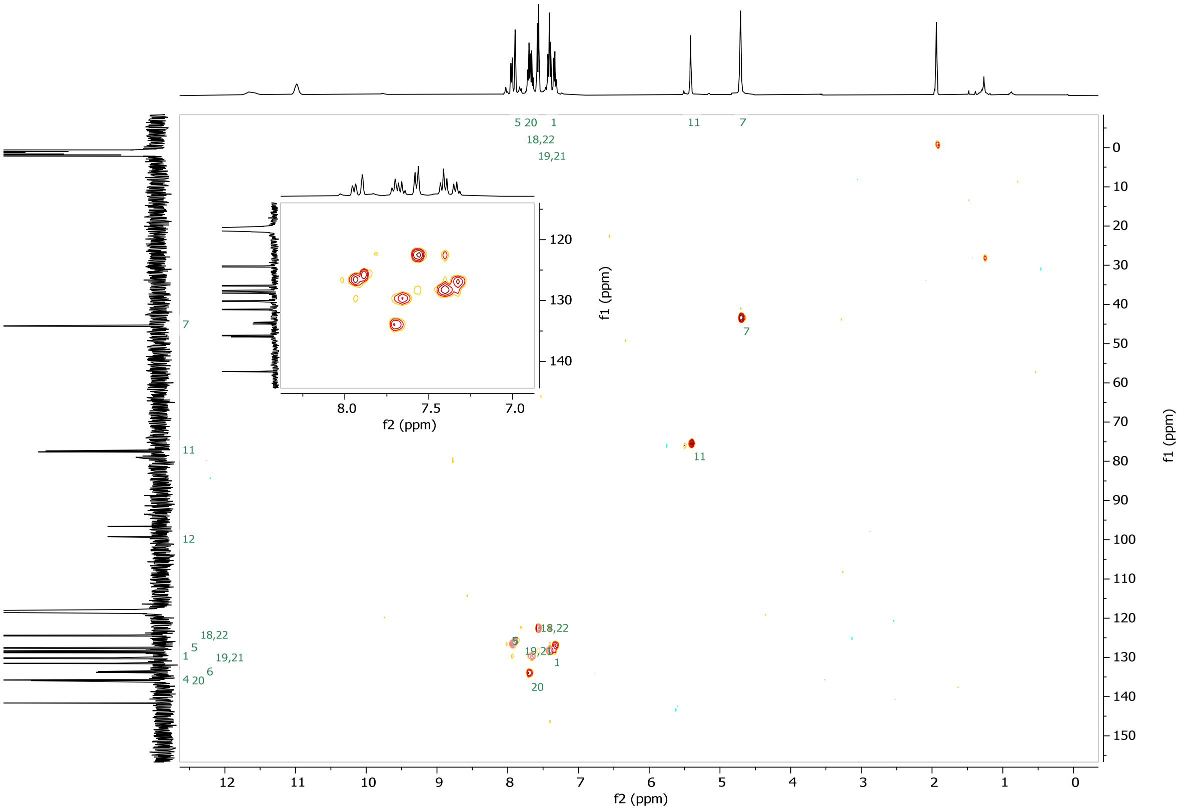

HMBC (*f1*: 101 MHz, *f2*: 400 MHz)

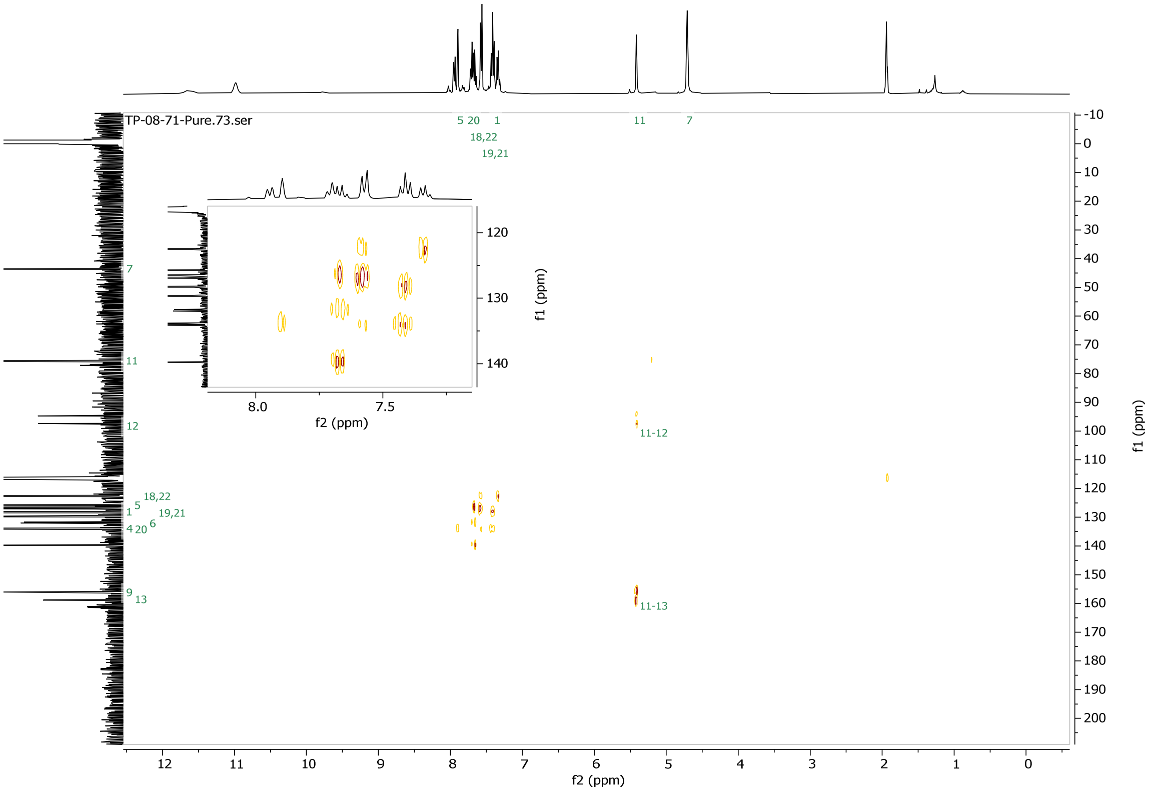

^1H NMR ($CDCl_3$, 400 MHz)

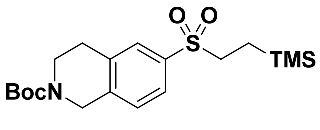

42

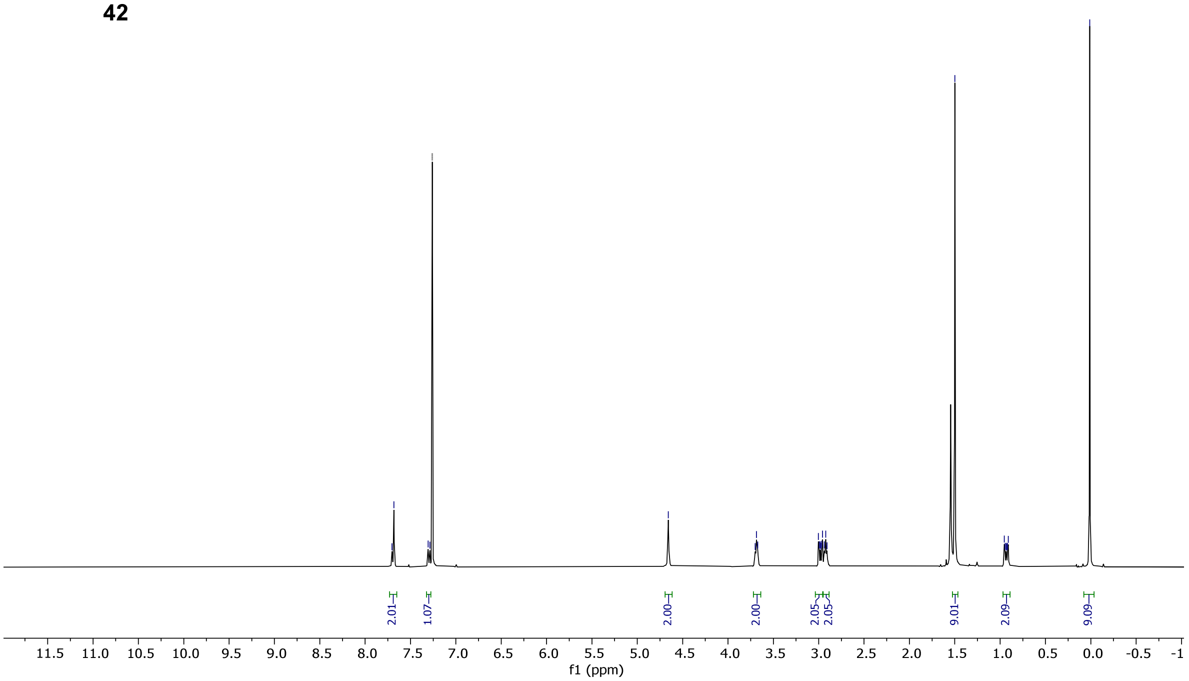

^{13}C NMR ($CDCl_3$; 101 MHz)

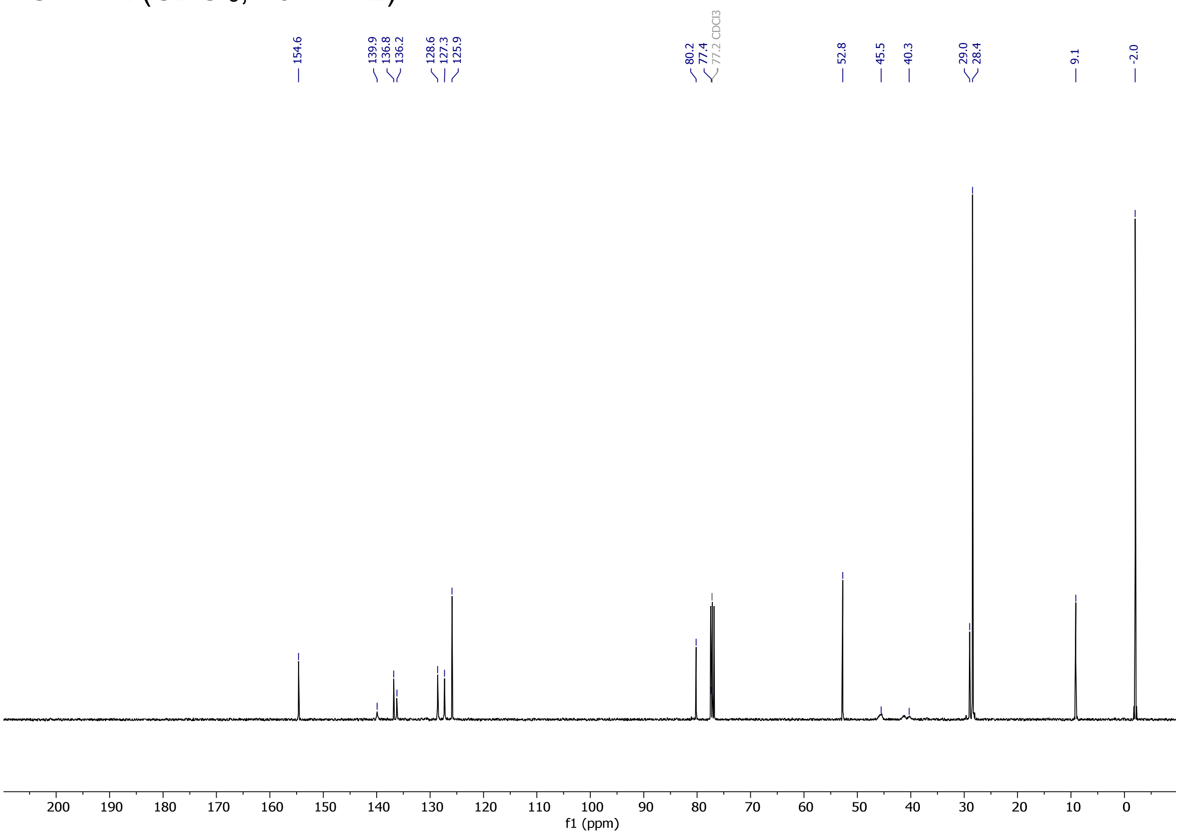

^{1}H NMR ($CDCl_3$, 400 MHz)

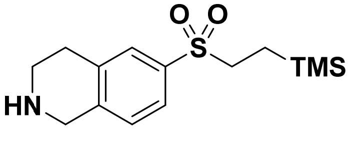

S20

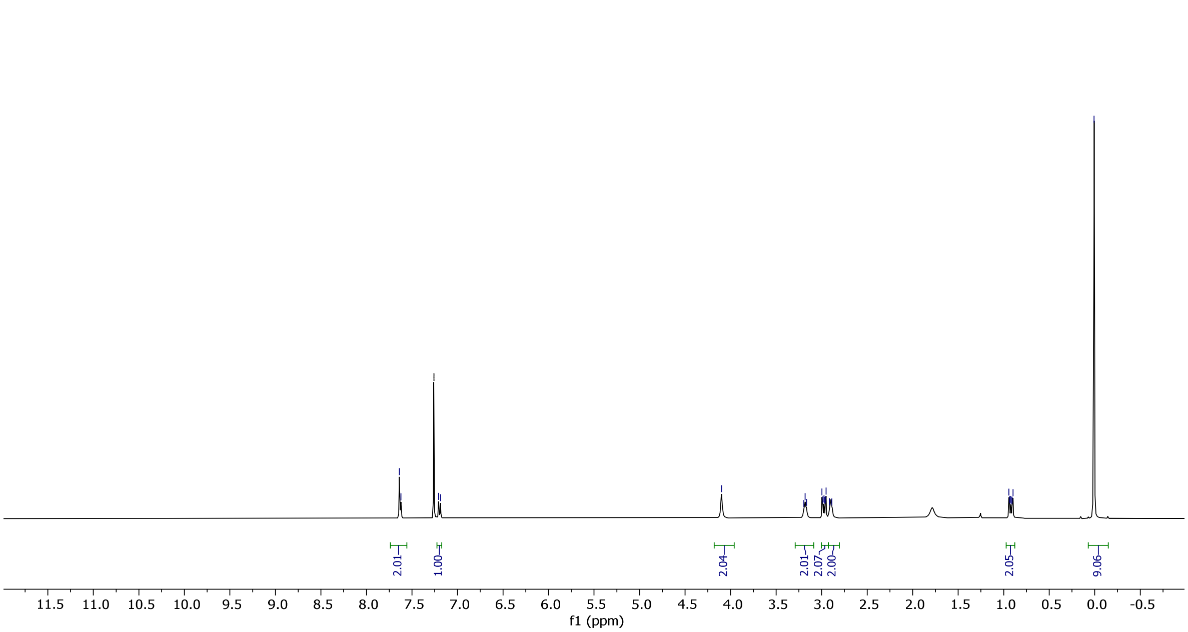

^{13}C NMR ($CDCl_3$; 101 MHz)

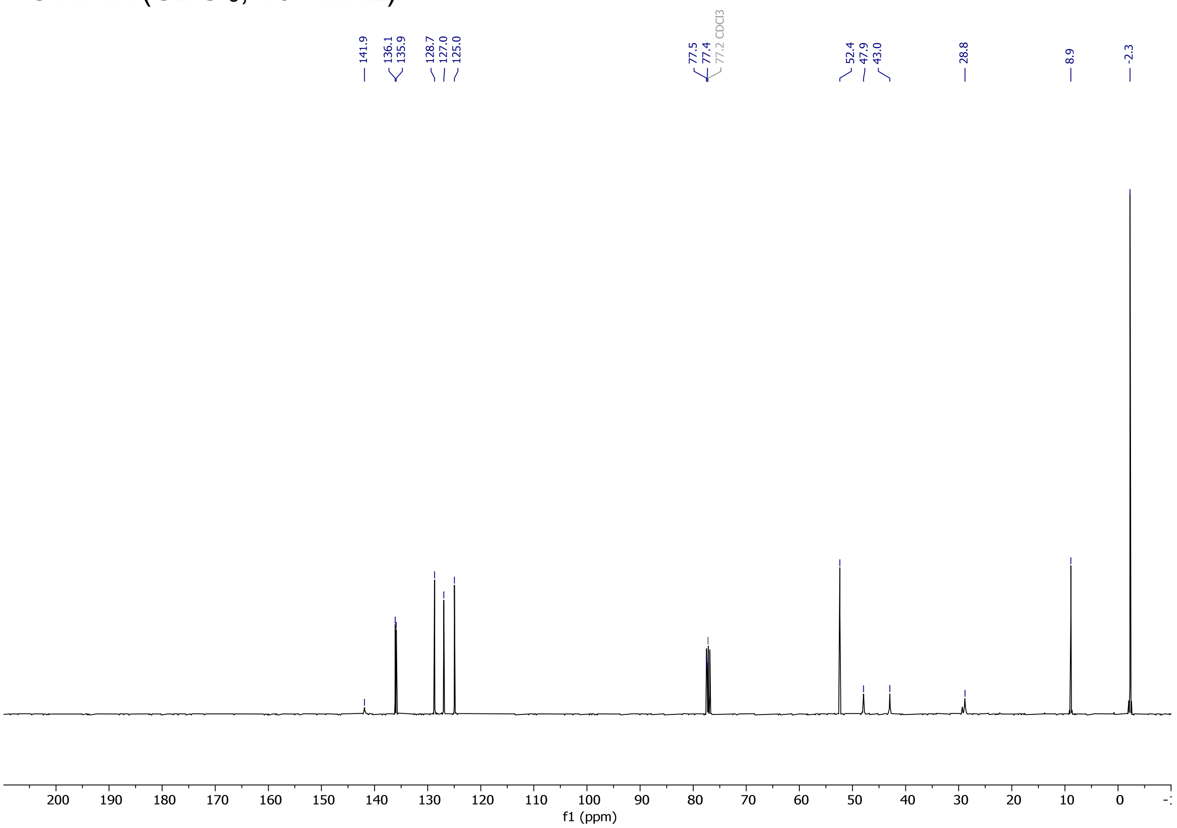

^{1}H NMR ($CDCl_3$, 400 MHz)

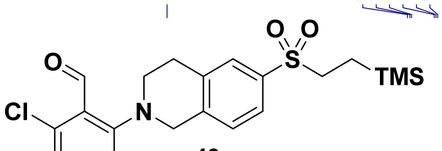

43

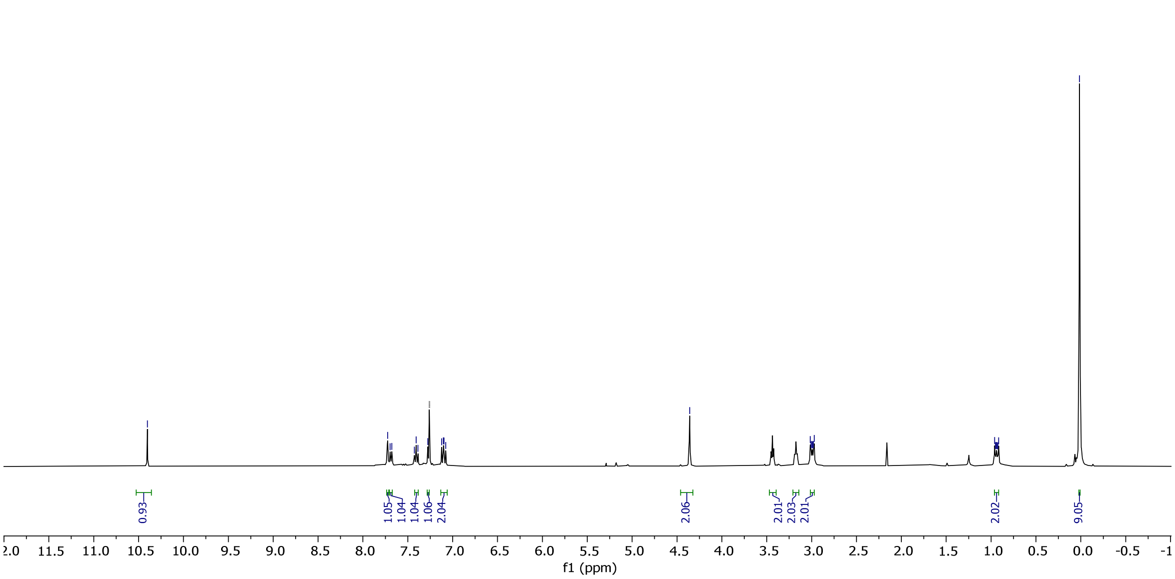

^{13}C NMR ($CDCl_3$; 101 MHz)

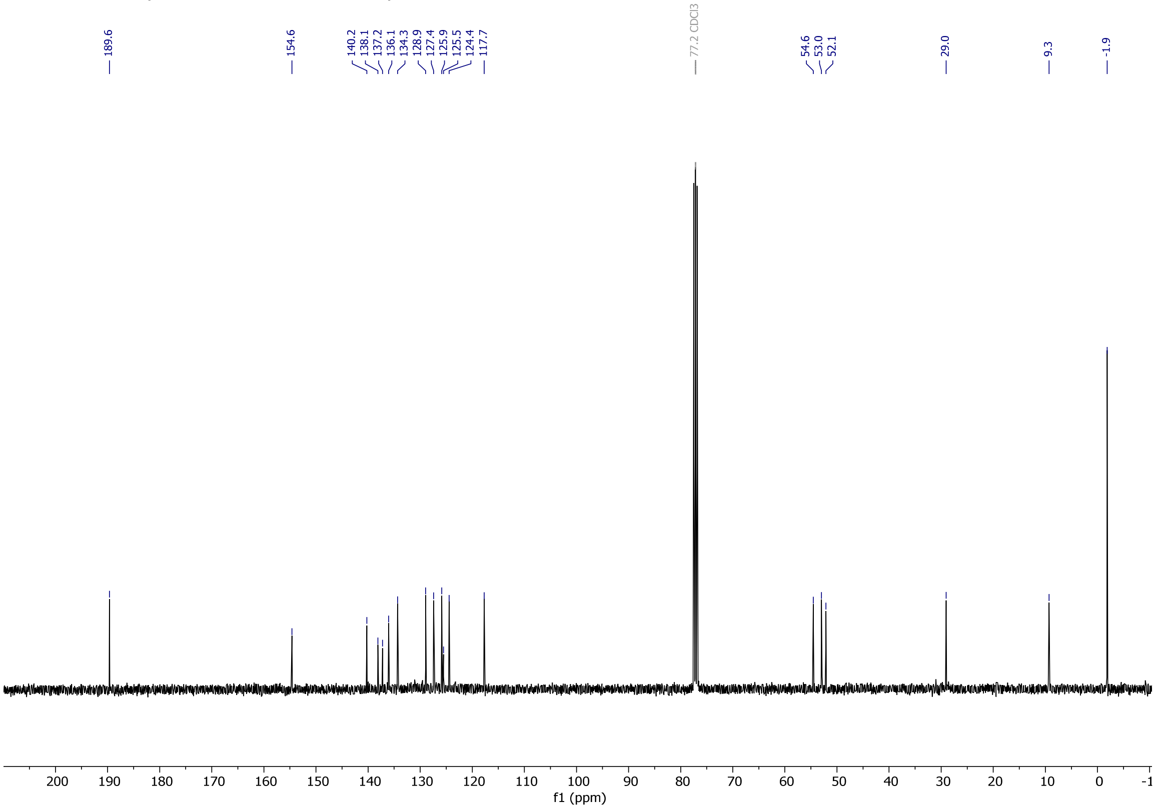

^{1}H NMR ($CDCl_3$, 400 MHz)

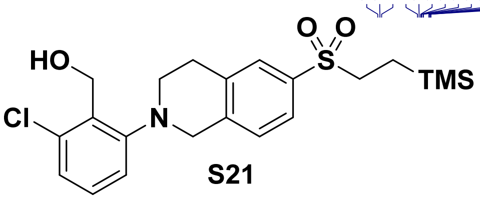

S21

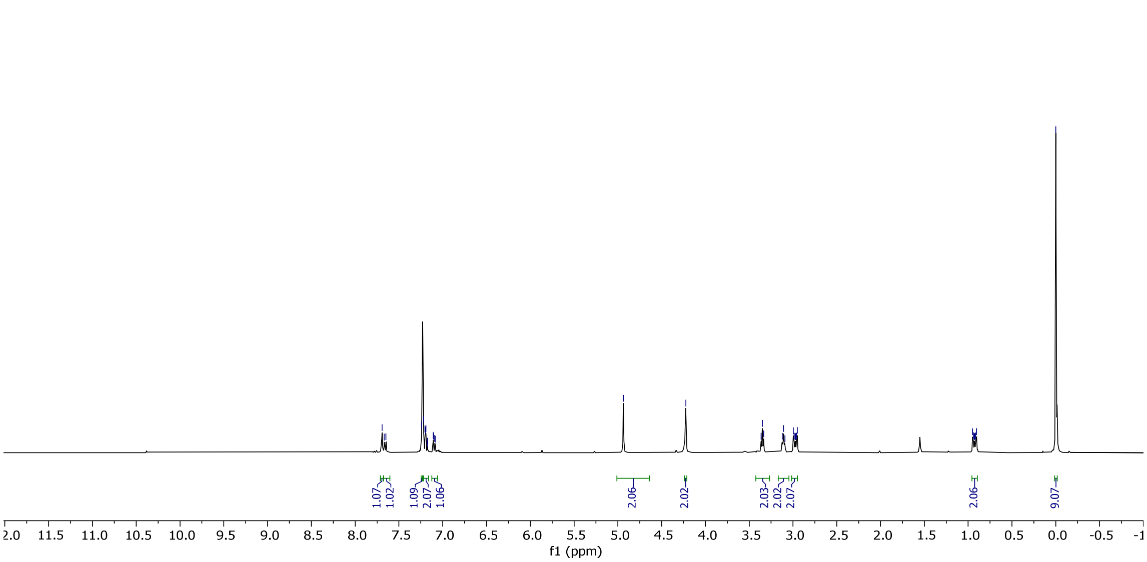

^{13}C NMR ($CDCl_3$; 101 MHz)

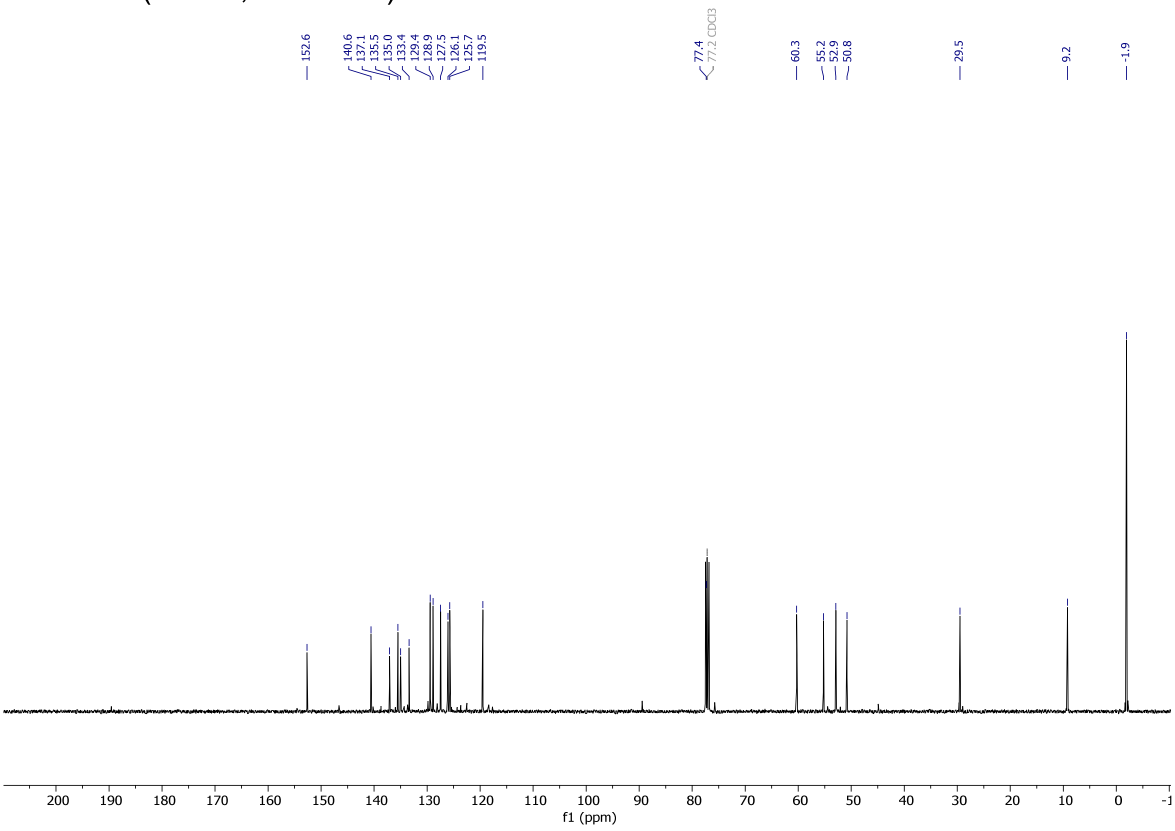

^{1}H NMR ($CDCl_3$, 400 MHz)

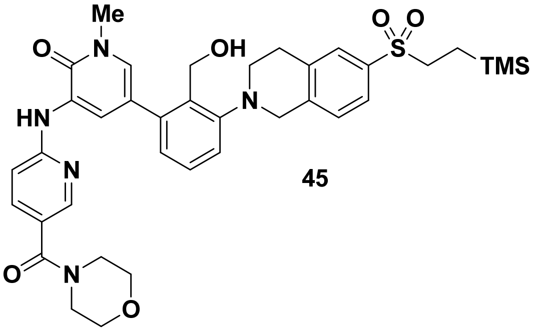

45

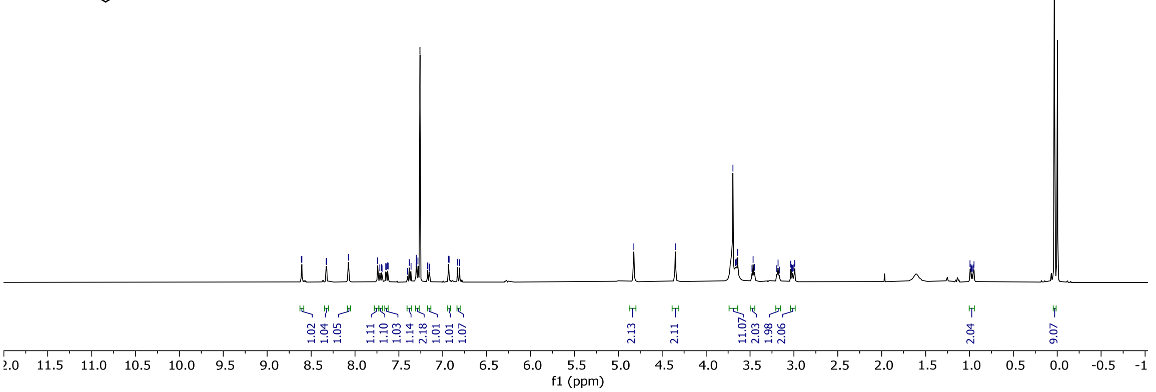

^{13}C NMR ($CDCl_3$; 101 MHz)

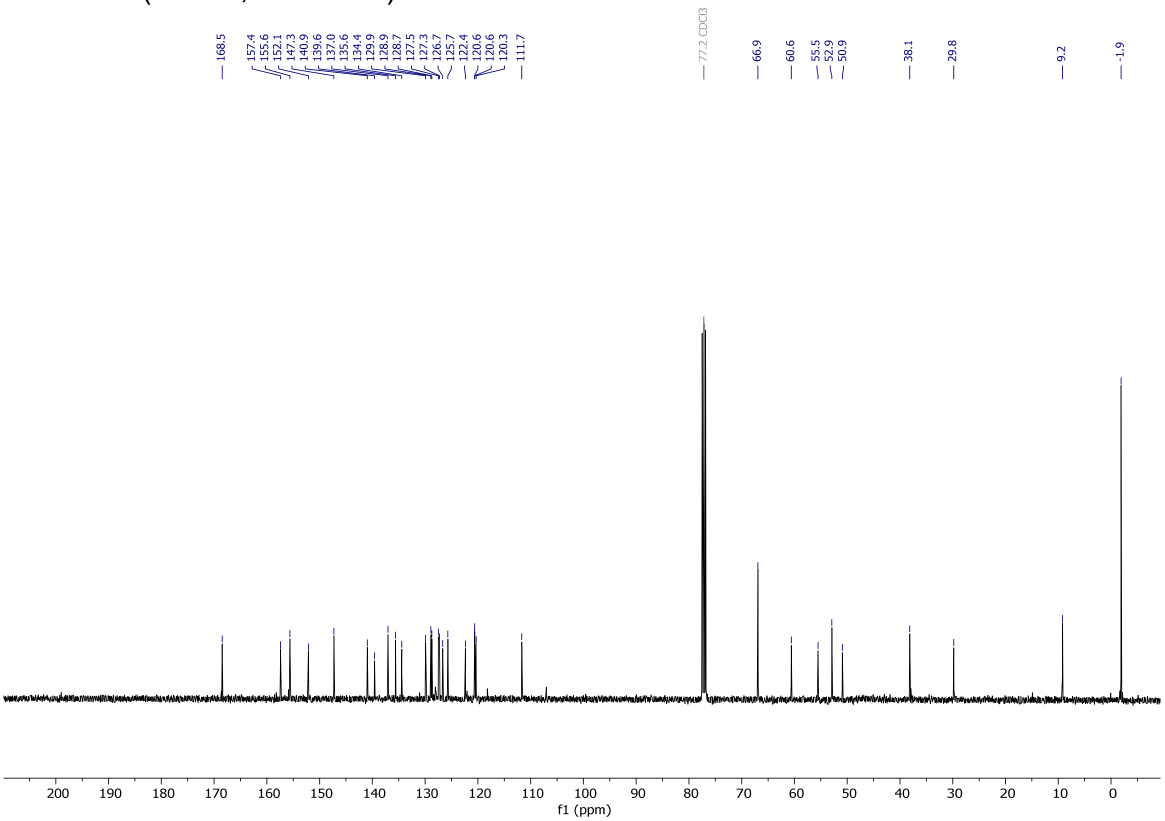

COSY ($f1$: 400 MHz, $f2$: 400 MHz)

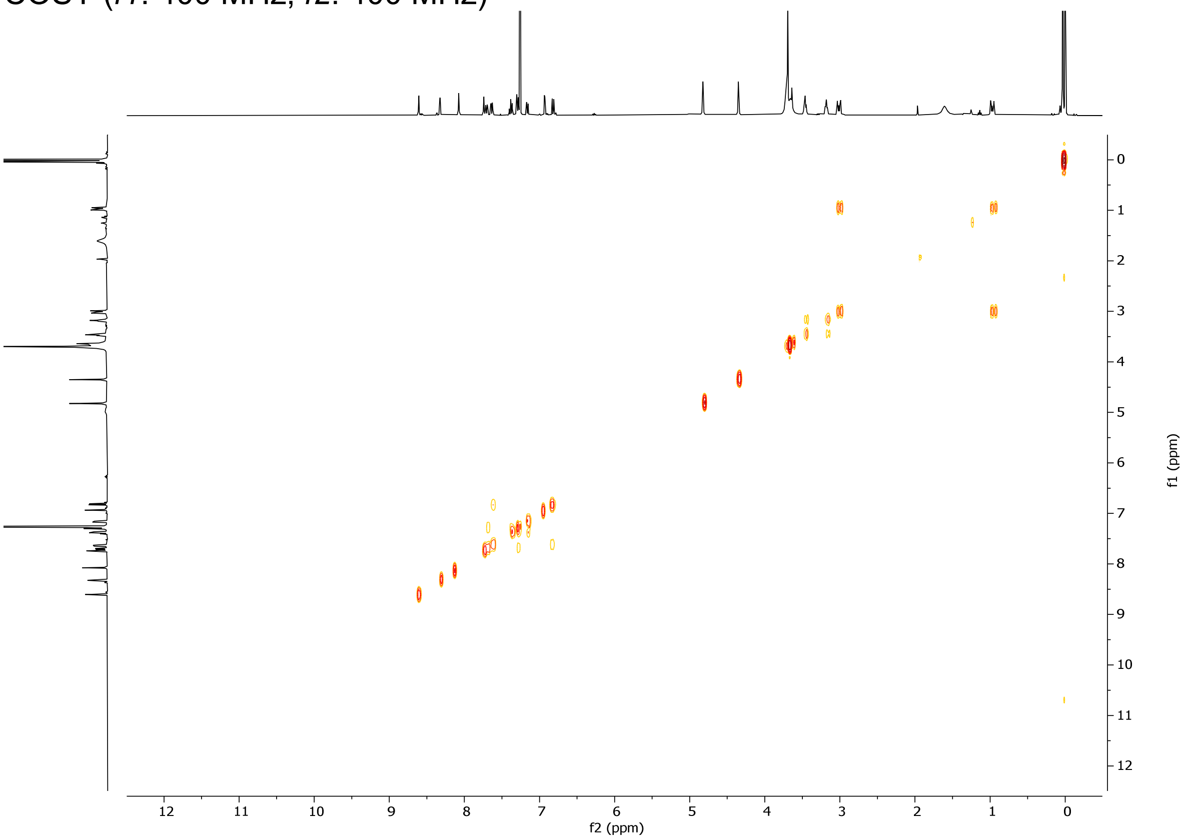

HSQC ($f1$: 101 MHz, $f2$: 400 MHz)

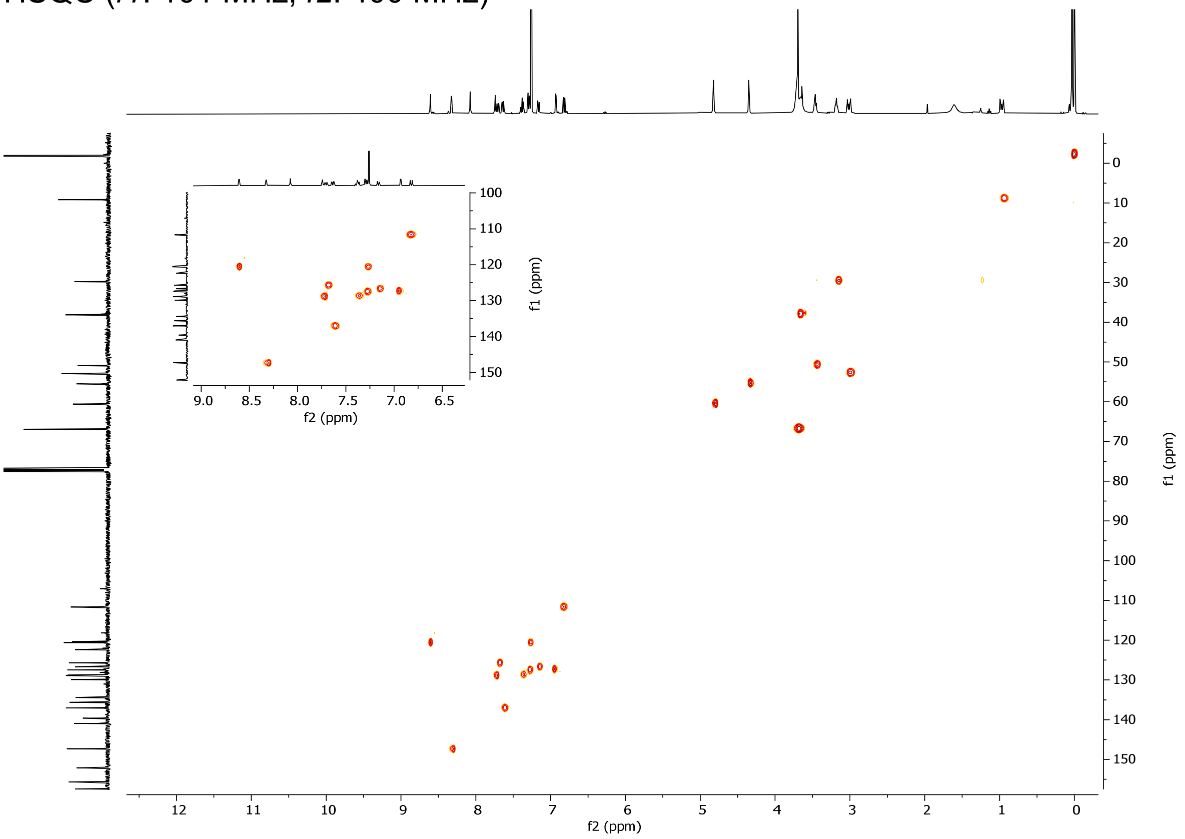

DEPT-135 ($CDCl_3$; 101 MHz)

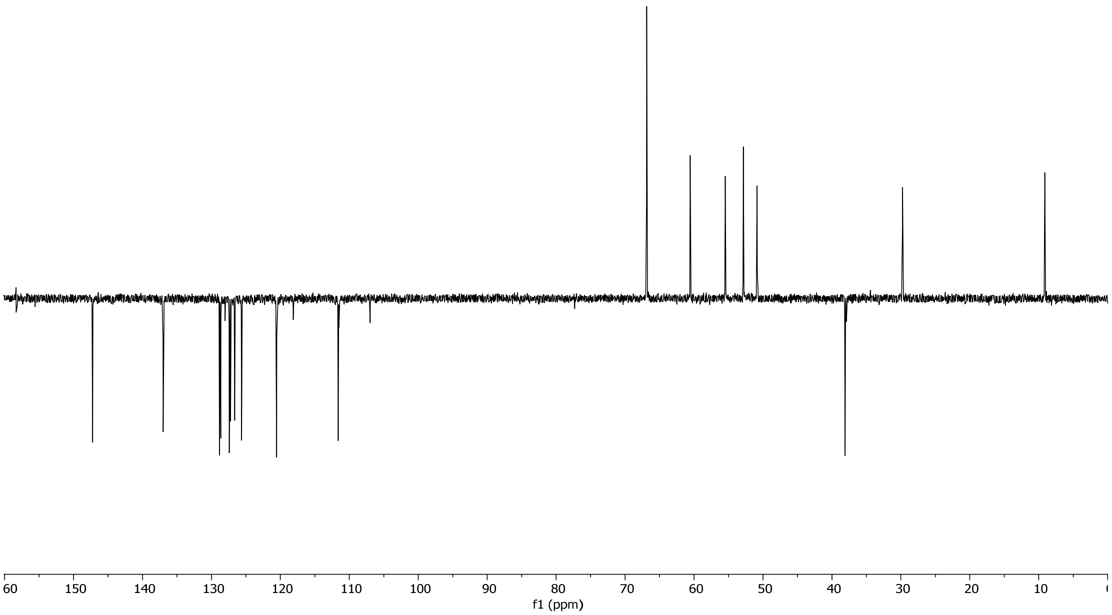

DEPT-90 ($CDCl_3$; 101 MHz)

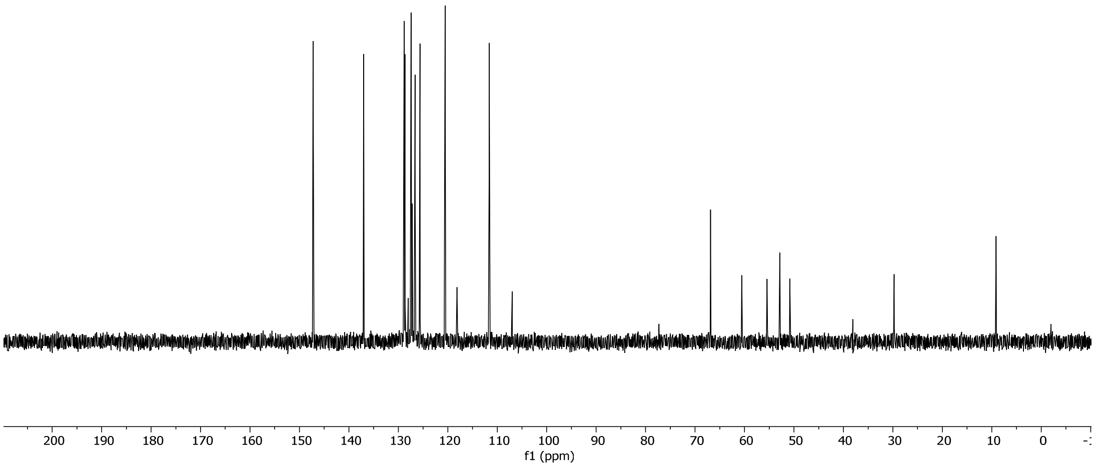

^{1}H NMR ($CDCl_3$, 400 MHz)

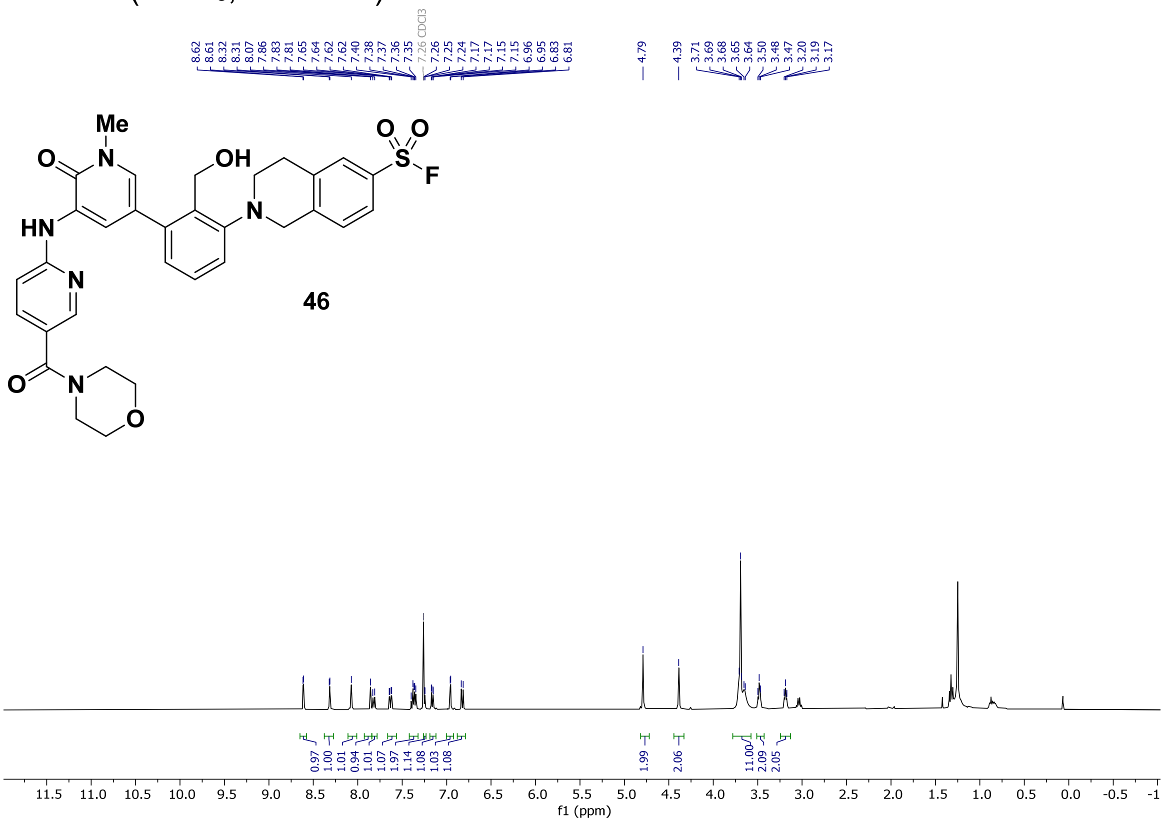

^{13}C NMR ($CDCl_3$; 101 MHz)

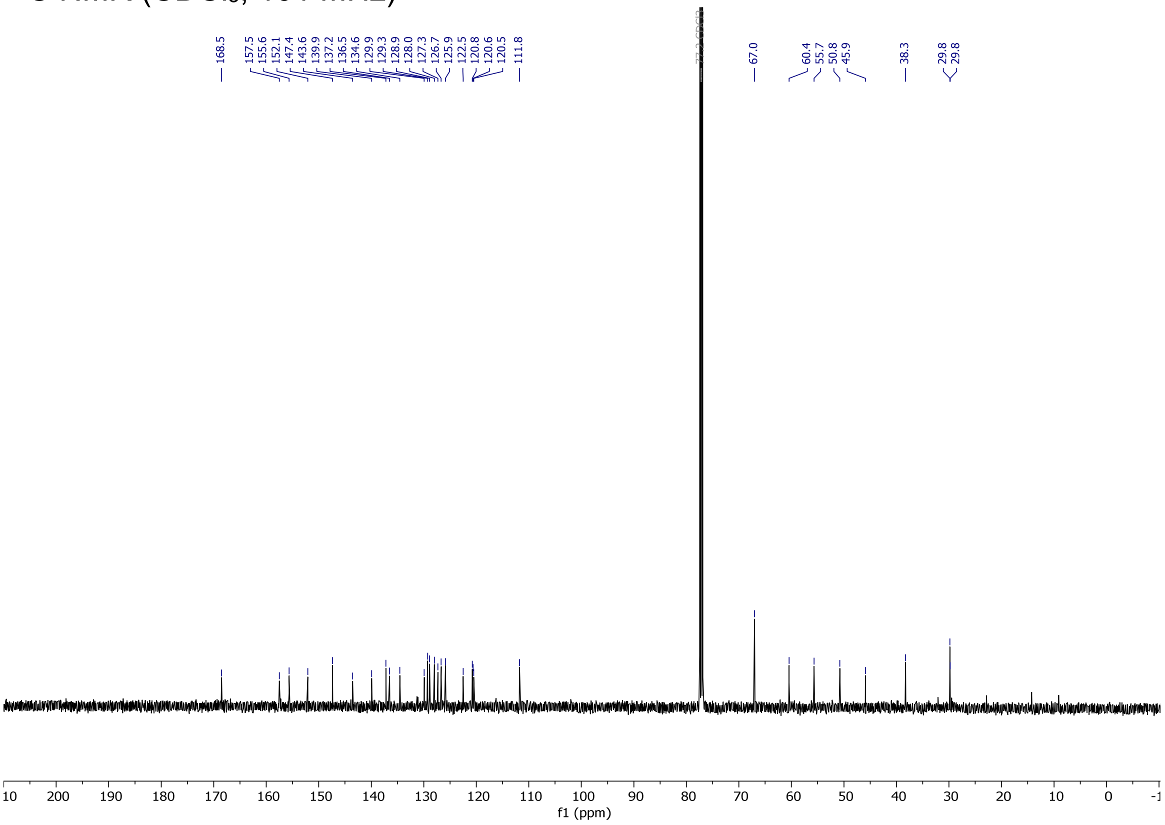

^{19}F NMR ($CDCl_3$; 376 MHz)

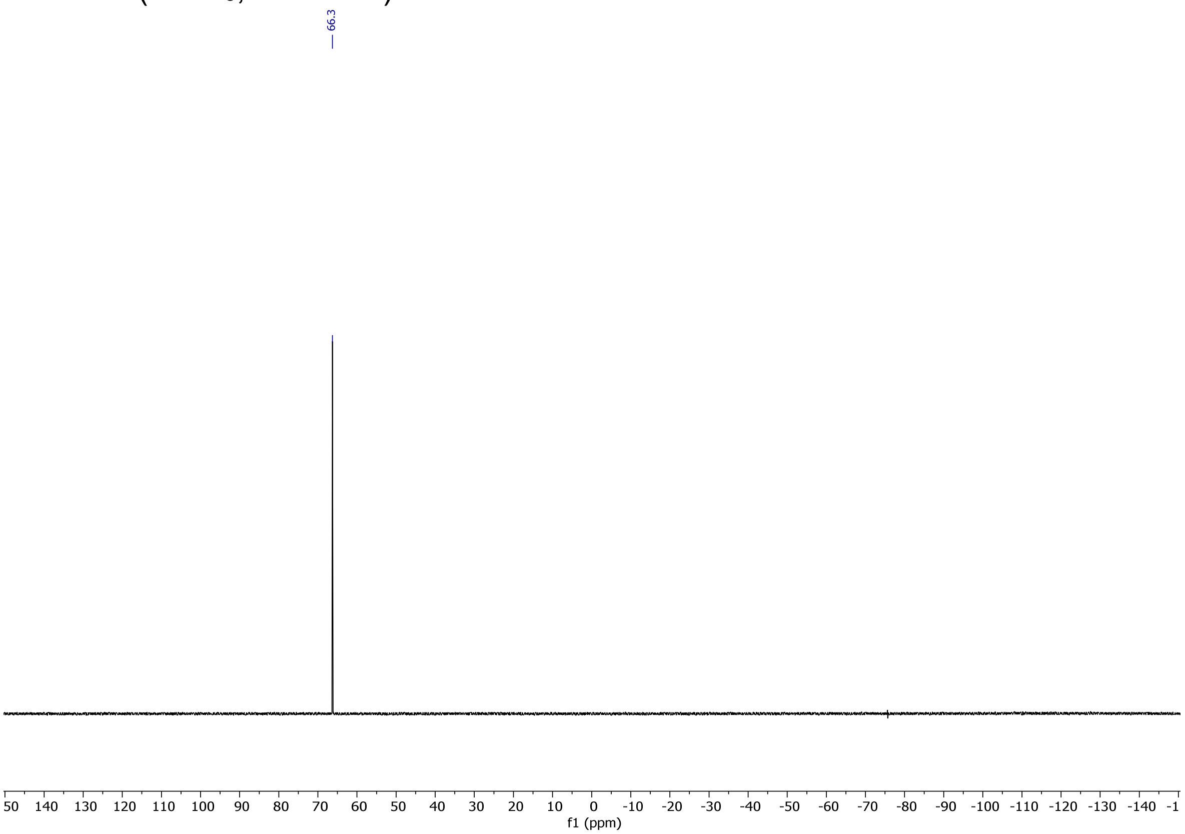

References:

(1) Sasano, Y.; Kogure, N.; Nagasawa, S.; Kasabata, K.; Iwabuchi, Y. 2-Azaadamantane N-oxyl (AZADO)/Cu Catalysis Enables Chemoselective Aerobic Oxidation of Alcohols Containing Electron-Rich Divalent Sulfur Functionalities. *Organic letters* **2018**, *20* (19), 6104-6107. DOI: 10.1021/acs.orglett.8b02528.
(2) Baskin, J. M.; Wang, Z. A mild, convenient synthesis of sulfinic acid salts and sulfonamides from alkyl and aryl halides. *Tetrahedron Lett.* **2002**, *43* (47), 8479-8483. DOI: 10.1016/s0040-4039(02)02073-7.
(3) Shavnya, A.; Hesp, K. D.; Tsai, A. S. A Versatile Reagent and Method for Direct Aliphatic Sulfonylation. *Adv. Synth. Catal.* **2018**, *360* (9), 1768-1774. DOI: 10.1002/adsc.201800071.
(4) Kim, D. K.; Um, H. S.; Park, H.; Kim, S.; Choi, J.; Lee, C. Silyloxymethanesulfinate as a sulfoxylate equivalent for the modular synthesis of sulfones and sulfonyl derivatives. *Chem. Sci.* **2020**, *11* (48), 13071-13078. DOI: 10.1039/d0sc02947e.
(5) Higham, J. I.; Bull, J. A. Copper catalysed oxidative α-sulfonylation of branched aldehydes using the acid enhanced reactivity of manganese(iv) oxide. *Chemical Communications* **2020**, *56* (33), 4587-4590, 10.1039/D0CC01576H. DOI: 10.1039/D0CC01576H.
(6) Greed, S.; Briggs, E. L.; Idiris, F. I. M.; White, A. J. P.; Lücking, U.; Bull, J. A. Synthesis of Highly Enantioenriched Sulfonimidoyl Fluorides and Sulfonimidamides by Stereospecific Sulfur–Fluorine Exchange (SuFEx) Reaction. **2020**, *26* (55), 12533-12538. DOI: https://doi.org/10.1002/chem.202002265.
(7) Zhang, L.; Cheng, X.; Zhou, Q.-L. Electrochemical Synthesis of Sulfonyl Fluorides with Triethylamine Hydrofluoride. **2022**, *40* (14), 1687-1692. DOI: https://doi.org/10.1002/cjoc.202200112.
(8) Tang, L.; Yang, Y.; Wen, L.; Yang, X.; Wang, Z. Catalyst-free radical fluorination of sulfonyl hydrazides in water. *Green Chemistry* **2016**, *18* (5), 1224-1228, 10.1039/C5GC02755A. DOI: 10.1039/C5GC02755A.
(9) Klan, P.; Solomek, T.; Bochet, C. G.; Blanc, A.; Givens, R.; Rubina, M.; Popik, V.; Kostikov, A.; Wirz, J. Photoremovable protecting groups in chemistry and biology: reaction mechanisms and efficacy. *Chem Rev* **2013**, *113* (1), 119-191. DOI: 10.1021/cr300177k.
(10) Wang, P. Photolabile Protecting Groups: Structure and Reactivity. *Asian J. Org. Chem.* **2013**, *2* (6), 452-464. DOI: 10.1002/ajoc.201200197 (acccessed 2025-04-15T17:31:23). Barltrop, J. A.; Plant, P. J.; Schofield, P. Photosensitive protective groups. *Chemical Communications (London)* **1966**, (22), 822. DOI: 10.1039/c19660000822 (acccessed 2025-04-15T17:34:16).
(11) Piloto, A. M.; Costa, S. P. G.; Gonçalves, M. S. T. Wavelength-selective cleavage of o-nitrobenzyl and polyheteroaromatic benzyl protecting groups. *Tetrahedron* **2014**, *70* (3), 650-657. DOI: 10.1016/j.tet.2013.11.100.
(12) Peng, Y.; Wang, Z.; Chen, Y.; Xu, W.; Hu, Y.; Chen, Z.; Xu, J.; Wu, Q. Photoinduced Promiscuity of Cyclohexanone Monooxygenase for the Enantioselective Synthesis of alpha-Fluoroketones. *Angew. Chem. Int. Ed.* **2022**, *61* (50), e202211199. DOI: 10.1002/anie.202211199 From NLM Medline.
(13) Hong, J.-B.; Davidson, J. P.; Jin, Q.; Lee, G. R.; Matchett, M.; O'Brien, E.; Welch, M.; Bingenheimer, B.; Sarma, K. Development of a Scalable Synthesis of a Bruton's Tyrosine Kinase Inhibitor via C–N and C–C Bond Couplings as an End Game Strategy. *Org. Process Res. Dev.* **2014**, *18* (1), 228-238. DOI: 10.1021/op4001077 (acccessed 2023-09-28T18:33:51).
